# Identifying the mediating role of socioeconomic status on the relationship between schizophrenia and major depressive disorder: a Mendelian randomisation analysis

**DOI:** 10.1038/s41537-023-00389-2

**Published:** 2023-08-29

**Authors:** Qiang Xu, Mengjing Cai, Yuan Ji, Juanwei Ma, Jiawei Liu, Qiyu Zhao, Yayuan Chen, Yao Zhao, Yijing Zhang, He Wang, Lining Guo, Kaizhong Xue, Zirui Wang, Mengge Liu, Chunyang Wang, Dan Zhu, Feng Liu

**Affiliations:** 1 Department of Radiology and Tianjin Key Laboratory of Functional Imaging, Tianjin Medical University General Hospital, Tianjin, China; 2 Department of Radiology, Tianjin Medical University Cancer Institute and Hospital, Tianjin, China; 3 Department of Scientific Research, Tianjin Medical University General Hospital, Tianjin, China; 4 Department of Radiology, Tianjin Medical University General Hospital Airport Hospital, Tianjin, China

**Keywords:** Schizophrenia, Psychosis

## Abstract

Depressive disorder prevalence in patients with schizophrenia has been reported to be 40%. People with low socioeconomic status (SES) are more likely to suffer from schizophrenia and major depressive disorder (MDD). However, the causal relationship between schizophrenia and depression and the potential mediating role of SES remains unclear. Two-sample Mendelian randomization (MR) analyses were conducted to explore the bidirectional causal relationship between schizophrenia and MDD with the largest sample size of European ancestry from public genome-wide association studies (sample size ranged from 130,644 to 480,359). Inverse variance weighted (IVW) method was used as the primary analysis, and several canonical MR methods were used as validation analyses. The mediating role of SES (educational years, household income, employment status, and Townsend deprivation index) was estimated by the two-step MR method. MR analyses showed that genetically predicted schizophrenia was associated with an increased risk of MDD (IVW odds ratio [OR] = 1.137 [95% CI 1.095, 1.181]). Reversely, MDD was also associated with an increased risk of schizophrenia (IVW OR = 1.323 [95% CI 1.118, 1.565]). The mediation analysis via the two-step MR method revealed that the causal effect of schizophrenia on MDD was partly mediated by the Townsend deprivation index with a proportion of 10.27%, but no significant mediation effect was found of SES on the causal effect of MDD on schizophrenia. These results suggest a robust bidirectional causal effect between schizophrenia and MDD. Patients with schizophrenia could benefit from the early and effective intervention of the Townsend deprivation index.

## Introduction

Schizophrenia and major depressive disorder (MDD) have long been viewed as two distinct psychiatric disorders. However, it is worth noting that MDD is a common co-occurring condition in people with schizophrenia. Additionally, the negative symptoms of schizophrenia overlap with those of MDD in multiple aspects, such as diminished emotional expression, flat affect, and lack of interest in social interactions^1,2^. People with schizophrenia are at an increased risk of developing MDD (the lifetime prevalence is estimated to be 28.6% (95% CI: 25.3–32.2%) compared with the general population^3^. Schizophrenia comorbid with MDD usually predicts adverse outcomes, and the worst of which is suicide; these patients also have a lower quality of life than those who do not suffer from depression^4,5^. In addition, people with MDD often experience symptoms of psychosis, including delusions and hallucinations^6^. Schizophrenia and MDD lead to significant impacts on the global healthcare burden, and therefore, effective prevention and treatment strategies are urgently needed.

Socioeconomic status (SES) is a term used to describe a person’s economic and social position relative to others, and it is commonly measured by educational years, income, employment status, and Townsend deprivation index (TDI)^7,8^. Of which, TDI is a composite measure of socioeconomic deprivation based on four variables: unemployment, non-home ownership, non-car ownership, and overcrowded households, with a higher score indicating a higher degree of deprivation. There has been sufficient evidence that low SES is associated with poor health outcomes^9,10^. Recently, researchers have paid increasing attention to the relationships between SES and mental health, including not only less prevalent disorders like schizophrenia but more common ones like MDD. Some observational epidemiological studies have found that people with low SES are likely to suffer from schizophrenia or MDD^11–15^ and revealed that both disorders are genetically correlated with SES^16^. As mentioned above, individuals with schizophrenia appear to be more susceptible to MDD than the general population, and we, therefore, speculate that this association is likely mediated by SES^3,17^. Identifying the mediating effect of SES might help to develop targeted and feasible strategies for early intervention of schizophrenia and reduce poor outcomes. However, it is unclear whether there is a causal association between schizophrenia and MDD, the direction of this relationship, and the effect of SES on this association.

Randomized controlled trials (RCTs) are considered the gold standard to support causal inference in epidemiological studies^18^. Nevertheless, some RCTs are difficult to implement owing to time, cost, medical ethics, and other problems. Observational studies are widely used for their relatively simple design and ease of implementation, but reverse causality or potential confounding factors can bias the results. As a consequence, the findings may be controversial and unable to be confirmed by experimental studies^19,20^. In recent years, Mendelian randomization (MR) provides an effective way to solve these problems. It utilizes genetic variants (i.e., single nucleotide polymorphisms, SNPs) as instrumental variables (IVs) to evaluate the causal effect of a modifiable exposure on a disease outcome^21,22^. According to Mendel’s laws of inheritance, those exposure-related genetic variants are randomly allocated at conception and are relatively independent, and MR is thereby able to minimize bias from confounders and reverse causality^23,24^. Benefiting from the explosion of genome-wide association studies (GWAS), large-scale public GWASs summary statistics can be used in MR studies for causal inference^25^. Despite the presence of MR studies between risk factors and psychiatric diseases^26,27^, no one has studied the causal relationship between SCZ and MDD. In the present study, the aim was mainly twofold: we conducted MR analyses to investigate the causal relationship between schizophrenia and MDD, and if so, whether SES could mediate the causal relationship.

## Methods and materials

MR analysis can yield valid causal estimates if the following assumptions are met^28,29^: (i) the variants are significantly associated with the exposure, (ii) the variants are not associated with the outcome via confounding pathways, and (iii) the variants cannot affect the outcome directly, only possibly indirectly via the exposure. To reduce the impact of population stratification^24^, we conducted MR analysis based on summary data from public GWASs of European ancestry with the largest sample size (Table S1). In brief, bidirectional two-sample MR analyses were first performed to assess the associations between schizophrenia and MDD, and two-step MR analysis was then performed to investigate the mediating effects of SES on these associations.

### Data sources

#### Schizophrenia

The GWAS summary data regarding schizophrenia were obtained from a recent study^18^, and the summary statistics of European ancestry samples were selected to construct genetic IVs, including 53,386 cases and 77,258 controls. All included schizophrenia patients were diagnosed according to general international criteria (e.g., ICD-10 or DSM-IV).

#### MDD

The summary-level data were obtained from a prior GWAS of European ancestry for MDD^30^, including 135,458 cases and 344,901 controls. The MDD cases were diagnosed according to general international criteria (e.g., ICD-10 or DSM-IV).

#### SES

In the present study, four indicators of SES including educational years, household income, employment status, and TDI were used as potential mediators for the causal association between schizophrenia and MDD. Genetic IVs for educational years were obtained from summary-level data of a published GWAS restricted to European ancestry with 766,345 individuals^31^. IVs for household income, employment status, and TDI were derived from IEU OpenGWAS project datasets (https://gwas.mrcieu.ac.uk/), with sample sizes of 397,751, 461,242, and 462,464, respectively.

All datasets for the exposure, mediators, and outcome should be non-overlapping as sample overlap can increase weak instrument bias^32^. Considering that both SES and MDD GWAS datasets included samples from UK Biobank, as well as the data availability of 23andMe cohort in the MDD GWAS dataset as an outcome in MR analysis, we thus used the data that excluded these two cohorts (UK Biobank: 29,740 subjects; 23andMe: 307,354 subjects) in the outcome dataset to avoid sample overlap in schizophrenia-mediators-MDD relationship, and 143,265 subjects (45,591 cases and 97,674 controls) were finally included. For the MR analysis with MDD as exposure, as only two independent SNPs (rs76025409 and rs1950829) survived with *p* < 5 × 10^−8^ in the MDD GWAS dataset excluding UK Biobank and 23andMe, we used the original GWAS dataset (135,458 cases and 344,901 controls) to increase the statistical power^30^. The sample information about the three datasets (i.e., schizophrenia, MDD, and SES) is presented in Supplementary Methods and Table S1.

### Genetic instrument selection

For all MR analyses, we constructed IVs using independent GWAS loci of European-ancestry summary data with a threshold of *p* < 5 × 10^−8^, as suggested by previous studies^33,34^. Then, the SNPs in these loci were clumped for independence using PLINK clumping method^35^. The independence among SNPs was defined as pairwise linkage disequilibrium (LD) *r*^2^ < 0.001 with a clumping window of 10,000 kb. For SNPs in LD, we retained the ones with the lowest *p*-value. If there were no SNPs for exposure in the outcome datasets, we replaced them with their proxy SNPs (*r*^2^ > 0.8); LD proxies were defined using 1000 Genomes European sample data^36^. Subsequently, we harmonized the effect alleles of these variants both in exposure and outcome datasets. After that, palindromic SNPs with intermediate allele frequencies (> 0.3) were removed. Steiger filtering was also performed to remove any SNPs which explained more variance in the outcome than the exposure to eliminate the MR bias of reverse causation^37^. The remaining SNPs were finally used for MR analysis.

### Testing instrument strength and statistical power

The *F* statistics of each IV were calculated according to previous studies^38,39^, representing the strength of associations between genetic IVs and exposure. To minimize weak instrument bias, IVs with *F* statistics > 10 were retained for subsequent analyses^40,41^. We also used an online tool (https://sb452.shinyapps.io/power/)^42^ for power calculation, and power > 80% was considered sufficient. Please see Supplementary Methods for details.

### Bidirectional univariable MR analyses

The random-effects inverse variance weighted (IVW) method was used for primary MR analysis to estimate bidirectional causal associations between schizophrenia and MDD. Specifically, the Wald ratio method uses a single IV to estimate the causal effect of exposure on outcome, and the IVW method utilized a meta-analytic approach to combine the Wald ratio estimates into a pooled causal estimate of the exposure-outcome association^43^. The IVW method could provide an unbiased estimation in the absence of horizontal pleiotropy or under the assumption of balanced pleiotropy^44^. The significance level for the associations between schizophrenia and MDD was set at *p* < 0.05.

In addition, other robust MR methods including MR-Egger, and weighted median were also used for causal inference. MR-Egger tests the exposure-outcome association adjusted for directional pleiotropy, which estimates the average pleiotropic effect across the genetic variants, and provides consistent causal estimates under the Instrument Strength Independent of Direct Effect (InSIDE) assumption; when all IVs violate the exclusion restriction assumption (assumption (iii)), it could still provide an unbiased estimate^45^. When no measurement error (NOME) assessed with *I*^2^ statistic was violated (i.e., *I*^2^ statistic < 0.9), MR-Egger with simulation extrapolation (SIMEX) correction would be used, which is based on the simulation of SNP-exposure association estimates to create new datasets^38,46^. The weighted median method gives valid causal estimates with up to 50% of the weight coming from invalid IVs, while all IVs are required to be valid in IVW method^47^.

### Mediation analysis

A two-step MR approach was used to assess the mediating effect of SES indicators between exposure (schizophrenia/MDD) and outcome (MDD/schizophrenia). In the first step, we used IVs for schizophrenia/MDD and performed a two-sample univariable MR analysis to estimate its causal influence on SES. In the second step, we conducted the two-sample multivariable MR analysis to estimate the independent effect of each exposure on the outcome while controlling for other exposures^48^. Specifically, the IVs from the related GWASs (four SES indicators and schizophrenia/MDD) were first combined and pruned by LD (*r*^2^ < 0.001) with a clumping window of 10,000 kb to ensure that the SNPs were independent, then the SNP effects and corresponding standard errors were extracted from the SES and schizophrenia/MDD GWAS summary statistics and harmonized with the outcome MDD/schizophrenia GWAS datasets, and finally, all these five exposures were simultaneously incorporated into two-sample multivariable MR model to estimate the independent causal effect of each exposure on outcome (MDD/schizophrenia). The multivariable MR extensions of the IVW method^49^ and MR-Egger method^50^ were used in the current analysis. For each mediator, a product of the coefficients method was utilized to estimate the indirect effect^51^ (i.e., the effect of schizophrenia/MDD on MDD/schizophrenia through the mediator). Standard errors for the indirect effect were derived by the delta method using the estimates obtained from two-sample MR analyses^52,53^.

#### Sensitivity analyses

Sensitivity analyses were performed to assess the robustness of our findings. Specifically, the heterogeneity test, horizontal pleiotropy test, Mendelian randomization pleiotropy residual sum and outlier (MR-PRESSO) test, and leave-one-out analysis were used. First, the heterogeneity among IVs was measured by Cochran’s *Q* statistic for IVW analyses and Rücker’s *Q* statistic for MR-Egger, respectively. Second, we performed MR-Egger regression to evaluate potential directional horizontal pleiotropy, whose intercept term deviating from zero was considered evidence of directional pleiotropic bias. Third, the MR-PRESSO test was also performed to identify possible horizontal pleiotropy by its MR-PRESSO global test^54^. If pleiotropy is present, the MR-PRESSO outlier test would be performed to identify outliers among IVs and calculate MR estimates after removing outliers, thereby eliminating detected pleiotropy. MR-PRESSO distortion test was conducted to assess the difference in MR estimates before and after outlier correction. We presented outlier-adjusted causal estimates when both global and distortion tests were significant. Finally, leave-one-out analyses were conducted to find whether the estimate was driven or biased by a single SNP. The significance levels of the heterogeneity test, MR-Egger intercept test, and MR-PRESSO test were set at *p* < 0.05.

All statistical analyses were conducted using the TwoSampleMR^55^ (version 0.5.6, https://mrcieu.github.io/TwoSampleMR), MendelianRandomization (version 0.6.0), and MRPRESSO^54^ (version 1.0) packages in *R* (version 4.1.3).

## Results

The genetic instruments used in our study are shown in Tables S2–S23. The *I*^2^ values for the evaluation of NOME assumption in MR analyses are shown in Table S24, and for those < 0.9, we performed SIMEX corrections to replace the MR-Egger results.

### Bidirectional univariable MR analyses between schizophrenia and MDD

**Fig. 1:**
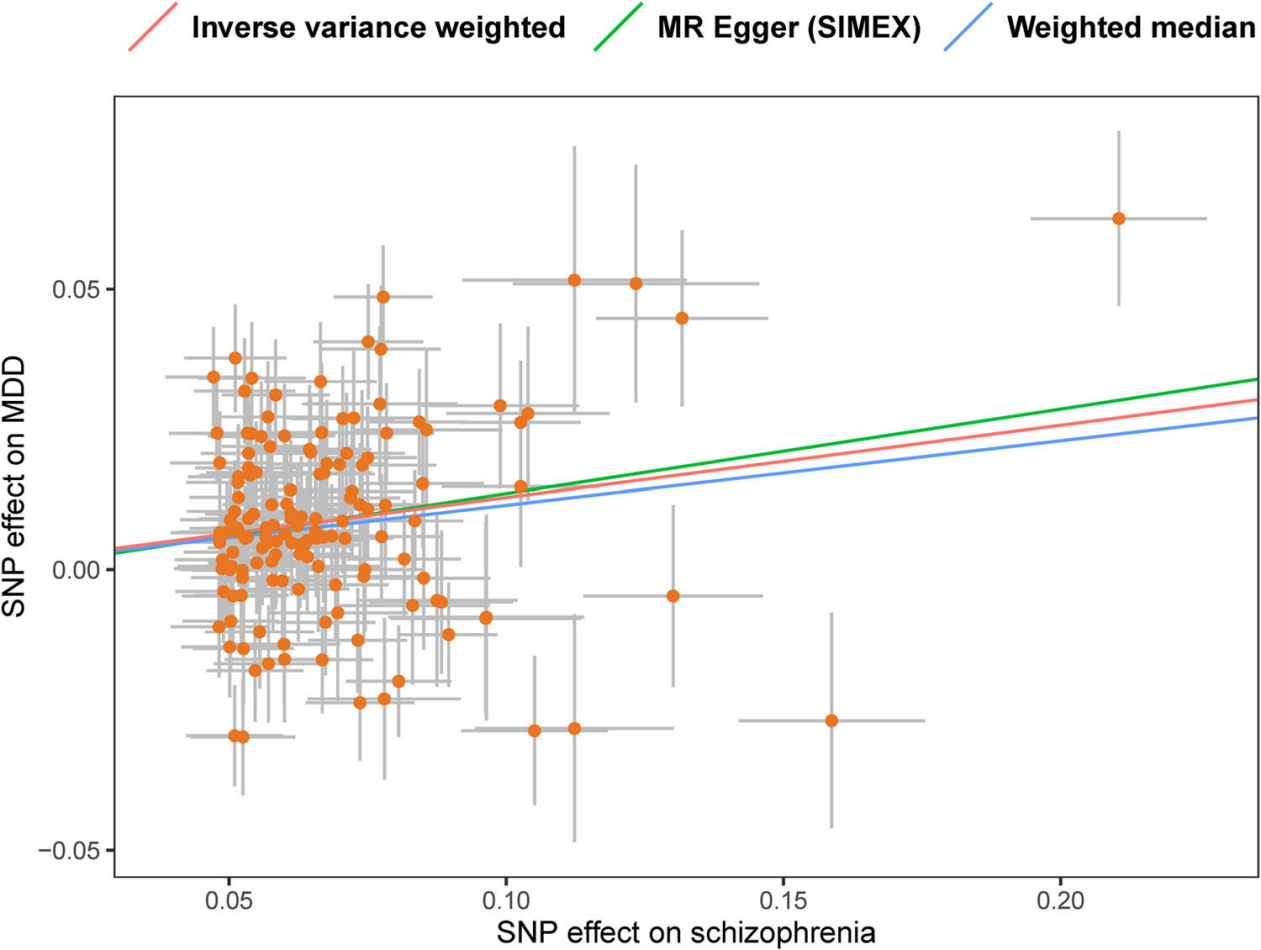
Scatter plot for the causal effect of schizophrenia on MDD. In the scatter plot, each dot (orange) represents an SNP, and the error bars (gray) at each dot represent the 95% confidence intervals. The horizontal axis is the SNP effect on exposure (schizophrenia), while the vertical axis is the SNP effect on outcome (MDD). The three fitted lines (colors) represent the results of MR under three methods (shown in the top panel), with the slope of each line corresponding to the estimated causal effect for each method. MDD major depressive disorder, MR Mendelian randomization, SIMEX simulation extrapolation, SNP single nucleotide polymorphism.

We performed a two-sample MR analysis with 146 independent SNPs associated with schizophrenia (Tables S2, S3) to estimate the causal effect of schizophrenia on MDD. The SNPs that were used as IVs for MDD and SCZ (derived from each GWAS, respectively) were independent. The results showed that schizophrenia increased the risk of MDD (IVW OR = 1.137, 95% CI: 1.095–1.181, *p* = 2.37 × 10^−11^), and the causal effect across other MR methods were consistent (Table 1). There was some evidence of heterogeneity (IVW *Qp* = 1.26 × 10^−15^, Egger *Qp* = 8.83 × 10^−16^, Table S25). The symmetric funnel plot and MR-Egger intercept indicated no directional pleiotropy (*p* = 0.772, Fig. S1 and Table S25). The scatter plot and leave-one-out analysis did not reveal any influential outliers (Fig. 1 and Table S26). MR-PRESSO identified two outliers (rs12129573, rs3795310), and the causal estimate was not significantly changed after removing the outliers (OR = 1.135, 95% CI: 1.094–1.177, *p* = 8.24 × 10^−12^, Table 1; MR-PRESSO distortion test *p* = 0.910). In the MR-Steiger test, there was no evidence of reverse causality (Table S25).

**Table 1:** Univariable MR results with schizophrenia as the exposure.

Outcome	Method	SNPs	Beta	SE	Lower CI	Upper CI	OR	Lower CI	Upper CI	*p*-value
MDD	MR Egger (SIMEX)	146	0.180	0.080	0.023	0.336	1.197	1.023	1.400	0.026
	Weighted median	146	0.115	0.022	0.072	0.157	1.122	1.075	1.170	1.13E−07
	Inverse variance weighted	146	0.129	0.019	0.091	0.166	1.137	1.095	1.181	2.37E−11
	MR-PRESSO outlier-corrected	144	0.126	0.018	0.090	0.163	1.135	1.094	1.177	8.24E−12
Educational years	MR Egger (SIMEX)	147	0.013	0.029	−0.045	0.070	1.013	0.956	1.072	0.668
	Weighted median	147	0.004	0.005	−0.006	0.013	1.004	0.994	1.013	0.434
	Inverse variance weighted	147	0.003	0.007	−0.012	0.017	1.003	0.989	1.017	0.699
	MR-PRESSO outlier-corrected	–	–	–	–	–	–	–	–	–
Household income	MR Egger (SIMEX)	146	−0.047	0.025	−0.097	0.002	0.954	0.908	1.002	0.064
	Weighted median	146	−0.028	0.007	−0.041	−0.015	0.972	0.960	0.985	1.80E−05
	Inverse variance weighted	146	−0.037	0.007	−0.050	−0.023	0.964	0.951	0.977	6.20E−08
	MR-PRESSO outlier-corrected	138	−0.025	0.006	−0.036	−0.014	0.975	0.965	0.986	9.18E−06
Employment status	MR Egger (SIMEX)	146	−0.015	0.007	−0.029	−1.70E−04	0.985	0.971	0.99983	0.049
	Weighted median	146	−0.007	0.002	−0.012	−0.002	0.993	0.988	0.998	0.003
	Inverse variance weighted	146	−0.008	0.002	−0.012	−0.005	0.992	0.988	0.995	4.05E−06
	MR-PRESSO outlier-corrected	145	−0.008	0.002	−0.011	−0.004	0.992	0.989	0.996	9.51E−06
TDI	MR Egger (SIMEX)	146	−0.026	0.019	−0.063	0.011	0.974	0.939	1.011	0.172
	Weighted median	146	0.016	0.005	0.007	0.026	1.016	1.007	1.026	0.001
	Inverse variance weighted	146	0.021	0.005	0.012	0.030	1.021	1.012	1.031	4.13E−06
	MR-PRESSO outlier-corrected	138	0.022	0.004	0.014	0.030	1.022	1.014	1.030	3.26E−08

When investigating the causal effect of schizophrenia on education years, MR-PRESSO outlier correction was not performed due to the non-significant MR results found by other MR methods.

*CI* confidence interval, *MDD* major depressive disorder, *MR-PRESSO* Mendelian randomization pleiotropy residual sum and outlier, *OR* odds ratio, *SE* standard error, *SES* socioeconomic status, *SIMEX* simulation extrapolation, *SNP* single nucleotide polymorphism, *TDI* Townsend deprivation index.

With MDD as the exposure, we also performed a two-sample MR analysis to estimate its causal effect on schizophrenia. The results showed that MDD was associated with a higher risk of schizophrenia (IVW OR = 1.323, 95% CI: 1.118–1.565, *p* = 0.001; Table S27). The funnel plot and MR-Egger intercept test suggested that there was no significant directional pleiotropy (*p* = 0.242, Fig. S2 and Table S25), but significant heterogeneity among IVs was found (IVW *Qp* = 0.020; Egger *Qp* = 0.028, Table S25). MR-PRESSO identified one outlier (rs9427672), and the causal estimate was not significantly changed (IVW OR = 1.387, 95% CI: 1.207–1.593, *p* = 3.74 × 10^−6^, Table S27; MR-PRESSO distortion test *p* = 0.585) after outlier removal. There were no influential outliers in both scatter plots and leave-one-out analyses (Fig. S3 and Table S28), and the MR-Steiger test suggested that there was no reverse causality (Table S25). All IVs used in the analysis are shown in Tables S14, S15.

### Mediation analysis

**Fig. 2:**
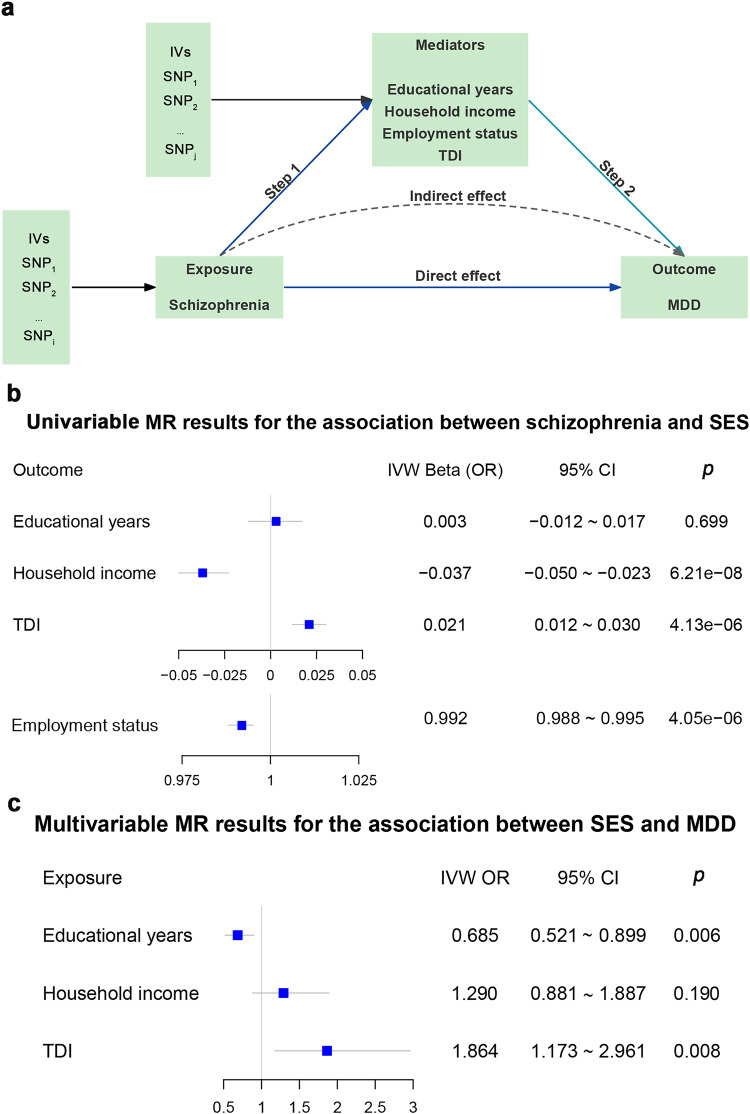
Mediation analysis of the causal effect of schizophrenia on MDD via potential mediators. **a** The framework for two-step MR analysis. In step 1, we used IVs for schizophrenia to estimate its causal effect on potential mediators (SES, including educational years, household income, employment status, and TDI); in step 2, we used IVs for SES to estimate the causal effect of potential mediators on MDD conditioning on schizophrenia. “Direct effect” refers to the effect of schizophrenia on MDD after adjusting for the mediators. “Indirect effect” refers to the effect of schizophrenia on MDD via the mediator, namely the mediating effect. **b** Univariable MR results for the association between schizophrenia and SES. **c** Multivariable MR results for the association between SES and MDD conditioning on schizophrenia in one model. In **b** and **c**, the squares (blue) represent causal estimates (IVW OR for binary outcomes, IVW β for continuous outcomes), and the error bars (gray) represent 95% CI. CI confidence interval, IVs instrumental variables, IVW inverse variance weighted, MDD major depressive disorder, MR Mendelian randomization, OR odds ratio, SES socioeconomic status, SNP single nucleotide polymorphism, TDI Townsend deprivation index.

To assess the mediating role of SES indicators between exposure (schizophrenia/MDD) and outcome (MDD/schizophrenia), a two-step MR analysis was performed (Fig. 2a).

In the first step, we performed a univariable two-sample MR analysis to investigate the causal relationships of schizophrenia/MDD to each potential SES mediator (Fig. 2b and Table 1). For the analysis of schizophrenia as exposure, we found that schizophrenia was causally associated with low income (IVW *β* = −0.037, 95% CI: −0.050 to −0.023, *p* = 6.20 × 10^−8^), poor employment status (IVW OR = 0.992, 95% CI: 0.988–0.995, *p* = 4.05 × 10^−6^), high TDI (IVW *β* = 0.021, 95% CI: 0.012–0.030, *p* = 4.13 × 10^−6^), but not causally associated with educational years (IVW *β* = 0.003, 95% CI: −0.012 to 0.017, *p* = 0.699). The results of heterogeneity tests and pleiotropy tests are presented in Table S25. Symmetric funnel plot indicated no directional pleiotropy (Figs. S4–S6). In the schizophrenia-income relationship, MR-PRESSO identified eight outliers (rs10117, rs11210892, rs12883788, rs13107325, rs4632195, rs60135207, rs6546857, and rs708228), and the causal estimate was still significant after removing these outliers (*β* = −0.025, 95% CI: −0.036 to −0.014, *p* = 9.18 × 10^−6^; MR-PRESSO distortion test *p* = 0.016). In the schizophrenia-employment status relationship, after removing outlier rs3814883, the result was not significantly changed (*β* = −0.008, 95% CI: −0.011 to −0.004, *p* = 9.51 × 10^−6^; MR-PRESSO distortion test *p* = 0.737). In the schizophrenia-TDI relationship, the causal estimate was not significantly changed (*β* = 0.022, 95% CI: 0.014–0.030, *p* = 3.26 × 10^−8^; MR-PRESSO distortion test *p* = 0.847) after removing eight outliers (rs1000237, rs11191580, rs11534045, rs11664298, rs12883788, rs1604060, rs61937595, and rs9636107). Neither the scatter plots nor the leave-one-out analysis revealed any influential outliers (Figs. S7–S9 and Tables S29–S31), and no reverse causality was found across these analyses in MR-Steiger tests (Table S25). In addition, we also separately examined the relationships between MDD and four SES indicators and found no significant causal associations (all *p*-values > 0.05, Tables S25 and S27). The IVs used in the schizophrenia-SES and MDD-SES MR analyses are shown in Tables S4–S11 and Tables S16–S23, respectively.

In the second step, we performed multivariable MR analysis to investigate the causal relationships of SES to MDD/schizophrenia. For the analysis of MDD as an outcome, there was no remaining SNP of employment status after LD clumping among IVs of four SES indicators and schizophrenia exposures. Therefore, with 269 SNPs (Tables S12–S13), we estimated the independent effect of each of the remaining three SES indicators (educational years, household income, and TDI) on MDD while controlling for the other two SES indicators and schizophrenia (Table 2 and Fig. 2c). We found causal relationships between educational years and MDD (IVW OR = 0.685, 95% CI: 0.521–0.899, *p* = 0.006) as well as between TDI and MDD (IVW OR = 1.864, 95% CI: 1.173–2.961, *p* = 0.008). In the sensitivity analyses (Table S32), we found significant heterogeneity (IVW *Qp* = 1.31 × 10^−12^, Egger *Qp* = 1.18 × 10^−12^) but no pleiotropy (*p* = 0.491). Notably, in the first step of exploring the mediating effect of SES in the MDD–schizophrenia association, we found that MDD was not causally associated with SES indicators, and thus the multivariable MR analysis of causal effects of SES on schizophrenia was unnecessary. Finally, the indirect effect of schizophrenia-SES-MDD was estimated using the product of coefficients method, and a significant mediating effect of TDI (*β* = 0.013, 95% CI: 0.002–0.025, Table 3) was found with a mediation proportion of 10.27%.

**Table 2:** Multivariable MR results for the causal effect of SES on MDD.

Exposures	Total SNPs^a^	IVW				MR Egger			
		OR	Lower CI	Upper CI	*p*-value	OR	Lower CI	Upper CI	*p*-value
Schizophrenia	269	1.136	1.090	1.183	1.12E−09	1.135	1.089	1.182	1.45E−09
Educational years		0.685	0.521	0.899	0.006	0.758	0.509	1.129	0.173
Household income		1.290	0.881	1.887	0.190	1.273	0.868	1.867	0.216
TDI		1.864	1.173	2.961	0.008	1.857	1.168	2.952	0.009

^a^Less than the total number of IVs because there is overlap between the IVs sets. There was no remaining SNP of employment status after clumping LD among IVs of four SES indicators and schizophrenia exposures.

*CI* confidence interval, *IVW* inverse variance weighted, *MDD* major depressive disorder, *MR* Mendelian randomization, *OR* odds ratio, *SES* socioeconomic status, *SNP* single nucleotide polymorphism.

**Table 3:** The mediation effect of TDI on the causal effect of schizophrenia on MDD.

Mediator	Total effect (*β*)	Direct effect *a*	Direct effect *b*	Indirect effect (*β*)	95% CI	Mediation proportion
TDI	0.129	0.021	0.623	0.013	(0.002, 0.025)	10.27%

The “Total effect” refers to the effect of schizophrenia on depression; “Direct effect *a*” refers to the effect of schizophrenia on TDI; “Direct effect *b*” refers to the effect of TDI on depression conditioning on schizophrenia; “Indirect effect” refers to the effect of schizophrenia on depression via TDI, namely the mediating effect of TDI.

*CI* confidence interval, *MDD* major depressive disorder, *TDI* Townsend deprivation index.

### Instrument strength and statistical power analysis

The explained variance (*R*^2^) and *F* statistics for each IV are shown in Tables S2−S23. All *F* statistics > 10 indicated that there was no weak instrumental bias. As the power analysis indicated, we had sufficient power to detect the relationships between schizophrenia and MDD (99.9%), between schizophrenia and income (99.9%)/TDI (89.1%), while low power (< 80%) to detect the relationships between MDD and schizophrenia/SES. Additionally, we also had enough power to detect the relationships between SES (except for employment status) and schizophrenia. Detailed results of power analyses are shown in Table S33.

## Discussion

To the best of our knowledge, this study is the first to investigate the bidirectional causal relationships between schizophrenia and MDD, as well as the mediating role of TDI on the causal effect of schizophrenia on MDD. These results were largely consistent across MR sensitivity analyses, including different MR methods, leave-one-out analysis, and MR-PRESSO analysis, suggesting that horizontal pleiotropy is unlikely to be an adequate explanation for our results.

Patients with schizophrenia have an increased risk of suffering from MDD^56^. Conversely, patients with MDD have also been shown to be at a higher risk of developing psychosis^57^. Genetic correlation analysis showed a shared genetic risk between schizophrenia and MDD^58^. However, these correlations may arise from pleiotropy (i.e., genes independently affecting both schizophrenia and MDD). MR analysis could provide evidence for the causal effect of disease exposure if key assumptions relating to instrument validity are met. Our MR results exhibited the robust causal effect of schizophrenia on MDD, and an increased risk of schizophrenia was associated with a higher risk of MDD. Bidirectional MR analysis, with genetic liability for schizophrenia as an outcome, indicated that the genetic liability for MDD is also a possible causal risk factor of schizophrenia. Previous research reported several lines of evidence that there is a mutual relationship of risk between schizophrenia and MDD, suggesting potential overlap in the pathophysiology of the two disorders^59–62^. Negative symptoms like anhedonia, anergia, and avolition, which are typically seen in individuals with schizophrenia, are also commonly observed in those with MDD^63^. Additionally, ~15–19% of individuals diagnosed with MDD may experience hallucinations and/or delusions^57^.

Birchwood and colleagues have put forward a potential pathway linking schizophrenia to MDD, in which patients may feel stigmatized and socially marginalized due to their psychotic status^64^. This perception may contribute to the development of MDD, and the impact of SES factors is considered to mediate the relationship between schizophrenia and MDD. In the present study, we conducted a two-step MR for mediation analysis and found that the causal effect of schizophrenia on MDD was partially mediated by socioeconomic deprivation (10.27%), but not income or educational years. In the first MR step, MR analysis identified a causal relationship that genetically predicted schizophrenia was associated with an increased TDI (*β* = 0.021) and fewer job opportunities (OR = 0.99). Studies have reported that patients with schizophrenia are more likely to reside in areas characterized by higher social deprivation and occupy lower socioeconomic positions^65^. Interestingly, we did not find a causal effect of schizophrenia on education years through large-scale MR design. This seems to be at odds with a general view that the level of education in schizophrenia is lower than in normal population^66^, however, it should be pointed out that the observed relationships in traditional epidemiological research can be constrained by sample size, reverse causality, and confounding effects. Social drift hypothesis claimed that patients with schizophrenia suffered from downward social mobility, including residing in deprived areas and with unemployment status after experiencing psychotic-like symptoms^7^. The previous studies showed evidence that polygenic risk for schizophrenia was statistically significant with area deprivation^67^, which is consistent with our first-step estimate. The second MR step provided evidence that genetically predicted higher TDI (OR = 1.865) and shorter educational years (OR = 0.685) were independently associated with a higher risk of MDD. There was substantial evidence that lower SES (education, occupation, income, and TDI) is associated with a higher risk of MDD^68–71^, while an MR analysis provided the support that MDD did not affect educational attainment, household income, or TDI^72^. These results are consistent with the second step estimate of our mediation analysis and MR analysis with MDD as exposure and SES as outcomes.

This two-sample bidirectional MR study that investigated the relationship between genetic liability for schizophrenia and MDD risk had several strengths. First, by utilizing the largest available GWAS summary statistics, we were able to include the maximum number of instruments for the exposures, thus increasing our statistical power. Second, because all datasets were limited to individuals of European ancestry, population stratification was not an issue in our study. Third, MR analysis offers the advantage of being able to identify causal relationships without being skewed by reverse causation and confounding. Strong evidence supported the bidirectional causal relationships of schizophrenia and MDD, which in turn enabled us to comprehend the shared symptoms of both conditions. Finally, we found that the comorbidity of schizophrenia with MDD is perhaps partly mediated by TDI. Therefore, by decreasing socioeconomic deprivation, it is possible to reduce the risk of comorbid MDD in schizophrenia, offering a possible avenue for interventions to improve mental health outcomes in this population.

There are several limitations of our study that merit consideration. First, when exploring the causal effect of MDD on SES, the UK Biobank cohort was included in both MDD and SES GWAS datasets, which, although with less overlap (<10%), may still bias the MR estimates. Second, significant heterogeneity was observed in MR results. Despite the fact that we used the random-effects IVW method to alleviate this influence, the effect may not be fully ruled out. Third, the non-significant MR results (e.g., the causal relationship of MDD on SES) may be partially due to insufficient statistical power (Table S33). A larger sample size is needed to confirm our findings in the future. Fourth, to minimize bias from population stratification, our study was restricted to individuals of European ancestry, which may limit the generalizability of our findings to other populations. Finally, the MR approach provides a genetically predicted causal relationship estimate between exposure and outcome in non-experimental data; however, genetic variation reflects lifelong exposure to risk factors, which may differ from the effects of clinical intervention. RCTs are needed to further confirm this causal effect.

In conclusion, this study employed the largest exposure and outcome GWASs datasets to conduct MR analysis to infer a causal relationship between schizophrenia and MDD. We found robust genetic evidence for a risk association between schizophrenia and MDD, and TDI mediated the causal relationship of schizophrenia on MDD. The potential implications of our results deepened the understanding of the mechanisms underlying comorbid MDD in schizophrenia and increased effective therapeutic attention to mood symptoms in well-powered randomized clinical trials.

### Supplementary information

Supplementary Material

## Data Availability

This study used deidentified publicly available summary-level data. Analysis scripts are available on request from Q.X. (xuqiang@tmu.edu.cn).

## References

[1] Castle D, Bosanac P (2012). Depression and schizophrenia. Adv. Psychiatr. Treat..

[2] Samsom JN, Wong AH (2015). Schizophrenia and depression co-morbidity: what we have learned from animal models. Front. Psychiatry.

[3] Li W (2020). Prevalence of comorbid depression in schizophrenia: a meta-analysis of observational studies. J. Affect. Disord..

[4] Conley RR, Ascher-Svanum H, Zhu B, Faries DE, Kinon BJ (2007). The burden of depressive symptoms in the long-term treatment of patients with schizophrenia. Schizophr. Res..

[5] Dutta R, Murray RM, Allardyce J, Jones PB, Boydell J (2011). Early risk factors for suicide in an epidemiological first episode psychosis cohort. Schizophr. Res..

[6] Dubovsky SL, Ghosh BM, Serotte JC, Cranwell V (2021). Psychotic depression: diagnosis, differential diagnosis, and treatment. Psychother. Psychosom..

[7] Goldberg EM, Morrison SL (1963). Schizophrenia and social class. Br. J. Psychiatry.

[8] Dai J, Xu Y, Wang T, Zeng P (2023). Exploring the relationship between socioeconomic deprivation index and Alzheimer’s disease using summary-level data: from genetic correlation to causality. Prog. Neuropsychopharmacol. Biol. Psychiatry.

[9] Matthews KA, Gallo LC (2011). Psychological perspectives on pathways linking socioeconomic status and physical health. Annu. Rev. Psychol..

[10] Kivimäki M (2020). Association between socioeconomic status and the development of mental and physical health conditions in adulthood: a multi-cohort study. Lancet Public Health.

[11] Burns JK, Tomita A, Kapadia AS (2014). Income inequality and schizophrenia: increased schizophrenia incidence in countries with high levels of income inequality. Int. J. Soc. Psychiatry.

[12] Werner S, Malaspina D, Rabinowitz J (2007). Socioeconomic status at birth is associated with risk of schizophrenia: population-based multilevel study. Schizophr. Bull..

[13] Agerbo E (2015). Polygenic risk score, parental socioeconomic status, family history of psychiatric disorders, and the risk for schizophrenia: a Danish Population-Based Study and Meta-analysis. JAMA Psychiatry.

[14] Weich S, Lewis G (1998). Poverty, unemployment, and common mental disorders: population based cohort study. BMJ.

[15] Saraceno B, Levav I, Kohn R (2005). The public mental health significance of research on socio-economic factors in schizophrenia and major depression. World Psychiatry.

[16] Marees AT (2021). Genetic correlates of socio-economic status influence the pattern of shared heritability across mental health traits. Nat. Hum. Behav..

[17] Upthegrove R, Marwaha S, Birchwood M (2017). Depression and schizophrenia: cause, consequence, or trans-diagnostic issue?. Schizophr. Bull..

[18] Trubetskoy V (2022). Mapping genomic loci implicates genes and synaptic biology in schizophrenia. Nature.

[19] Jones DS, Podolsky SH (2015). The history and fate of the gold standard. Lancet.

[20] Lin LJ, Wei YY, Zhang RY, Chen F (2019). Application of Mendelian randomization methods in causal inference of observational study. Chin. J. Prev. Med..

[21] Smith GD, Ebrahim S (2003). ‘Mendelian randomization’: can genetic epidemiology contribute to understanding environmental determinants of disease?. Int. J. Epidemiol..

[22] Emdin CA, Khera AV, Kathiresan S (2017). Mendelian randomization. JAMA.

[23] Burgess S (2019). Guidelines for performing Mendelian randomization investigations. Wellcome Open Res..

[24] Lawlor DA, Harbord RM, Sterne JA, Timpson N, Davey Smith G (2008). Mendelian randomization: using genes as instruments for making causal inferences in epidemiology. Stat. Med..

[25] Zheng J (2017). Recent developments in Mendelian randomization studies. Curr. Epidemiol. Rep..

[26] Saccaro LF, Gasparini S, Rutigliano G (2022). Applications of Mendelian randomization in psychiatry: a comprehensive systematic review. Psychiatr. Genet..

[27] Zhu D (2022). Total brain volumetric measures and schizophrenia risk: a two-sample Mendelian Randomization Study. Front. Genet..

[28] Didelez V, Sheehan N (2007). Mendelian randomization as an instrumental variable approach to causal inference. Stat. Methods Med. Res..

[29] Davies NM, Holmes MV, Davey Smith G (2018). Reading Mendelian randomisation studies: a guide, glossary, and checklist for clinicians. BMJ.

[30] Wray NR (2018). Genome-wide association analyses identify 44 risk variants and refine the genetic architecture of major depression. Nat. Genet..

[31] Lee JJ (2018). Gene discovery and polygenic prediction from a genome-wide association study of educational attainment in 1.1 million individuals. Nat. Genet..

[32] Burgess S, Davies NM, Thompson SG (2016). Bias due to participant overlap in two-sample Mendelian randomization. Genet. Epidemiol..

[33] Dudbridge F, Gusnanto A (2008). Estimation of significance thresholds for genomewide association scans. Genet. Epidemiol..

[34] Uffelmann E (2021). Genome-wide association studies. Nat. Rev. Methods Primers.

[35] Purcell S (2007). PLINK: a tool set for whole-genome association and population-based linkage analyses. Am. J. Hum. Genet..

[36] Machiela MJ, Chanock SJ (2015). LDlink: a web-based application for exploring population-specific haplotype structure and linking correlated alleles of possible functional variants. Bioinformatics.

[37] Hemani G, Tilling K, Davey Smith G (2017). Orienting the causal relationship between imprecisely measured traits using GWAS summary data. PLoS Genet..

[38] Gormley M (2020). A multivariable Mendelian randomization analysis investigating smoking and alcohol consumption in oral and oropharyngeal cancer. Nat. Commun..

[39] Wootton RE (2018). Evaluation of the causal effects between subjective wellbeing and cardiometabolic health: Mendelian randomisation study. BMJ.

[40] Burgess S, Thompson SG (2011). Avoiding bias from weak instruments in Mendelian randomization studies. Int. J. Epidemiol..

[41] Pierce BL, Ahsan H, Vanderweele TJ (2011). Power and instrument strength requirements for Mendelian randomization studies using multiple genetic variants. Int. J. Epidemiol..

[42] Burgess S (2014). Sample size and power calculations in Mendelian randomization with a single instrumental variable and a binary outcome. Int. J. Epidemiol..

[43] Bowden J (2017). A framework for the investigation of pleiotropy in two-sample summary data Mendelian randomization. Stat. Med..

[44] Hemani G, Bowden J, Davey Smith G (2018). Evaluating the potential role of pleiotropy in Mendelian randomization studies. Hum. Mol. Genet..

[45] Bowden J, Davey Smith G, Burgess S (2015). Mendelian randomization with invalid instruments: effect estimation and bias detection through Egger regression. Int. J. Epidemiol..

[46] Bowden J (2016). Assessing the suitability of summary data for two-sample Mendelian randomization analyses using MR-Egger regression: the role of the I2 statistic. Int. J. Epidemiol..

[47] Bowden J, Davey Smith G, Haycock PC, Burgess S (2016). Consistent estimation in Mendelian randomization with some invalid instruments using a weighted median estimator. Genet. Epidemiol..

[48] Sanderson E, Davey Smith G, Windmeijer F, Bowden J (2019). An examination of multivariable Mendelian randomization in the single-sample and two-sample summary data settings. Int. J. Epidemiol..

[49] Burgess S, Thompson SG (2015). Multivariable Mendelian randomization: the use of pleiotropic genetic variants to estimate causal effects. Am. J. Epidemiol..

[50] Rees JMB, Wood AM, Burgess S (2017). Extending the MR-Egger method for multivariable Mendelian randomization to correct for both measured and unmeasured pleiotropy. Stat. Med..

[51] VanderWeele TJ (2016). Mediation analysis: a practitioner’s guide. Annu. Rev. Public Health.

[52] Carter AR (2019). Understanding the consequences of education inequality on cardiovascular disease: Mendelian randomisation study. BMJ.

[53] Cheung MW (2009). Comparison of methods for constructing confidence intervals of standardized indirect effects. Behav. Res. Methods.

[54] Verbanck M, Chen CY, Neale B, Do R (2018). Detection of widespread horizontal pleiotropy in causal relationships inferred from Mendelian randomization between complex traits and diseases. Nat. Genet..

[55] Hemani G (2018). The MR-Base platform supports systematic causal inference across the human phenome. Elife.

[56] Buckley PF, Miller BJ, Lehrer DS, Castle DJ (2009). Psychiatric comorbidities and schizophrenia. Schizophr. Bull..

[57] Ohayon MM, Schatzberg AF (2002). Prevalence of depressive episodes with psychotic features in the general population. Am. J. Psychiatry.

[58] Lee SH, Cross-Disorder Group of the Psychiatric Genomics, C. (2013). Genetic relationship between five psychiatric disorders estimated from genome-wide SNPs. Nat. Genet..

[59] Sonmez N, Romm KL, Andreasssen OA, Melle I, Rossberg JI (2013). Depressive symptoms in first episode psychosis: a one-year follow-up study. BMC Psychiatry.

[60] McGlashan TH, Carpenter WT (1976). Postpsychotic depression in schizophrenia. Arch. Gen. Psychiatry.

[61] Park SC (2014). Distinctive clinical correlates of psychotic major depression: the CRESCEND Study. Psychiatry Investig..

[62] Gournellis R, Oulis P, Howard R (2014). Psychotic major depression in older people: a systematic review. Int. J. Geriatr. Psychiatry.

[63] Krynicki CR, Upthegrove R, Deakin JFW, Barnes TRE (2018). The relationship between negative symptoms and depression in schizophrenia: a systematic review. Acta Psychiatr. Scand..

[64] Birchwood M, Iqbal Z, Upthegrove R (2005). Psychological pathways to depression in schizophrenia: studies in acute psychosis, post psychotic depression and auditory hallucinations. Eur. Arch. Psychiatry Clin. Neurosci..

[65] Byrne M, Agerbo E, Eaton WW, Mortensen PB (2004). Parental socio-economic status and risk of first admission with schizophrenia—a Danish national register based study. Soc. Psychiatry Psychiatr. Epidemiol..

[66] Tesli M (2022). Educational attainment and mortality in schizophrenia. Acta Psychiatr. Scand..

[67] Sariaslan A (2016). Schizophrenia and subsequent neighborhood deprivation: revisiting the social drift hypothesis using population, twin and molecular genetic data. Transl. Psychiatry.

[68] Freeman A (2016). The role of socio-economic status in depression: results from the COURAGE (aging survey in Europe). BMC Public Health.

[69] Hoebel J, Maske UE, Zeeb H, Lampert T (2017). Social inequalities and depressive symptoms in adults: the role of objective and subjective socioeconomic status. PLoS ONE.

[70] Ye J (2021). Socioeconomic deprivation index is associated with psychiatric disorders: an observational and genome-wide gene-by-environment interaction analysis in the UK Biobank Cohort. Biol. Psychiatry.

[71] Cai J (2022). Socioeconomic status, individual behaviors and risk for mental disorders: a Mendelian randomization study. Eur. Psychiatry.

[72] Campbell D (2022). Effects of depression on employment and social outcomes: a Mendelian randomisation study. J. Epidemiol. Community Health.

