# Supplementary material for: Identifying the mediating role of socioeconomic status on the relationship between schizophrenia and major depressive disorder: a Mendelian randomisation analysis
Source: Schizophrenia (Heidelb). 2023 Aug 29;9(1):53. doi: 10.1038/s41537-023-00389-2 (PMC10465573; doi:10.1038/s41537-023-00389-2)
Supplement: Supplementary file 1 — Supplementary Material [file 41537_2023_389_MOESM1_ESM.docx]

**Supplementary Materials**

**Identifying the mediating role of socioeconomic status on the relationship between schizophrenia and major depressive disorder:** **a Mendelian randomisation analysis**

Content

[Supplementary Methods 4](#_Toc143628611)

[Data sources 4](#_Toc143628612)

[Testing instrument strength and statistical power 5](#_Toc143628613)

[Supplementary Tables 7](#_Toc143628614)

[Table S1. Data sources and sample sizes of MR analyses. 7](#_Toc143628615)

[Table S2. Summary information on genetic instruments for schizophrenia-MDD MR analysis in schizophrenia GWAS dataset. 8](#_Toc143628616)

[Table S3. Summary information on genetic instruments for schizophrenia-MDD MR analysis in MDD GWAS dataset. 14](#_Toc143628617)

[Table S4. Summary information on genetic instruments for schizophrenia-educational years MR analysis in schizophrenia GWAS dataset. 20](#_Toc143628618)

[Table S5. Summary information on genetic instruments for schizophrenia-educational years MR analysis in educational years GWAS dataset. 26](#_Toc143628619)

[Table S6. Summary information on genetic instruments for schizophrenia-income MR analysis in schizophrenia GWAS dataset. 32](#_Toc143628620)

[Table S7. Summary information on genetic instruments for schizophrenia-income MR analysis in income GWAS dataset. 38](#_Toc143628621)

[Table S8. Summary information on genetic instruments for schizophrenia-employment status MR analysis in schizophrenia GWAS dataset. 44](#_Toc143628622)

[Table S9. Summary information on genetic instruments for schizophrenia-employment status MR analysis in employment status GWAS dataset. 50](#_Toc143628623)

[Table S10. Summary information on genetic instruments for schizophrenia-TDI MR analysis in schizophrenia GWAS dataset. 56](#_Toc143628624)

[Table S11. Summary information on genetic instruments for schizophrenia-TDI MR analysis in TDI GWAS dataset. 62](#_Toc143628625)

[Table S12. Summary information on genetic instruments for SES-MDD multivariable MR analysis in GWAS datasets of the exposures. 68](#_Toc143628626)

[Table S13. Summary information on genetic instruments for SES-MDD multivariable MR analysis in MDD GWAS dataset. 78](#_Toc143628627)

[Table S14. Summary information on genetic instruments for MDD-schizophrenia MR analysis in MDD GWAS dataset. 88](#_Toc143628628)

[Table S15. Summary information on genetic instruments for MDD-schizophrenia MR analysis in schizophrenia GWAS dataset. 89](#_Toc143628629)

[Table S16. Summary information on genetic instruments for MDD-educational years MR analysis in MDD GWAS dataset. 90](#_Toc143628630)

[Table S17. Summary information on genetic instruments for MDD-educational years MR analysis in educational years GWAS dataset. 92](#_Toc143628631)

[Table S18. Summary information on genetic instruments for MDD-income MR analysis in MDD GWAS dataset. 94](#_Toc143628632)

[Table S19. Summary information on genetic instruments for MDD-income MR analysis in income GWAS dataset. 96](#_Toc143628633)

[Table S20. Summary information on genetic instruments for MDD-employment status MR analysis in MDD GWAS dataset. 98](#_Toc143628634)

[Table S21. Summary information on genetic instruments for MDD-employment status MR analysis in employment status GWAS dataset. 100](#_Toc143628635)

[Table S22. Summary information on genetic instruments for MDD-TDI MR analysis in MDD GWAS dataset. 102](#_Toc143628636)

[Table S23. Summary information on genetic instruments for MDD-TDI MR analysis in TDI GWAS dataset. 104](#_Toc143628637)

[Table S24. *I*^2^ values for the evaluation of NOME assumption in MR analyses. 106](#_Toc143628638)

[Table S25. Results for sensitivity analyses and Steiger tests in univariable MR analyses. 107](#_Toc143628639)

[Table S26. MR leave-one-out sensitivity analysis for causal effect of schizophrenia on MDD. 108](#_Toc143628640)

[Table S27. Univariable MR results with MDD as the exposure. 114](#_Toc143628641)

[Table S28. MR leave-one-out sensitivity analysis for causal effect of MDD on schizophrenia. 115](#_Toc143628642)

[Table S29. MR leave-one-out sensitivity analysis for causal effect of schizophrenia on employment status. 116](#_Toc143628643)

[Table S30. MR leave-one-out sensitivity analysis for causal effect of schizophrenia on household income. 119](#_Toc143628644)

[Table S31. MR leave-one-out sensitivity analysis for causal effect of schizophrenia on TDI. 122](#_Toc143628645)

[Table S32. Results for sensitivity analyses in multivariable MR analysis. 126](#_Toc143628646)

[Table S33. The statistical power for the univariable MR analyses. 127](#_Toc143628647)

[Supplementary Figures 128](#_Toc143628648)

[Fig. S1. Funnel plot for the causal effect of schizophrenia on MDD. 128](#_Toc143628649)

[Fig. S2. Funnel plot for the causal effect of MDD on schizophrenia. 129](#_Toc143628650)

[Fig. S3. Scatter plot for the causal effect of MDD on schizophrenia. 130](#_Toc143628651)

[Fig. S4. Funnel plot for the causal effect of schizophrenia on household income. 131](#_Toc143628652)

[Fig. S5. Funnel plot for the causal effect of schizophrenia on employment status. 132](#_Toc143628653)

[Fig. S6. Funnel plot for the causal effect of schizophrenia on TDI. 133](#_Toc143628654)

[Fig. S7. Scatter plot for the causal effect of schizophrenia on household income. 134](#_Toc143628655)

[Fig. S8. Scatter plot for the causal effect of schizophrenia on employment status. 135](#_Toc143628656)

[Fig. S9. Scatter plot for the causal effect of schizophrenia on TDI. 136](#_Toc143628657)

[References 137](#_Toc143628658)

# Supplementary Methods

## Data sources

*Socioeconomic status (SES)*

1. Genotyping and imputation of the MRC-Integrative Epidemiology Unit (IEU) UK Biobank genome wide association study (GWAS) pipeline. The dataset included a total of 488,377 samples, of which 49,979 individuals were genotyped using the UK BiLEVE array and 438,398 using the UK Biobank axiom array. Pre-imputation quality control, phasing, and imputation are described elsewhere ^1^. In short, multiallelic single nucleotide polymorphisms (SNPs) or those with minor allele frequency (MAF) ≤ 1% were removed before phasing. The phasing of genotype data was performed with an improved version of the SHAPEIT2 algorithm ^2^. IMPUTE2 algorithms ^3^ were used to perform genotype imputation to the reference set which combines the UK10K haplotype and Haplotype Reference Consortium (HRC) reference panels ^4^. These analyses were limited to autosomal variants within the HRC site list using a graded filtering with varying imputation quality for different allele frequency ranges. Hence, rarer genetic variants are required to have higher imputation INFO scores (INFO > 0.3 for MAF > 3%; INFO > 0.6 for MAF 1%-3%; INFO > 0.8 for MAF 0.5%-1%; INFO > 0.9 for MAF 0.1%-0.5%) with MAF and INFO scores being recalculated on the internally derived European subset^5^.

2. Data quality control

Sex-mismatched individuals (obtained by comparing genetic sex with reported sex), individuals with sex-chromosome aneuploidy as well as outliers in heterozygosity and missing rates were excluded from the analysis (*n* = 1,812). The sample was restricted to European ancestry as defined by an internal *k*-means cluster analysis performed with the first four principal components provided by UK Biobank in *R*. The present analysis includes the largest cluster from this analysis (*n* = 464,708) ^5^.

3. Association analysis: statistical methods

GWAS was conducted using the linear mixed model (LMM) association method implemented in BOLT-LMM (v2.3) ^6^. To simulate population structure in the sample, 143,006 SNPs of direct genotype were used, which were obtained after filtering on MAF > 0.01, genotyping rate > 0.015, Hardy-Weinberg equilibrium *p* < 0.0001, and LD pruning to an *r*^2^ threshold of 0.1 using PLINK v2.00. Genotype array and sex were adjusted in the model. Since BOLT-LMM association statistics are linear, test statistics (*β* and standard error) were therefore transformed to log odds ratios and corresponding 95% confidence intervals on the liability scale using the Taylor transformation expansion series ^7^.

4. GWAS of SES in UK Biobank of IEU OpenGWAS project datasets

GWAS of three SES indicators (household income, employment status, and Townsend deprivation index [TDI]) were performed in European participants from UK Biobank recruited at initial assessment (in 2006-2010) based on internal *k*-means cluster analysis using the first four principal components after standard exclusions, including withdrawn consent (up to 2017-08-31), mismatch between genetic and reported gender (*n* = 378), putative sex chromosomes are not XX or XY (*n* = 652), and outliers in heterozygosity and missing rates (*n* = 968). Finally, 1,812 non-overlapping individuals were excluded. SNPs associated with each of the SES indicators were identified using the BOLT-LMM (linear mixed model) software v2.3. Gender and genotypic array (the UK Biobank axiom array or the UK BiLEVE array) were adjusted in the analyses. BOLT-LMM algorithm tests the association between genotypes and phenotypes, where population stratification and relatedness are explained by the LMM. Compared with other approaches for analyzing UK Biobank data, this method could achieve higher power under the condition of maximum sample inclusions ^7^.

## Testing instrument strength and statistical power

In the univariable two-sample MR analysis, the strength of instrument variables can be measured by *F*-statistics; however, we were unable to calculate conditional *F* statistics to assess the strength of instrument variables in multivariable MR analysis due to sample overlapping in multiple exposure summary-level data sources ^8^. In addition, the power calculation for the main inverse variance weighted (IVW) analyses of bidirectional MR between schizophrenia and depression was performed using an online power calculation tool (https://sb452.shinyapps.io/power/) ^9^. Briefly, parameters of sample size in outcome GWAS, the ratio of cases to controls for binary outcome, causal effect (odds ratio for binary outcome/*β* for continuous outcome) and *R*^2^ by total IVs for the exposure were required. The power greater than 80% was considered sufficient

# Supplementary Tables

## Table S1. Data sources and sample sizes of MR analyses.

| **Phenotype definition of exposures and outcomes** | **Publication/Source** | **Sample size** | **Variable type** | **Ancestry** | **ID** |
| --- | --- | --- | --- | --- | --- |
| ***Socioeconomic status*** |  |  |  |  |  |
| Years of schooling | Lee JJ et al., 2018, Nature Genetics ^10^ | 766,345 | Continuous | European | ieu-a-1239 |
| Household income before tax | Elsworth B, et al., 2018, MRC IEU OpenGWAS database ^11,12^ | 397,751 | Categorical ordered | European | ukb-b-7408 |
| Employment status: in paid employment or self-employed | Elsworth B, et al., 2018, MRC IEU OpenGWAS database ^11,12^ | 461,242 | Binary | European | ukb-b-6740 |
| Townsend deprivation index | Elsworth B, et al., 2018, MRC IEU OpenGWAS database ^11,12^ | 462,464 | Continuous | European | ukb-b-10011 |
| ***Psychiatric disorders*** |  |  |  |  |  |
| Schizophrenia | Trubetskoy V, et al., 2022, Nature ^13^ | 130,644 (53,386 cases and 77,258 controls) | Binary | European | NA |
| Major depressive disorder (outcome)* | Wray NR, et al., 2018, Nature Genetics ^14^ | 143,265 (45,591 cases and 97,674 controls) | Binary | European | NA |
| Major depressive disorder (exposure) | Wray NR, et al., 2018, Nature Genetics ^14^ | 480,359 (135,458 cases and 344,901 controls) | Binary | European | NA |

Note: *indicates that when MDD was taken as outcome, we used the GWAS summary-level statistics excluding UK biobank (29,740 subjects) and 23andMe (307,354 subjects). Considering that both SES and MDD GWAS datasets included samples from UK Biobank, as well as the data availability of 23andMe cohort in the MDD GWAS dataset as outcome in MR analysis, we thus used the data that excluded these two cohorts (UK Biobank: 29,740 subjects, 23andMe: 307,354 subjects) in the outcome dataset to avoid overlap in schizophrenia-mediators-MDD relationship, and 143,265 subjects (45,591 cases and 97,674 controls) were finally included. Since MR analyses were conducted based on summary-level multicenter data, please refer to the corresponding literature for more detailed demographic information.

Abbreviations: ID, identification; MR, Mendelian randomisation; NA, not available.

## Table S2. Summary information on genetic instruments for schizophrenia-MDD MR analysis in schizophrenia GWAS dataset.

| **SNP** | **Effect Allele** | **Alternate Allele** | **EAF** | **Beta** | **SE** | ***p* value** | ***R*^2^** | ***F* statistics** | **Overall *R*^2^** | **Mean *F* statistics** |
| --- | --- | --- | --- | --- | --- | --- | --- | --- | --- | --- |
| rs1000237 | T | A | 0.627 | -0.073 | 0.009 | 2.80E-16 | 0.0005 | 67.656 | 0.050 | 44.562 |
| rs10035564 | A | G | 0.650 | -0.067 | 0.009 | 4.38E-13 | 0.0004 | 52.724 |  |  |
| rs10086619 | A | G | 0.831 | -0.072 | 0.012 | 4.97E-10 | 0.0003 | 38.745 |  |  |
| rs10108980 | C | T | 0.785 | -0.063 | 0.011 | 2.73E-09 | 0.0003 | 35.102 |  |  |
| rs10117 | G | A | 0.613 | 0.055 | 0.009 | 4.66E-10 | 0.0003 | 39.062 |  |  |
| rs10861176 | G | A | 0.257 | -0.056 | 0.010 | 1.59E-08 | 0.0002 | 32.075 |  |  |
| rs10876446 | G | C | 0.674 | -0.054 | 0.009 | 1.03E-08 | 0.0003 | 33.004 |  |  |
| rs11027839 | A | C | 0.488 | -0.052 | 0.009 | 2.40E-09 | 0.0003 | 35.866 |  |  |
| rs11136325 | G | A | 0.436 | 0.054 | 0.009 | 3.05E-09 | 0.0003 | 34.948 |  |  |
| rs11165867 | C | T | 0.829 | -0.074 | 0.012 | 1.30E-10 | 0.0003 | 41.030 |  |  |
| rs11191580 | T | C | 0.920 | 0.132 | 0.016 | 1.77E-17 | 0.0006 | 72.199 |  |  |
| rs11210892 | G | A | 0.347 | 0.064 | 0.009 | 2.68E-12 | 0.0004 | 48.694 |  |  |
| rs11223774 | A | G | 0.301 | 0.052 | 0.009 | 2.74E-08 | 0.0002 | 31.191 |  |  |
| rs113264400 | T | C | 0.948 | -0.112 | 0.020 | 2.87E-08 | 0.0002 | 30.905 |  |  |
| rs11534045 | G | A | 0.686 | 0.063 | 0.009 | 1.40E-11 | 0.0003 | 45.594 |  |  |
| rs11587347 | C | G | 0.895 | -0.104 | 0.015 | 1.53E-12 | 0.0004 | 49.952 |  |  |
| rs11664298 | G | A | 0.794 | -0.077 | 0.011 | 8.94E-13 | 0.0004 | 51.360 |  |  |
| rs11693094 | C | T | 0.556 | 0.054 | 0.009 | 4.29E-10 | 0.0003 | 39.103 |  |  |
| rs117178087 | C | T | 0.939 | 0.096 | 0.018 | 4.89E-08 | 0.0002 | 29.663 |  |  |
| rs11941714 | G | A | 0.673 | 0.052 | 0.009 | 3.07E-08 | 0.0002 | 30.780 |  |  |
| rs1198588 | A | T | 0.194 | -0.103 | 0.011 | 1.73E-21 | 0.0007 | 90.246 |  |  |
| rs12129573 | C | A | 0.616 | -0.078 | 0.009 | 2.28E-18 | 0.0006 | 76.414 |  |  |
| rs12138231 | T | A | 0.171 | -0.067 | 0.012 | 7.99E-09 | 0.0003 | 33.355 |  |  |
| rs12151767 | G | A | 0.524 | 0.061 | 0.009 | 1.31E-12 | 0.0004 | 50.484 |  |  |
| rs12285419 | C | A | 0.800 | -0.085 | 0.011 | 1.05E-14 | 0.0005 | 59.577 |  |  |
| rs12293670 | A | G | 0.678 | 0.070 | 0.009 | 1.56E-14 | 0.0004 | 58.715 |  |  |
| rs12303743 | G | C | 0.898 | -0.087 | 0.015 | 1.59E-09 | 0.0003 | 36.414 |  |  |
| rs12489270 | T | C | 0.612 | -0.058 | 0.009 | 7.47E-11 | 0.0003 | 42.330 |  |  |
| rs12652777 | T | C | 0.489 | 0.049 | 0.009 | 1.52E-08 | 0.0002 | 32.199 |  |  |
| rs12712510 | T | C | 0.488 | 0.057 | 0.009 | 5.14E-11 | 0.0003 | 43.531 |  |  |
| rs12771371 | G | A | 0.697 | 0.052 | 0.009 | 1.94E-08 | 0.0002 | 31.750 |  |  |
| rs12833624 | C | T | 0.646 | -0.050 | 0.009 | 2.77E-08 | 0.0002 | 31.111 |  |  |
| rs12877581 | G | C | 0.716 | -0.060 | 0.010 | 1.80E-09 | 0.0003 | 36.245 |  |  |
| rs12883788 | C | T | 0.527 | -0.061 | 0.009 | 1.86E-12 | 0.0004 | 49.648 |  |  |
| rs13016542 | T | C | 0.876 | 0.088 | 0.013 | 8.28E-12 | 0.0004 | 46.858 |  |  |
| rs13107325 | C | T | 0.919 | -0.159 | 0.017 | 2.90E-21 | 0.0007 | 89.238 |  |  |
| rs13195636 | A | C | 0.926 | 0.211 | 0.016 | 6.55E-40 | 0.0013 | 175.278 |  |  |
| rs13233308 | C | T | 0.526 | 0.049 | 0.009 | 1.75E-08 | 0.0002 | 32.073 |  |  |
| rs132582 | C | T | 0.471 | 0.051 | 0.009 | 3.26E-09 | 0.0003 | 35.164 |  |  |
| rs1427633 | G | C | 0.419 | 0.048 | 0.009 | 4.10E-08 | 0.0002 | 30.131 |  |  |
| rs1430894 | C | T | 0.506 | -0.053 | 0.009 | 6.15E-10 | 0.0003 | 38.404 |  |  |
| rs145071536 | T | C | 0.799 | -0.085 | 0.012 | 1.62E-12 | 0.0004 | 50.292 |  |  |
| rs1451488 | A | G | 0.432 | -0.071 | 0.009 | 4.47E-16 | 0.0005 | 66.403 |  |  |
| rs149165 | T | G | 0.569 | 0.048 | 0.009 | 3.01E-08 | 0.0002 | 30.694 |  |  |
| rs1593304 | A | G | 0.193 | -0.064 | 0.011 | 7.45E-09 | 0.0003 | 33.349 |  |  |
| rs1604060 | A | G | 0.103 | -0.077 | 0.014 | 3.24E-08 | 0.0002 | 30.411 |  |  |
| rs1615350 | C | T | 0.273 | 0.074 | 0.010 | 4.92E-14 | 0.0004 | 56.409 |  |  |
| rs167924 | A | G | 0.359 | -0.050 | 0.009 | 2.34E-08 | 0.0002 | 31.111 |  |  |
| rs16851048 | T | C | 0.792 | -0.074 | 0.011 | 4.15E-12 | 0.0004 | 48.474 |  |  |
| rs16867571 | A | G | 0.778 | 0.066 | 0.010 | 2.68E-10 | 0.0003 | 39.913 |  |  |
| rs17016552 | C | G | 0.661 | 0.052 | 0.009 | 1.20E-08 | 0.0002 | 32.278 |  |  |
| rs17194490 | G | T | 0.827 | -0.078 | 0.012 | 1.80E-11 | 0.0003 | 45.446 |  |  |
| rs17731 | G | A | 0.620 | -0.052 | 0.009 | 4.37E-09 | 0.0003 | 34.663 |  |  |
| rs187557 | C | T | 0.163 | 0.067 | 0.012 | 2.03E-08 | 0.0002 | 31.412 |  |  |
| rs1881046 | G | T | 0.670 | 0.051 | 0.009 | 3.39E-08 | 0.0002 | 30.373 |  |  |
| rs1901512 | T | C | 0.318 | 0.058 | 0.009 | 5.72E-10 | 0.0003 | 38.600 |  |  |
| rs1915019 | A | G | 0.265 | 0.057 | 0.010 | 6.57E-09 | 0.0003 | 33.947 |  |  |
| rs2053079 | A | G | 0.754 | -0.060 | 0.010 | 3.01E-09 | 0.0003 | 35.172 |  |  |
| rs2078266 | A | G | 0.178 | 0.070 | 0.013 | 2.94E-08 | 0.0002 | 30.513 |  |  |
| rs215412 | G | A | 0.661 | -0.058 | 0.009 | 2.69E-10 | 0.0003 | 40.209 |  |  |
| rs217336 | C | A | 0.583 | 0.050 | 0.009 | 8.05E-09 | 0.0003 | 33.431 |  |  |
| rs2238057 | T | G | 0.568 | -0.084 | 0.009 | 8.50E-22 | 0.0007 | 92.118 |  |  |
| rs2252074 | T | G | 0.590 | -0.069 | 0.009 | 6.19E-15 | 0.0005 | 60.599 |  |  |
| rs2332700 | C | G | 0.258 | 0.075 | 0.010 | 3.88E-14 | 0.0004 | 57.542 |  |  |
| rs2333321 | A | G | 0.218 | 0.071 | 0.011 | 1.25E-11 | 0.0004 | 45.986 |  |  |
| rs2381411 | T | C | 0.586 | -0.050 | 0.009 | 1.25E-08 | 0.0003 | 32.800 |  |  |
| rs2455415 | C | T | 0.577 | -0.049 | 0.009 | 1.69E-08 | 0.0002 | 31.634 |  |  |
| rs2456020 | C | T | 0.773 | 0.082 | 0.010 | 1.13E-15 | 0.0005 | 63.998 |  |  |
| rs2514218 | C | T | 0.668 | 0.070 | 0.009 | 1.35E-14 | 0.0004 | 58.715 |  |  |
| rs2710323 | T | C | 0.531 | 0.078 | 0.009 | 1.23E-19 | 0.0006 | 83.116 |  |  |
| rs2815731 | C | A | 0.660 | 0.060 | 0.009 | 4.39E-11 | 0.0003 | 43.478 |  |  |
| rs2909457 | G | A | 0.451 | 0.049 | 0.009 | 1.48E-08 | 0.0002 | 31.721 |  |  |
| rs2999392 | C | T | 0.299 | -0.052 | 0.009 | 3.05E-08 | 0.0002 | 30.366 |  |  |
| rs308697 | C | A | 0.574 | 0.050 | 0.009 | 8.83E-09 | 0.0003 | 33.166 |  |  |
| rs35351411 | A | C | 0.439 | -0.064 | 0.009 | 2.21E-13 | 0.0004 | 53.281 |  |  |
| rs35734242 | T | C | 0.562 | -0.051 | 0.009 | 1.37E-08 | 0.0002 | 32.457 |  |  |
| rs3739118 | G | A | 0.719 | 0.057 | 0.010 | 2.36E-09 | 0.0003 | 36.005 |  |  |
| rs3770754 | C | G | 0.645 | 0.053 | 0.009 | 5.35E-09 | 0.0003 | 33.788 |  |  |
| rs3791710 | T | C | 0.804 | 0.060 | 0.011 | 3.02E-08 | 0.0002 | 30.868 |  |  |
| rs3795310 | C | T | 0.543 | 0.051 | 0.009 | 5.75E-09 | 0.0003 | 34.360 |  |  |
| rs3802924 | A | C | 0.805 | 0.074 | 0.011 | 9.58E-12 | 0.0004 | 46.446 |  |  |
| rs3814883 | C | T | 0.548 | 0.067 | 0.009 | 1.58E-14 | 0.0005 | 59.481 |  |  |
| rs3824451 | T | C | 0.838 | -0.066 | 0.012 | 2.54E-08 | 0.0002 | 30.901 |  |  |
| rs4129585 | A | C | 0.456 | 0.075 | 0.009 | 5.11E-18 | 0.0006 | 74.309 |  |  |
| rs4575535 | A | G | 0.281 | -0.056 | 0.010 | 5.77E-09 | 0.0003 | 33.783 |  |  |
| rs4632195 | C | T | 0.471 | -0.047 | 0.009 | 4.59E-08 | 0.0002 | 30.118 |  |  |
| rs4636654 | G | A | 0.608 | 0.048 | 0.009 | 4.89E-08 | 0.0002 | 29.457 |  |  |
| rs4653164 | C | T | 0.323 | -0.051 | 0.009 | 3.08E-08 | 0.0002 | 30.855 |  |  |
| rs4702 | G | A | 0.461 | 0.084 | 0.009 | 2.79E-21 | 0.0007 | 89.726 |  |  |
| rs4766428 | C | T | 0.539 | -0.075 | 0.009 | 3.93E-17 | 0.0005 | 71.021 |  |  |
| rs4779050 | T | G | 0.381 | 0.058 | 0.009 | 7.27E-11 | 0.0003 | 42.462 |  |  |
| rs4812325 | G | A | 0.370 | -0.072 | 0.009 | 8.96E-16 | 0.0005 | 65.272 |  |  |
| rs4921741 | A | G | 0.729 | -0.056 | 0.010 | 1.21E-08 | 0.0002 | 32.652 |  |  |
| rs498591 | A | T | 0.847 | -0.072 | 0.012 | 2.11E-09 | 0.0003 | 35.896 |  |  |
| rs500102 | T | C | 0.413 | 0.052 | 0.009 | 4.87E-09 | 0.0003 | 34.516 |  |  |
| rs505061 | C | A | 0.495 | -0.053 | 0.009 | 5.80E-10 | 0.0003 | 38.694 |  |  |
| rs56205728 | G | A | 0.700 | -0.063 | 0.010 | 1.01E-10 | 0.0003 | 42.188 |  |  |
| rs56335113 | A | G | 0.316 | 0.065 | 0.009 | 6.02E-12 | 0.0004 | 47.377 |  |  |
| rs57433322 | C | G | 0.885 | 0.083 | 0.014 | 1.99E-09 | 0.0003 | 35.741 |  |  |
| rs5751191 | T | C | 0.484 | -0.066 | 0.009 | 3.00E-14 | 0.0004 | 58.176 |  |  |
| rs58120505 | T | C | 0.602 | 0.090 | 0.009 | 2.24E-24 | 0.0008 | 103.676 |  |  |
| rs60135207 | G | T | 0.596 | 0.050 | 0.009 | 1.53E-08 | 0.0002 | 31.768 |  |  |
| rs6125656 | G | A | 0.809 | -0.064 | 0.011 | 6.29E-09 | 0.0003 | 33.761 |  |  |
| rs61857878 | A | T | 0.760 | 0.060 | 0.010 | 4.44E-09 | 0.0003 | 34.715 |  |  |
| rs61937595 | C | T | 0.917 | 0.130 | 0.016 | 1.15E-15 | 0.0005 | 64.493 |  |  |
| rs62018952 | T | C | 0.264 | -0.058 | 0.010 | 1.94E-09 | 0.0003 | 36.251 |  |  |
| rs62183855 | A | C | 0.814 | 0.066 | 0.011 | 2.66E-09 | 0.0003 | 35.458 |  |  |
| rs634940 | G | T | 0.737 | -0.066 | 0.010 | 1.78E-11 | 0.0003 | 44.980 |  |  |
| rs6482437 | A | C | 0.099 | -0.099 | 0.014 | 3.33E-12 | 0.0004 | 48.512 |  |  |
| rs6520064 | A | G | 0.787 | -0.058 | 0.011 | 3.58E-08 | 0.0002 | 30.456 |  |  |
| rs6538539 | G | T | 0.462 | 0.057 | 0.009 | 4.43E-11 | 0.0003 | 43.615 |  |  |
| rs6546857 | A | G | 0.757 | -0.060 | 0.010 | 2.74E-09 | 0.0003 | 35.062 |  |  |
| rs6549963 | T | C | 0.596 | 0.048 | 0.009 | 4.31E-08 | 0.0002 | 30.131 |  |  |
| rs6673880 | A | G | 0.492 | -0.062 | 0.009 | 7.20E-12 | 0.0004 | 46.871 |  |  |
| rs6715366 | G | A | 0.723 | -0.054 | 0.010 | 2.49E-08 | 0.0002 | 31.103 |  |  |
| rs6798742 | A | G | 0.680 | -0.061 | 0.009 | 4.57E-11 | 0.0003 | 43.162 |  |  |
| rs6943762 | T | C | 0.883 | 0.105 | 0.013 | 1.57E-15 | 0.0005 | 63.394 |  |  |
| rs6974218 | A | C | 0.634 | 0.055 | 0.009 | 6.80E-10 | 0.0003 | 38.044 |  |  |
| rs6984242 | G | A | 0.413 | 0.055 | 0.009 | 3.86E-10 | 0.0003 | 39.526 |  |  |
| rs708228 | C | T | 0.661 | -0.053 | 0.009 | 6.56E-09 | 0.0003 | 33.665 |  |  |
| rs7112616 | T | C | 0.515 | 0.052 | 0.009 | 1.52E-09 | 0.0003 | 36.847 |  |  |
| rs713692 | G | A | 0.299 | -0.057 | 0.010 | 2.67E-09 | 0.0003 | 35.499 |  |  |
| rs7251 | C | G | 0.682 | 0.064 | 0.009 | 8.29E-12 | 0.0004 | 46.502 |  |  |
| rs72802868 | G | T | 0.724 | 0.069 | 0.010 | 4.55E-13 | 0.0004 | 51.959 |  |  |
| rs728055 | T | A | 0.658 | 0.067 | 0.009 | 8.85E-14 | 0.0004 | 56.078 |  |  |
| rs72943392 | G | C | 0.707 | -0.053 | 0.010 | 2.39E-08 | 0.0002 | 31.052 |  |  |
| rs72986630 | C | T | 0.926 | -0.112 | 0.018 | 3.59E-10 | 0.0003 | 39.357 |  |  |
| rs73229090 | C | A | 0.897 | 0.103 | 0.014 | 4.34E-13 | 0.0004 | 52.207 |  |  |
| rs73292401 | T | A | 0.799 | -0.068 | 0.011 | 5.48E-10 | 0.0003 | 38.468 |  |  |
| rs7515363 | C | T | 0.389 | 0.054 | 0.009 | 1.84E-09 | 0.0003 | 36.139 |  |  |
| rs7575796 | A | G | 0.917 | 0.096 | 0.017 | 2.07E-08 | 0.0002 | 31.347 |  |  |
| rs7634476 | A | G | 0.397 | -0.058 | 0.009 | 5.46E-11 | 0.0003 | 42.997 |  |  |
| rs7647398 | C | T | 0.811 | 0.077 | 0.011 | 1.07E-12 | 0.0004 | 50.551 |  |  |
| rs76838079 | C | T | 0.849 | -0.078 | 0.014 | 1.53E-08 | 0.0002 | 31.951 |  |  |
| rs778371 | A | G | 0.699 | -0.081 | 0.010 | 1.50E-17 | 0.0006 | 71.987 |  |  |
| rs7798283 | T | G | 0.875 | 0.074 | 0.013 | 3.49E-08 | 0.0002 | 30.499 |  |  |
| rs7830315 | T | C | 0.478 | -0.048 | 0.009 | 3.08E-08 | 0.0002 | 30.899 |  |  |
| rs79210963 | T | C | 0.884 | -0.086 | 0.014 | 4.14E-10 | 0.0003 | 39.041 |  |  |
| rs79445414 | T | C | 0.956 | -0.123 | 0.022 | 2.80E-08 | 0.0002 | 30.898 |  |  |
| rs8055219 | G | A | 0.758 | -0.067 | 0.010 | 5.69E-11 | 0.0003 | 43.355 |  |  |
| rs9304548 | C | A | 0.261 | 0.057 | 0.010 | 1.59E-08 | 0.0002 | 32.151 |  |  |
| rs9318627 | A | C | 0.613 | 0.061 | 0.009 | 4.35E-12 | 0.0004 | 48.363 |  |  |
| rs9461916 | T | C | 0.388 | -0.053 | 0.009 | 1.64E-09 | 0.0003 | 36.679 |  |  |
| rs9636107 | A | G | 0.506 | -0.070 | 0.009 | 5.12E-16 | 0.0005 | 66.057 |  |  |
| rs9687282 | T | G | 0.650 | -0.053 | 0.009 | 7.33E-09 | 0.0003 | 33.410 |  |  |
| rs9876421 | C | T | 0.645 | -0.063 | 0.009 | 9.19E-12 | 0.0004 | 46.156 |  |  |

Abbreviations: EAF, effect allele frequency; GWAS, genome-wide association studies; MDD, major depressive disorder; MR, Mendelian randomisation; SE, standard error; SNP, single nucleotide polymorphism.

## Table S3. Summary information on genetic instruments for schizophrenia-MDD MR analysis in MDD GWAS dataset.

| **SNP** | **Effect Allele** | **Alternate Allele** | **EAF** | **Beta** | **SE** | ***p* value** |
| --- | --- | --- | --- | --- | --- | --- |
| rs1000237 | T | A | 0.640 | 0.013 | 0.009 | 1.75E-01 |
| rs10035564 | A | G | 0.684 | 0.016 | 0.010 | 9.12E-02 |
| rs10086619 | A | G | 0.831 | -0.014 | 0.012 | 2.41E-01 |
| rs10108980 | C | T | 0.797 | -0.004 | 0.011 | 6.91E-01 |
| rs10117 | G | A | 0.603 | 0.001 | 0.009 | 8.94E-01 |
| rs10861176 | G | A | 0.266 | 0.011 | 0.010 | 2.76E-01 |
| rs10876446 | G | C | 0.680 | -0.024 | 0.010 | 1.59E-02 |
| rs11027839 | A | C | 0.495 | -0.007 | 0.009 | 4.03E-01 |
| rs11136325 | G | A | 0.417 | 0.017 | 0.010 | 9.16E-02 |
| rs11165867 | C | T | 0.837 | 0.001 | 0.012 | 9.19E-01 |
| rs11191580 | T | C | 0.915 | 0.045 | 0.016 | 4.45E-03 |
| rs11210892 | G | A | 0.325 | 0.004 | 0.010 | 6.56E-01 |
| rs11223774 | A | G | 0.290 | -0.030 | 0.010 | 4.34E-03 |
| rs113264400 | T | C | 0.954 | -0.052 | 0.024 | 3.08E-02 |
| rs11534045 | G | A | 0.688 | 0.003 | 0.010 | 7.73E-01 |
| rs11587347 | C | G | 0.907 | -0.028 | 0.016 | 7.29E-02 |
| rs11664298 | G | A | 0.801 | -0.039 | 0.011 | 4.80E-04 |
| rs11693094 | C | T | 0.534 | 0.010 | 0.009 | 2.67E-01 |
| rs117178087 | C | T | 0.936 | -0.009 | 0.018 | 6.42E-01 |
| rs11941714 | G | A | 0.678 | 0.015 | 0.010 | 1.09E-01 |
| rs1198588 | A | T | 0.207 | -0.026 | 0.011 | 1.73E-02 |
| rs12129573 | C | A | 0.625 | -0.049 | 0.009 | 1.34E-07 |
| rs12138231 | T | A | 0.184 | -0.006 | 0.012 | 6.18E-01 |
| rs12151767 | G | A | 0.513 | 0.014 | 0.009 | 1.12E-01 |
| rs12285419 | C | A | 0.804 | -0.015 | 0.011 | 1.78E-01 |
| rs12293670 | A | G | 0.659 | 0.009 | 0.009 | 3.54E-01 |
| rs12303743 | G | C | 0.905 | 0.005 | 0.015 | 7.19E-01 |
| rs12489270 | T | C | 0.624 | 0.002 | 0.009 | 8.32E-01 |
| rs12652777 | T | C | 0.490 | 0.002 | 0.009 | 8.39E-01 |
| rs12712510 | T | C | 0.467 | 0.022 | 0.009 | 1.53E-02 |
| rs12771371 | G | A | 0.691 | -0.001 | 0.010 | 8.88E-01 |
| rs12833624 | C | T | 0.659 | -0.009 | 0.009 | 3.44E-01 |
| rs12877581 | G | C | 0.721 | 0.002 | 0.010 | 8.47E-01 |
| rs12883788 | C | T | 0.577 | -0.005 | 0.009 | 6.10E-01 |
| rs13016542 | T | C | 0.859 | -0.006 | 0.013 | 6.54E-01 |
| rs13107325 | C | T | 0.943 | 0.027 | 0.019 | 1.62E-01 |
| rs13195636 | A | C | 0.908 | 0.063 | 0.016 | 5.82E-05 |
| rs13233308 | C | T | 0.521 | 0.000 | 0.009 | 9.79E-01 |
| rs132582 | C | T | 0.456 | 0.010 | 0.009 | 2.49E-01 |
| rs1427633 | G | C | 0.415 | 0.007 | 0.009 | 4.66E-01 |
| rs1430894 | C | T | 0.514 | -0.006 | 0.009 | 5.12E-01 |
| rs145071536 | T | C | 0.809 | 0.001 | 0.013 | 9.08E-01 |
| rs1451488 | A | G | 0.436 | -0.006 | 0.009 | 5.32E-01 |
| rs149165 | T | G | 0.551 | -0.010 | 0.009 | 2.57E-01 |
| rs1593304 | A | G | 0.198 | -0.005 | 0.012 | 6.49E-01 |
| rs1604060 | A | G | 0.120 | -0.030 | 0.014 | 3.25E-02 |
| rs1615350 | C | T | 0.245 | -0.024 | 0.010 | 2.12E-02 |
| rs167924 | A | G | 0.365 | 0.000 | 0.009 | 9.96E-01 |
| rs16851048 | T | C | 0.796 | 0.000 | 0.011 | 9.97E-01 |
| rs16867571 | A | G | 0.752 | 0.006 | 0.011 | 5.97E-01 |
| rs17016552 | C | G | 0.665 | 0.017 | 0.009 | 7.99E-02 |
| rs17194490 | G | T | 0.836 | -0.012 | 0.013 | 3.55E-01 |
| rs17731 | G | A | 0.629 | 0.000 | 0.009 | 9.87E-01 |
| rs187557 | C | T | 0.161 | 0.024 | 0.012 | 4.91E-02 |
| rs1881046 | G | T | 0.661 | -0.005 | 0.010 | 6.17E-01 |
| rs1901512 | T | C | 0.297 | 0.003 | 0.010 | 7.84E-01 |
| rs1915019 | A | G | 0.241 | -0.017 | 0.010 | 1.06E-01 |
| rs2053079 | A | G | 0.761 | 0.013 | 0.011 | 2.06E-01 |
| rs2078266 | A | G | 0.173 | -0.008 | 0.016 | 6.27E-01 |
| rs215412 | G | A | 0.664 | -0.001 | 0.009 | 8.72E-01 |
| rs217336 | C | A | 0.560 | -0.009 | 0.009 | 3.02E-01 |
| rs2238057 | T | G | 0.583 | -0.009 | 0.009 | 3.33E-01 |
| rs2252074 | T | G | 0.595 | -0.006 | 0.009 | 5.07E-01 |
| rs2332700 | C | G | 0.258 | 0.041 | 0.010 | 8.24E-05 |
| rs2333321 | A | G | 0.220 | 0.021 | 0.011 | 5.50E-02 |
| rs2381411 | T | C | 0.606 | -0.001 | 0.009 | 9.49E-01 |
| rs2455415 | C | T | 0.574 | -0.001 | 0.009 | 9.46E-01 |
| rs2456020 | C | T | 0.769 | 0.002 | 0.011 | 8.59E-01 |
| rs2514218 | C | T | 0.657 | 0.027 | 0.009 | 4.16E-03 |
| rs2710323 | T | C | 0.516 | 0.024 | 0.009 | 6.53E-03 |
| rs2815731 | C | A | 0.644 | 0.024 | 0.009 | 1.08E-02 |
| rs2909457 | G | A | 0.445 | -0.004 | 0.009 | 6.69E-01 |
| rs2999392 | C | T | 0.309 | -0.007 | 0.010 | 4.90E-01 |
| rs308697 | C | A | 0.563 | -0.014 | 0.009 | 1.22E-01 |
| rs35351411 | A | C | 0.453 | -0.005 | 0.009 | 6.02E-01 |
| rs35734242 | T | C | 0.577 | -0.003 | 0.010 | 7.50E-01 |
| rs3739118 | G | A | 0.718 | 0.027 | 0.010 | 5.81E-03 |
| rs3770754 | C | G | 0.636 | 0.006 | 0.009 | 5.50E-01 |
| rs3791710 | T | C | 0.797 | -0.016 | 0.011 | 1.51E-01 |
| rs3795310 | C | T | 0.537 | -0.030 | 0.009 | 9.85E-04 |
| rs3802924 | A | C | 0.779 | 0.012 | 0.011 | 2.87E-01 |
| rs3814883 | C | T | 0.532 | 0.017 | 0.009 | 5.92E-02 |
| rs3824451 | T | C | 0.840 | -0.009 | 0.012 | 4.53E-01 |
| rs4129585 | A | C | 0.440 | 0.011 | 0.009 | 2.38E-01 |
| rs4575535 | A | G | 0.299 | -0.024 | 0.010 | 1.56E-02 |
| rs4632195 | C | T | 0.456 | -0.034 | 0.009 | 1.24E-04 |
| rs4636654 | G | A | 0.606 | 0.019 | 0.009 | 3.79E-02 |
| rs4653164 | C | T | 0.313 | -0.038 | 0.010 | 8.50E-05 |
| rs4702 | G | A | 0.442 | 0.026 | 0.010 | 5.68E-03 |
| rs4766428 | C | T | 0.555 | -0.020 | 0.009 | 2.83E-02 |
| rs4779050 | T | G | 0.366 | 0.008 | 0.009 | 3.96E-01 |
| rs4812325 | G | A | 0.380 | -0.013 | 0.009 | 1.70E-01 |
| rs4921741 | A | G | 0.747 | -0.004 | 0.010 | 7.05E-01 |
| rs498591 | A | T | 0.850 | -0.027 | 0.013 | 3.21E-02 |
| rs500102 | T | C | 0.426 | 0.013 | 0.009 | 1.57E-01 |
| rs505061 | C | A | 0.511 | -0.021 | 0.009 | 2.03E-02 |
| rs56205728 | G | A | 0.714 | -0.009 | 0.011 | 3.85E-01 |
| rs56335113 | A | G | 0.312 | 0.021 | 0.010 | 3.23E-02 |
| rs57433322 | C | G | 0.882 | -0.006 | 0.014 | 6.48E-01 |
| rs5751191 | T | C | 0.492 | -0.007 | 0.009 | 4.38E-01 |
| rs58120505 | T | C | 0.573 | -0.012 | 0.009 | 2.11E-01 |
| rs60135207 | G | T | 0.587 | 0.007 | 0.009 | 4.55E-01 |
| rs6125656 | G | A | 0.817 | -0.021 | 0.012 | 6.39E-02 |
| rs61857878 | A | T | 0.757 | 0.006 | 0.011 | 5.53E-01 |
| rs61937595 | C | T | 0.912 | -0.005 | 0.016 | 7.73E-01 |
| rs62018952 | T | C | 0.274 | -0.031 | 0.010 | 1.74E-03 |
| rs62183855 | A | C | 0.808 | 0.001 | 0.012 | 9.61E-01 |
| rs634940 | G | T | 0.739 | -0.017 | 0.010 | 9.61E-02 |
| rs6482437 | A | C | 0.105 | -0.029 | 0.015 | 4.68E-02 |
| rs6520064 | A | G | 0.796 | -0.005 | 0.011 | 6.47E-01 |
| rs6538539 | G | T | 0.450 | 0.005 | 0.009 | 5.97E-01 |
| rs6546857 | A | G | 0.763 | -0.012 | 0.011 | 2.69E-01 |
| rs6549963 | T | C | 0.577 | 0.005 | 0.009 | 5.93E-01 |
| rs6673880 | A | G | 0.475 | -0.008 | 0.011 | 4.84E-01 |
| rs6715366 | G | A | 0.721 | -0.034 | 0.010 | 6.15E-04 |
| rs6798742 | A | G | 0.700 | -0.010 | 0.010 | 2.92E-01 |
| rs6943762 | T | C | 0.871 | -0.029 | 0.013 | 3.14E-02 |
| rs6974218 | A | C | 0.627 | 0.017 | 0.009 | 5.89E-02 |
| rs6984242 | G | A | 0.392 | -0.018 | 0.009 | 4.70E-02 |
| rs708228 | C | T | 0.667 | -0.032 | 0.009 | 7.27E-04 |
| rs7112616 | T | C | 0.498 | -0.005 | 0.009 | 6.06E-01 |
| rs713692 | G | A | 0.300 | -0.006 | 0.010 | 5.63E-01 |
| rs7251 | C | G | 0.672 | 0.002 | 0.010 | 8.24E-01 |
| rs72802868 | G | T | 0.697 | -0.003 | 0.010 | 7.80E-01 |
| rs728055 | T | A | 0.656 | -0.009 | 0.010 | 3.23E-01 |
| rs72943392 | G | C | 0.691 | -0.018 | 0.010 | 6.42E-02 |
| rs72986630 | C | T | 0.938 | 0.028 | 0.020 | 1.63E-01 |
| rs73229090 | C | A | 0.885 | 0.015 | 0.014 | 2.99E-01 |
| rs73292401 | T | A | 0.806 | -0.019 | 0.011 | 9.86E-02 |
| rs7515363 | C | T | 0.395 | 0.009 | 0.009 | 3.24E-01 |
| rs7575796 | A | G | 0.908 | -0.009 | 0.017 | 6.13E-01 |
| rs7634476 | A | G | 0.443 | -0.012 | 0.009 | 2.09E-01 |
| rs7647398 | C | T | 0.802 | 0.006 | 0.011 | 5.96E-01 |
| rs76838079 | C | T | 0.849 | 0.023 | 0.014 | 1.11E-01 |
| rs778371 | A | G | 0.713 | 0.020 | 0.010 | 4.42E-02 |
| rs7798283 | T | G | 0.865 | 0.019 | 0.013 | 1.64E-01 |
| rs7830315 | T | C | 0.492 | -0.024 | 0.009 | 6.34E-03 |
| rs79210963 | T | C | 0.888 | -0.025 | 0.014 | 7.76E-02 |
| rs79445414 | T | C | 0.950 | -0.051 | 0.021 | 1.64E-02 |
| rs8055219 | G | A | 0.772 | -0.034 | 0.011 | 1.56E-03 |
| rs9304548 | C | A | 0.251 | 0.007 | 0.010 | 4.72E-01 |
| rs9318627 | A | C | 0.593 | 0.009 | 0.009 | 3.18E-01 |
| rs9461916 | T | C | 0.411 | -0.024 | 0.009 | 7.90E-03 |
| rs9636107 | A | G | 0.532 | -0.019 | 0.009 | 3.64E-02 |
| rs9687282 | T | G | 0.669 | 0.014 | 0.010 | 1.47E-01 |
| rs9876421 | C | T | 0.648 | 0.003 | 0.009 | 7.05E-01 |

Abbreviations: EAF, effect allele frequency; GWAS, genome-wide association studies; MDD, major depressive disorder; MR, Mendelian randomisation; SE, standard error; SNP, single nucleotide polymorphism.

## Table S4. Summary information on genetic instruments for schizophrenia-educational years MR analysis in schizophrenia GWAS dataset.

| **SNP** | **Effect Allele** | **Alternate Allele** | **EAF** | **Beta** | **SE** | ***p* value** | ***R*^2^** | ***F* statistics** | **Overall *R*^2^** | **Mean *F* statistics** |
| --- | --- | --- | --- | --- | --- | --- | --- | --- | --- | --- |
| rs1000237 | T | A | 0.627 | -0.073 | 0.009 | 2.80E-16 | 0.0005 | 67.656 | 0.050 | 44.622 |
| rs10035564 | A | G | 0.650 | -0.067 | 0.009 | 4.38E-13 | 0.0004 | 52.724 |  |  |
| rs10086619 | A | G | 0.831 | -0.072 | 0.012 | 4.97E-10 | 0.0003 | 38.745 |  |  |
| rs10108980 | C | T | 0.785 | -0.063 | 0.011 | 2.73E-09 | 0.0003 | 35.102 |  |  |
| rs10117 | G | A | 0.613 | 0.055 | 0.009 | 4.66E-10 | 0.0003 | 39.062 |  |  |
| rs10861176 | G | A | 0.257 | -0.056 | 0.010 | 1.59E-08 | 0.0002 | 32.075 |  |  |
| rs10873538 | T | G | 0.654 | -0.067 | 0.009 | 3.01E-13 | 0.0004 | 53.407 |  |  |
| rs10876446 | G | C | 0.674 | -0.054 | 0.009 | 1.03E-08 | 0.0003 | 33.004 |  |  |
| rs11027839 | A | C | 0.488 | -0.052 | 0.009 | 2.40E-09 | 0.0003 | 35.866 |  |  |
| rs11136325 | G | A | 0.436 | 0.054 | 0.009 | 3.05E-09 | 0.0003 | 34.948 |  |  |
| rs11165867 | C | T | 0.829 | -0.074 | 0.012 | 1.30E-10 | 0.0003 | 41.030 |  |  |
| rs11191580 | T | C | 0.920 | 0.132 | 0.016 | 1.77E-17 | 0.0006 | 72.199 |  |  |
| rs11210892 | G | A | 0.347 | 0.064 | 0.009 | 2.68E-12 | 0.0004 | 48.694 |  |  |
| rs11223774 | A | G | 0.301 | 0.052 | 0.009 | 2.74E-08 | 0.0002 | 31.191 |  |  |
| rs113264400 | T | C | 0.948 | -0.112 | 0.020 | 2.87E-08 | 0.0002 | 30.905 |  |  |
| rs11534045 | G | A | 0.686 | 0.063 | 0.009 | 1.40E-11 | 0.0003 | 45.594 |  |  |
| rs11587347 | C | G | 0.895 | -0.104 | 0.015 | 1.53E-12 | 0.0004 | 49.952 |  |  |
| rs11664298 | G | A | 0.794 | -0.077 | 0.011 | 8.94E-13 | 0.0004 | 51.360 |  |  |
| rs11693094 | C | T | 0.556 | 0.054 | 0.009 | 4.29E-10 | 0.0003 | 39.103 |  |  |
| rs117178087 | C | T | 0.939 | 0.096 | 0.018 | 4.89E-08 | 0.0002 | 29.663 |  |  |
| rs11941714 | G | A | 0.673 | 0.052 | 0.009 | 3.07E-08 | 0.0002 | 30.780 |  |  |
| rs1198588 | A | T | 0.194 | -0.103 | 0.011 | 1.73E-21 | 0.0007 | 90.246 |  |  |
| rs12129573 | C | A | 0.616 | -0.078 | 0.009 | 2.28E-18 | 0.0006 | 76.414 |  |  |
| rs12138231 | T | A | 0.171 | -0.067 | 0.012 | 7.99E-09 | 0.0003 | 33.355 |  |  |
| rs12151767 | G | A | 0.524 | 0.061 | 0.009 | 1.31E-12 | 0.0004 | 50.484 |  |  |
| rs12285419 | C | A | 0.800 | -0.085 | 0.011 | 1.05E-14 | 0.0005 | 59.577 |  |  |
| rs12293670 | A | G | 0.678 | 0.070 | 0.009 | 1.56E-14 | 0.0004 | 58.715 |  |  |
| rs12303743 | G | C | 0.898 | -0.087 | 0.015 | 1.59E-09 | 0.0003 | 36.414 |  |  |
| rs12489270 | T | C | 0.612 | -0.058 | 0.009 | 7.47E-11 | 0.0003 | 42.330 |  |  |
| rs12652777 | T | C | 0.489 | 0.049 | 0.009 | 1.52E-08 | 0.0002 | 32.199 |  |  |
| rs12712510 | T | C | 0.488 | 0.057 | 0.009 | 5.14E-11 | 0.0003 | 43.531 |  |  |
| rs12771371 | G | A | 0.697 | 0.052 | 0.009 | 1.94E-08 | 0.0002 | 31.750 |  |  |
| rs12833624 | C | T | 0.646 | -0.050 | 0.009 | 2.77E-08 | 0.0002 | 31.111 |  |  |
| rs12877581 | G | C | 0.716 | -0.060 | 0.010 | 1.80E-09 | 0.0003 | 36.245 |  |  |
| rs12883788 | C | T | 0.527 | -0.061 | 0.009 | 1.86E-12 | 0.0004 | 49.648 |  |  |
| rs13016542 | T | C | 0.876 | 0.088 | 0.013 | 8.28E-12 | 0.0004 | 46.858 |  |  |
| rs13107325 | C | T | 0.919 | -0.159 | 0.017 | 2.90E-21 | 0.0007 | 89.238 |  |  |
| rs13195636 | A | C | 0.926 | 0.211 | 0.016 | 6.55E-40 | 0.0013 | 175.278 |  |  |
| rs13233308 | C | T | 0.526 | 0.049 | 0.009 | 1.75E-08 | 0.0002 | 32.073 |  |  |
| rs132582 | C | T | 0.471 | 0.051 | 0.009 | 3.26E-09 | 0.0003 | 35.164 |  |  |
| rs1427633 | G | C | 0.419 | 0.048 | 0.009 | 4.10E-08 | 0.0002 | 30.131 |  |  |
| rs1430894 | C | T | 0.506 | -0.053 | 0.009 | 6.15E-10 | 0.0003 | 38.404 |  |  |
| rs145071536 | T | C | 0.799 | -0.085 | 0.012 | 1.62E-12 | 0.0004 | 50.292 |  |  |
| rs1451488 | A | G | 0.432 | -0.071 | 0.009 | 4.47E-16 | 0.0005 | 66.403 |  |  |
| rs149165 | T | G | 0.569 | 0.048 | 0.009 | 3.01E-08 | 0.0002 | 30.694 |  |  |
| rs1593304 | A | G | 0.193 | -0.064 | 0.011 | 7.45E-09 | 0.0003 | 33.349 |  |  |
| rs1604060 | A | G | 0.103 | -0.077 | 0.014 | 3.24E-08 | 0.0002 | 30.411 |  |  |
| rs1615350 | C | T | 0.273 | 0.074 | 0.010 | 4.92E-14 | 0.0004 | 56.409 |  |  |
| rs167924 | A | G | 0.359 | -0.050 | 0.009 | 2.34E-08 | 0.0002 | 31.111 |  |  |
| rs16851048 | T | C | 0.792 | -0.074 | 0.011 | 4.15E-12 | 0.0004 | 48.474 |  |  |
| rs16867571 | A | G | 0.778 | 0.066 | 0.010 | 2.68E-10 | 0.0003 | 39.913 |  |  |
| rs17016552 | C | G | 0.661 | 0.052 | 0.009 | 1.20E-08 | 0.0002 | 32.278 |  |  |
| rs17194490 | G | T | 0.827 | -0.078 | 0.012 | 1.80E-11 | 0.0003 | 45.446 |  |  |
| rs17731 | G | A | 0.620 | -0.052 | 0.009 | 4.37E-09 | 0.0003 | 34.663 |  |  |
| rs187557 | C | T | 0.163 | 0.067 | 0.012 | 2.03E-08 | 0.0002 | 31.412 |  |  |
| rs1881046 | G | T | 0.670 | 0.051 | 0.009 | 3.39E-08 | 0.0002 | 30.373 |  |  |
| rs1901512 | T | C | 0.318 | 0.058 | 0.009 | 5.72E-10 | 0.0003 | 38.600 |  |  |
| rs1915019 | A | G | 0.265 | 0.057 | 0.010 | 6.57E-09 | 0.0003 | 33.947 |  |  |
| rs2053079 | A | G | 0.754 | -0.060 | 0.010 | 3.01E-09 | 0.0003 | 35.172 |  |  |
| rs2078266 | A | G | 0.178 | 0.070 | 0.013 | 2.94E-08 | 0.0002 | 30.513 |  |  |
| rs215412 | G | A | 0.661 | -0.058 | 0.009 | 2.69E-10 | 0.0003 | 40.209 |  |  |
| rs217336 | C | A | 0.583 | 0.050 | 0.009 | 8.05E-09 | 0.0003 | 33.431 |  |  |
| rs2238057 | T | G | 0.568 | -0.084 | 0.009 | 8.50E-22 | 0.0007 | 92.118 |  |  |
| rs2252074 | T | G | 0.590 | -0.069 | 0.009 | 6.19E-15 | 0.0005 | 60.599 |  |  |
| rs2332700 | C | G | 0.258 | 0.075 | 0.010 | 3.88E-14 | 0.0004 | 57.542 |  |  |
| rs2333321 | A | G | 0.218 | 0.071 | 0.011 | 1.25E-11 | 0.0004 | 45.986 |  |  |
| rs2381411 | T | C | 0.586 | -0.050 | 0.009 | 1.25E-08 | 0.0003 | 32.800 |  |  |
| rs2455415 | C | T | 0.577 | -0.049 | 0.009 | 1.69E-08 | 0.0002 | 31.634 |  |  |
| rs2456020 | C | T | 0.773 | 0.082 | 0.010 | 1.13E-15 | 0.0005 | 63.998 |  |  |
| rs2514218 | C | T | 0.668 | 0.070 | 0.009 | 1.35E-14 | 0.0004 | 58.715 |  |  |
| rs2710323 | T | C | 0.531 | 0.078 | 0.009 | 1.23E-19 | 0.0006 | 83.116 |  |  |
| rs2815731 | C | A | 0.660 | 0.060 | 0.009 | 4.39E-11 | 0.0003 | 43.478 |  |  |
| rs2909457 | G | A | 0.451 | 0.049 | 0.009 | 1.48E-08 | 0.0002 | 31.721 |  |  |
| rs2999392 | C | T | 0.299 | -0.052 | 0.009 | 3.05E-08 | 0.0002 | 30.366 |  |  |
| rs308697 | C | A | 0.574 | 0.050 | 0.009 | 8.83E-09 | 0.0003 | 33.166 |  |  |
| rs35351411 | A | C | 0.439 | -0.064 | 0.009 | 2.21E-13 | 0.0004 | 53.281 |  |  |
| rs35734242 | T | C | 0.562 | -0.051 | 0.009 | 1.37E-08 | 0.0002 | 32.457 |  |  |
| rs3739118 | G | A | 0.719 | 0.057 | 0.010 | 2.36E-09 | 0.0003 | 36.005 |  |  |
| rs3770754 | C | G | 0.645 | 0.053 | 0.009 | 5.35E-09 | 0.0003 | 33.788 |  |  |
| rs3791710 | T | C | 0.804 | 0.060 | 0.011 | 3.02E-08 | 0.0002 | 30.868 |  |  |
| rs3795310 | C | T | 0.543 | 0.051 | 0.009 | 5.75E-09 | 0.0003 | 34.360 |  |  |
| rs3802924 | A | C | 0.805 | 0.074 | 0.011 | 9.58E-12 | 0.0004 | 46.446 |  |  |
| rs3814883 | C | T | 0.548 | 0.067 | 0.009 | 1.58E-14 | 0.0005 | 59.481 |  |  |
| rs3824451 | T | C | 0.838 | -0.066 | 0.012 | 2.54E-08 | 0.0002 | 30.901 |  |  |
| rs4129585 | A | C | 0.456 | 0.075 | 0.009 | 5.11E-18 | 0.0006 | 74.309 |  |  |
| rs4575535 | A | G | 0.281 | -0.056 | 0.010 | 5.77E-09 | 0.0003 | 33.783 |  |  |
| rs4632195 | C | T | 0.471 | -0.047 | 0.009 | 4.59E-08 | 0.0002 | 30.118 |  |  |
| rs4636654 | G | A | 0.608 | 0.048 | 0.009 | 4.89E-08 | 0.0002 | 29.457 |  |  |
| rs4653164 | C | T | 0.323 | -0.051 | 0.009 | 3.08E-08 | 0.0002 | 30.855 |  |  |
| rs4702 | G | A | 0.461 | 0.084 | 0.009 | 2.79E-21 | 0.0007 | 89.726 |  |  |
| rs4766428 | C | T | 0.539 | -0.075 | 0.009 | 3.93E-17 | 0.0005 | 71.021 |  |  |
| rs4779050 | T | G | 0.381 | 0.058 | 0.009 | 7.27E-11 | 0.0003 | 42.462 |  |  |
| rs4812325 | G | A | 0.370 | -0.072 | 0.009 | 8.96E-16 | 0.0005 | 65.272 |  |  |
| rs4921741 | A | G | 0.729 | -0.056 | 0.010 | 1.21E-08 | 0.0002 | 32.652 |  |  |
| rs498591 | A | T | 0.847 | -0.072 | 0.012 | 2.11E-09 | 0.0003 | 35.896 |  |  |
| rs500102 | T | C | 0.413 | 0.052 | 0.009 | 4.87E-09 | 0.0003 | 34.516 |  |  |
| rs505061 | C | A | 0.495 | -0.053 | 0.009 | 5.80E-10 | 0.0003 | 38.694 |  |  |
| rs56205728 | G | A | 0.700 | -0.063 | 0.010 | 1.01E-10 | 0.0003 | 42.188 |  |  |
| rs56335113 | A | G | 0.316 | 0.065 | 0.009 | 6.02E-12 | 0.0004 | 47.377 |  |  |
| rs57433322 | C | G | 0.885 | 0.083 | 0.014 | 1.99E-09 | 0.0003 | 35.741 |  |  |
| rs5751191 | T | C | 0.484 | -0.066 | 0.009 | 3.00E-14 | 0.0004 | 58.176 |  |  |
| rs58120505 | T | C | 0.602 | 0.090 | 0.009 | 2.24E-24 | 0.0008 | 103.676 |  |  |
| rs60135207 | G | T | 0.596 | 0.050 | 0.009 | 1.53E-08 | 0.0002 | 31.768 |  |  |
| rs6125656 | G | A | 0.809 | -0.064 | 0.011 | 6.29E-09 | 0.0003 | 33.761 |  |  |
| rs61857878 | A | T | 0.760 | 0.060 | 0.010 | 4.44E-09 | 0.0003 | 34.715 |  |  |
| rs61937595 | C | T | 0.917 | 0.130 | 0.016 | 1.15E-15 | 0.0005 | 64.493 |  |  |
| rs62018952 | T | C | 0.264 | -0.058 | 0.010 | 1.94E-09 | 0.0003 | 36.251 |  |  |
| rs62183855 | A | C | 0.814 | 0.066 | 0.011 | 2.66E-09 | 0.0003 | 35.458 |  |  |
| rs634940 | G | T | 0.737 | -0.066 | 0.010 | 1.78E-11 | 0.0003 | 44.980 |  |  |
| rs6482437 | A | C | 0.099 | -0.099 | 0.014 | 3.33E-12 | 0.0004 | 48.512 |  |  |
| rs6520064 | A | G | 0.787 | -0.058 | 0.011 | 3.58E-08 | 0.0002 | 30.456 |  |  |
| rs6538539 | G | T | 0.462 | 0.057 | 0.009 | 4.43E-11 | 0.0003 | 43.615 |  |  |
| rs6546857 | A | G | 0.757 | -0.060 | 0.010 | 2.74E-09 | 0.0003 | 35.062 |  |  |
| rs6549963 | T | C | 0.596 | 0.048 | 0.009 | 4.31E-08 | 0.0002 | 30.131 |  |  |
| rs6673880 | A | G | 0.492 | -0.062 | 0.009 | 7.20E-12 | 0.0004 | 46.871 |  |  |
| rs6715366 | G | A | 0.723 | -0.054 | 0.010 | 2.49E-08 | 0.0002 | 31.103 |  |  |
| rs6798742 | A | G | 0.680 | -0.061 | 0.009 | 4.57E-11 | 0.0003 | 43.162 |  |  |
| rs6943762 | T | C | 0.883 | 0.105 | 0.013 | 1.57E-15 | 0.0005 | 63.394 |  |  |
| rs6974218 | A | C | 0.634 | 0.055 | 0.009 | 6.80E-10 | 0.0003 | 38.044 |  |  |
| rs6984242 | G | A | 0.413 | 0.055 | 0.009 | 3.86E-10 | 0.0003 | 39.526 |  |  |
| rs708228 | C | T | 0.661 | -0.053 | 0.009 | 6.56E-09 | 0.0003 | 33.665 |  |  |
| rs7112616 | T | C | 0.515 | 0.052 | 0.009 | 1.52E-09 | 0.0003 | 36.847 |  |  |
| rs713692 | G | A | 0.299 | -0.057 | 0.010 | 2.67E-09 | 0.0003 | 35.499 |  |  |
| rs7251 | C | G | 0.682 | 0.064 | 0.009 | 8.29E-12 | 0.0004 | 46.502 |  |  |
| rs72802868 | G | T | 0.724 | 0.069 | 0.010 | 4.55E-13 | 0.0004 | 51.959 |  |  |
| rs728055 | T | A | 0.658 | 0.067 | 0.009 | 8.85E-14 | 0.0004 | 56.078 |  |  |
| rs72943392 | G | C | 0.707 | -0.053 | 0.010 | 2.39E-08 | 0.0002 | 31.052 |  |  |
| rs72986630 | C | T | 0.926 | -0.112 | 0.018 | 3.59E-10 | 0.0003 | 39.357 |  |  |
| rs73229090 | C | A | 0.897 | 0.103 | 0.014 | 4.34E-13 | 0.0004 | 52.207 |  |  |
| rs73292401 | T | A | 0.799 | -0.068 | 0.011 | 5.48E-10 | 0.0003 | 38.468 |  |  |
| rs7515363 | C | T | 0.389 | 0.054 | 0.009 | 1.84E-09 | 0.0003 | 36.139 |  |  |
| rs7575796 | A | G | 0.917 | 0.096 | 0.017 | 2.07E-08 | 0.0002 | 31.347 |  |  |
| rs7634476 | A | G | 0.397 | -0.058 | 0.009 | 5.46E-11 | 0.0003 | 42.997 |  |  |
| rs7647398 | C | T | 0.811 | 0.077 | 0.011 | 1.07E-12 | 0.0004 | 50.551 |  |  |
| rs76838079 | C | T | 0.849 | -0.078 | 0.014 | 1.53E-08 | 0.0002 | 31.951 |  |  |
| rs778371 | A | G | 0.699 | -0.081 | 0.010 | 1.50E-17 | 0.0006 | 71.987 |  |  |
| rs7798283 | T | G | 0.875 | 0.074 | 0.013 | 3.49E-08 | 0.0002 | 30.499 |  |  |
| rs7830315 | T | C | 0.478 | -0.048 | 0.009 | 3.08E-08 | 0.0002 | 30.899 |  |  |
| rs79210963 | T | C | 0.884 | -0.086 | 0.014 | 4.14E-10 | 0.0003 | 39.041 |  |  |
| rs79445414 | T | C | 0.956 | -0.123 | 0.022 | 2.80E-08 | 0.0002 | 30.898 |  |  |
| rs8055219 | G | A | 0.758 | -0.067 | 0.010 | 5.69E-11 | 0.0003 | 43.355 |  |  |
| rs9304548 | C | A | 0.261 | 0.057 | 0.010 | 1.59E-08 | 0.0002 | 32.151 |  |  |
| rs9318627 | A | C | 0.613 | 0.061 | 0.009 | 4.35E-12 | 0.0004 | 48.363 |  |  |
| rs9461916 | T | C | 0.388 | -0.053 | 0.009 | 1.64E-09 | 0.0003 | 36.679 |  |  |
| rs9636107 | A | G | 0.506 | -0.070 | 0.009 | 5.12E-16 | 0.0005 | 66.057 |  |  |
| rs9687282 | T | G | 0.650 | -0.053 | 0.009 | 7.33E-09 | 0.0003 | 33.410 |  |  |
| rs9876421 | C | T | 0.645 | -0.063 | 0.009 | 9.19E-12 | 0.0004 | 46.156 |  |  |

Abbreviations: EAF, effect allele frequency; GWAS, genome-wide association studies; MR, Mendelian randomisation; SE, standard error; SNP, single nucleotide polymorphism.

## Table S5. Summary information on genetic instruments for schizophrenia-educational years MR analysis in educational years GWAS dataset.

| **SNP** | **Effect Allele** | **Alternate Allele** | **EAF** | **Beta** | **SE** | ***p* value** |
| --- | --- | --- | --- | --- | --- | --- |
| rs1000237 | T | A | 0.633 | 0.0095 | 0.002 | 9.12E-08 |
| rs10035564 | A | G | 0.674 | -0.0051 | 0.002 | 5.04E-03 |
| rs10086619 | A | G | 0.855 | 0.0016 | 0.002 | 4.84E-01 |
| rs10108980 | C | T | 0.796 | 0.0009 | 0.002 | 6.70E-01 |
| rs10117 | G | A | 0.546 | -0.0011 | 0.002 | 5.16E-01 |
| rs10861176 | G | A | 0.270 | -0.0050 | 0.002 | 9.17E-03 |
| rs10873538 | T | G | 0.708 | -0.0050 | 0.002 | 5.64E-03 |
| rs10876446 | G | C | 0.708 | -0.0046 | 0.002 | 1.29E-02 |
| rs11027839 | A | C | 0.473 | 0.0017 | 0.002 | 3.15E-01 |
| rs11136325 | G | A | 0.388 | 0.0018 | 0.002 | 2.97E-01 |
| rs11165867 | C | T | 0.837 | -0.0044 | 0.002 | 5.38E-02 |
| rs11191580 | T | C | 0.910 | 0.0022 | 0.003 | 4.82E-01 |
| rs11210892 | G | A | 0.366 | -0.0162 | 0.002 | 6.40E-19 |
| rs11223774 | A | G | 0.257 | 0.0005 | 0.002 | 7.93E-01 |
| rs113264400 | T | C | 0.957 | 0.0040 | 0.004 | 3.12E-01 |
| rs11534045 | G | A | 0.663 | -0.0016 | 0.002 | 3.97E-01 |
| rs11587347 | C | G | 0.896 | -0.0041 | 0.003 | 1.60E-01 |
| rs11664298 | G | A | 0.827 | 0.0124 | 0.002 | 3.90E-09 |
| rs11693094 | C | T | 0.592 | 0.0030 | 0.002 | 7.84E-02 |
| rs117178087 | C | T | 0.940 | -0.0009 | 0.003 | 7.96E-01 |
| rs11941714 | G | A | 0.629 | -0.0010 | 0.002 | 6.01E-01 |
| rs1198588 | A | T | 0.211 | -0.0111 | 0.002 | 9.71E-08 |
| rs12129573 | C | A | 0.645 | 0.0028 | 0.002 | 1.20E-01 |
| rs12138231 | T | A | 0.185 | 0.0055 | 0.002 | 1.31E-02 |
| rs12151767 | G | A | 0.492 | 0.0022 | 0.002 | 2.01E-01 |
| rs12285419 | C | A | 0.794 | -0.0025 | 0.002 | 2.48E-01 |
| rs12293670 | A | G | 0.650 | 0.0046 | 0.002 | 1.15E-02 |
| rs12303743 | G | C | 0.917 | 0.0010 | 0.003 | 7.26E-01 |
| rs12489270 | T | C | 0.622 | 0.0115 | 0.002 | 4.87E-11 |
| rs12652777 | T | C | 0.466 | 0.0004 | 0.002 | 8.12E-01 |
| rs12712510 | T | C | 0.473 | -0.0011 | 0.002 | 5.09E-01 |
| rs12771371 | G | A | 0.684 | -0.0011 | 0.002 | 5.59E-01 |
| rs12833624 | C | T | 0.650 | -0.0021 | 0.002 | 2.55E-01 |
| rs12877581 | G | C | 0.689 | 0.0033 | 0.002 | 9.23E-02 |
| rs12883788 | C | T | 0.529 | 0.0052 | 0.002 | 2.34E-03 |
| rs13016542 | T | C | 0.866 | 0.0071 | 0.003 | 4.85E-03 |
| rs13107325 | C | T | 0.910 | 0.0188 | 0.003 | 4.45E-08 |
| rs13195636 | A | C | 0.912 | -0.0026 | 0.003 | 3.45E-01 |
| rs13233308 | C | T | 0.526 | -0.0033 | 0.002 | 5.22E-02 |
| rs132582 | C | T | 0.451 | -0.0097 | 0.002 | 1.45E-08 |
| rs1427633 | G | C | 0.412 | -0.0012 | 0.002 | 4.79E-01 |
| rs1430894 | C | T | 0.554 | -0.0008 | 0.002 | 6.21E-01 |
| rs145071536 | T | C | 0.784 | -0.0057 | 0.002 | 1.29E-02 |
| rs1451488 | A | G | 0.469 | -0.0020 | 0.002 | 2.35E-01 |
| rs149165 | T | G | 0.565 | 0.0027 | 0.002 | 1.16E-01 |
| rs1593304 | A | G | 0.209 | -0.0014 | 0.002 | 5.08E-01 |
| rs1604060 | A | G | 0.107 | 0.0061 | 0.003 | 2.03E-02 |
| rs1615350 | C | T | 0.282 | 0.0213 | 0.002 | 3.62E-27 |
| rs167924 | A | G | 0.361 | -0.0015 | 0.002 | 3.80E-01 |
| rs16851048 | T | C | 0.777 | -0.0028 | 0.002 | 2.00E-01 |
| rs16867571 | A | G | 0.786 | 0.0098 | 0.002 | 7.25E-07 |
| rs17016552 | C | G | 0.617 | 0.0002 | 0.002 | 9.05E-01 |
| rs17194490 | G | T | 0.840 | -0.0093 | 0.002 | 5.57E-05 |
| rs17731 | G | A | 0.629 | -0.0003 | 0.002 | 8.81E-01 |
| rs187557 | C | T | 0.191 | 0.0086 | 0.002 | 3.68E-04 |
| rs1881046 | G | T | 0.646 | -0.0073 | 0.002 | 5.44E-05 |
| rs1901512 | T | C | 0.330 | 0.0019 | 0.002 | 3.04E-01 |
| rs1915019 | A | G | 0.248 | 0.0035 | 0.002 | 7.90E-02 |
| rs2053079 | A | G | 0.733 | 0.0035 | 0.002 | 7.49E-02 |
| rs2078266 | A | G | 0.158 | -0.0043 | 0.002 | 7.41E-02 |
| rs215412 | G | A | 0.691 | -0.0031 | 0.002 | 9.05E-02 |
| rs217336 | C | A | 0.553 | -0.0001 | 0.002 | 9.67E-01 |
| rs2238057 | T | G | 0.573 | 0.0014 | 0.002 | 4.28E-01 |
| rs2252074 | T | G | 0.597 | 0.0050 | 0.002 | 4.06E-03 |
| rs2332700 | C | G | 0.242 | 0.0055 | 0.002 | 5.15E-03 |
| rs2333321 | A | G | 0.213 | -0.0081 | 0.002 | 1.11E-04 |
| rs2381411 | T | C | 0.634 | 0.0024 | 0.002 | 1.71E-01 |
| rs2455415 | C | T | 0.585 | 0.0005 | 0.002 | 7.59E-01 |
| rs2456020 | C | T | 0.776 | -0.0042 | 0.002 | 3.67E-02 |
| rs2514218 | C | T | 0.617 | -0.0060 | 0.002 | 7.18E-04 |
| rs2710323 | T | C | 0.539 | 0.0004 | 0.002 | 8.14E-01 |
| rs2815731 | C | A | 0.677 | 0.0023 | 0.002 | 1.99E-01 |
| rs2909457 | G | A | 0.454 | -0.0113 | 0.002 | 4.12E-11 |
| rs2999392 | C | T | 0.310 | 0.0027 | 0.002 | 1.49E-01 |
| rs308697 | C | A | 0.532 | 0.0055 | 0.002 | 1.35E-03 |
| rs35351411 | A | C | 0.437 | 0.0003 | 0.002 | 8.66E-01 |
| rs35734242 | T | C | 0.592 | -0.0037 | 0.002 | 3.44E-02 |
| rs3739118 | G | A | 0.730 | -0.0024 | 0.002 | 2.01E-01 |
| rs3770754 | C | G | 0.622 | 0.0021 | 0.002 | 2.41E-01 |
| rs3791710 | T | C | 0.799 | 0.0054 | 0.002 | 1.13E-02 |
| rs3795310 | C | T | 0.514 | -0.0031 | 0.002 | 7.05E-02 |
| rs3802924 | A | C | 0.804 | -0.0084 | 0.002 | 6.65E-05 |
| rs3814883 | C | T | 0.551 | 0.0005 | 0.002 | 7.92E-01 |
| rs3824451 | T | C | 0.832 | 0.0027 | 0.002 | 2.44E-01 |
| rs4129585 | A | C | 0.446 | -0.0114 | 0.002 | 2.82E-11 |
| rs4575535 | A | G | 0.279 | -0.0023 | 0.002 | 2.18E-01 |
| rs4632195 | C | T | 0.492 | 0.0104 | 0.002 | 1.11E-09 |
| rs4636654 | G | A | 0.626 | -0.0066 | 0.002 | 1.60E-04 |
| rs4653164 | C | T | 0.320 | -0.0002 | 0.002 | 8.96E-01 |
| rs4702 | G | A | 0.407 | 0.0001 | 0.002 | 9.61E-01 |
| rs4766428 | C | T | 0.548 | 0.0016 | 0.002 | 3.38E-01 |
| rs4779050 | T | G | 0.390 | -0.0032 | 0.002 | 6.79E-02 |
| rs4812325 | G | A | 0.442 | -0.0052 | 0.002 | 2.67E-03 |
| rs4921741 | A | G | 0.770 | -0.0042 | 0.002 | 3.60E-02 |
| rs498591 | A | T | 0.869 | 0.0035 | 0.002 | 1.46E-01 |
| rs500102 | T | C | 0.378 | 0.0020 | 0.002 | 2.58E-01 |
| rs505061 | C | A | 0.512 | -0.0003 | 0.002 | 8.41E-01 |
| rs56205728 | G | A | 0.696 | -0.0029 | 0.002 | 1.30E-01 |
| rs56335113 | A | G | 0.323 | 0.0029 | 0.002 | 1.14E-01 |
| rs57433322 | C | G | 0.890 | 0.0164 | 0.003 | 1.72E-09 |
| rs5751191 | T | C | 0.522 | 0.0062 | 0.002 | 2.61E-04 |
| rs58120505 | T | C | 0.556 | 0.0056 | 0.002 | 1.12E-03 |
| rs60135207 | G | T | 0.554 | -0.0128 | 0.002 | 7.97E-14 |
| rs6125656 | G | A | 0.804 | -0.0002 | 0.002 | 9.16E-01 |
| rs61857878 | A | T | 0.753 | 0.0079 | 0.002 | 7.44E-05 |
| rs61937595 | C | T | 0.901 | -0.0047 | 0.003 | 1.20E-01 |
| rs62018952 | T | C | 0.296 | 0.0044 | 0.002 | 2.12E-02 |
| rs62183855 | A | C | 0.787 | -0.0019 | 0.002 | 3.81E-01 |
| rs634940 | G | T | 0.748 | -0.0033 | 0.002 | 9.36E-02 |
| rs6482437 | A | C | 0.088 | -0.0087 | 0.003 | 1.17E-03 |
| rs6520064 | A | G | 0.786 | -0.0024 | 0.002 | 2.55E-01 |
| rs6538539 | G | T | 0.447 | -0.0029 | 0.002 | 8.69E-02 |
| rs6546857 | A | G | 0.794 | 0.0083 | 0.002 | 3.05E-05 |
| rs6549963 | T | C | 0.611 | -0.0006 | 0.002 | 7.11E-01 |
| rs6673880 | A | G | 0.526 | -0.0018 | 0.002 | 3.09E-01 |
| rs6715366 | G | A | 0.735 | -0.0006 | 0.002 | 7.36E-01 |
| rs6798742 | A | G | 0.670 | 0.0002 | 0.002 | 9.19E-01 |
| rs6943762 | T | C | 0.869 | 0.0030 | 0.003 | 2.41E-01 |
| rs6974218 | A | C | 0.590 | 0.0059 | 0.002 | 7.28E-04 |
| rs6984242 | G | A | 0.383 | -0.0006 | 0.002 | 7.38E-01 |
| rs708228 | C | T | 0.680 | 0.0097 | 0.002 | 8.65E-08 |
| rs7112616 | T | C | 0.498 | 0.0060 | 0.002 | 4.23E-04 |
| rs713692 | G | A | 0.328 | -0.0051 | 0.002 | 5.59E-03 |
| rs7251 | C | G | 0.692 | -0.0007 | 0.002 | 7.22E-01 |
| rs72802868 | G | T | 0.726 | 0.0097 | 0.002 | 1.75E-07 |
| rs728055 | T | A | 0.638 | 0.0128 | 0.002 | 9.15E-13 |
| rs72943392 | G | C | 0.738 | 0.0014 | 0.002 | 4.70E-01 |
| rs72986630 | C | T | 0.942 | -0.0128 | 0.004 | 4.46E-04 |
| rs73229090 | C | A | 0.896 | -0.0015 | 0.003 | 5.60E-01 |
| rs73292401 | T | A | 0.823 | 0.0071 | 0.002 | 1.04E-03 |
| rs7515363 | C | T | 0.345 | 0.0003 | 0.002 | 8.63E-01 |
| rs7575796 | A | G | 0.951 | 0.0075 | 0.003 | 2.40E-02 |
| rs7634476 | A | G | 0.401 | 0.0016 | 0.002 | 3.64E-01 |
| rs7647398 | C | T | 0.789 | 0.0003 | 0.002 | 8.76E-01 |
| rs76838079 | C | T | 0.849 | -0.0012 | 0.002 | 6.39E-01 |
| rs778371 | A | G | 0.682 | -0.0100 | 0.002 | 1.22E-07 |
| rs7798283 | T | G | 0.859 | -0.0059 | 0.003 | 2.22E-02 |
| rs7830315 | T | C | 0.531 | -0.0044 | 0.002 | 9.35E-03 |
| rs79210963 | T | C | 0.883 | -0.0229 | 0.003 | 3.36E-18 |
| rs79445414 | T | C | 0.944 | 0.0027 | 0.004 | 5.03E-01 |
| rs8055219 | G | A | 0.750 | -0.0084 | 0.002 | 3.73E-05 |
| rs9304548 | C | A | 0.257 | 0.0005 | 0.002 | 7.86E-01 |
| rs9318627 | A | C | 0.619 | -0.0001 | 0.002 | 9.44E-01 |
| rs9461916 | T | C | 0.342 | -0.0026 | 0.002 | 1.40E-01 |
| rs9636107 | A | G | 0.524 | 0.0037 | 0.002 | 2.87E-02 |
| rs9687282 | T | G | 0.670 | 0.0004 | 0.002 | 8.42E-01 |
| rs9876421 | C | T | 0.624 | -0.0052 | 0.002 | 3.72E-03 |

Abbreviations: EAF, effect allele frequency; GWAS, genome-wide association studies; MR, Mendelian randomisation; SE, standard error; SNP, single nucleotide polymorphism.

## Table S6. Summary information on genetic instruments for schizophrenia-income MR analysis in schizophrenia GWAS dataset.

| **SNP** | **Effect Allele** | **Alternate Allele** | **EAF** | **Beta** | **SE** | ***p* value** | ***R*^2^** | ***F* statistics** | **Overall *R*^2^** | **Mean *F* statistics** |
| --- | --- | --- | --- | --- | --- | --- | --- | --- | --- | --- |
| rs1000237 | T | A | 0.627 | -0.073 | 0.009 | 2.80E-16 | 0.0005 | 67.656 | 0.050 | 44.722 |
| rs10035564 | A | G | 0.650 | -0.067 | 0.009 | 4.38E-13 | 0.0004 | 52.724 |  |  |
| rs10086619 | A | G | 0.831 | -0.072 | 0.012 | 4.97E-10 | 0.0003 | 38.745 |  |  |
| rs10108980 | C | T | 0.785 | -0.063 | 0.011 | 2.73E-09 | 0.0003 | 35.102 |  |  |
| rs10117 | G | A | 0.613 | 0.055 | 0.009 | 4.66E-10 | 0.0003 | 39.062 |  |  |
| rs10861176 | G | A | 0.257 | -0.056 | 0.010 | 1.59E-08 | 0.0002 | 32.075 |  |  |
| rs10873538 | T | G | 0.654 | -0.067 | 0.009 | 3.01E-13 | 0.0004 | 53.407 |  |  |
| rs10876446 | G | C | 0.674 | -0.054 | 0.009 | 1.03E-08 | 0.0003 | 33.004 |  |  |
| rs11027839 | A | C | 0.488 | -0.052 | 0.009 | 2.40E-09 | 0.0003 | 35.866 |  |  |
| rs11136325 | G | A | 0.436 | 0.054 | 0.009 | 3.05E-09 | 0.0003 | 34.948 |  |  |
| rs11165867 | C | T | 0.829 | -0.074 | 0.012 | 1.30E-10 | 0.0003 | 41.030 |  |  |
| rs11191580 | T | C | 0.920 | 0.132 | 0.016 | 1.77E-17 | 0.0006 | 72.199 |  |  |
| rs11210892 | G | A | 0.347 | 0.064 | 0.009 | 2.68E-12 | 0.0004 | 48.694 |  |  |
| rs11223774 | A | G | 0.301 | 0.052 | 0.009 | 2.74E-08 | 0.0002 | 31.191 |  |  |
| rs113264400 | T | C | 0.948 | -0.112 | 0.020 | 2.87E-08 | 0.0002 | 30.905 |  |  |
| rs11534045 | G | A | 0.686 | 0.063 | 0.009 | 1.40E-11 | 0.0003 | 45.594 |  |  |
| rs11587347 | C | G | 0.895 | -0.104 | 0.015 | 1.53E-12 | 0.0004 | 49.952 |  |  |
| rs11664298 | G | A | 0.794 | -0.077 | 0.011 | 8.94E-13 | 0.0004 | 51.360 |  |  |
| rs11693094 | C | T | 0.556 | 0.054 | 0.009 | 4.29E-10 | 0.0003 | 39.103 |  |  |
| rs117178087 | C | T | 0.939 | 0.096 | 0.018 | 4.89E-08 | 0.0002 | 29.663 |  |  |
| rs11941714 | G | A | 0.673 | 0.052 | 0.009 | 3.07E-08 | 0.0002 | 30.780 |  |  |
| rs1198588 | A | T | 0.194 | -0.103 | 0.011 | 1.73E-21 | 0.0007 | 90.246 |  |  |
| rs12129573 | C | A | 0.616 | -0.078 | 0.009 | 2.28E-18 | 0.0006 | 76.414 |  |  |
| rs12138231 | T | A | 0.171 | -0.067 | 0.012 | 7.99E-09 | 0.0003 | 33.355 |  |  |
| rs12151767 | G | A | 0.524 | 0.061 | 0.009 | 1.31E-12 | 0.0004 | 50.484 |  |  |
| rs12285419 | C | A | 0.800 | -0.085 | 0.011 | 1.05E-14 | 0.0005 | 59.577 |  |  |
| rs12293670 | A | G | 0.678 | 0.070 | 0.009 | 1.56E-14 | 0.0004 | 58.715 |  |  |
| rs12303743 | G | C | 0.898 | -0.087 | 0.015 | 1.59E-09 | 0.0003 | 36.414 |  |  |
| rs12489270 | T | C | 0.612 | -0.058 | 0.009 | 7.47E-11 | 0.0003 | 42.330 |  |  |
| rs12652777 | T | C | 0.489 | 0.049 | 0.009 | 1.52E-08 | 0.0002 | 32.199 |  |  |
| rs12712510 | T | C | 0.488 | 0.057 | 0.009 | 5.14E-11 | 0.0003 | 43.531 |  |  |
| rs12771371 | G | A | 0.697 | 0.052 | 0.009 | 1.94E-08 | 0.0002 | 31.750 |  |  |
| rs12833624 | C | T | 0.646 | -0.050 | 0.009 | 2.77E-08 | 0.0002 | 31.111 |  |  |
| rs12877581 | G | C | 0.716 | -0.060 | 0.010 | 1.80E-09 | 0.0003 | 36.245 |  |  |
| rs12883788 | C | T | 0.527 | -0.061 | 0.009 | 1.86E-12 | 0.0004 | 49.648 |  |  |
| rs13016542 | T | C | 0.876 | 0.088 | 0.013 | 8.28E-12 | 0.0004 | 46.858 |  |  |
| rs13107325 | C | T | 0.919 | -0.159 | 0.017 | 2.90E-21 | 0.0007 | 89.238 |  |  |
| rs13195636 | A | C | 0.926 | 0.211 | 0.016 | 6.55E-40 | 0.0013 | 175.278 |  |  |
| rs13233308 | C | T | 0.526 | 0.049 | 0.009 | 1.75E-08 | 0.0002 | 32.073 |  |  |
| rs132582 | C | T | 0.471 | 0.051 | 0.009 | 3.26E-09 | 0.0003 | 35.164 |  |  |
| rs1430894 | C | T | 0.506 | -0.053 | 0.009 | 6.15E-10 | 0.0003 | 38.404 |  |  |
| rs145071536 | T | C | 0.799 | -0.085 | 0.012 | 1.62E-12 | 0.0004 | 50.292 |  |  |
| rs1451488 | A | G | 0.432 | -0.071 | 0.009 | 4.47E-16 | 0.0005 | 66.403 |  |  |
| rs149165 | T | G | 0.569 | 0.048 | 0.009 | 3.01E-08 | 0.0002 | 30.694 |  |  |
| rs1593304 | A | G | 0.193 | -0.064 | 0.011 | 7.45E-09 | 0.0003 | 33.349 |  |  |
| rs1604060 | A | G | 0.103 | -0.077 | 0.014 | 3.24E-08 | 0.0002 | 30.411 |  |  |
| rs1615350 | C | T | 0.273 | 0.074 | 0.010 | 4.92E-14 | 0.0004 | 56.409 |  |  |
| rs167924 | A | G | 0.359 | -0.050 | 0.009 | 2.34E-08 | 0.0002 | 31.111 |  |  |
| rs16851048 | T | C | 0.792 | -0.074 | 0.011 | 4.15E-12 | 0.0004 | 48.474 |  |  |
| rs16867571 | A | G | 0.778 | 0.066 | 0.010 | 2.68E-10 | 0.0003 | 39.913 |  |  |
| rs17016552 | C | G | 0.661 | 0.052 | 0.009 | 1.20E-08 | 0.0002 | 32.278 |  |  |
| rs17194490 | G | T | 0.827 | -0.078 | 0.012 | 1.80E-11 | 0.0003 | 45.446 |  |  |
| rs17731 | G | A | 0.620 | -0.052 | 0.009 | 4.37E-09 | 0.0003 | 34.663 |  |  |
| rs187557 | C | T | 0.163 | 0.067 | 0.012 | 2.03E-08 | 0.0002 | 31.412 |  |  |
| rs1881046 | G | T | 0.670 | 0.051 | 0.009 | 3.39E-08 | 0.0002 | 30.373 |  |  |
| rs1901512 | T | C | 0.318 | 0.058 | 0.009 | 5.72E-10 | 0.0003 | 38.600 |  |  |
| rs1915019 | A | G | 0.265 | 0.057 | 0.010 | 6.57E-09 | 0.0003 | 33.947 |  |  |
| rs2053079 | A | G | 0.754 | -0.060 | 0.010 | 3.01E-09 | 0.0003 | 35.172 |  |  |
| rs2078266 | A | G | 0.178 | 0.070 | 0.013 | 2.94E-08 | 0.0002 | 30.513 |  |  |
| rs215412 | G | A | 0.661 | -0.058 | 0.009 | 2.69E-10 | 0.0003 | 40.209 |  |  |
| rs217336 | C | A | 0.583 | 0.050 | 0.009 | 8.05E-09 | 0.0003 | 33.431 |  |  |
| rs2238057 | T | G | 0.568 | -0.084 | 0.009 | 8.50E-22 | 0.0007 | 92.118 |  |  |
| rs2252074 | T | G | 0.590 | -0.069 | 0.009 | 6.19E-15 | 0.0005 | 60.599 |  |  |
| rs2332700 | C | G | 0.258 | 0.075 | 0.010 | 3.88E-14 | 0.0004 | 57.542 |  |  |
| rs2333321 | A | G | 0.218 | 0.071 | 0.011 | 1.25E-11 | 0.0004 | 45.986 |  |  |
| rs2381411 | T | C | 0.586 | -0.050 | 0.009 | 1.25E-08 | 0.0003 | 32.800 |  |  |
| rs2455415 | C | T | 0.577 | -0.049 | 0.009 | 1.69E-08 | 0.0002 | 31.634 |  |  |
| rs2456020 | C | T | 0.773 | 0.082 | 0.010 | 1.13E-15 | 0.0005 | 63.998 |  |  |
| rs2514218 | C | T | 0.668 | 0.070 | 0.009 | 1.35E-14 | 0.0004 | 58.715 |  |  |
| rs2710323 | T | C | 0.531 | 0.078 | 0.009 | 1.23E-19 | 0.0006 | 83.116 |  |  |
| rs2815731 | C | A | 0.660 | 0.060 | 0.009 | 4.39E-11 | 0.0003 | 43.478 |  |  |
| rs2909457 | G | A | 0.451 | 0.049 | 0.009 | 1.48E-08 | 0.0002 | 31.721 |  |  |
| rs2999392 | C | T | 0.299 | -0.052 | 0.009 | 3.05E-08 | 0.0002 | 30.366 |  |  |
| rs308697 | C | A | 0.574 | 0.050 | 0.009 | 8.83E-09 | 0.0003 | 33.166 |  |  |
| rs35351411 | A | C | 0.439 | -0.064 | 0.009 | 2.21E-13 | 0.0004 | 53.281 |  |  |
| rs35734242 | T | C | 0.562 | -0.051 | 0.009 | 1.37E-08 | 0.0002 | 32.457 |  |  |
| rs3739118 | G | A | 0.719 | 0.057 | 0.010 | 2.36E-09 | 0.0003 | 36.005 |  |  |
| rs3770754 | C | G | 0.645 | 0.053 | 0.009 | 5.35E-09 | 0.0003 | 33.788 |  |  |
| rs3791710 | T | C | 0.804 | 0.060 | 0.011 | 3.02E-08 | 0.0002 | 30.868 |  |  |
| rs3795310 | C | T | 0.543 | 0.051 | 0.009 | 5.75E-09 | 0.0003 | 34.360 |  |  |
| rs3802924 | A | C | 0.805 | 0.074 | 0.011 | 9.58E-12 | 0.0004 | 46.446 |  |  |
| rs3814883 | C | T | 0.548 | 0.067 | 0.009 | 1.58E-14 | 0.0005 | 59.481 |  |  |
| rs3824451 | T | C | 0.838 | -0.066 | 0.012 | 2.54E-08 | 0.0002 | 30.901 |  |  |
| rs4129585 | A | C | 0.456 | 0.075 | 0.009 | 5.11E-18 | 0.0006 | 74.309 |  |  |
| rs4575535 | A | G | 0.281 | -0.056 | 0.010 | 5.77E-09 | 0.0003 | 33.783 |  |  |
| rs4632195 | C | T | 0.471 | -0.047 | 0.009 | 4.59E-08 | 0.0002 | 30.118 |  |  |
| rs4636654 | G | A | 0.608 | 0.048 | 0.009 | 4.89E-08 | 0.0002 | 29.457 |  |  |
| rs4653164 | C | T | 0.323 | -0.051 | 0.009 | 3.08E-08 | 0.0002 | 30.855 |  |  |
| rs4702 | G | A | 0.461 | 0.084 | 0.009 | 2.79E-21 | 0.0007 | 89.726 |  |  |
| rs4766428 | C | T | 0.539 | -0.075 | 0.009 | 3.93E-17 | 0.0005 | 71.021 |  |  |
| rs4779050 | T | G | 0.381 | 0.058 | 0.009 | 7.27E-11 | 0.0003 | 42.462 |  |  |
| rs4812325 | G | A | 0.370 | -0.072 | 0.009 | 8.96E-16 | 0.0005 | 65.272 |  |  |
| rs4921741 | A | G | 0.729 | -0.056 | 0.010 | 1.21E-08 | 0.0002 | 32.652 |  |  |
| rs498591 | A | T | 0.847 | -0.072 | 0.012 | 2.11E-09 | 0.0003 | 35.896 |  |  |
| rs500102 | T | C | 0.413 | 0.052 | 0.009 | 4.87E-09 | 0.0003 | 34.516 |  |  |
| rs505061 | C | A | 0.495 | -0.053 | 0.009 | 5.80E-10 | 0.0003 | 38.694 |  |  |
| rs56205728 | G | A | 0.700 | -0.063 | 0.010 | 1.01E-10 | 0.0003 | 42.188 |  |  |
| rs56335113 | A | G | 0.316 | 0.065 | 0.009 | 6.02E-12 | 0.0004 | 47.377 |  |  |
| rs57433322 | C | G | 0.885 | 0.083 | 0.014 | 1.99E-09 | 0.0003 | 35.741 |  |  |
| rs5751191 | T | C | 0.484 | -0.066 | 0.009 | 3.00E-14 | 0.0004 | 58.176 |  |  |
| rs58120505 | T | C | 0.602 | 0.090 | 0.009 | 2.24E-24 | 0.0008 | 103.676 |  |  |
| rs60135207 | G | T | 0.596 | 0.050 | 0.009 | 1.53E-08 | 0.0002 | 31.768 |  |  |
| rs6125656 | G | A | 0.809 | -0.064 | 0.011 | 6.29E-09 | 0.0003 | 33.761 |  |  |
| rs61857878 | A | T | 0.760 | 0.060 | 0.010 | 4.44E-09 | 0.0003 | 34.715 |  |  |
| rs61937595 | C | T | 0.917 | 0.130 | 0.016 | 1.15E-15 | 0.0005 | 64.493 |  |  |
| rs62018952 | T | C | 0.264 | -0.058 | 0.010 | 1.94E-09 | 0.0003 | 36.251 |  |  |
| rs62183855 | A | C | 0.814 | 0.066 | 0.011 | 2.66E-09 | 0.0003 | 35.458 |  |  |
| rs634940 | G | T | 0.737 | -0.066 | 0.010 | 1.78E-11 | 0.0003 | 44.980 |  |  |
| rs6482437 | A | C | 0.099 | -0.099 | 0.014 | 3.33E-12 | 0.0004 | 48.512 |  |  |
| rs6520064 | A | G | 0.787 | -0.058 | 0.011 | 3.58E-08 | 0.0002 | 30.456 |  |  |
| rs6538539 | G | T | 0.462 | 0.057 | 0.009 | 4.43E-11 | 0.0003 | 43.615 |  |  |
| rs6546857 | A | G | 0.757 | -0.060 | 0.010 | 2.74E-09 | 0.0003 | 35.062 |  |  |
| rs6549963 | T | C | 0.596 | 0.048 | 0.009 | 4.31E-08 | 0.0002 | 30.131 |  |  |
| rs6673880 | A | G | 0.492 | -0.062 | 0.009 | 7.20E-12 | 0.0004 | 46.871 |  |  |
| rs6715366 | G | A | 0.723 | -0.054 | 0.010 | 2.49E-08 | 0.0002 | 31.103 |  |  |
| rs6798742 | A | G | 0.680 | -0.061 | 0.009 | 4.57E-11 | 0.0003 | 43.162 |  |  |
| rs6943762 | T | C | 0.883 | 0.105 | 0.013 | 1.57E-15 | 0.0005 | 63.394 |  |  |
| rs6974218 | A | C | 0.634 | 0.055 | 0.009 | 6.80E-10 | 0.0003 | 38.044 |  |  |
| rs6984242 | G | A | 0.413 | 0.055 | 0.009 | 3.86E-10 | 0.0003 | 39.526 |  |  |
| rs708228 | C | T | 0.661 | -0.053 | 0.009 | 6.56E-09 | 0.0003 | 33.665 |  |  |
| rs7112616 | T | C | 0.515 | 0.052 | 0.009 | 1.52E-09 | 0.0003 | 36.847 |  |  |
| rs713692 | G | A | 0.299 | -0.057 | 0.010 | 2.67E-09 | 0.0003 | 35.499 |  |  |
| rs7251 | C | G | 0.682 | 0.064 | 0.009 | 8.29E-12 | 0.0004 | 46.502 |  |  |
| rs72802868 | G | T | 0.724 | 0.069 | 0.010 | 4.55E-13 | 0.0004 | 51.959 |  |  |
| rs728055 | T | A | 0.658 | 0.067 | 0.009 | 8.85E-14 | 0.0004 | 56.078 |  |  |
| rs72943392 | G | C | 0.707 | -0.053 | 0.010 | 2.39E-08 | 0.0002 | 31.052 |  |  |
| rs72986630 | C | T | 0.926 | -0.112 | 0.018 | 3.59E-10 | 0.0003 | 39.357 |  |  |
| rs73229090 | C | A | 0.897 | 0.103 | 0.014 | 4.34E-13 | 0.0004 | 52.207 |  |  |
| rs73292401 | T | A | 0.799 | -0.068 | 0.011 | 5.48E-10 | 0.0003 | 38.468 |  |  |
| rs7515363 | C | T | 0.389 | 0.054 | 0.009 | 1.84E-09 | 0.0003 | 36.139 |  |  |
| rs7575796 | A | G | 0.917 | 0.096 | 0.017 | 2.07E-08 | 0.0002 | 31.347 |  |  |
| rs7634476 | A | G | 0.397 | -0.058 | 0.009 | 5.46E-11 | 0.0003 | 42.997 |  |  |
| rs7647398 | C | T | 0.811 | 0.077 | 0.011 | 1.07E-12 | 0.0004 | 50.551 |  |  |
| rs76838079 | C | T | 0.849 | -0.078 | 0.014 | 1.53E-08 | 0.0002 | 31.951 |  |  |
| rs778371 | A | G | 0.699 | -0.081 | 0.010 | 1.50E-17 | 0.0006 | 71.987 |  |  |
| rs7798283 | T | G | 0.875 | 0.074 | 0.013 | 3.49E-08 | 0.0002 | 30.499 |  |  |
| rs7830315 | T | C | 0.478 | -0.048 | 0.009 | 3.08E-08 | 0.0002 | 30.899 |  |  |
| rs79210963 | T | C | 0.884 | -0.086 | 0.014 | 4.14E-10 | 0.0003 | 39.041 |  |  |
| rs79445414 | T | C | 0.956 | -0.123 | 0.022 | 2.80E-08 | 0.0002 | 30.898 |  |  |
| rs8055219 | G | A | 0.758 | -0.067 | 0.010 | 5.69E-11 | 0.0003 | 43.355 |  |  |
| rs9304548 | C | A | 0.261 | 0.057 | 0.010 | 1.59E-08 | 0.0002 | 32.151 |  |  |
| rs9318627 | A | C | 0.613 | 0.061 | 0.009 | 4.35E-12 | 0.0004 | 48.363 |  |  |
| rs9461916 | T | C | 0.388 | -0.053 | 0.009 | 1.64E-09 | 0.0003 | 36.679 |  |  |
| rs9636107 | A | G | 0.506 | -0.070 | 0.009 | 5.12E-16 | 0.0005 | 66.057 |  |  |
| rs9687282 | T | G | 0.650 | -0.053 | 0.009 | 7.33E-09 | 0.0003 | 33.410 |  |  |
| rs9876421 | C | T | 0.645 | -0.063 | 0.009 | 9.19E-12 | 0.0004 | 46.156 |  |  |

Abbreviations: EAF, effect allele frequency; GWAS, genome-wide association studies; MR, Mendelian randomisation; SE, standard error; SNP, single nucleotide polymorphism.

## Table S7. Summary information on genetic instruments for schizophrenia-income MR analysis in income GWAS dataset.

| **SNP** | **Effect Allele** | **Alternate Allele** | **EAF** | **Beta** | **SE** | ***p*** **value** |
| --- | --- | --- | --- | --- | --- | --- |
| rs1000237 | T | A | 0.645 | 0.0032 | 0.003 | 2.40E-01 |
| rs10035564 | A | G | 0.672 | 0.0049 | 0.003 | 8.20E-02 |
| rs10086619 | A | G | 0.832 | -0.0025 | 0.004 | 4.80E-01 |
| rs10108980 | C | T | 0.809 | 0.0004 | 0.003 | 8.90E-01 |
| rs10117 | G | A | 0.604 | -0.0134 | 0.003 | 6.80E-07 |
| rs10861176 | G | A | 0.267 | -0.0040 | 0.003 | 1.80E-01 |
| rs10873538 | T | G | 0.661 | 0.0021 | 0.003 | 4.60E-01 |
| rs10876446 | G | C | 0.683 | 0.0034 | 0.003 | 2.30E-01 |
| rs11027839 | A | C | 0.492 | 0.0009 | 0.003 | 7.30E-01 |
| rs11136325 | G | A | 0.400 | 0.0011 | 0.003 | 6.90E-01 |
| rs11165867 | C | T | 0.839 | -0.0014 | 0.004 | 7.00E-01 |
| rs11191580 | T | C | 0.922 | -0.0070 | 0.005 | 1.50E-01 |
| rs11210892 | G | A | 0.325 | -0.0157 | 0.003 | 2.50E-08 |
| rs11223774 | A | G | 0.292 | 0.0007 | 0.003 | 8.00E-01 |
| rs113264400 | T | C | 0.953 | 0.0078 | 0.006 | 2.20E-01 |
| rs11534045 | G | A | 0.689 | -0.0015 | 0.003 | 6.00E-01 |
| rs11587347 | C | G | 0.902 | 0.0055 | 0.004 | 2.20E-01 |
| rs11664298 | G | A | 0.791 | 0.0098 | 0.003 | 2.60E-03 |
| rs11693094 | C | T | 0.535 | 0.0013 | 0.003 | 6.10E-01 |
| rs117178087 | C | T | 0.935 | -0.0008 | 0.005 | 8.80E-01 |
| rs11941714 | G | A | 0.673 | -0.0033 | 0.003 | 2.50E-01 |
| rs1198588 | A | T | 0.203 | -0.0060 | 0.003 | 7.00E-02 |
| rs12129573 | C | A | 0.651 | 0.0038 | 0.003 | 1.70E-01 |
| rs12138231 | T | A | 0.185 | 0.0017 | 0.003 | 6.30E-01 |
| rs12151767 | G | A | 0.521 | 0.0059 | 0.003 | 2.60E-02 |
| rs12285419 | C | A | 0.805 | -0.0006 | 0.003 | 8.60E-01 |
| rs12293670 | A | G | 0.665 | 0.0019 | 0.003 | 5.10E-01 |
| rs12303743 | G | C | 0.902 | 0.0063 | 0.004 | 1.50E-01 |
| rs12489270 | T | C | 0.630 | 0.0085 | 0.003 | 2.10E-03 |
| rs12652777 | T | C | 0.488 | -0.0081 | 0.003 | 2.30E-03 |
| rs12712510 | T | C | 0.464 | -0.0033 | 0.003 | 2.20E-01 |
| rs12771371 | G | A | 0.684 | 0.0014 | 0.003 | 6.20E-01 |
| rs12833624 | C | T | 0.663 | 0.0042 | 0.003 | 1.30E-01 |
| rs12877581 | G | C | 0.718 | 0.00004 | 0.003 | 9.90E-01 |
| rs12883788 | C | T | 0.540 | 0.0189 | 0.003 | 1.40E-12 |
| rs13016542 | T | C | 0.873 | -0.0030 | 0.004 | 4.50E-01 |
| rs13107325 | C | T | 0.925 | 0.0251 | 0.005 | 6.30E-07 |
| rs13195636 | A | C | 0.885 | -0.0060 | 0.004 | 1.50E-01 |
| rs13233308 | C | T | 0.517 | -0.0005 | 0.003 | 8.60E-01 |
| rs132582 | C | T | 0.441 | -0.0083 | 0.003 | 2.00E-03 |
| rs1430894 | C | T | 0.525 | 0.0072 | 0.003 | 6.40E-03 |
| rs145071536 | T | C | 0.809 | 0.0039 | 0.003 | 2.60E-01 |
| rs1451488 | A | G | 0.443 | -0.0009 | 0.003 | 7.40E-01 |
| rs149165 | T | G | 0.534 | -0.0014 | 0.003 | 5.90E-01 |
| rs1593304 | A | G | 0.207 | -0.0053 | 0.003 | 1.10E-01 |
| rs1604060 | A | G | 0.112 | 0.0114 | 0.004 | 6.80E-03 |
| rs1615350 | C | T | 0.249 | 0.0081 | 0.003 | 7.70E-03 |
| rs167924 | A | G | 0.376 | 0.0048 | 0.003 | 8.20E-02 |
| rs16851048 | T | C | 0.807 | 0.0016 | 0.003 | 6.30E-01 |
| rs16867571 | A | G | 0.759 | 0.0044 | 0.003 | 1.60E-01 |
| rs17016552 | C | G | 0.646 | -0.0015 | 0.003 | 5.90E-01 |
| rs17194490 | G | T | 0.835 | -0.0001 | 0.004 | 9.80E-01 |
| rs17731 | G | A | 0.627 | 0.0041 | 0.003 | 1.40E-01 |
| rs187557 | C | T | 0.151 | 0.0100 | 0.004 | 6.90E-03 |
| rs1881046 | G | T | 0.657 | -0.0031 | 0.003 | 2.70E-01 |
| rs1901512 | T | C | 0.306 | -0.0069 | 0.003 | 1.80E-02 |
| rs1915019 | A | G | 0.240 | 0.0003 | 0.003 | 9.20E-01 |
| rs2053079 | A | G | 0.750 | 0.0087 | 0.003 | 4.50E-03 |
| rs2078266 | A | G | 0.169 | -0.0012 | 0.004 | 7.50E-01 |
| rs215412 | G | A | 0.675 | 0.0092 | 0.003 | 1.20E-03 |
| rs217336 | C | A | 0.562 | -0.0045 | 0.003 | 8.90E-02 |
| rs2238057 | T | G | 0.581 | -0.0022 | 0.003 | 4.10E-01 |
| rs2252074 | T | G | 0.601 | 0.0064 | 0.003 | 1.70E-02 |
| rs2332700 | C | G | 0.248 | 0.0033 | 0.003 | 2.80E-01 |
| rs2333321 | A | G | 0.207 | -0.0050 | 0.003 | 1.30E-01 |
| rs2381411 | T | C | 0.597 | 0.0014 | 0.003 | 6.20E-01 |
| rs2455415 | C | T | 0.570 | -0.0001 | 0.003 | 9.70E-01 |
| rs2456020 | C | T | 0.770 | -0.0124 | 0.003 | 7.50E-05 |
| rs2514218 | C | T | 0.644 | -0.0022 | 0.003 | 4.20E-01 |
| rs2710323 | T | C | 0.528 | -0.0041 | 0.003 | 1.30E-01 |
| rs2815731 | C | A | 0.657 | 0.0024 | 0.003 | 4.00E-01 |
| rs2909457 | G | A | 0.465 | -0.0044 | 0.003 | 9.50E-02 |
| rs2999392 | C | T | 0.303 | -0.0026 | 0.003 | 3.70E-01 |
| rs308697 | C | A | 0.562 | 0.0017 | 0.003 | 5.30E-01 |
| rs35351411 | A | C | 0.445 | 0.0004 | 0.003 | 8.80E-01 |
| rs35734242 | T | C | 0.571 | 0.0007 | 0.003 | 8.00E-01 |
| rs3739118 | G | A | 0.712 | -0.0066 | 0.003 | 2.40E-02 |
| rs3770754 | C | G | 0.640 | -0.0036 | 0.003 | 1.90E-01 |
| rs3791710 | T | C | 0.797 | 0.0014 | 0.003 | 6.80E-01 |
| rs3795310 | C | T | 0.548 | -0.0069 | 0.003 | 9.40E-03 |
| rs3802924 | A | C | 0.802 | -0.0074 | 0.003 | 2.70E-02 |
| rs3814883 | C | T | 0.517 | 0.0005 | 0.003 | 8.50E-01 |
| rs3824451 | T | C | 0.839 | 0.0031 | 0.004 | 3.80E-01 |
| rs4129585 | A | C | 0.439 | -0.0092 | 0.003 | 5.30E-04 |
| rs4575535 | A | G | 0.306 | -0.0031 | 0.003 | 2.80E-01 |
| rs4632195 | C | T | 0.488 | 0.0136 | 0.003 | 2.90E-07 |
| rs4636654 | G | A | 0.604 | -0.0096 | 0.003 | 3.90E-04 |
| rs4653164 | C | T | 0.309 | -0.0018 | 0.003 | 5.30E-01 |
| rs4702 | G | A | 0.444 | 0.0056 | 0.003 | 3.50E-02 |
| rs4766428 | C | T | 0.568 | -0.0030 | 0.003 | 2.70E-01 |
| rs4779050 | T | G | 0.371 | -0.0004 | 0.003 | 8.90E-01 |
| rs4812325 | G | A | 0.390 | -0.0018 | 0.003 | 5.00E-01 |
| rs4921741 | A | G | 0.762 | -0.0062 | 0.003 | 4.60E-02 |
| rs498591 | A | T | 0.858 | 0.0098 | 0.004 | 9.70E-03 |
| rs500102 | T | C | 0.402 | 0.0022 | 0.003 | 4.20E-01 |
| rs505061 | C | A | 0.514 | -0.0015 | 0.003 | 5.70E-01 |
| rs56205728 | G | A | 0.713 | -0.0018 | 0.003 | 5.50E-01 |
| rs56335113 | A | G | 0.309 | -0.0004 | 0.003 | 8.90E-01 |
| rs57433322 | C | G | 0.883 | 0.0021 | 0.004 | 6.10E-01 |
| rs5751191 | T | C | 0.496 | 0.0053 | 0.003 | 4.80E-02 |
| rs58120505 | T | C | 0.566 | -0.0046 | 0.003 | 8.20E-02 |
| rs60135207 | G | T | 0.571 | -0.0139 | 0.003 | 2.10E-07 |
| rs6125656 | G | A | 0.818 | 0.0041 | 0.003 | 2.30E-01 |
| rs61857878 | A | T | 0.743 | -0.0039 | 0.003 | 2.00E-01 |
| rs61937595 | C | T | 0.906 | -0.0033 | 0.005 | 4.80E-01 |
| rs62018952 | T | C | 0.273 | 0.0081 | 0.003 | 6.70E-03 |
| rs62183855 | A | C | 0.795 | -0.0011 | 0.003 | 7.40E-01 |
| rs634940 | G | T | 0.737 | -0.0065 | 0.003 | 3.20E-02 |
| rs6482437 | A | C | 0.111 | -0.0086 | 0.004 | 4.10E-02 |
| rs6520064 | A | G | 0.798 | 0.0004 | 0.003 | 9.00E-01 |
| rs6538539 | G | T | 0.446 | -0.0073 | 0.003 | 5.90E-03 |
| rs6546857 | A | G | 0.762 | 0.0140 | 0.003 | 6.70E-06 |
| rs6549963 | T | C | 0.574 | 0.0054 | 0.003 | 4.70E-02 |
| rs6673880 | A | G | 0.513 | 0.0026 | 0.003 | 3.30E-01 |
| rs6715366 | G | A | 0.724 | -0.0016 | 0.003 | 5.90E-01 |
| rs6798742 | A | G | 0.702 | 0.0010 | 0.003 | 7.40E-01 |
| rs6943762 | T | C | 0.873 | -0.0073 | 0.004 | 6.60E-02 |
| rs6974218 | A | C | 0.617 | -0.0063 | 0.003 | 2.00E-02 |
| rs6984242 | G | A | 0.401 | -0.0028 | 0.003 | 2.90E-01 |
| rs708228 | C | T | 0.672 | 0.0149 | 0.003 | 1.40E-07 |
| rs7112616 | T | C | 0.499 | 0.0051 | 0.003 | 5.60E-02 |
| rs713692 | G | A | 0.315 | 0.0019 | 0.003 | 5.10E-01 |
| rs7251 | C | G | 0.680 | 0.0048 | 0.003 | 8.90E-02 |
| rs72802868 | G | T | 0.702 | -0.0025 | 0.003 | 4.00E-01 |
| rs728055 | T | A | 0.632 | 0.0021 | 0.003 | 4.40E-01 |
| rs72943392 | G | C | 0.718 | 0.0015 | 0.003 | 6.10E-01 |
| rs72986630 | C | T | 0.936 | -0.0009 | 0.006 | 8.70E-01 |
| rs73229090 | C | A | 0.883 | -0.0068 | 0.004 | 1.00E-01 |
| rs73292401 | T | A | 0.809 | 0.0077 | 0.003 | 2.30E-02 |
| rs7515363 | C | T | 0.385 | -0.0014 | 0.003 | 6.00E-01 |
| rs7575796 | A | G | 0.921 | 0.0003 | 0.005 | 9.60E-01 |
| rs7634476 | A | G | 0.398 | 0.0059 | 0.003 | 2.90E-02 |
| rs7647398 | C | T | 0.800 | -0.0024 | 0.003 | 4.70E-01 |
| rs76838079 | C | T | 0.836 | -0.0005 | 0.004 | 8.80E-01 |
| rs778371 | A | G | 0.721 | -0.0011 | 0.003 | 7.00E-01 |
| rs7798283 | T | G | 0.872 | -0.0053 | 0.004 | 1.90E-01 |
| rs7830315 | T | C | 0.502 | -0.0030 | 0.003 | 2.60E-01 |
| rs79210963 | T | C | 0.882 | -0.0020 | 0.004 | 6.30E-01 |
| rs79445414 | T | C | 0.953 | 0.0088 | 0.006 | 1.70E-01 |
| rs8055219 | G | A | 0.774 | 0.0065 | 0.003 | 4.00E-02 |
| rs9304548 | C | A | 0.260 | 0.0013 | 0.003 | 6.60E-01 |
| rs9318627 | A | C | 0.592 | 0.0028 | 0.003 | 3.00E-01 |
| rs9461916 | T | C | 0.410 | 0.0035 | 0.003 | 2.00E-01 |
| rs9636107 | A | G | 0.529 | 0.0065 | 0.003 | 1.50E-02 |
| rs9687282 | T | G | 0.666 | 0.0007 | 0.003 | 8.00E-01 |
| rs9876421 | C | T | 0.657 | 0.0009 | 0.003 | 7.60E-01 |

Abbreviations: EAF, effect allele frequency; GWAS, genome-wide association studies; MR, Mendelian randomisation; SE, standard error; SNP, single nucleotide polymorphism.

## Table S8. Summary information on genetic instruments for schizophrenia-employment status MR analysis in schizophrenia GWAS dataset.

| **SNP** | **Effect Allele** | **Alternate Allele** | **EAF** | **Beta** | **SE** | ***p* value** | ***R*^2^** | ***F* statistics** | **Overall *R*^2^** | **Mean *F* statistics** |
| --- | --- | --- | --- | --- | --- | --- | --- | --- | --- | --- |
| rs1000237 | T | A | 0.627 | -0.073 | 0.009 | 2.80E-16 | 0.0005 | 67.656 | 0.050 | 44.722 |
| rs10035564 | A | G | 0.650 | -0.067 | 0.009 | 4.38E-13 | 0.0004 | 52.724 |  |  |
| rs10086619 | A | G | 0.831 | -0.072 | 0.012 | 4.97E-10 | 0.0003 | 38.745 |  |  |
| rs10108980 | C | T | 0.785 | -0.063 | 0.011 | 2.73E-09 | 0.0003 | 35.102 |  |  |
| rs10117 | G | A | 0.613 | 0.055 | 0.009 | 4.66E-10 | 0.0003 | 39.062 |  |  |
| rs10861176 | G | A | 0.257 | -0.056 | 0.010 | 1.59E-08 | 0.0002 | 32.075 |  |  |
| rs10873538 | T | G | 0.654 | -0.067 | 0.009 | 3.01E-13 | 0.0004 | 53.407 |  |  |
| rs10876446 | G | C | 0.674 | -0.054 | 0.009 | 1.03E-08 | 0.0003 | 33.004 |  |  |
| rs11027839 | A | C | 0.488 | -0.052 | 0.009 | 2.40E-09 | 0.0003 | 35.866 |  |  |
| rs11136325 | G | A | 0.436 | 0.054 | 0.009 | 3.05E-09 | 0.0003 | 34.948 |  |  |
| rs11165867 | C | T | 0.829 | -0.074 | 0.012 | 1.30E-10 | 0.0003 | 41.030 |  |  |
| rs11191580 | T | C | 0.920 | 0.132 | 0.016 | 1.77E-17 | 0.0006 | 72.199 |  |  |
| rs11210892 | G | A | 0.347 | 0.064 | 0.009 | 2.68E-12 | 0.0004 | 48.694 |  |  |
| rs11223774 | A | G | 0.301 | 0.052 | 0.009 | 2.74E-08 | 0.0002 | 31.191 |  |  |
| rs113264400 | T | C | 0.948 | -0.112 | 0.020 | 2.87E-08 | 0.0002 | 30.905 |  |  |
| rs11534045 | G | A | 0.686 | 0.063 | 0.009 | 1.40E-11 | 0.0003 | 45.594 |  |  |
| rs11587347 | C | G | 0.895 | -0.104 | 0.015 | 1.53E-12 | 0.0004 | 49.952 |  |  |
| rs11664298 | G | A | 0.794 | -0.077 | 0.011 | 8.94E-13 | 0.0004 | 51.360 |  |  |
| rs11693094 | C | T | 0.556 | 0.054 | 0.009 | 4.29E-10 | 0.0003 | 39.103 |  |  |
| rs117178087 | C | T | 0.939 | 0.096 | 0.018 | 4.89E-08 | 0.0002 | 29.663 |  |  |
| rs11941714 | G | A | 0.673 | 0.052 | 0.009 | 3.07E-08 | 0.0002 | 30.780 |  |  |
| rs1198588 | A | T | 0.194 | -0.103 | 0.011 | 1.73E-21 | 0.0007 | 90.246 |  |  |
| rs12129573 | C | A | 0.616 | -0.078 | 0.009 | 2.28E-18 | 0.0006 | 76.414 |  |  |
| rs12138231 | T | A | 0.171 | -0.067 | 0.012 | 7.99E-09 | 0.0003 | 33.355 |  |  |
| rs12151767 | G | A | 0.524 | 0.061 | 0.009 | 1.31E-12 | 0.0004 | 50.484 |  |  |
| rs12285419 | C | A | 0.800 | -0.085 | 0.011 | 1.05E-14 | 0.0005 | 59.577 |  |  |
| rs12293670 | A | G | 0.678 | 0.070 | 0.009 | 1.56E-14 | 0.0004 | 58.715 |  |  |
| rs12303743 | G | C | 0.898 | -0.087 | 0.015 | 1.59E-09 | 0.0003 | 36.414 |  |  |
| rs12489270 | T | C | 0.612 | -0.058 | 0.009 | 7.47E-11 | 0.0003 | 42.330 |  |  |
| rs12652777 | T | C | 0.489 | 0.049 | 0.009 | 1.52E-08 | 0.0002 | 32.199 |  |  |
| rs12712510 | T | C | 0.488 | 0.057 | 0.009 | 5.14E-11 | 0.0003 | 43.531 |  |  |
| rs12771371 | G | A | 0.697 | 0.052 | 0.009 | 1.94E-08 | 0.0002 | 31.750 |  |  |
| rs12833624 | C | T | 0.646 | -0.050 | 0.009 | 2.77E-08 | 0.0002 | 31.111 |  |  |
| rs12877581 | G | C | 0.716 | -0.060 | 0.010 | 1.80E-09 | 0.0003 | 36.245 |  |  |
| rs12883788 | C | T | 0.527 | -0.061 | 0.009 | 1.86E-12 | 0.0004 | 49.648 |  |  |
| rs13016542 | T | C | 0.876 | 0.088 | 0.013 | 8.28E-12 | 0.0004 | 46.858 |  |  |
| rs13107325 | C | T | 0.919 | -0.159 | 0.017 | 2.90E-21 | 0.0007 | 89.238 |  |  |
| rs13195636 | A | C | 0.926 | 0.211 | 0.016 | 6.55E-40 | 0.0013 | 175.278 |  |  |
| rs13233308 | C | T | 0.526 | 0.049 | 0.009 | 1.75E-08 | 0.0002 | 32.073 |  |  |
| rs132582 | C | T | 0.471 | 0.051 | 0.009 | 3.26E-09 | 0.0003 | 35.164 |  |  |
| rs1430894 | C | T | 0.506 | -0.053 | 0.009 | 6.15E-10 | 0.0003 | 38.404 |  |  |
| rs145071536 | T | C | 0.799 | -0.085 | 0.012 | 1.62E-12 | 0.0004 | 50.292 |  |  |
| rs1451488 | A | G | 0.432 | -0.071 | 0.009 | 4.47E-16 | 0.0005 | 66.403 |  |  |
| rs149165 | T | G | 0.569 | 0.048 | 0.009 | 3.01E-08 | 0.0002 | 30.694 |  |  |
| rs1593304 | A | G | 0.193 | -0.064 | 0.011 | 7.45E-09 | 0.0003 | 33.349 |  |  |
| rs1604060 | A | G | 0.103 | -0.077 | 0.014 | 3.24E-08 | 0.0002 | 30.411 |  |  |
| rs1615350 | C | T | 0.273 | 0.074 | 0.010 | 4.92E-14 | 0.0004 | 56.409 |  |  |
| rs167924 | A | G | 0.359 | -0.050 | 0.009 | 2.34E-08 | 0.0002 | 31.111 |  |  |
| rs16851048 | T | C | 0.792 | -0.074 | 0.011 | 4.15E-12 | 0.0004 | 48.474 |  |  |
| rs16867571 | A | G | 0.778 | 0.066 | 0.010 | 2.68E-10 | 0.0003 | 39.913 |  |  |
| rs17016552 | C | G | 0.661 | 0.052 | 0.009 | 1.20E-08 | 0.0002 | 32.278 |  |  |
| rs17194490 | G | T | 0.827 | -0.078 | 0.012 | 1.80E-11 | 0.0003 | 45.446 |  |  |
| rs17731 | G | A | 0.620 | -0.052 | 0.009 | 4.37E-09 | 0.0003 | 34.663 |  |  |
| rs187557 | C | T | 0.163 | 0.067 | 0.012 | 2.03E-08 | 0.0002 | 31.412 |  |  |
| rs1881046 | G | T | 0.670 | 0.051 | 0.009 | 3.39E-08 | 0.0002 | 30.373 |  |  |
| rs1901512 | T | C | 0.318 | 0.058 | 0.009 | 5.72E-10 | 0.0003 | 38.600 |  |  |
| rs1915019 | A | G | 0.265 | 0.057 | 0.010 | 6.57E-09 | 0.0003 | 33.947 |  |  |
| rs2053079 | A | G | 0.754 | -0.060 | 0.010 | 3.01E-09 | 0.0003 | 35.172 |  |  |
| rs2078266 | A | G | 0.178 | 0.070 | 0.013 | 2.94E-08 | 0.0002 | 30.513 |  |  |
| rs215412 | G | A | 0.661 | -0.058 | 0.009 | 2.69E-10 | 0.0003 | 40.209 |  |  |
| rs217336 | C | A | 0.583 | 0.050 | 0.009 | 8.05E-09 | 0.0003 | 33.431 |  |  |
| rs2238057 | T | G | 0.568 | -0.084 | 0.009 | 8.50E-22 | 0.0007 | 92.118 |  |  |
| rs2252074 | T | G | 0.590 | -0.069 | 0.009 | 6.19E-15 | 0.0005 | 60.599 |  |  |
| rs2332700 | C | G | 0.258 | 0.075 | 0.010 | 3.88E-14 | 0.0004 | 57.542 |  |  |
| rs2333321 | A | G | 0.218 | 0.071 | 0.011 | 1.25E-11 | 0.0004 | 45.986 |  |  |
| rs2381411 | T | C | 0.586 | -0.050 | 0.009 | 1.25E-08 | 0.0003 | 32.800 |  |  |
| rs2455415 | C | T | 0.577 | -0.049 | 0.009 | 1.69E-08 | 0.0002 | 31.634 |  |  |
| rs2456020 | C | T | 0.773 | 0.082 | 0.010 | 1.13E-15 | 0.0005 | 63.998 |  |  |
| rs2514218 | C | T | 0.668 | 0.070 | 0.009 | 1.35E-14 | 0.0004 | 58.715 |  |  |
| rs2710323 | T | C | 0.531 | 0.078 | 0.009 | 1.23E-19 | 0.0006 | 83.116 |  |  |
| rs2815731 | C | A | 0.660 | 0.060 | 0.009 | 4.39E-11 | 0.0003 | 43.478 |  |  |
| rs2909457 | G | A | 0.451 | 0.049 | 0.009 | 1.48E-08 | 0.0002 | 31.721 |  |  |
| rs2999392 | C | T | 0.299 | -0.052 | 0.009 | 3.05E-08 | 0.0002 | 30.366 |  |  |
| rs308697 | C | A | 0.574 | 0.050 | 0.009 | 8.83E-09 | 0.0003 | 33.166 |  |  |
| rs35351411 | A | C | 0.439 | -0.064 | 0.009 | 2.21E-13 | 0.0004 | 53.281 |  |  |
| rs35734242 | T | C | 0.562 | -0.051 | 0.009 | 1.37E-08 | 0.0002 | 32.457 |  |  |
| rs3739118 | G | A | 0.719 | 0.057 | 0.010 | 2.36E-09 | 0.0003 | 36.005 |  |  |
| rs3770754 | C | G | 0.645 | 0.053 | 0.009 | 5.35E-09 | 0.0003 | 33.788 |  |  |
| rs3791710 | T | C | 0.804 | 0.060 | 0.011 | 3.02E-08 | 0.0002 | 30.868 |  |  |
| rs3795310 | C | T | 0.543 | 0.051 | 0.009 | 5.75E-09 | 0.0003 | 34.360 |  |  |
| rs3802924 | A | C | 0.805 | 0.074 | 0.011 | 9.58E-12 | 0.0004 | 46.446 |  |  |
| rs3814883 | C | T | 0.548 | 0.067 | 0.009 | 1.58E-14 | 0.0005 | 59.481 |  |  |
| rs3824451 | T | C | 0.838 | -0.066 | 0.012 | 2.54E-08 | 0.0002 | 30.901 |  |  |
| rs4129585 | A | C | 0.456 | 0.075 | 0.009 | 5.11E-18 | 0.0006 | 74.309 |  |  |
| rs4575535 | A | G | 0.281 | -0.056 | 0.010 | 5.77E-09 | 0.0003 | 33.783 |  |  |
| rs4632195 | C | T | 0.471 | -0.047 | 0.009 | 4.59E-08 | 0.0002 | 30.118 |  |  |
| rs4636654 | G | A | 0.608 | 0.048 | 0.009 | 4.89E-08 | 0.0002 | 29.457 |  |  |
| rs4653164 | C | T | 0.323 | -0.051 | 0.009 | 3.08E-08 | 0.0002 | 30.855 |  |  |
| rs4702 | G | A | 0.461 | 0.084 | 0.009 | 2.79E-21 | 0.0007 | 89.726 |  |  |
| rs4766428 | C | T | 0.539 | -0.075 | 0.009 | 3.93E-17 | 0.0005 | 71.021 |  |  |
| rs4779050 | T | G | 0.381 | 0.058 | 0.009 | 7.27E-11 | 0.0003 | 42.462 |  |  |
| rs4812325 | G | A | 0.370 | -0.072 | 0.009 | 8.96E-16 | 0.0005 | 65.272 |  |  |
| rs4921741 | A | G | 0.729 | -0.056 | 0.010 | 1.21E-08 | 0.0002 | 32.652 |  |  |
| rs498591 | A | T | 0.847 | -0.072 | 0.012 | 2.11E-09 | 0.0003 | 35.896 |  |  |
| rs500102 | T | C | 0.413 | 0.052 | 0.009 | 4.87E-09 | 0.0003 | 34.516 |  |  |
| rs505061 | C | A | 0.495 | -0.053 | 0.009 | 5.80E-10 | 0.0003 | 38.694 |  |  |
| rs56205728 | G | A | 0.700 | -0.063 | 0.010 | 1.01E-10 | 0.0003 | 42.188 |  |  |
| rs56335113 | A | G | 0.316 | 0.065 | 0.009 | 6.02E-12 | 0.0004 | 47.377 |  |  |
| rs57433322 | C | G | 0.885 | 0.083 | 0.014 | 1.99E-09 | 0.0003 | 35.741 |  |  |
| rs5751191 | T | C | 0.484 | -0.066 | 0.009 | 3.00E-14 | 0.0004 | 58.176 |  |  |
| rs58120505 | T | C | 0.602 | 0.090 | 0.009 | 2.24E-24 | 0.0008 | 103.676 |  |  |
| rs60135207 | G | T | 0.596 | 0.050 | 0.009 | 1.53E-08 | 0.0002 | 31.768 |  |  |
| rs6125656 | G | A | 0.809 | -0.064 | 0.011 | 6.29E-09 | 0.0003 | 33.761 |  |  |
| rs61857878 | A | T | 0.760 | 0.060 | 0.010 | 4.44E-09 | 0.0003 | 34.715 |  |  |
| rs61937595 | C | T | 0.917 | 0.130 | 0.016 | 1.15E-15 | 0.0005 | 64.493 |  |  |
| rs62018952 | T | C | 0.264 | -0.058 | 0.010 | 1.94E-09 | 0.0003 | 36.251 |  |  |
| rs62183855 | A | C | 0.814 | 0.066 | 0.011 | 2.66E-09 | 0.0003 | 35.458 |  |  |
| rs634940 | G | T | 0.737 | -0.066 | 0.010 | 1.78E-11 | 0.0003 | 44.980 |  |  |
| rs6482437 | A | C | 0.099 | -0.099 | 0.014 | 3.33E-12 | 0.0004 | 48.512 |  |  |
| rs6520064 | A | G | 0.787 | -0.058 | 0.011 | 3.58E-08 | 0.0002 | 30.456 |  |  |
| rs6538539 | G | T | 0.462 | 0.057 | 0.009 | 4.43E-11 | 0.0003 | 43.615 |  |  |
| rs6546857 | A | G | 0.757 | -0.060 | 0.010 | 2.74E-09 | 0.0003 | 35.062 |  |  |
| rs6549963 | T | C | 0.596 | 0.048 | 0.009 | 4.31E-08 | 0.0002 | 30.131 |  |  |
| rs6673880 | A | G | 0.492 | -0.062 | 0.009 | 7.20E-12 | 0.0004 | 46.871 |  |  |
| rs6715366 | G | A | 0.723 | -0.054 | 0.010 | 2.49E-08 | 0.0002 | 31.103 |  |  |
| rs6798742 | A | G | 0.680 | -0.061 | 0.009 | 4.57E-11 | 0.0003 | 43.162 |  |  |
| rs6943762 | T | C | 0.883 | 0.105 | 0.013 | 1.57E-15 | 0.0005 | 63.394 |  |  |
| rs6974218 | A | C | 0.634 | 0.055 | 0.009 | 6.80E-10 | 0.0003 | 38.044 |  |  |
| rs6984242 | G | A | 0.413 | 0.055 | 0.009 | 3.86E-10 | 0.0003 | 39.526 |  |  |
| rs708228 | C | T | 0.661 | -0.053 | 0.009 | 6.56E-09 | 0.0003 | 33.665 |  |  |
| rs7112616 | T | C | 0.515 | 0.052 | 0.009 | 1.52E-09 | 0.0003 | 36.847 |  |  |
| rs713692 | G | A | 0.299 | -0.057 | 0.010 | 2.67E-09 | 0.0003 | 35.499 |  |  |
| rs7251 | C | G | 0.682 | 0.064 | 0.009 | 8.29E-12 | 0.0004 | 46.502 |  |  |
| rs72802868 | G | T | 0.724 | 0.069 | 0.010 | 4.55E-13 | 0.0004 | 51.959 |  |  |
| rs728055 | T | A | 0.658 | 0.067 | 0.009 | 8.85E-14 | 0.0004 | 56.078 |  |  |
| rs72943392 | G | C | 0.707 | -0.053 | 0.010 | 2.39E-08 | 0.0002 | 31.052 |  |  |
| rs72986630 | C | T | 0.926 | -0.112 | 0.018 | 3.59E-10 | 0.0003 | 39.357 |  |  |
| rs73229090 | C | A | 0.897 | 0.103 | 0.014 | 4.34E-13 | 0.0004 | 52.207 |  |  |
| rs73292401 | T | A | 0.799 | -0.068 | 0.011 | 5.48E-10 | 0.0003 | 38.468 |  |  |
| rs7515363 | C | T | 0.389 | 0.054 | 0.009 | 1.84E-09 | 0.0003 | 36.139 |  |  |
| rs7575796 | A | G | 0.917 | 0.096 | 0.017 | 2.07E-08 | 0.0002 | 31.347 |  |  |
| rs7634476 | A | G | 0.397 | -0.058 | 0.009 | 5.46E-11 | 0.0003 | 42.997 |  |  |
| rs7647398 | C | T | 0.811 | 0.077 | 0.011 | 1.07E-12 | 0.0004 | 50.551 |  |  |
| rs76838079 | C | T | 0.849 | -0.078 | 0.014 | 1.53E-08 | 0.0002 | 31.951 |  |  |
| rs778371 | A | G | 0.699 | -0.081 | 0.010 | 1.50E-17 | 0.0006 | 71.987 |  |  |
| rs7798283 | T | G | 0.875 | 0.074 | 0.013 | 3.49E-08 | 0.0002 | 30.499 |  |  |
| rs7830315 | T | C | 0.478 | -0.048 | 0.009 | 3.08E-08 | 0.0002 | 30.899 |  |  |
| rs79210963 | T | C | 0.884 | -0.086 | 0.014 | 4.14E-10 | 0.0003 | 39.041 |  |  |
| rs79445414 | T | C | 0.956 | -0.123 | 0.022 | 2.80E-08 | 0.0002 | 30.898 |  |  |
| rs8055219 | G | A | 0.758 | -0.067 | 0.010 | 5.69E-11 | 0.0003 | 43.355 |  |  |
| rs9304548 | C | A | 0.261 | 0.057 | 0.010 | 1.59E-08 | 0.0002 | 32.151 |  |  |
| rs9318627 | A | C | 0.613 | 0.061 | 0.009 | 4.35E-12 | 0.0004 | 48.363 |  |  |
| rs9461916 | T | C | 0.388 | -0.053 | 0.009 | 1.64E-09 | 0.0003 | 36.679 |  |  |
| rs9636107 | A | G | 0.506 | -0.070 | 0.009 | 5.12E-16 | 0.0005 | 66.057 |  |  |
| rs9687282 | T | G | 0.650 | -0.053 | 0.009 | 7.33E-09 | 0.0003 | 33.410 |  |  |
| rs9876421 | C | T | 0.645 | -0.063 | 0.009 | 9.19E-12 | 0.0004 | 46.156 |  |  |

Abbreviations: EAF, effect allele frequency; GWAS, genome-wide association studies; MR, Mendelian randomisation; SE, standard error; SNP, single nucleotide polymorphism.

## Table S9. Summary information on genetic instruments for schizophrenia-employment status MR analysis in employment status GWAS dataset.

| **SNP** | **Effect Allele** | **Alternate Allele** | **EAF** | **Beta** | **SE** | ***p* value** |
| --- | --- | --- | --- | --- | --- | --- |
| rs1000237 | T | A | 0.645 | 0.0022 | 0.001 | 4.10E-02 |
| rs10035564 | A | G | 0.672 | -0.0013 | 0.001 | 2.50E-01 |
| rs10086619 | A | G | 0.832 | 0.0010 | 0.001 | 4.90E-01 |
| rs10108980 | C | T | 0.809 | -0.0011 | 0.001 | 4.20E-01 |
| rs10117 | G | A | 0.604 | -0.0035 | 0.001 | 8.60E-04 |
| rs10861176 | G | A | 0.267 | -0.0025 | 0.001 | 3.40E-02 |
| rs10873538 | T | G | 0.661 | -0.0005 | 0.001 | 6.20E-01 |
| rs10876446 | G | C | 0.683 | 0.0029 | 0.001 | 8.80E-03 |
| rs11027839 | A | C | 0.492 | -0.0006 | 0.001 | 5.40E-01 |
| rs11136325 | G | A | 0.400 | 0.0008 | 0.001 | 4.30E-01 |
| rs11165867 | C | T | 0.839 | -0.0028 | 0.001 | 4.70E-02 |
| rs11191580 | T | C | 0.922 | -0.0038 | 0.002 | 4.50E-02 |
| rs11210892 | G | A | 0.326 | -0.0009 | 0.001 | 4.10E-01 |
| rs11223774 | A | G | 0.292 | -0.0001 | 0.001 | 9.00E-01 |
| rs113264400 | T | C | 0.953 | -0.0021 | 0.002 | 3.80E-01 |
| rs11534045 | G | A | 0.689 | -0.0022 | 0.001 | 4.80E-02 |
| rs11587347 | C | G | 0.902 | -0.0015 | 0.002 | 3.80E-01 |
| rs11664298 | G | A | 0.791 | 0.0004 | 0.001 | 7.60E-01 |
| rs11693094 | C | T | 0.535 | -0.0008 | 0.001 | 4.30E-01 |
| rs117178087 | C | T | 0.935 | 0.0005 | 0.002 | 8.20E-01 |
| rs11941714 | G | A | 0.673 | -0.0010 | 0.001 | 3.80E-01 |
| rs1198588 | A | T | 0.203 | 0.0006 | 0.001 | 6.10E-01 |
| rs12129573 | C | A | 0.651 | -0.0001 | 0.001 | 9.10E-01 |
| rs12138231 | T | A | 0.184 | 0.0007 | 0.001 | 5.90E-01 |
| rs12151767 | G | A | 0.521 | -0.0011 | 0.001 | 3.10E-01 |
| rs12285419 | C | A | 0.805 | 0.0008 | 0.001 | 5.30E-01 |
| rs12293670 | A | G | 0.665 | -0.0018 | 0.001 | 9.60E-02 |
| rs12303743 | G | C | 0.902 | -0.0017 | 0.002 | 3.30E-01 |
| rs12489270 | T | C | 0.630 | -0.0003 | 0.001 | 7.60E-01 |
| rs12652777 | T | C | 0.488 | 0.0003 | 0.001 | 7.50E-01 |
| rs12712510 | T | C | 0.464 | 0.0002 | 0.001 | 8.70E-01 |
| rs12771371 | G | A | 0.684 | 0.0011 | 0.001 | 3.30E-01 |
| rs12833624 | C | T | 0.663 | 0.0022 | 0.001 | 4.40E-02 |
| rs12877581 | G | C | 0.718 | -0.0002 | 0.001 | 8.90E-01 |
| rs12883788 | C | T | 0.540 | 0.0020 | 0.001 | 4.90E-02 |
| rs13016542 | T | C | 0.873 | 0.0015 | 0.002 | 3.50E-01 |
| rs13107325 | C | T | 0.925 | 0.0081 | 0.002 | 3.80E-05 |
| rs13195636 | A | C | 0.885 | 0.0003 | 0.002 | 8.50E-01 |
| rs13233308 | C | T | 0.517 | -0.0022 | 0.001 | 3.00E-02 |
| rs132582 | C | T | 0.441 | 0.0005 | 0.001 | 6.30E-01 |
| rs1430894 | C | T | 0.525 | 0.0003 | 0.001 | 7.90E-01 |
| rs145071536 | T | C | 0.809 | 0.0015 | 0.001 | 2.50E-01 |
| rs1451488 | A | G | 0.443 | 0.0005 | 0.001 | 6.10E-01 |
| rs149165 | T | G | 0.534 | 0.0013 | 0.001 | 2.10E-01 |
| rs1593304 | A | G | 0.207 | -0.0004 | 0.001 | 7.40E-01 |
| rs1604060 | A | G | 0.112 | 0.0035 | 0.002 | 3.40E-02 |
| rs1615350 | C | T | 0.249 | -0.0015 | 0.001 | 2.10E-01 |
| rs167924 | A | G | 0.376 | 0.0011 | 0.001 | 2.90E-01 |
| rs16851048 | T | C | 0.807 | -2.20E-05 | 0.001 | 9.90E-01 |
| rs16867571 | A | G | 0.759 | -0.0009 | 0.001 | 4.80E-01 |
| rs17016552 | C | G | 0.645 | -0.0009 | 0.001 | 4.10E-01 |
| rs17194490 | G | T | 0.835 | -0.0003 | 0.001 | 8.10E-01 |
| rs17731 | G | A | 0.627 | 0.0012 | 0.001 | 2.40E-01 |
| rs187557 | C | T | 0.151 | -0.0005 | 0.001 | 7.40E-01 |
| rs1881046 | G | T | 0.657 | -0.0022 | 0.001 | 3.90E-02 |
| rs1901512 | T | C | 0.306 | -0.0001 | 0.001 | 9.10E-01 |
| rs1915019 | A | G | 0.240 | -0.0008 | 0.001 | 5.10E-01 |
| rs2053079 | A | G | 0.750 | 0.0015 | 0.001 | 2.20E-01 |
| rs2078266 | A | G | 0.170 | -0.0008 | 0.001 | 6.00E-01 |
| rs215412 | G | A | 0.675 | 0.0010 | 0.001 | 3.70E-01 |
| rs217336 | C | A | 0.563 | 0.0006 | 0.001 | 5.90E-01 |
| rs2238057 | T | G | 0.581 | -0.0005 | 0.001 | 6.00E-01 |
| rs2252074 | T | G | 0.601 | 0.0008 | 0.001 | 4.60E-01 |
| rs2332700 | C | G | 0.248 | 0.0002 | 0.001 | 8.60E-01 |
| rs2333321 | A | G | 0.207 | -0.0011 | 0.001 | 3.80E-01 |
| rs2381411 | T | C | 0.597 | 0.0021 | 0.001 | 4.60E-02 |
| rs2455415 | C | T | 0.570 | 0.0007 | 0.001 | 4.80E-01 |
| rs2456020 | C | T | 0.770 | -0.0035 | 0.001 | 4.30E-03 |
| rs2514218 | C | T | 0.644 | -0.0041 | 0.001 | 1.50E-04 |
| rs2710323 | T | C | 0.528 | -0.0011 | 0.001 | 3.00E-01 |
| rs2815731 | C | A | 0.657 | -7.77E-07 | 0.001 | 1.00E+00 |
| rs2909457 | G | A | 0.465 | 0.0002 | 0.001 | 8.20E-01 |
| rs2999392 | C | T | 0.303 | -0.0011 | 0.001 | 3.30E-01 |
| rs308697 | C | A | 0.561 | 0.0012 | 0.001 | 2.50E-01 |
| rs35351411 | A | C | 0.445 | -0.0003 | 0.001 | 8.00E-01 |
| rs35734242 | T | C | 0.571 | 0.0010 | 0.001 | 3.30E-01 |
| rs3739118 | G | A | 0.712 | -0.0001 | 0.001 | 9.30E-01 |
| rs3770754 | C | G | 0.641 | 0.0003 | 0.001 | 7.60E-01 |
| rs3791710 | T | C | 0.797 | -0.0023 | 0.001 | 7.50E-02 |
| rs3795310 | C | T | 0.548 | -0.0008 | 0.001 | 4.50E-01 |
| rs3802924 | A | C | 0.802 | 0.0007 | 0.001 | 6.00E-01 |
| rs3814883 | C | T | 0.518 | -0.0044 | 0.001 | 1.80E-05 |
| rs3824451 | T | C | 0.839 | -0.0013 | 0.001 | 3.60E-01 |
| rs4129585 | A | C | 0.439 | -0.0030 | 0.001 | 3.40E-03 |
| rs4575535 | A | G | 0.306 | 0.0007 | 0.001 | 5.30E-01 |
| rs4632195 | C | T | 0.488 | 0.0009 | 0.001 | 4.00E-01 |
| rs4636654 | G | A | 0.604 | -0.0038 | 0.001 | 3.30E-04 |
| rs4653164 | C | T | 0.309 | 0.0010 | 0.001 | 3.50E-01 |
| rs4702 | G | A | 0.444 | -0.0007 | 0.001 | 5.00E-01 |
| rs4766428 | C | T | 0.568 | -0.0010 | 0.001 | 3.30E-01 |
| rs4779050 | T | G | 0.371 | -0.0004 | 0.001 | 7.30E-01 |
| rs4812325 | G | A | 0.391 | -0.0004 | 0.001 | 6.90E-01 |
| rs4921741 | A | G | 0.762 | -0.0020 | 0.001 | 1.00E-01 |
| rs498591 | A | T | 0.858 | 0.0002 | 0.001 | 9.10E-01 |
| rs500102 | T | C | 0.402 | 0.0007 | 0.001 | 5.10E-01 |
| rs505061 | C | A | 0.514 | -0.0024 | 0.001 | 2.00E-02 |
| rs56205728 | G | A | 0.714 | 0.0005 | 0.001 | 6.70E-01 |
| rs56335113 | A | G | 0.309 | -0.0009 | 0.001 | 4.10E-01 |
| rs57433322 | C | G | 0.883 | -0.0032 | 0.002 | 4.40E-02 |
| rs5751191 | T | C | 0.496 | 0.0008 | 0.001 | 4.30E-01 |
| rs58120505 | T | C | 0.566 | -0.0015 | 0.001 | 1.60E-01 |
| rs60135207 | G | T | 0.572 | -0.0012 | 0.001 | 2.30E-01 |
| rs6125656 | G | A | 0.818 | 0.0008 | 0.001 | 5.50E-01 |
| rs61857878 | A | T | 0.743 | 0.0012 | 0.001 | 3.00E-01 |
| rs61937595 | C | T | 0.906 | -0.0039 | 0.002 | 2.80E-02 |
| rs62018952 | T | C | 0.273 | 0.0015 | 0.001 | 2.00E-01 |
| rs62183855 | A | C | 0.795 | 0.0003 | 0.001 | 8.00E-01 |
| rs634940 | G | T | 0.737 | -0.0007 | 0.001 | 5.70E-01 |
| rs6482437 | A | C | 0.111 | 0.0008 | 0.002 | 6.40E-01 |
| rs6520064 | A | G | 0.798 | -0.0003 | 0.001 | 8.10E-01 |
| rs6538539 | G | T | 0.446 | -0.0014 | 0.001 | 1.90E-01 |
| rs6546857 | A | G | 0.762 | 0.0019 | 0.001 | 1.20E-01 |
| rs6549963 | T | C | 0.574 | 0.0014 | 0.001 | 1.90E-01 |
| rs6673880 | A | G | 0.513 | -0.0001 | 0.001 | 9.50E-01 |
| rs6715366 | G | A | 0.724 | 0.0006 | 0.001 | 6.30E-01 |
| rs6798742 | A | G | 0.702 | -2.29E-05 | 0.001 | 9.80E-01 |
| rs6943762 | T | C | 0.873 | -0.0034 | 0.002 | 2.60E-02 |
| rs6974218 | A | C | 0.616 | -0.0002 | 0.001 | 8.60E-01 |
| rs6984242 | G | A | 0.401 | -0.0011 | 0.001 | 3.20E-01 |
| rs708228 | C | T | 0.672 | 0.0018 | 0.001 | 1.00E-01 |
| rs7112616 | T | C | 0.499 | -0.0002 | 0.001 | 8.30E-01 |
| rs713692 | G | A | 0.315 | -0.0010 | 0.001 | 3.60E-01 |
| rs7251 | C | G | 0.680 | -0.0013 | 0.001 | 2.50E-01 |
| rs72802868 | G | T | 0.702 | 0.0006 | 0.001 | 6.20E-01 |
| rs728055 | T | A | 0.633 | -0.0007 | 0.001 | 5.50E-01 |
| rs72943392 | G | C | 0.718 | 0.0004 | 0.001 | 7.10E-01 |
| rs72986630 | C | T | 0.935 | -0.0005 | 0.002 | 8.20E-01 |
| rs73229090 | C | A | 0.883 | -0.0016 | 0.002 | 3.30E-01 |
| rs73292401 | T | A | 0.808 | 0.0023 | 0.001 | 8.40E-02 |
| rs7515363 | C | T | 0.385 | -0.0002 | 0.001 | 8.40E-01 |
| rs7575796 | A | G | 0.921 | -0.0002 | 0.002 | 9.30E-01 |
| rs7634476 | A | G | 0.398 | 0.0018 | 0.001 | 8.80E-02 |
| rs7647398 | C | T | 0.800 | 0.0016 | 0.001 | 2.30E-01 |
| rs76838079 | C | T | 0.836 | -0.0009 | 0.001 | 5.00E-01 |
| rs778371 | A | G | 0.721 | 0.0013 | 0.001 | 2.60E-01 |
| rs7798283 | T | G | 0.872 | 0.0020 | 0.002 | 2.00E-01 |
| rs7830315 | T | C | 0.502 | -0.0012 | 0.001 | 2.60E-01 |
| rs79210963 | T | C | 0.883 | -0.0008 | 0.002 | 6.20E-01 |
| rs79445414 | T | C | 0.953 | -0.0023 | 0.002 | 3.50E-01 |
| rs8055219 | G | A | 0.775 | 0.0003 | 0.001 | 7.90E-01 |
| rs9304548 | C | A | 0.260 | 0.0012 | 0.001 | 2.90E-01 |
| rs9318627 | A | C | 0.593 | 0.0009 | 0.001 | 4.10E-01 |
| rs9461916 | T | C | 0.410 | 0.0011 | 0.001 | 3.20E-01 |
| rs9636107 | A | G | 0.529 | 0.0008 | 0.001 | 4.20E-01 |
| rs9687282 | T | G | 0.666 | -0.0002 | 0.001 | 8.60E-01 |
| rs9876421 | C | T | 0.658 | -0.0012 | 0.001 | 2.80E-01 |

Abbreviations: EAF, effect allele frequency; GWAS, genome-wide association studies; MR, Mendelian randomisation; SE, standard error; SNP, single nucleotide polymorphism.

## Table S10. Summary information on genetic instruments for schizophrenia-TDI MR analysis in schizophrenia GWAS dataset.

| **SNP** | **Effect Allele** | **Alternate Allele** | **EAF** | **Beta** | **SE** | ***p* value** | ***R*^2^** | ***F* statistics** | **Overall *R*^2^** | **Mean *F* statistics** |
| --- | --- | --- | --- | --- | --- | --- | --- | --- | --- | --- |
| rs1000237 | T | A | 0.627 | -0.073 | 0.009 | 2.80E-16 | 0.0005 | 67.656 | 0.050 | 44.722 |
| rs10035564 | A | G | 0.650 | -0.067 | 0.009 | 4.38E-13 | 0.0004 | 52.724 |  |  |
| rs10086619 | A | G | 0.831 | -0.072 | 0.012 | 4.97E-10 | 0.0003 | 38.745 |  |  |
| rs10108980 | C | T | 0.785 | -0.063 | 0.011 | 2.73E-09 | 0.0003 | 35.102 |  |  |
| rs10117 | G | A | 0.613 | 0.055 | 0.009 | 4.66E-10 | 0.0003 | 39.062 |  |  |
| rs10861176 | G | A | 0.257 | -0.056 | 0.010 | 1.59E-08 | 0.0002 | 32.075 |  |  |
| rs10873538 | T | G | 0.654 | -0.067 | 0.009 | 3.01E-13 | 0.0004 | 53.407 |  |  |
| rs10876446 | G | C | 0.674 | -0.054 | 0.009 | 1.03E-08 | 0.0003 | 33.004 |  |  |
| rs11027839 | A | C | 0.488 | -0.052 | 0.009 | 2.40E-09 | 0.0003 | 35.866 |  |  |
| rs11136325 | G | A | 0.436 | 0.054 | 0.009 | 3.05E-09 | 0.0003 | 34.948 |  |  |
| rs11165867 | C | T | 0.829 | -0.074 | 0.012 | 1.30E-10 | 0.0003 | 41.030 |  |  |
| rs11191580 | T | C | 0.920 | 0.132 | 0.016 | 1.77E-17 | 0.0006 | 72.199 |  |  |
| rs11210892 | G | A | 0.347 | 0.064 | 0.009 | 2.68E-12 | 0.0004 | 48.694 |  |  |
| rs11223774 | A | G | 0.301 | 0.052 | 0.009 | 2.74E-08 | 0.0002 | 31.191 |  |  |
| rs113264400 | T | C | 0.948 | -0.112 | 0.020 | 2.87E-08 | 0.0002 | 30.905 |  |  |
| rs11534045 | G | A | 0.686 | 0.063 | 0.009 | 1.40E-11 | 0.0003 | 45.594 |  |  |
| rs11587347 | C | G | 0.895 | -0.104 | 0.015 | 1.53E-12 | 0.0004 | 49.952 |  |  |
| rs11664298 | G | A | 0.794 | -0.077 | 0.011 | 8.94E-13 | 0.0004 | 51.360 |  |  |
| rs11693094 | C | T | 0.556 | 0.054 | 0.009 | 4.29E-10 | 0.0003 | 39.103 |  |  |
| rs117178087 | C | T | 0.939 | 0.096 | 0.018 | 4.89E-08 | 0.0002 | 29.663 |  |  |
| rs11941714 | G | A | 0.673 | 0.052 | 0.009 | 3.07E-08 | 0.0002 | 30.780 |  |  |
| rs1198588 | A | T | 0.194 | -0.103 | 0.011 | 1.73E-21 | 0.0007 | 90.246 |  |  |
| rs12129573 | C | A | 0.616 | -0.078 | 0.009 | 2.28E-18 | 0.0006 | 76.414 |  |  |
| rs12138231 | T | A | 0.171 | -0.067 | 0.012 | 7.99E-09 | 0.0003 | 33.355 |  |  |
| rs12151767 | G | A | 0.524 | 0.061 | 0.009 | 1.31E-12 | 0.0004 | 50.484 |  |  |
| rs12285419 | C | A | 0.800 | -0.085 | 0.011 | 1.05E-14 | 0.0005 | 59.577 |  |  |
| rs12293670 | A | G | 0.678 | 0.070 | 0.009 | 1.56E-14 | 0.0004 | 58.715 |  |  |
| rs12303743 | G | C | 0.898 | -0.087 | 0.015 | 1.59E-09 | 0.0003 | 36.414 |  |  |
| rs12489270 | T | C | 0.612 | -0.058 | 0.009 | 7.47E-11 | 0.0003 | 42.330 |  |  |
| rs12652777 | T | C | 0.489 | 0.049 | 0.009 | 1.52E-08 | 0.0002 | 32.199 |  |  |
| rs12712510 | T | C | 0.488 | 0.057 | 0.009 | 5.14E-11 | 0.0003 | 43.531 |  |  |
| rs12771371 | G | A | 0.697 | 0.052 | 0.009 | 1.94E-08 | 0.0002 | 31.750 |  |  |
| rs12833624 | C | T | 0.646 | -0.050 | 0.009 | 2.77E-08 | 0.0002 | 31.111 |  |  |
| rs12877581 | G | C | 0.716 | -0.060 | 0.010 | 1.80E-09 | 0.0003 | 36.245 |  |  |
| rs12883788 | C | T | 0.527 | -0.061 | 0.009 | 1.86E-12 | 0.0004 | 49.648 |  |  |
| rs13016542 | T | C | 0.876 | 0.088 | 0.013 | 8.28E-12 | 0.0004 | 46.858 |  |  |
| rs13107325 | C | T | 0.919 | -0.159 | 0.017 | 2.90E-21 | 0.0007 | 89.238 |  |  |
| rs13195636 | A | C | 0.926 | 0.211 | 0.016 | 6.55E-40 | 0.0013 | 175.278 |  |  |
| rs13233308 | C | T | 0.526 | 0.049 | 0.009 | 1.75E-08 | 0.0002 | 32.073 |  |  |
| rs132582 | C | T | 0.471 | 0.051 | 0.009 | 3.26E-09 | 0.0003 | 35.164 |  |  |
| rs1430894 | C | T | 0.506 | -0.053 | 0.009 | 6.15E-10 | 0.0003 | 38.404 |  |  |
| rs145071536 | T | C | 0.799 | -0.085 | 0.012 | 1.62E-12 | 0.0004 | 50.292 |  |  |
| rs1451488 | A | G | 0.432 | -0.071 | 0.009 | 4.47E-16 | 0.0005 | 66.403 |  |  |
| rs149165 | T | G | 0.569 | 0.048 | 0.009 | 3.01E-08 | 0.0002 | 30.694 |  |  |
| rs1593304 | A | G | 0.193 | -0.064 | 0.011 | 7.45E-09 | 0.0003 | 33.349 |  |  |
| rs1604060 | A | G | 0.103 | -0.077 | 0.014 | 3.24E-08 | 0.0002 | 30.411 |  |  |
| rs1615350 | C | T | 0.273 | 0.074 | 0.010 | 4.92E-14 | 0.0004 | 56.409 |  |  |
| rs167924 | A | G | 0.359 | -0.050 | 0.009 | 2.34E-08 | 0.0002 | 31.111 |  |  |
| rs16851048 | T | C | 0.792 | -0.074 | 0.011 | 4.15E-12 | 0.0004 | 48.474 |  |  |
| rs16867571 | A | G | 0.778 | 0.066 | 0.010 | 2.68E-10 | 0.0003 | 39.913 |  |  |
| rs17016552 | C | G | 0.661 | 0.052 | 0.009 | 1.20E-08 | 0.0002 | 32.278 |  |  |
| rs17194490 | G | T | 0.827 | -0.078 | 0.012 | 1.80E-11 | 0.0003 | 45.446 |  |  |
| rs17731 | G | A | 0.620 | -0.052 | 0.009 | 4.37E-09 | 0.0003 | 34.663 |  |  |
| rs187557 | C | T | 0.163 | 0.067 | 0.012 | 2.03E-08 | 0.0002 | 31.412 |  |  |
| rs1881046 | G | T | 0.670 | 0.051 | 0.009 | 3.39E-08 | 0.0002 | 30.373 |  |  |
| rs1901512 | T | C | 0.318 | 0.058 | 0.009 | 5.72E-10 | 0.0003 | 38.600 |  |  |
| rs1915019 | A | G | 0.265 | 0.057 | 0.010 | 6.57E-09 | 0.0003 | 33.947 |  |  |
| rs2053079 | A | G | 0.754 | -0.060 | 0.010 | 3.01E-09 | 0.0003 | 35.172 |  |  |
| rs2078266 | A | G | 0.178 | 0.070 | 0.013 | 2.94E-08 | 0.0002 | 30.513 |  |  |
| rs215412 | G | A | 0.661 | -0.058 | 0.009 | 2.69E-10 | 0.0003 | 40.209 |  |  |
| rs217336 | C | A | 0.583 | 0.050 | 0.009 | 8.05E-09 | 0.0003 | 33.431 |  |  |
| rs2238057 | T | G | 0.568 | -0.084 | 0.009 | 8.50E-22 | 0.0007 | 92.118 |  |  |
| rs2252074 | T | G | 0.590 | -0.069 | 0.009 | 6.19E-15 | 0.0005 | 60.599 |  |  |
| rs2332700 | C | G | 0.258 | 0.075 | 0.010 | 3.88E-14 | 0.0004 | 57.542 |  |  |
| rs2333321 | A | G | 0.218 | 0.071 | 0.011 | 1.25E-11 | 0.0004 | 45.986 |  |  |
| rs2381411 | T | C | 0.586 | -0.050 | 0.009 | 1.25E-08 | 0.0003 | 32.800 |  |  |
| rs2455415 | C | T | 0.577 | -0.049 | 0.009 | 1.69E-08 | 0.0002 | 31.634 |  |  |
| rs2456020 | C | T | 0.773 | 0.082 | 0.010 | 1.13E-15 | 0.0005 | 63.998 |  |  |
| rs2514218 | C | T | 0.668 | 0.070 | 0.009 | 1.35E-14 | 0.0004 | 58.715 |  |  |
| rs2710323 | T | C | 0.531 | 0.078 | 0.009 | 1.23E-19 | 0.0006 | 83.116 |  |  |
| rs2815731 | C | A | 0.660 | 0.060 | 0.009 | 4.39E-11 | 0.0003 | 43.478 |  |  |
| rs2909457 | G | A | 0.451 | 0.049 | 0.009 | 1.48E-08 | 0.0002 | 31.721 |  |  |
| rs2999392 | C | T | 0.299 | -0.052 | 0.009 | 3.05E-08 | 0.0002 | 30.366 |  |  |
| rs308697 | C | A | 0.574 | 0.050 | 0.009 | 8.83E-09 | 0.0003 | 33.166 |  |  |
| rs35351411 | A | C | 0.439 | -0.064 | 0.009 | 2.21E-13 | 0.0004 | 53.281 |  |  |
| rs35734242 | T | C | 0.562 | -0.051 | 0.009 | 1.37E-08 | 0.0002 | 32.457 |  |  |
| rs3739118 | G | A | 0.719 | 0.057 | 0.010 | 2.36E-09 | 0.0003 | 36.005 |  |  |
| rs3770754 | C | G | 0.645 | 0.053 | 0.009 | 5.35E-09 | 0.0003 | 33.788 |  |  |
| rs3791710 | T | C | 0.804 | 0.060 | 0.011 | 3.02E-08 | 0.0002 | 30.868 |  |  |
| rs3795310 | C | T | 0.543 | 0.051 | 0.009 | 5.75E-09 | 0.0003 | 34.360 |  |  |
| rs3802924 | A | C | 0.805 | 0.074 | 0.011 | 9.58E-12 | 0.0004 | 46.446 |  |  |
| rs3814883 | C | T | 0.548 | 0.067 | 0.009 | 1.58E-14 | 0.0005 | 59.481 |  |  |
| rs3824451 | T | C | 0.838 | -0.066 | 0.012 | 2.54E-08 | 0.0002 | 30.901 |  |  |
| rs4129585 | A | C | 0.456 | 0.075 | 0.009 | 5.11E-18 | 0.0006 | 74.309 |  |  |
| rs4575535 | A | G | 0.281 | -0.056 | 0.010 | 5.77E-09 | 0.0003 | 33.783 |  |  |
| rs4632195 | C | T | 0.471 | -0.047 | 0.009 | 4.59E-08 | 0.0002 | 30.118 |  |  |
| rs4636654 | G | A | 0.608 | 0.048 | 0.009 | 4.89E-08 | 0.0002 | 29.457 |  |  |
| rs4653164 | C | T | 0.323 | -0.051 | 0.009 | 3.08E-08 | 0.0002 | 30.855 |  |  |
| rs4702 | G | A | 0.461 | 0.084 | 0.009 | 2.79E-21 | 0.0007 | 89.726 |  |  |
| rs4766428 | C | T | 0.539 | -0.075 | 0.009 | 3.93E-17 | 0.0005 | 71.021 |  |  |
| rs4779050 | T | G | 0.381 | 0.058 | 0.009 | 7.27E-11 | 0.0003 | 42.462 |  |  |
| rs4812325 | G | A | 0.370 | -0.072 | 0.009 | 8.96E-16 | 0.0005 | 65.272 |  |  |
| rs4921741 | A | G | 0.729 | -0.056 | 0.010 | 1.21E-08 | 0.0002 | 32.652 |  |  |
| rs498591 | A | T | 0.847 | -0.072 | 0.012 | 2.11E-09 | 0.0003 | 35.896 |  |  |
| rs500102 | T | C | 0.413 | 0.052 | 0.009 | 4.87E-09 | 0.0003 | 34.516 |  |  |
| rs505061 | C | A | 0.495 | -0.053 | 0.009 | 5.80E-10 | 0.0003 | 38.694 |  |  |
| rs56205728 | G | A | 0.700 | -0.063 | 0.010 | 1.01E-10 | 0.0003 | 42.188 |  |  |
| rs56335113 | A | G | 0.316 | 0.065 | 0.009 | 6.02E-12 | 0.0004 | 47.377 |  |  |
| rs57433322 | C | G | 0.885 | 0.083 | 0.014 | 1.99E-09 | 0.0003 | 35.741 |  |  |
| rs5751191 | T | C | 0.484 | -0.066 | 0.009 | 3.00E-14 | 0.0004 | 58.176 |  |  |
| rs58120505 | T | C | 0.602 | 0.090 | 0.009 | 2.24E-24 | 0.0008 | 103.676 |  |  |
| rs60135207 | G | T | 0.596 | 0.050 | 0.009 | 1.53E-08 | 0.0002 | 31.768 |  |  |
| rs6125656 | G | A | 0.809 | -0.064 | 0.011 | 6.29E-09 | 0.0003 | 33.761 |  |  |
| rs61857878 | A | T | 0.760 | 0.060 | 0.010 | 4.44E-09 | 0.0003 | 34.715 |  |  |
| rs61937595 | C | T | 0.917 | 0.130 | 0.016 | 1.15E-15 | 0.0005 | 64.493 |  |  |
| rs62018952 | T | C | 0.264 | -0.058 | 0.010 | 1.94E-09 | 0.0003 | 36.251 |  |  |
| rs62183855 | A | C | 0.814 | 0.066 | 0.011 | 2.66E-09 | 0.0003 | 35.458 |  |  |
| rs634940 | G | T | 0.737 | -0.066 | 0.010 | 1.78E-11 | 0.0003 | 44.980 |  |  |
| rs6482437 | A | C | 0.099 | -0.099 | 0.014 | 3.33E-12 | 0.0004 | 48.512 |  |  |
| rs6520064 | A | G | 0.787 | -0.058 | 0.011 | 3.58E-08 | 0.0002 | 30.456 |  |  |
| rs6538539 | G | T | 0.462 | 0.057 | 0.009 | 4.43E-11 | 0.0003 | 43.615 |  |  |
| rs6546857 | A | G | 0.757 | -0.060 | 0.010 | 2.74E-09 | 0.0003 | 35.062 |  |  |
| rs6549963 | T | C | 0.596 | 0.048 | 0.009 | 4.31E-08 | 0.0002 | 30.131 |  |  |
| rs6673880 | A | G | 0.492 | -0.062 | 0.009 | 7.20E-12 | 0.0004 | 46.871 |  |  |
| rs6715366 | G | A | 0.723 | -0.054 | 0.010 | 2.49E-08 | 0.0002 | 31.103 |  |  |
| rs6798742 | A | G | 0.680 | -0.061 | 0.009 | 4.57E-11 | 0.0003 | 43.162 |  |  |
| rs6943762 | T | C | 0.883 | 0.105 | 0.013 | 1.57E-15 | 0.0005 | 63.394 |  |  |
| rs6974218 | A | C | 0.634 | 0.055 | 0.009 | 6.80E-10 | 0.0003 | 38.044 |  |  |
| rs6984242 | G | A | 0.413 | 0.055 | 0.009 | 3.86E-10 | 0.0003 | 39.526 |  |  |
| rs708228 | C | T | 0.661 | -0.053 | 0.009 | 6.56E-09 | 0.0003 | 33.665 |  |  |
| rs7112616 | T | C | 0.515 | 0.052 | 0.009 | 1.52E-09 | 0.0003 | 36.847 |  |  |
| rs713692 | G | A | 0.299 | -0.057 | 0.010 | 2.67E-09 | 0.0003 | 35.499 |  |  |
| rs7251 | C | G | 0.682 | 0.064 | 0.009 | 8.29E-12 | 0.0004 | 46.502 |  |  |
| rs72802868 | G | T | 0.724 | 0.069 | 0.010 | 4.55E-13 | 0.0004 | 51.959 |  |  |
| rs728055 | T | A | 0.658 | 0.067 | 0.009 | 8.85E-14 | 0.0004 | 56.078 |  |  |
| rs72943392 | G | C | 0.707 | -0.053 | 0.010 | 2.39E-08 | 0.0002 | 31.052 |  |  |
| rs72986630 | C | T | 0.926 | -0.112 | 0.018 | 3.59E-10 | 0.0003 | 39.357 |  |  |
| rs73229090 | C | A | 0.897 | 0.103 | 0.014 | 4.34E-13 | 0.0004 | 52.207 |  |  |
| rs73292401 | T | A | 0.799 | -0.068 | 0.011 | 5.48E-10 | 0.0003 | 38.468 |  |  |
| rs7515363 | C | T | 0.389 | 0.054 | 0.009 | 1.84E-09 | 0.0003 | 36.139 |  |  |
| rs7575796 | A | G | 0.917 | 0.096 | 0.017 | 2.07E-08 | 0.0002 | 31.347 |  |  |
| rs7634476 | A | G | 0.397 | -0.058 | 0.009 | 5.46E-11 | 0.0003 | 42.997 |  |  |
| rs7647398 | C | T | 0.811 | 0.077 | 0.011 | 1.07E-12 | 0.0004 | 50.551 |  |  |
| rs76838079 | C | T | 0.849 | -0.078 | 0.014 | 1.53E-08 | 0.0002 | 31.951 |  |  |
| rs778371 | A | G | 0.699 | -0.081 | 0.010 | 1.50E-17 | 0.0006 | 71.987 |  |  |
| rs7798283 | T | G | 0.875 | 0.074 | 0.013 | 3.49E-08 | 0.0002 | 30.499 |  |  |
| rs7830315 | T | C | 0.478 | -0.048 | 0.009 | 3.08E-08 | 0.0002 | 30.899 |  |  |
| rs79210963 | T | C | 0.884 | -0.086 | 0.014 | 4.14E-10 | 0.0003 | 39.041 |  |  |
| rs79445414 | T | C | 0.956 | -0.123 | 0.022 | 2.80E-08 | 0.0002 | 30.898 |  |  |
| rs8055219 | G | A | 0.758 | -0.067 | 0.010 | 5.69E-11 | 0.0003 | 43.355 |  |  |
| rs9304548 | C | A | 0.261 | 0.057 | 0.010 | 1.59E-08 | 0.0002 | 32.151 |  |  |
| rs9318627 | A | C | 0.613 | 0.061 | 0.009 | 4.35E-12 | 0.0004 | 48.363 |  |  |
| rs9461916 | T | C | 0.388 | -0.053 | 0.009 | 1.64E-09 | 0.0003 | 36.679 |  |  |
| rs9636107 | A | G | 0.506 | -0.070 | 0.009 | 5.12E-16 | 0.0005 | 66.057 |  |  |
| rs9687282 | T | G | 0.650 | -0.053 | 0.009 | 7.33E-09 | 0.0003 | 33.410 |  |  |
| rs9876421 | C | T | 0.645 | -0.063 | 0.009 | 9.19E-12 | 0.0004 | 46.156 |  |  |

Abbreviations: EAF, effect allele frequency; GWAS, genome-wide association studies; MR, Mendelian randomisation; SE, standard error; SNP, single nucleotide polymorphism; TDI, Townsend deprivation index.

## Table S11. Summary information on genetic instruments for schizophrenia-TDI MR analysis in TDI GWAS dataset.

| **SNP** | **Effect Allele** | **Alternate Allele** | **EAF** | **Beta** | **SE** | ***p* value** |
| --- | --- | --- | --- | --- | --- | --- |
| rs1000237 | T | A | 0.645 | 0.0071 | 0.002 | 8.10E-04 |
| rs10035564 | A | G | 0.672 | -0.0055 | 0.002 | 1.10E-02 |
| rs10086619 | A | G | 0.832 | -0.0016 | 0.003 | 5.60E-01 |
| rs10108980 | C | T | 0.809 | 0.0008 | 0.003 | 7.60E-01 |
| rs10117 | G | A | 0.604 | 0.0067 | 0.002 | 1.20E-03 |
| rs10861176 | G | A | 0.267 | 0.0025 | 0.002 | 2.70E-01 |
| rs10873538 | T | G | 0.661 | -0.0072 | 0.002 | 7.50E-04 |
| rs10876446 | G | C | 0.683 | -0.0028 | 0.002 | 1.90E-01 |
| rs11027839 | A | C | 0.492 | -0.0024 | 0.002 | 2.40E-01 |
| rs11136325 | G | A | 0.400 | 0.0036 | 0.002 | 8.50E-02 |
| rs11165867 | C | T | 0.839 | -0.0005 | 0.003 | 8.70E-01 |
| rs11191580 | T | C | 0.922 | -0.0147 | 0.004 | 9.30E-05 |
| rs11210892 | G | A | 0.326 | 0.0080 | 0.002 | 2.00E-04 |
| rs11223774 | A | G | 0.292 | -0.0006 | 0.002 | 8.00E-01 |
| rs113264400 | T | C | 0.953 | -0.0011 | 0.005 | 8.20E-01 |
| rs11534045 | G | A | 0.689 | -0.0067 | 0.002 | 2.30E-03 |
| rs11587347 | C | G | 0.902 | 0.0032 | 0.003 | 3.50E-01 |
| rs11664298 | G | A | 0.791 | -0.0110 | 0.003 | 1.10E-05 |
| rs11693094 | C | T | 0.535 | 0.0018 | 0.002 | 3.80E-01 |
| rs117178087 | C | T | 0.935 | 0.0021 | 0.004 | 6.10E-01 |
| rs11941714 | G | A | 0.673 | 0.0058 | 0.002 | 8.40E-03 |
| rs1198588 | A | T | 0.203 | -0.0002 | 0.003 | 9.40E-01 |
| rs12129573 | C | A | 0.651 | -0.0051 | 0.002 | 1.60E-02 |
| rs12138231 | T | A | 0.184 | -0.0044 | 0.003 | 9.50E-02 |
| rs12151767 | G | A | 0.521 | 0.0008 | 0.002 | 6.80E-01 |
| rs12285419 | C | A | 0.805 | 0.0011 | 0.003 | 6.70E-01 |
| rs12293670 | A | G | 0.665 | 0.0014 | 0.002 | 5.20E-01 |
| rs12303743 | G | C | 0.902 | -0.0061 | 0.003 | 7.20E-02 |
| rs12489270 | T | C | 0.630 | -0.0049 | 0.002 | 2.10E-02 |
| rs12652777 | T | C | 0.488 | 0.0070 | 0.002 | 5.60E-04 |
| rs12712510 | T | C | 0.464 | 0.0044 | 0.002 | 3.20E-02 |
| rs12771371 | G | A | 0.684 | 0.0031 | 0.002 | 1.50E-01 |
| rs12833624 | C | T | 0.663 | -0.0027 | 0.002 | 2.00E-01 |
| rs12877581 | G | C | 0.718 | -0.0028 | 0.002 | 2.20E-01 |
| rs12883788 | C | T | 0.540 | -0.0102 | 0.002 | 5.90E-07 |
| rs13016542 | T | C | 0.873 | 0.0007 | 0.003 | 8.10E-01 |
| rs13107325 | C | T | 0.925 | 0.0061 | 0.004 | 1.10E-01 |
| rs13195636 | A | C | 0.885 | 0.0105 | 0.003 | 8.80E-04 |
| rs13233308 | C | T | 0.517 | -0.0011 | 0.002 | 5.80E-01 |
| rs132582 | C | T | 0.441 | 0.0020 | 0.002 | 3.20E-01 |
| rs1430894 | C | T | 0.525 | -0.0052 | 0.002 | 1.10E-02 |
| rs145071536 | T | C | 0.809 | 0.0007 | 0.003 | 7.80E-01 |
| rs1451488 | A | G | 0.443 | -0.0003 | 0.002 | 8.90E-01 |
| rs149165 | T | G | 0.534 | 0.0037 | 0.002 | 6.90E-02 |
| rs1593304 | A | G | 0.207 | 0.0010 | 0.003 | 6.90E-01 |
| rs1604060 | A | G | 0.112 | -0.0141 | 0.003 | 1.20E-05 |
| rs1615350 | C | T | 0.249 | -0.0009 | 0.002 | 7.10E-01 |
| rs167924 | A | G | 0.376 | -0.0030 | 0.002 | 1.50E-01 |
| rs16851048 | T | C | 0.807 | 0.0036 | 0.003 | 1.60E-01 |
| rs16867571 | A | G | 0.759 | -0.0013 | 0.002 | 5.90E-01 |
| rs17016552 | C | G | 0.645 | -0.0009 | 0.002 | 6.80E-01 |
| rs17194490 | G | T | 0.835 | -0.0015 | 0.003 | 5.90E-01 |
| rs17731 | G | A | 0.627 | -0.0020 | 0.002 | 3.50E-01 |
| rs187557 | C | T | 0.151 | 0.0038 | 0.003 | 1.80E-01 |
| rs1881046 | G | T | 0.657 | -0.0006 | 0.002 | 8.00E-01 |
| rs1901512 | T | C | 0.306 | 0.0069 | 0.002 | 1.80E-03 |
| rs1915019 | A | G | 0.240 | 0.0013 | 0.002 | 5.80E-01 |
| rs2053079 | A | G | 0.751 | 0.0026 | 0.002 | 2.70E-01 |
| rs2078266 | A | G | 0.170 | 0.0046 | 0.003 | 1.10E-01 |
| rs215412 | G | A | 0.675 | -0.0026 | 0.002 | 2.30E-01 |
| rs217336 | C | A | 0.563 | 0.0002 | 0.002 | 9.30E-01 |
| rs2238057 | T | G | 0.581 | 0.0019 | 0.002 | 3.50E-01 |
| rs2252074 | T | G | 0.601 | -0.0025 | 0.002 | 2.30E-01 |
| rs2332700 | C | G | 0.248 | -0.0009 | 0.002 | 7.00E-01 |
| rs2333321 | A | G | 0.207 | 0.0017 | 0.003 | 4.90E-01 |
| rs2381411 | T | C | 0.597 | 0.0008 | 0.002 | 6.90E-01 |
| rs2455415 | C | T | 0.570 | -0.0004 | 0.002 | 8.50E-01 |
| rs2456020 | C | T | 0.770 | 0.0033 | 0.002 | 1.70E-01 |
| rs2514218 | C | T | 0.644 | 0.0020 | 0.002 | 3.50E-01 |
| rs2710323 | T | C | 0.528 | -0.0034 | 0.002 | 9.40E-02 |
| rs2815731 | C | A | 0.657 | -0.0009 | 0.002 | 6.70E-01 |
| rs2909457 | G | A | 0.465 | -0.0008 | 0.002 | 7.00E-01 |
| rs2999392 | C | T | 0.303 | -0.0003 | 0.002 | 8.90E-01 |
| rs308697 | C | A | 0.561 | 0.0003 | 0.002 | 9.00E-01 |
| rs35351411 | A | C | 0.445 | 0.0029 | 0.002 | 1.60E-01 |
| rs35734242 | T | C | 0.571 | -0.0013 | 0.002 | 5.40E-01 |
| rs3739118 | G | A | 0.712 | 0.0030 | 0.002 | 1.90E-01 |
| rs3770754 | C | G | 0.641 | 0.0007 | 0.002 | 7.40E-01 |
| rs3791710 | T | C | 0.797 | 0.0025 | 0.003 | 3.20E-01 |
| rs3795310 | C | T | 0.548 | 0.0042 | 0.002 | 3.80E-02 |
| rs3802924 | A | C | 0.802 | 0.0048 | 0.003 | 5.90E-02 |
| rs3814883 | C | T | 0.518 | -0.0022 | 0.002 | 2.90E-01 |
| rs3824451 | T | C | 0.839 | -0.0015 | 0.003 | 5.90E-01 |
| rs4129585 | A | C | 0.439 | 0.0042 | 0.002 | 3.80E-02 |
| rs4575535 | A | G | 0.306 | -0.0010 | 0.002 | 6.40E-01 |
| rs4632195 | C | T | 0.488 | -0.0021 | 0.002 | 3.10E-01 |
| rs4636654 | G | A | 0.604 | 0.0021 | 0.002 | 3.20E-01 |
| rs4653164 | C | T | 0.309 | -0.0030 | 0.002 | 1.70E-01 |
| rs4702 | G | A | 0.444 | 0.0004 | 0.002 | 8.50E-01 |
| rs4766428 | C | T | 0.568 | 0.0035 | 0.002 | 9.00E-02 |
| rs4779050 | T | G | 0.371 | -0.0010 | 0.002 | 6.30E-01 |
| rs4812325 | G | A | 0.391 | -0.0012 | 0.002 | 5.80E-01 |
| rs4921741 | A | G | 0.762 | 0.0029 | 0.002 | 2.30E-01 |
| rs498591 | A | T | 0.858 | -0.0039 | 0.003 | 1.80E-01 |
| rs500102 | T | C | 0.402 | 0.0068 | 0.002 | 1.00E-03 |
| rs505061 | C | A | 0.514 | -0.0029 | 0.002 | 1.50E-01 |
| rs56205728 | G | A | 0.714 | 0.0002 | 0.002 | 9.20E-01 |
| rs56335113 | A | G | 0.309 | -0.0021 | 0.002 | 3.40E-01 |
| rs57433322 | C | G | 0.883 | -0.0049 | 0.003 | 1.20E-01 |
| rs5751191 | T | C | 0.496 | 0.0059 | 0.002 | 3.80E-03 |
| rs58120505 | T | C | 0.566 | 0.0022 | 0.002 | 2.90E-01 |
| rs60135207 | G | T | 0.572 | 0.0036 | 0.002 | 7.90E-02 |
| rs6125656 | G | A | 0.818 | -0.0019 | 0.003 | 4.80E-01 |
| rs61857878 | A | T | 0.743 | 0.0032 | 0.002 | 1.80E-01 |
| rs61937595 | C | T | 0.906 | -0.0109 | 0.004 | 2.10E-03 |
| rs62018952 | T | C | 0.273 | -0.0047 | 0.002 | 3.90E-02 |
| rs62183855 | A | C | 0.795 | -0.0001 | 0.003 | 9.60E-01 |
| rs634940 | G | T | 0.737 | -0.0014 | 0.002 | 5.50E-01 |
| rs6482437 | A | C | 0.111 | 0.0042 | 0.003 | 1.90E-01 |
| rs6520064 | A | G | 0.798 | -0.0049 | 0.003 | 5.20E-02 |
| rs6538539 | G | T | 0.446 | 0.0033 | 0.002 | 1.10E-01 |
| rs6546857 | A | G | 0.762 | -0.0032 | 0.002 | 1.80E-01 |
| rs6549963 | T | C | 0.574 | -0.0013 | 0.002 | 5.30E-01 |
| rs6673880 | A | G | 0.513 | 0.0002 | 0.002 | 9.10E-01 |
| rs6715366 | G | A | 0.724 | 0.0018 | 0.002 | 4.30E-01 |
| rs6798742 | A | G | 0.702 | 0.0007 | 0.002 | 7.70E-01 |
| rs6943762 | T | C | 0.873 | -0.0029 | 0.003 | 3.40E-01 |
| rs6974218 | A | C | 0.616 | 0.0035 | 0.002 | 9.40E-02 |
| rs6984242 | G | A | 0.401 | -0.0019 | 0.002 | 3.70E-01 |
| rs708228 | C | T | 0.672 | -0.0084 | 0.002 | 9.60E-05 |
| rs7112616 | T | C | 0.499 | -0.0005 | 0.002 | 7.90E-01 |
| rs713692 | G | A | 0.315 | -0.0040 | 0.002 | 6.80E-02 |
| rs7251 | C | G | 0.680 | -0.0021 | 0.002 | 3.20E-01 |
| rs72802868 | G | T | 0.702 | 0.0086 | 0.002 | 1.10E-04 |
| rs728055 | T | A | 0.633 | 0.0016 | 0.002 | 4.40E-01 |
| rs72943392 | G | C | 0.718 | -0.0011 | 0.002 | 6.40E-01 |
| rs72986630 | C | T | 0.935 | -0.0008 | 0.004 | 8.50E-01 |
| rs73229090 | C | A | 0.883 | 0.0014 | 0.003 | 6.60E-01 |
| rs73292401 | T | A | 0.808 | -0.0007 | 0.003 | 7.90E-01 |
| rs7515363 | C | T | 0.385 | 0.0018 | 0.002 | 3.90E-01 |
| rs7575796 | A | G | 0.921 | 0.0015 | 0.004 | 7.00E-01 |
| rs7634476 | A | G | 0.398 | 0.0022 | 0.002 | 2.80E-01 |
| rs7647398 | C | T | 0.800 | -0.0035 | 0.003 | 1.70E-01 |
| rs76838079 | C | T | 0.836 | -0.0064 | 0.003 | 1.80E-02 |
| rs778371 | A | G | 0.721 | -0.0005 | 0.002 | 8.20E-01 |
| rs7798283 | T | G | 0.872 | 0.0020 | 0.003 | 5.10E-01 |
| rs7830315 | T | C | 0.502 | 0.0010 | 0.002 | 6.20E-01 |
| rs79210963 | T | C | 0.883 | -0.0053 | 0.003 | 9.30E-02 |
| rs79445414 | T | C | 0.953 | -0.0112 | 0.005 | 2.10E-02 |
| rs8055219 | G | A | 0.775 | -0.0065 | 0.002 | 7.10E-03 |
| rs9304548 | C | A | 0.260 | 0.0005 | 0.002 | 8.30E-01 |
| rs9318627 | A | C | 0.593 | 0.0009 | 0.002 | 6.50E-01 |
| rs9461916 | T | C | 0.410 | -0.0003 | 0.002 | 8.80E-01 |
| rs9636107 | A | G | 0.529 | -0.0088 | 0.002 | 1.70E-05 |
| rs9687282 | T | G | 0.666 | -0.0014 | 0.002 | 5.30E-01 |
| rs9876421 | C | T | 0.658 | -0.0013 | 0.002 | 5.40E-01 |

Abbreviations: EAF, effect allele frequency; GWAS, genome-wide association studies; MR, Mendelian randomisation; SE, standard error; SNP, single nucleotide polymorphism; TDI, Townsend deprivation index.

## Table S12. Summary information on genetic instruments for SES-MDD multivariable MR analysis in GWAS datasets of the exposures.

| **SNP** | **Effect Allele** | **Alternate Allele** | **Educational years** | | | **Household income** | | | **TDI** | | | **Schizophrenia** | | |
| --- | --- | --- | --- | --- | --- | --- | --- | --- | --- | --- | --- | --- | --- | --- |
|  |  |  | **Beta** | **SE** | ***p* value** | **Beta** | **SE** | ***p* value** | **Beta** | **SE** | ***p* value** | **Beta** | **SE** | ***p* value** |
| rs10035564 | A | G | -0.0051 | 0.002 | 1.83E-10 | 0.0049 | 0.003 | 8.20E-02 | -0.0055 | 0.002 | 1.10E-02 | -0.0668 | 0.009 | 4.38E-13 |
| rs10073890 | A | G | 0.0126 | 0.002 | 1.00E-10 | 0.0048 | 0.003 | 1.10E-01 | -0.0047 | 0.002 | 4.30E-02 | 0.0116 | 0.010 | 2.37E-01 |
| rs10086619 | A | G | 0.0016 | 0.002 | 7.44E-03 | -0.0025 | 0.004 | 4.80E-01 | -0.0016 | 0.003 | 5.60E-01 | -0.0722 | 0.012 | 4.97E-10 |
| rs10108980 | C | T | 0.0009 | 0.002 | 2.31E-02 | 0.0004 | 0.003 | 8.90E-01 | 0.0008 | 0.003 | 7.60E-01 | -0.0628 | 0.011 | 2.73E-09 |
| rs10189857 | A | G | 0.0173 | 0.002 | 1.00E-10 | 0.0127 | 0.003 | 2.10E-06 | -0.0014 | 0.002 | 5.00E-01 | 0.0409 | 0.009 | 2.53E-06 |
| rs10215082 | A | G | -0.0130 | 0.002 | 1.00E-10 | -0.0090 | 0.003 | 7.20E-04 | 0.0056 | 0.002 | 5.50E-03 | 0.0104 | 0.009 | 2.30E-01 |
| rs10240905 | T | C | -0.0117 | 0.002 | 1.00E-10 | -0.0019 | 0.003 | 5.00E-01 | 0.0059 | 0.002 | 5.50E-03 | -0.0030 | 0.009 | 7.34E-01 |
| rs10429582 | T | C | -0.0240 | 0.002 | 1.00E-10 | -0.0270 | 0.003 | 7.60E-24 | 0.0021 | 0.002 | 3.00E-01 | 0.0185 | 0.009 | 3.58E-02 |
| rs10456918 | A | C | -0.0149 | 0.002 | 1.00E-10 | -0.0164 | 0.003 | 1.80E-06 | 0.0033 | 0.003 | 2.10E-01 | -0.0317 | 0.012 | 6.00E-03 |
| rs10460095 | G | A | 0.0107 | 0.002 | 1.00E-10 | 0.0051 | 0.003 | 5.60E-02 | -0.0049 | 0.002 | 1.80E-02 | -0.0220 | 0.009 | 1.18E-02 |
| rs1051474 | T | C | -0.0130 | 0.002 | 1.00E-10 | -0.0073 | 0.003 | 1.30E-02 | 0.0048 | 0.002 | 3.40E-02 | 0.0253 | 0.010 | 8.30E-03 |
| rs10765775 | G | A | -0.0149 | 0.002 | 1.00E-10 | -0.0090 | 0.003 | 1.00E-03 | -0.0004 | 0.002 | 8.30E-01 | -0.0107 | 0.009 | 2.31E-01 |
| rs10798418 | C | T | 0.0096 | 0.002 | 1.00E-10 | 0.0011 | 0.003 | 6.80E-01 | -0.0017 | 0.002 | 4.20E-01 | 0.0239 | 0.009 | 6.32E-03 |
| rs10861176 | G | A | -0.0050 | 0.002 | 2.95E-10 | -0.0040 | 0.003 | 1.80E-01 | 0.0025 | 0.002 | 2.70E-01 | -0.0555 | 0.010 | 1.59E-08 |
| rs10887801 | G | T | -0.0109 | 0.002 | 1.00E-10 | -0.0036 | 0.003 | 1.80E-01 | 0.0013 | 0.002 | 5.20E-01 | 0.0005 | 0.009 | 9.54E-01 |
| rs10940921 | T | G | 0.0109 | 0.002 | 1.00E-10 | 0.0106 | 0.003 | 8.70E-05 | -0.0053 | 0.002 | 1.00E-02 | -0.0049 | 0.009 | 5.76E-01 |
| rs10994777 | G | A | -0.0146 | 0.002 | 1.00E-10 | -0.0089 | 0.004 | 1.40E-02 | 0.0118 | 0.003 | 2.00E-05 | -0.0044 | 0.012 | 7.12E-01 |
| rs11023749 | G | A | -0.0113 | 0.002 | 1.00E-10 | -0.0041 | 0.003 | 1.50E-01 | -0.0005 | 0.002 | 8.30E-01 | 0.0002 | 0.009 | 9.86E-01 |
| rs1105307 | G | A | 0.0117 | 0.002 | 1.00E-10 | 0.0087 | 0.003 | 4.10E-03 | 0.0021 | 0.002 | 3.70E-01 | -0.0313 | 0.010 | 1.33E-03 |
| rs11081529 | T | C | 0.0131 | 0.002 | 1.00E-10 | 0.0084 | 0.003 | 4.00E-03 | -0.0007 | 0.002 | 7.50E-01 | -0.0100 | 0.010 | 2.99E-01 |
| rs11136325 | G | A | 0.0018 | 0.002 | 6.36E-04 | 0.0011 | 0.003 | 6.90E-01 | 0.0036 | 0.002 | 8.50E-02 | 0.0538 | 0.009 | 3.05E-09 |
| rs111821073 | C | T | -0.0139 | 0.002 | 1.00E-10 | -0.0171 | 0.004 | 3.20E-06 | 0.0057 | 0.003 | 4.30E-02 | 0.0244 | 0.012 | 4.45E-02 |
| rs11191580 | T | C | 0.0022 | 0.003 | 7.31E-03 | -0.0070 | 0.005 | 1.50E-01 | -0.0147 | 0.004 | 9.30E-05 | 0.1317 | 0.016 | 1.77E-17 |
| rs11210892 | G | A | -0.0162 | 0.002 | 1.00E-10 | -0.0157 | 0.003 | 2.50E-08 | 0.0080 | 0.002 | 2.00E-04 | 0.0635 | 0.009 | 2.68E-12 |
| rs112687095 | G | A | -0.0133 | 0.002 | 1.00E-10 | -0.0048 | 0.004 | 1.70E-01 | 0.0012 | 0.003 | 6.50E-01 | -0.0088 | 0.012 | 4.79E-01 |
| rs113615161 | C | T | 0.0147 | 0.003 | 1.00E-10 | 0.0176 | 0.004 | 6.00E-06 | -0.0090 | 0.003 | 2.50E-03 | 0.0252 | 0.013 | 5.09E-02 |
| rs1143770 | C | T | -0.0114 | 0.002 | 1.00E-10 | -0.0078 | 0.003 | 3.60E-03 | 0.0007 | 0.002 | 7.50E-01 | 0.0079 | 0.009 | 3.71E-01 |
| rs11534045 | G | A | -0.0016 | 0.002 | 3.04E-03 | -0.0015 | 0.003 | 6.00E-01 | -0.0067 | 0.002 | 2.30E-03 | 0.0628 | 0.009 | 1.40E-11 |
| rs115454970 | G | T | 0.0119 | 0.002 | 1.00E-10 | 0.0081 | 0.003 | 8.20E-03 | -0.0024 | 0.002 | 3.00E-01 | -0.0091 | 0.010 | 3.64E-01 |
| rs11588857 | G | A | -0.0221 | 0.002 | 1.00E-10 | -0.0213 | 0.003 | 4.90E-11 | 0.0071 | 0.002 | 4.20E-03 | -0.0381 | 0.010 | 2.62E-04 |
| rs11601122 | A | G | 0.0195 | 0.002 | 1.00E-10 | 0.0129 | 0.004 | 3.60E-04 | -0.0012 | 0.003 | 6.80E-01 | 0.0003 | 0.012 | 9.77E-01 |
| rs11620355 | G | A | -0.0176 | 0.003 | 1.00E-10 | -0.0182 | 0.005 | 1.10E-04 | 0.0102 | 0.004 | 5.00E-03 | 0.0321 | 0.015 | 3.58E-02 |
| rs11627087 | A | G | 0.0179 | 0.003 | 1.00E-10 | 0.0189 | 0.005 | 2.40E-04 | -0.0077 | 0.004 | 5.20E-02 | -0.0196 | 0.017 | 2.37E-01 |
| rs11635092 | G | A | 0.0123 | 0.002 | 1.00E-10 | 0.0015 | 0.003 | 6.00E-01 | 0.0038 | 0.002 | 7.60E-02 | -0.0031 | 0.009 | 7.42E-01 |
| rs11657342 | G | A | -0.0140 | 0.002 | 1.00E-10 | -0.0077 | 0.003 | 4.90E-03 | 0.0012 | 0.002 | 5.80E-01 | 0.0211 | 0.010 | 3.55E-02 |
| rs11664298 | G | A | 0.0124 | 0.002 | 1.00E-10 | 0.0098 | 0.003 | 2.60E-03 | -0.0110 | 0.003 | 1.10E-05 | -0.0774 | 0.011 | 8.94E-13 |
| rs11678980 | G | A | 0.0174 | 0.002 | 1.00E-10 | 0.0085 | 0.003 | 2.00E-03 | -0.0028 | 0.002 | 1.90E-01 | -0.0320 | 0.009 | 4.37E-04 |
| rs11732657 | G | A | 0.0127 | 0.002 | 1.00E-10 | 0.0131 | 0.003 | 1.50E-05 | 0.0011 | 0.002 | 6.30E-01 | 0.0095 | 0.010 | 3.40E-01 |
| rs11752914 | T | C | 0.0121 | 0.002 | 1.00E-10 | 0.0127 | 0.003 | 1.40E-04 | -0.0064 | 0.003 | 1.30E-02 | 0.0369 | 0.011 | 7.64E-04 |
| rs11772580 | G | T | 0.0120 | 0.002 | 1.00E-10 | 0.0070 | 0.003 | 2.50E-02 | -0.0048 | 0.002 | 4.70E-02 | 0.0132 | 0.010 | 2.02E-01 |
| rs11855821 | G | A | -0.0151 | 0.002 | 1.00E-10 | -0.0138 | 0.003 | 3.80E-06 | 0.0126 | 0.002 | 3.90E-08 | 0.0054 | 0.010 | 5.84E-01 |
| rs11877758 | T | G | 0.0195 | 0.002 | 1.00E-10 | 0.0204 | 0.003 | 1.20E-12 | -0.0068 | 0.002 | 1.90E-03 | -0.0165 | 0.009 | 7.69E-02 |
| rs11941714 | G | A | -0.0010 | 0.002 | 1.66E-02 | -0.0033 | 0.003 | 2.50E-01 | 0.0058 | 0.002 | 8.40E-03 | 0.0516 | 0.009 | 3.07E-08 |
| rs12028010 | T | C | 0.0170 | 0.002 | 1.00E-10 | 0.0039 | 0.003 | 2.10E-01 | 0.0072 | 0.002 | 2.80E-03 | -0.0080 | 0.010 | 4.37E-01 |
| rs12129573 | C | A | 0.0028 | 0.002 | 1.84E-06 | 0.0038 | 0.003 | 1.70E-01 | -0.0051 | 0.002 | 1.60E-02 | -0.0778 | 0.009 | 2.28E-18 |
| rs12151767 | G | A | 0.0022 | 0.002 | 5.48E-05 | 0.0059 | 0.003 | 2.60E-02 | 0.0008 | 0.002 | 6.80E-01 | 0.0611 | 0.009 | 1.31E-12 |
| rs12203592 | C | T | -0.0019 | 0.002 | 3.47E-03 | 0.0168 | 0.003 | 9.30E-08 | -0.0170 | 0.002 | 1.70E-12 | 0.0059 | 0.013 | 6.59E-01 |
| rs12285419 | C | A | -0.0025 | 0.002 | 2.13E-04 | -0.0006 | 0.003 | 8.60E-01 | 0.0011 | 0.003 | 6.70E-01 | -0.0849 | 0.011 | 1.05E-14 |
| rs12293670 | A | G | 0.0046 | 0.002 | 3.84E-10 | 0.0019 | 0.003 | 5.10E-01 | 0.0014 | 0.002 | 5.20E-01 | 0.0705 | 0.009 | 1.56E-14 |
| rs12375949 | T | C | -0.0145 | 0.002 | 1.00E-10 | -0.0121 | 0.003 | 5.10E-06 | 0.0038 | 0.002 | 6.20E-02 | 0.0017 | 0.009 | 8.46E-01 |
| rs12468040 | T | G | 0.0143 | 0.002 | 1.00E-10 | 0.0121 | 0.003 | 8.90E-06 | -0.0037 | 0.002 | 8.00E-02 | -0.0120 | 0.009 | 1.76E-01 |
| rs12489270 | T | C | 0.0115 | 0.002 | 1.00E-10 | 0.0085 | 0.003 | 2.10E-03 | -0.0049 | 0.002 | 2.10E-02 | -0.0579 | 0.009 | 7.47E-11 |
| rs12574281 | A | C | -0.0108 | 0.002 | 1.00E-10 | -0.0066 | 0.003 | 1.50E-02 | 0.0025 | 0.002 | 2.20E-01 | -0.0047 | 0.009 | 6.03E-01 |
| rs12652777 | T | C | 0.0004 | 0.002 | 3.75E-02 | -0.0081 | 0.003 | 2.30E-03 | 0.0070 | 0.002 | 5.60E-04 | 0.0488 | 0.009 | 1.52E-08 |
| rs12682775 | T | C | -0.0119 | 0.002 | 1.00E-10 | -0.0084 | 0.003 | 8.40E-03 | 0.0005 | 0.002 | 8.40E-01 | 0.0025 | 0.010 | 8.09E-01 |
| rs12712510 | T | C | -0.0011 | 0.002 | 9.11E-03 | -0.0033 | 0.003 | 2.20E-01 | 0.0044 | 0.002 | 3.20E-02 | 0.0574 | 0.009 | 5.14E-11 |
| rs12771371 | G | A | -0.0011 | 0.002 | 1.29E-02 | 0.0014 | 0.003 | 6.20E-01 | 0.0031 | 0.002 | 1.50E-01 | 0.0524 | 0.009 | 1.94E-08 |
| rs12833624 | C | T | -0.0021 | 0.002 | 2.53E-04 | 0.0042 | 0.003 | 1.30E-01 | -0.0027 | 0.002 | 2.00E-01 | -0.0502 | 0.009 | 2.77E-08 |
| rs12883788 | C | T | 0.0052 | 0.002 | 1.33E-10 | 0.0189 | 0.003 | 1.40E-12 | -0.0102 | 0.002 | 5.90E-07 | -0.0613 | 0.009 | 1.86E-12 |
| rs12940014 | T | C | -0.0094 | 0.002 | 1.00E-10 | -0.0033 | 0.003 | 2.20E-01 | -0.0003 | 0.002 | 8.80E-01 | 0.0240 | 0.009 | 5.29E-03 |
| rs13010566 | A | C | -0.0106 | 0.002 | 1.00E-10 | -0.0075 | 0.003 | 4.50E-03 | 0.0022 | 0.002 | 2.80E-01 | -0.0014 | 0.009 | 8.71E-01 |
| rs13016542 | T | C | 0.0071 | 0.003 | 1.79E-10 | -0.0030 | 0.004 | 4.50E-01 | 0.0007 | 0.003 | 8.10E-01 | 0.0883 | 0.013 | 8.28E-12 |
| rs13029509 | G | A | 0.0105 | 0.002 | 1.00E-10 | 0.0124 | 0.003 | 2.80E-06 | -0.0064 | 0.002 | 1.70E-03 | -0.0268 | 0.009 | 1.86E-03 |
| rs13107325 | C | T | 0.0188 | 0.003 | 1.00E-10 | 0.0251 | 0.005 | 6.30E-07 | 0.0061 | 0.004 | 1.10E-01 | -0.1587 | 0.017 | 2.90E-21 |
| rs13141210 | C | T | -0.0136 | 0.002 | 1.00E-10 | -0.0102 | 0.003 | 1.30E-04 | -0.0001 | 0.002 | 9.80E-01 | 0.0145 | 0.009 | 9.46E-02 |
| rs13145650 | C | T | 0.0192 | 0.003 | 1.00E-10 | 0.0115 | 0.005 | 1.50E-02 | -0.0100 | 0.004 | 5.80E-03 | 0.0480 | 0.016 | 2.23E-03 |
| rs13195636 | A | C | -0.0026 | 0.003 | 1.48E-03 | -0.0060 | 0.004 | 1.50E-01 | 0.0105 | 0.003 | 8.80E-04 | 0.2105 | 0.016 | 6.55E-40 |
| rs132582 | C | T | -0.0097 | 0.002 | 1.00E-10 | -0.0083 | 0.003 | 2.00E-03 | 0.0020 | 0.002 | 3.20E-01 | 0.0510 | 0.009 | 3.26E-09 |
| rs1334297 | G | A | -0.0245 | 0.002 | 1.00E-10 | -0.0120 | 0.003 | 6.50E-05 | 0.0025 | 0.002 | 2.80E-01 | 0.0023 | 0.010 | 8.15E-01 |
| rs13422673 | C | T | 0.0120 | 0.002 | 1.00E-10 | 0.0058 | 0.003 | 3.00E-02 | 0.0018 | 0.002 | 3.70E-01 | -0.0019 | 0.009 | 8.24E-01 |
| rs1381247 | T | C | 0.0101 | 0.002 | 1.00E-10 | 0.0116 | 0.003 | 3.50E-05 | -0.0001 | 0.002 | 9.60E-01 | -0.0137 | 0.009 | 1.46E-01 |
| rs1391438 | T | C | 0.0167 | 0.002 | 1.00E-10 | 0.0110 | 0.003 | 1.10E-04 | 0.0051 | 0.002 | 2.00E-02 | 0.0186 | 0.009 | 4.16E-02 |
| rs1430894 | C | T | -0.0008 | 0.002 | 1.84E-02 | 0.0072 | 0.003 | 6.40E-03 | -0.0052 | 0.002 | 1.10E-02 | -0.0533 | 0.009 | 6.15E-10 |
| rs145071536 | T | C | -0.0057 | 0.002 | 4.48E-10 | 0.0039 | 0.003 | 2.60E-01 | 0.0007 | 0.003 | 7.80E-01 | -0.0851 | 0.012 | 1.62E-12 |
| rs1450782 | T | G | 0.0095 | 0.002 | 1.00E-10 | 0.0048 | 0.003 | 7.00E-02 | -0.0018 | 0.002 | 3.70E-01 | -0.0087 | 0.009 | 3.23E-01 |
| rs1451488 | A | G | -0.0020 | 0.002 | 1.51E-04 | -0.0009 | 0.003 | 7.40E-01 | -0.0003 | 0.002 | 8.90E-01 | -0.0709 | 0.009 | 4.47E-16 |
| rs152603 | A | G | -0.0102 | 0.002 | 1.00E-10 | -0.0108 | 0.003 | 7.60E-05 | -0.0004 | 0.002 | 8.30E-01 | -0.0310 | 0.009 | 5.69E-04 |
| rs1558727 | C | T | 0.0107 | 0.002 | 1.00E-10 | 0.0110 | 0.003 | 3.60E-05 | -0.0003 | 0.002 | 8.80E-01 | -0.0119 | 0.009 | 1.70E-01 |
| rs1584469 | C | T | 0.0130 | 0.002 | 1.00E-10 | 0.0082 | 0.003 | 4.60E-03 | -0.0004 | 0.002 | 8.70E-01 | -0.0031 | 0.010 | 7.41E-01 |
| rs1595973 | C | T | 0.0100 | 0.002 | 1.00E-10 | 0.0066 | 0.003 | 1.40E-02 | -0.0020 | 0.002 | 3.30E-01 | 0.0051 | 0.009 | 5.60E-01 |
| rs1615350 | C | T | 0.0213 | 0.002 | 1.00E-10 | 0.0081 | 0.003 | 7.70E-03 | -0.0009 | 0.002 | 7.10E-01 | 0.0736 | 0.010 | 4.92E-14 |
| rs1618725 | C | T | -0.0148 | 0.002 | 1.00E-10 | -0.0037 | 0.003 | 1.60E-01 | -0.0037 | 0.002 | 7.10E-02 | 0.0028 | 0.009 | 7.47E-01 |
| rs1620977 | A | G | 0.0205 | 0.002 | 1.00E-10 | 0.0090 | 0.003 | 2.50E-03 | -0.0001 | 0.002 | 9.80E-01 | 0.0163 | 0.010 | 1.04E-01 |
| rs16846463 | A | G | 0.0226 | 0.003 | 1.00E-10 | 0.0169 | 0.004 | 1.30E-04 | -0.0024 | 0.003 | 4.70E-01 | -0.0065 | 0.014 | 6.54E-01 |
| rs16851048 | T | C | -0.0028 | 0.002 | 5.31E-05 | 0.0016 | 0.003 | 6.30E-01 | 0.0036 | 0.003 | 1.60E-01 | -0.0745 | 0.011 | 4.15E-12 |
| rs16854920 | T | C | -0.0101 | 0.002 | 1.00E-10 | -0.0030 | 0.003 | 2.80E-01 | 0.0039 | 0.002 | 6.80E-02 | -0.0263 | 0.009 | 4.47E-03 |
| rs16867571 | A | G | 0.0098 | 0.002 | 1.00E-10 | 0.0044 | 0.003 | 1.60E-01 | -0.0013 | 0.002 | 5.90E-01 | 0.0657 | 0.010 | 2.68E-10 |
| rs16995054 | C | T | 0.0139 | 0.002 | 1.00E-10 | 0.0065 | 0.003 | 4.80E-02 | -0.0003 | 0.003 | 8.90E-01 | -0.0061 | 0.011 | 5.67E-01 |
| rs17194490 | G | T | -0.0093 | 0.002 | 1.01E-10 | -0.0001 | 0.004 | 9.80E-01 | -0.0015 | 0.003 | 5.90E-01 | -0.0782 | 0.012 | 1.80E-11 |
| rs17425572 | A | G | 0.0122 | 0.002 | 1.00E-10 | 0.0098 | 0.003 | 2.40E-04 | -0.0039 | 0.002 | 5.30E-02 | 0.0019 | 0.009 | 8.27E-01 |
| rs17489649 | A | G | 0.0139 | 0.002 | 1.00E-10 | 0.0017 | 0.003 | 5.50E-01 | 0.0042 | 0.002 | 4.90E-02 | 0.0169 | 0.009 | 6.93E-02 |
| rs17551064 | A | G | 0.0149 | 0.002 | 1.00E-10 | 0.0119 | 0.004 | 8.90E-04 | -0.0034 | 0.003 | 2.10E-01 | 0.0187 | 0.012 | 1.09E-01 |
| rs17563464 | C | A | 0.0148 | 0.002 | 1.00E-10 | 0.0078 | 0.003 | 1.60E-02 | -0.0039 | 0.002 | 1.10E-01 | 0.0053 | 0.012 | 6.46E-01 |
| rs17565975 | G | A | 0.0114 | 0.002 | 1.00E-10 | 0.0053 | 0.003 | 4.60E-02 | -0.0067 | 0.002 | 1.20E-03 | -0.0042 | 0.009 | 6.31E-01 |
| rs17598675 | T | C | -0.0120 | 0.002 | 1.00E-10 | -0.0044 | 0.003 | 9.70E-02 | 0.0016 | 0.002 | 4.40E-01 | -0.0050 | 0.009 | 5.60E-01 |
| rs176218 | G | T | -0.0188 | 0.002 | 1.00E-10 | -0.0158 | 0.003 | 2.80E-06 | -0.0031 | 0.003 | 2.30E-01 | 0.0236 | 0.011 | 3.00E-02 |
| rs17731 | G | A | -0.0003 | 0.002 | 4.43E-02 | 0.0041 | 0.003 | 1.40E-01 | -0.0020 | 0.002 | 3.50E-01 | -0.0524 | 0.009 | 4.37E-09 |
| rs1866823 | G | A | -0.0101 | 0.002 | 1.00E-10 | -0.0121 | 0.003 | 6.90E-06 | 0.0006 | 0.002 | 7.70E-01 | 0.0214 | 0.009 | 1.41E-02 |
| rs1915019 | A | G | 0.0035 | 0.002 | 1.52E-07 | 0.0003 | 0.003 | 9.20E-01 | 0.0013 | 0.002 | 5.80E-01 | 0.0571 | 0.010 | 6.57E-09 |
| rs192436652 | C | T | 0.0350 | 0.005 | 1.00E-10 | 0.0114 | 0.008 | 1.60E-01 | -0.0112 | 0.006 | 7.20E-02 | 0.0749 | 0.028 | 8.17E-03 |
| rs1925576 | A | G | -0.0100 | 0.002 | 1.00E-10 | -0.0065 | 0.003 | 1.70E-02 | 0.0019 | 0.002 | 3.50E-01 | -0.0119 | 0.009 | 1.69E-01 |
| rs1964927 | A | G | 0.0142 | 0.002 | 1.00E-10 | 0.0056 | 0.003 | 4.30E-02 | -0.0043 | 0.002 | 4.30E-02 | 0.0059 | 0.009 | 5.12E-01 |
| rs215412 | G | A | -0.0031 | 0.002 | 3.28E-07 | 0.0092 | 0.003 | 1.20E-03 | -0.0026 | 0.002 | 2.30E-01 | -0.0577 | 0.009 | 2.69E-10 |
| rs217336 | C | A | -0.0001 | 0.002 | 5.22E-02 | -0.0045 | 0.003 | 8.90E-02 | 0.0002 | 0.002 | 9.30E-01 | 0.0503 | 0.009 | 8.05E-09 |
| rs2182505 | T | C | 0.0109 | 0.002 | 1.00E-10 | 0.0051 | 0.003 | 8.50E-02 | -0.0049 | 0.002 | 3.40E-02 | 0.0019 | 0.010 | 8.41E-01 |
| rs2238057 | T | G | 0.0014 | 0.002 | 4.35E-03 | -0.0022 | 0.003 | 4.10E-01 | 0.0019 | 0.002 | 3.50E-01 | -0.0835 | 0.009 | 8.50E-22 |
| rs2252074 | T | G | 0.0050 | 0.002 | 1.63E-10 | 0.0064 | 0.003 | 1.70E-02 | -0.0025 | 0.002 | 2.30E-01 | -0.0685 | 0.009 | 6.19E-15 |
| rs225291 | A | G | 0.0121 | 0.002 | 1.00E-10 | 0.0037 | 0.003 | 2.70E-01 | -0.0009 | 0.003 | 7.30E-01 | 0.0154 | 0.011 | 1.48E-01 |
| rs2283076 | A | G | 0.0114 | 0.002 | 1.00E-10 | 0.0172 | 0.003 | 5.50E-08 | -0.0033 | 0.002 | 1.70E-01 | -0.0238 | 0.011 | 2.39E-02 |
| rs2287838 | G | A | 0.0115 | 0.002 | 1.00E-10 | 0.0067 | 0.003 | 1.20E-02 | 0.0016 | 0.002 | 4.30E-01 | -0.0183 | 0.009 | 3.54E-02 |
| rs2302761 | C | T | -0.0135 | 0.002 | 1.00E-10 | -0.0090 | 0.003 | 5.50E-03 | 0.0039 | 0.002 | 1.10E-01 | -0.0063 | 0.011 | 5.51E-01 |
| rs2332719 | A | G | 0.0102 | 0.002 | 1.00E-10 | 0.0183 | 0.003 | 5.70E-10 | -0.0085 | 0.002 | 1.60E-04 | -0.0100 | 0.010 | 2.97E-01 |
| rs2333321 | A | G | -0.0081 | 0.002 | 1.01E-10 | -0.0050 | 0.003 | 1.30E-01 | 0.0017 | 0.003 | 4.90E-01 | 0.0712 | 0.011 | 1.25E-11 |
| rs2347526 | T | C | -0.0140 | 0.002 | 1.00E-10 | -0.0114 | 0.003 | 4.40E-05 | 0.0003 | 0.002 | 9.00E-01 | -0.0163 | 0.009 | 7.41E-02 |
| rs2381411 | T | C | 0.0024 | 0.002 | 1.88E-05 | 0.0014 | 0.003 | 6.20E-01 | 0.0008 | 0.002 | 6.90E-01 | -0.0504 | 0.009 | 1.25E-08 |
| rs242093 | G | A | 0.0103 | 0.002 | 1.00E-10 | 0.0088 | 0.003 | 1.10E-03 | -0.0061 | 0.002 | 3.10E-03 | -0.0045 | 0.009 | 6.10E-01 |
| rs2422859 | T | G | -0.0073 | 0.002 | 1.00E-10 | -0.0164 | 0.003 | 6.20E-10 | 0.0013 | 0.002 | 5.30E-01 | 0.0052 | 0.009 | 5.59E-01 |
| rs2441111 | G | A | -0.0109 | 0.002 | 1.00E-10 | -0.0022 | 0.003 | 4.00E-01 | -0.0015 | 0.002 | 4.80E-01 | -0.0295 | 0.009 | 6.65E-04 |
| rs2447535 | A | G | -0.0118 | 0.002 | 1.00E-10 | -0.0054 | 0.003 | 6.40E-02 | 0.0017 | 0.002 | 4.50E-01 | 0.0191 | 0.010 | 4.46E-02 |
| rs2456020 | C | T | -0.0042 | 0.002 | 5.04E-09 | -0.0124 | 0.003 | 7.50E-05 | 0.0033 | 0.002 | 1.70E-01 | 0.0816 | 0.010 | 1.13E-15 |
| rs2514218 | C | T | -0.0060 | 0.002 | 1.09E-10 | -0.0022 | 0.003 | 4.20E-01 | 0.0020 | 0.002 | 3.50E-01 | 0.0705 | 0.009 | 1.35E-14 |
| rs2554835 | G | A | -0.0097 | 0.002 | 1.00E-10 | -0.0044 | 0.003 | 1.10E-01 | 0.0019 | 0.002 | 3.70E-01 | 0.0154 | 0.009 | 9.26E-02 |
| rs2570497 | C | T | 0.0123 | 0.002 | 1.00E-10 | 0.0057 | 0.003 | 3.90E-02 | -0.0011 | 0.002 | 6.00E-01 | 0.0086 | 0.009 | 3.33E-01 |
| rs2710323 | T | C | 0.0004 | 0.002 | 3.77E-02 | -0.0041 | 0.003 | 1.30E-01 | -0.0034 | 0.002 | 9.40E-02 | 0.0784 | 0.009 | 1.23E-19 |
| rs2725370 | T | C | -0.0154 | 0.002 | 1.00E-10 | -0.0135 | 0.003 | 3.00E-06 | 0.0058 | 0.002 | 9.30E-03 | 0.0031 | 0.009 | 7.43E-01 |
| rs277828 | C | A | 0.0109 | 0.002 | 1.00E-10 | 0.0021 | 0.003 | 4.90E-01 | -0.0010 | 0.002 | 6.50E-01 | -0.0319 | 0.010 | 1.58E-03 |
| rs2787101 | C | T | -0.0097 | 0.002 | 1.00E-10 | -0.0094 | 0.003 | 5.20E-04 | 0.0042 | 0.002 | 4.40E-02 | 0.0415 | 0.009 | 2.61E-06 |
| rs2815731 | C | A | 0.0023 | 0.002 | 5.13E-05 | 0.0024 | 0.003 | 4.00E-01 | -0.0009 | 0.002 | 6.70E-01 | 0.0600 | 0.009 | 4.39E-11 |
| rs2820314 | A | C | 0.0110 | 0.002 | 1.00E-10 | 0.0166 | 0.003 | 2.90E-09 | -0.0057 | 0.002 | 7.50E-03 | -0.0159 | 0.009 | 8.45E-02 |
| rs28513670 | A | G | -0.0148 | 0.002 | 1.00E-10 | -0.0077 | 0.004 | 2.80E-02 | 0.0035 | 0.003 | 2.00E-01 | -0.0249 | 0.011 | 2.64E-02 |
| rs2885198 | A | G | 0.0103 | 0.002 | 1.00E-10 | 0.0073 | 0.003 | 6.00E-03 | -0.0040 | 0.002 | 5.00E-02 | 0.0064 | 0.009 | 4.69E-01 |
| rs2998315 | A | G | -0.0127 | 0.002 | 1.00E-10 | -0.0053 | 0.003 | 4.40E-02 | -0.0026 | 0.002 | 2.00E-01 | -0.0045 | 0.009 | 6.04E-01 |
| rs301800 | T | C | 0.0152 | 0.002 | 1.00E-10 | 0.0135 | 0.003 | 9.80E-05 | -0.0044 | 0.003 | 9.80E-02 | -0.0299 | 0.011 | 7.68E-03 |
| rs3026996 | A | C | 0.0154 | 0.002 | 1.00E-10 | 0.0045 | 0.003 | 1.50E-01 | -0.0017 | 0.002 | 4.60E-01 | 0.0261 | 0.010 | 1.13E-02 |
| rs31940 | G | A | -0.0155 | 0.002 | 1.00E-10 | -0.0079 | 0.004 | 3.80E-02 | 0.0003 | 0.003 | 9.30E-01 | 0.0104 | 0.013 | 4.08E-01 |
| rs32940 | T | C | -0.0090 | 0.002 | 1.00E-10 | -0.0211 | 0.003 | 2.90E-13 | -0.0007 | 0.002 | 7.70E-01 | 0.0019 | 0.010 | 8.45E-01 |
| rs337637 | G | A | -0.0112 | 0.002 | 1.00E-10 | -0.0079 | 0.003 | 3.90E-03 | -0.0006 | 0.002 | 7.70E-01 | 0.0007 | 0.009 | 9.42E-01 |
| rs34316 | A | C | 0.0202 | 0.002 | 1.00E-10 | 0.0076 | 0.003 | 4.50E-03 | -0.0037 | 0.002 | 7.60E-02 | -0.0215 | 0.009 | 1.37E-02 |
| rs34473884 | G | A | -0.0147 | 0.002 | 1.00E-10 | -0.0172 | 0.003 | 1.70E-08 | 0.0063 | 0.002 | 7.30E-03 | -0.0257 | 0.010 | 1.22E-02 |
| rs34485537 | C | T | -0.0108 | 0.002 | 1.00E-10 | -0.0064 | 0.003 | 1.80E-02 | 0.0004 | 0.002 | 8.40E-01 | 0.0101 | 0.009 | 2.65E-01 |
| rs35039375 | A | G | 0.0198 | 0.003 | 1.00E-10 | 0.0119 | 0.005 | 1.00E-02 | -0.0106 | 0.004 | 2.80E-03 | -0.0016 | 0.015 | 9.19E-01 |
| rs35309068 | T | G | -0.0132 | 0.002 | 1.00E-10 | -0.0108 | 0.003 | 5.10E-05 | 0.0006 | 0.002 | 7.80E-01 | -0.0139 | 0.009 | 1.11E-01 |
| rs35316276 | C | T | -0.0117 | 0.002 | 1.00E-10 | -0.0114 | 0.003 | 1.10E-04 | 0.0045 | 0.002 | 4.70E-02 | 0.0117 | 0.010 | 2.46E-01 |
| rs35351411 | A | C | 0.0003 | 0.002 | 4.28E-02 | 0.0004 | 0.003 | 8.80E-01 | 0.0029 | 0.002 | 1.60E-01 | -0.0635 | 0.009 | 2.21E-13 |
| rs35417702 | C | T | 0.0145 | 0.002 | 1.00E-10 | 0.0073 | 0.003 | 5.90E-03 | -0.0009 | 0.002 | 6.50E-01 | 0.0478 | 0.009 | 2.78E-08 |
| rs35475880 | G | T | 0.0151 | 0.002 | 1.00E-10 | 0.0126 | 0.003 | 1.20E-04 | -0.0053 | 0.003 | 3.50E-02 | 0.0218 | 0.011 | 4.42E-02 |
| rs36119825 | G | A | -0.0106 | 0.002 | 1.00E-10 | 0.0010 | 0.003 | 7.10E-01 | 0.0020 | 0.002 | 3.20E-01 | -0.0145 | 0.009 | 9.26E-02 |
| rs363096 | T | C | -0.0136 | 0.002 | 1.00E-10 | -0.0101 | 0.003 | 1.60E-04 | 0.0046 | 0.002 | 2.40E-02 | 0.0163 | 0.009 | 6.22E-02 |
| rs3739118 | G | A | -0.0024 | 0.002 | 5.48E-05 | -0.0066 | 0.003 | 2.40E-02 | 0.0030 | 0.002 | 1.90E-01 | 0.0570 | 0.010 | 2.36E-09 |
| rs3791710 | T | C | 0.0054 | 0.002 | 3.75E-10 | 0.0014 | 0.003 | 6.80E-01 | 0.0025 | 0.003 | 3.20E-01 | 0.0600 | 0.011 | 3.02E-08 |
| rs3802924 | A | C | -0.0084 | 0.002 | 1.01E-10 | -0.0074 | 0.003 | 2.70E-02 | 0.0048 | 0.003 | 5.90E-02 | 0.0736 | 0.011 | 9.58E-12 |
| rs3814883 | C | T | 0.0005 | 0.002 | 3.55E-02 | 0.0005 | 0.003 | 8.50E-01 | -0.0022 | 0.002 | 2.90E-01 | 0.0671 | 0.009 | 1.58E-14 |
| rs3824451 | T | C | 0.0027 | 0.002 | 1.92E-04 | 0.0031 | 0.004 | 3.80E-01 | -0.0015 | 0.003 | 5.90E-01 | -0.0656 | 0.012 | 2.54E-08 |
| rs4115668 | G | A | 0.0165 | 0.002 | 1.00E-10 | 0.0180 | 0.003 | 1.10E-10 | -0.0017 | 0.002 | 4.30E-01 | -0.0187 | 0.009 | 4.24E-02 |
| rs4129585 | A | C | -0.0114 | 0.002 | 1.00E-10 | -0.0092 | 0.003 | 5.30E-04 | 0.0042 | 0.002 | 3.80E-02 | 0.0750 | 0.009 | 5.11E-18 |
| rs4382592 | T | G | -0.0164 | 0.002 | 1.00E-10 | -0.0003 | 0.003 | 9.10E-01 | 0.0028 | 0.002 | 2.10E-01 | 0.0002 | 0.009 | 9.81E-01 |
| rs4384309 | G | A | -0.0109 | 0.002 | 1.00E-10 | -0.0093 | 0.003 | 4.90E-04 | 0.0074 | 0.002 | 2.90E-04 | -0.0024 | 0.009 | 7.88E-01 |
| rs4392737 | A | G | 0.0097 | 0.002 | 1.00E-10 | 0.0094 | 0.003 | 4.90E-04 | -0.0037 | 0.002 | 7.70E-02 | -0.0033 | 0.009 | 7.05E-01 |
| rs4442732 | A | G | 0.0106 | 0.002 | 1.00E-10 | 0.0072 | 0.003 | 8.10E-03 | -0.0052 | 0.002 | 1.40E-02 | -0.0231 | 0.009 | 9.37E-03 |
| rs4632195 | C | T | 0.0104 | 0.002 | 1.00E-10 | 0.0136 | 0.003 | 2.90E-07 | -0.0021 | 0.002 | 3.10E-01 | -0.0472 | 0.009 | 4.59E-08 |
| rs4667025 | G | A | -0.0096 | 0.002 | 1.00E-10 | -0.0053 | 0.003 | 5.00E-02 | 0.0046 | 0.002 | 2.80E-02 | 0.0269 | 0.009 | 2.40E-03 |
| rs4702 | G | A | 0.0001 | 0.002 | 5.17E-02 | 0.0056 | 0.003 | 3.50E-02 | 0.0004 | 0.002 | 8.50E-01 | 0.0843 | 0.009 | 2.79E-21 |
| rs4726070 | G | A | -0.0125 | 0.002 | 1.00E-10 | -0.0063 | 0.003 | 1.90E-02 | 0.0029 | 0.002 | 1.60E-01 | 0.0002 | 0.009 | 9.82E-01 |
| rs4766428 | C | T | 0.0016 | 0.002 | 1.32E-03 | -0.0030 | 0.003 | 2.70E-01 | 0.0035 | 0.002 | 9.00E-02 | -0.0750 | 0.009 | 3.93E-17 |
| rs4779050 | T | G | -0.0032 | 0.002 | 6.83E-08 | -0.0004 | 0.003 | 8.90E-01 | -0.0010 | 0.002 | 6.30E-01 | 0.0580 | 0.009 | 7.27E-11 |
| rs4785187 | G | A | 0.0106 | 0.002 | 1.00E-10 | 0.0122 | 0.003 | 1.30E-04 | -0.0138 | 0.002 | 1.50E-08 | -0.0021 | 0.010 | 8.40E-01 |
| rs4810227 | G | A | -0.0127 | 0.002 | 1.00E-10 | -0.0104 | 0.003 | 1.40E-04 | 0.0014 | 0.002 | 5.10E-01 | 0.0084 | 0.009 | 3.47E-01 |
| rs4812325 | G | A | -0.0052 | 0.002 | 1.38E-10 | -0.0018 | 0.003 | 5.00E-01 | -0.0012 | 0.002 | 5.80E-01 | -0.0719 | 0.009 | 8.96E-16 |
| rs4839155 | T | G | 0.0125 | 0.002 | 1.00E-10 | 0.0015 | 0.003 | 6.30E-01 | -0.0010 | 0.002 | 6.70E-01 | 0.0067 | 0.010 | 5.10E-01 |
| rs4846724 | G | A | -0.0102 | 0.002 | 1.00E-10 | -0.0077 | 0.003 | 3.90E-03 | 0.0048 | 0.002 | 1.80E-02 | -0.0139 | 0.009 | 1.07E-01 |
| rs4888746 | A | G | 0.0095 | 0.002 | 1.00E-10 | -0.0006 | 0.003 | 8.40E-01 | -0.0031 | 0.002 | 1.50E-01 | -0.0099 | 0.009 | 2.69E-01 |
| rs4904523 | G | A | 0.0094 | 0.002 | 1.00E-10 | 0.0052 | 0.003 | 5.10E-02 | 0.0030 | 0.002 | 1.50E-01 | 0.0023 | 0.009 | 7.90E-01 |
| rs4945424 | C | A | 0.0099 | 0.002 | 1.00E-10 | 0.0065 | 0.003 | 1.60E-02 | -0.0026 | 0.002 | 2.00E-01 | 0.0169 | 0.009 | 5.45E-02 |
| rs4964046 | A | G | -0.0105 | 0.002 | 1.00E-10 | -0.0085 | 0.003 | 2.10E-03 | 0.0017 | 0.002 | 4.20E-01 | -0.0148 | 0.009 | 9.94E-02 |
| rs4972400 | G | A | -0.0116 | 0.002 | 1.00E-10 | -0.0042 | 0.003 | 1.30E-01 | -0.0005 | 0.002 | 8.00E-01 | -0.0212 | 0.010 | 2.52E-02 |
| rs56335113 | A | G | 0.0029 | 0.002 | 1.33E-06 | -0.0004 | 0.003 | 8.90E-01 | -0.0021 | 0.002 | 3.40E-01 | 0.0647 | 0.009 | 6.02E-12 |
| rs575113 | G | A | -0.0129 | 0.002 | 1.00E-10 | -0.0060 | 0.003 | 4.10E-02 | -0.0043 | 0.002 | 5.40E-02 | -0.0123 | 0.009 | 1.92E-01 |
| rs5751191 | T | C | 0.0062 | 0.002 | 1.03E-10 | 0.0053 | 0.003 | 4.80E-02 | 0.0059 | 0.002 | 3.80E-03 | -0.0656 | 0.009 | 3.00E-14 |
| rs58120505 | T | C | 0.0056 | 0.002 | 1.15E-10 | -0.0046 | 0.003 | 8.20E-02 | 0.0022 | 0.002 | 2.90E-01 | 0.0896 | 0.009 | 2.24E-24 |
| rs59123361 | G | A | 0.0209 | 0.003 | 1.00E-10 | 0.0104 | 0.004 | 1.60E-02 | -0.0070 | 0.003 | 3.50E-02 | -0.0056 | 0.015 | 7.06E-01 |
| rs60135207 | G | T | -0.0128 | 0.002 | 1.00E-10 | -0.0139 | 0.003 | 2.10E-07 | 0.0036 | 0.002 | 7.90E-02 | 0.0496 | 0.009 | 1.53E-08 |
| rs6122735 | C | T | -0.0105 | 0.002 | 1.00E-10 | 0.0072 | 0.003 | 7.70E-03 | 0.0000 | 0.002 | 9.90E-01 | -0.0159 | 0.009 | 7.02E-02 |
| rs6123924 | A | G | 0.0153 | 0.002 | 1.00E-10 | 0.0098 | 0.004 | 7.90E-03 | -0.0024 | 0.003 | 4.00E-01 | -0.0090 | 0.012 | 4.49E-01 |
| rs61747885 | G | T | -0.0138 | 0.002 | 1.00E-10 | -0.0110 | 0.004 | 2.70E-03 | -0.0008 | 0.003 | 7.80E-01 | -0.0051 | 0.012 | 6.67E-01 |
| rs61937595 | C | T | -0.0047 | 0.003 | 1.84E-06 | -0.0033 | 0.005 | 4.80E-01 | -0.0109 | 0.004 | 2.10E-03 | 0.1301 | 0.016 | 1.15E-15 |
| rs62183028 | G | T | 0.0140 | 0.002 | 1.00E-10 | 0.0190 | 0.003 | 3.20E-11 | -0.0028 | 0.002 | 2.10E-01 | -0.0056 | 0.010 | 5.51E-01 |
| rs62183776 | C | T | 0.0131 | 0.002 | 1.00E-10 | 0.0066 | 0.003 | 5.00E-02 | 0.0028 | 0.003 | 2.80E-01 | 0.0189 | 0.011 | 9.07E-02 |
| rs622169 | C | T | -0.0100 | 0.002 | 1.00E-10 | -0.0050 | 0.003 | 6.90E-02 | -0.0020 | 0.002 | 3.60E-01 | -0.0316 | 0.010 | 8.64E-04 |
| rs62439690 | G | A | 0.0109 | 0.002 | 1.00E-10 | 0.0034 | 0.003 | 2.60E-01 | -0.0028 | 0.002 | 2.30E-01 | 0.0050 | 0.010 | 6.21E-01 |
| rs634940 | G | T | -0.0033 | 0.002 | 3.99E-07 | -0.0065 | 0.003 | 3.20E-02 | -0.0014 | 0.002 | 5.50E-01 | -0.0664 | 0.010 | 1.78E-11 |
| rs6482437 | A | C | -0.0087 | 0.003 | 1.15E-10 | -0.0086 | 0.004 | 4.10E-02 | 0.0042 | 0.003 | 1.90E-01 | -0.0989 | 0.014 | 3.33E-12 |
| rs6493265 | C | T | 0.0139 | 0.002 | 1.00E-10 | 0.0086 | 0.003 | 1.50E-03 | -0.0015 | 0.002 | 4.60E-01 | -0.0179 | 0.009 | 4.17E-02 |
| rs6520064 | A | G | -0.0024 | 0.002 | 2.53E-04 | 0.0004 | 0.003 | 9.00E-01 | -0.0049 | 0.003 | 5.20E-02 | -0.0585 | 0.011 | 3.58E-08 |
| rs6538539 | G | T | -0.0029 | 0.002 | 2.59E-07 | -0.0073 | 0.003 | 5.90E-03 | 0.0033 | 0.002 | 1.10E-01 | 0.0568 | 0.009 | 4.43E-11 |
| rs6546857 | A | G | 0.0083 | 0.002 | 1.00E-10 | 0.0140 | 0.003 | 6.70E-06 | -0.0032 | 0.002 | 1.80E-01 | -0.0604 | 0.010 | 2.74E-09 |
| rs6549963 | T | C | -0.0006 | 0.002 | 2.72E-02 | 0.0054 | 0.003 | 4.70E-02 | -0.0013 | 0.002 | 5.30E-01 | 0.0483 | 0.009 | 4.31E-08 |
| rs6557171 | T | C | -0.0157 | 0.002 | 1.00E-10 | -0.0115 | 0.003 | 3.70E-05 | 0.0047 | 0.002 | 2.90E-02 | -0.0008 | 0.009 | 9.29E-01 |
| rs66568921 | T | G | -0.0157 | 0.002 | 1.00E-10 | -0.0086 | 0.003 | 2.10E-03 | 0.0024 | 0.002 | 2.60E-01 | -0.0085 | 0.009 | 3.41E-01 |
| rs6673880 | A | G | -0.0018 | 0.002 | 8.00E-04 | 0.0026 | 0.003 | 3.30E-01 | 0.0002 | 0.002 | 9.10E-01 | -0.0623 | 0.009 | 7.20E-12 |
| rs6699397 | A | G | 0.0165 | 0.002 | 1.00E-10 | 0.0191 | 0.003 | 3.50E-12 | -0.0137 | 0.002 | 8.00E-11 | -0.0279 | 0.009 | 1.61E-03 |
| rs6731373 | G | A | 0.0126 | 0.002 | 1.00E-10 | 0.0021 | 0.003 | 4.50E-01 | 0.0032 | 0.002 | 1.30E-01 | 0.0236 | 0.009 | 1.23E-02 |
| rs6798742 | A | G | 0.0002 | 0.002 | 4.79E-02 | 0.0010 | 0.003 | 7.40E-01 | 0.0007 | 0.002 | 7.70E-01 | -0.0611 | 0.009 | 4.57E-11 |
| rs6803651 | G | T | -0.0113 | 0.002 | 1.00E-10 | -0.0021 | 0.003 | 4.20E-01 | 0.0012 | 0.002 | 5.40E-01 | 0.0097 | 0.009 | 2.75E-01 |
| rs6805241 | T | C | 0.0141 | 0.002 | 1.00E-10 | 0.0109 | 0.003 | 5.20E-04 | 0.0021 | 0.002 | 3.80E-01 | 0.0083 | 0.010 | 4.24E-01 |
| rs6938002 | G | A | 0.0101 | 0.002 | 1.00E-10 | 0.0056 | 0.003 | 3.70E-02 | -0.0005 | 0.002 | 8.30E-01 | 0.0122 | 0.009 | 1.66E-01 |
| rs6943762 | T | C | 0.0030 | 0.003 | 1.78E-04 | -0.0073 | 0.004 | 6.60E-02 | -0.0029 | 0.003 | 3.40E-01 | 0.1051 | 0.013 | 1.57E-15 |
| rs6959891 | A | G | 0.0114 | 0.002 | 1.00E-10 | 0.0048 | 0.003 | 1.00E-01 | 0.0020 | 0.002 | 3.80E-01 | -0.0050 | 0.010 | 6.04E-01 |
| rs7012546 | C | T | -0.0101 | 0.002 | 1.00E-10 | -0.0055 | 0.003 | 4.00E-02 | -0.0032 | 0.002 | 1.20E-01 | 0.0067 | 0.009 | 4.42E-01 |
| rs7031698 | T | C | -0.0125 | 0.002 | 1.00E-10 | -0.0112 | 0.003 | 3.90E-04 | 0.0038 | 0.002 | 1.20E-01 | 0.0064 | 0.011 | 5.44E-01 |
| rs708228 | C | T | 0.0097 | 0.002 | 1.00E-10 | 0.0149 | 0.003 | 1.40E-07 | -0.0084 | 0.002 | 9.60E-05 | -0.0528 | 0.009 | 6.56E-09 |
| rs710629 | G | A | -0.0105 | 0.002 | 1.00E-10 | -0.0017 | 0.003 | 5.30E-01 | 0.0018 | 0.002 | 4.00E-01 | 0.0141 | 0.009 | 1.17E-01 |
| rs7112616 | T | C | 0.0060 | 0.002 | 1.05E-10 | 0.0051 | 0.003 | 5.60E-02 | -0.0005 | 0.002 | 7.90E-01 | 0.0522 | 0.009 | 1.52E-09 |
| rs71646142 | C | T | -0.0129 | 0.002 | 1.00E-10 | -0.0099 | 0.003 | 3.30E-03 | 0.0008 | 0.003 | 7.50E-01 | 0.0178 | 0.011 | 1.08E-01 |
| rs7257460 | T | C | 0.0115 | 0.002 | 1.00E-10 | -0.0035 | 0.003 | 2.40E-01 | 0.0050 | 0.002 | 2.70E-02 | 0.0033 | 0.010 | 7.29E-01 |
| rs72802868 | G | T | 0.0097 | 0.002 | 1.00E-10 | -0.0025 | 0.003 | 4.00E-01 | 0.0086 | 0.002 | 1.10E-04 | 0.0692 | 0.010 | 4.55E-13 |
| rs72840994 | T | G | -0.0125 | 0.002 | 1.00E-10 | -0.0071 | 0.003 | 3.40E-02 | 0.0060 | 0.003 | 1.90E-02 | -0.0085 | 0.011 | 4.44E-01 |
| rs730384 | G | A | -0.0102 | 0.002 | 1.00E-10 | -0.0083 | 0.003 | 2.10E-03 | 0.0048 | 0.002 | 1.80E-02 | 0.0223 | 0.009 | 1.05E-02 |
| rs7321274 | A | G | 0.0128 | 0.002 | 1.00E-10 | 0.0093 | 0.003 | 4.60E-03 | -0.0055 | 0.003 | 2.90E-02 | -0.0118 | 0.011 | 2.75E-01 |
| rs73229090 | C | A | -0.0015 | 0.003 | 1.30E-02 | -0.0068 | 0.004 | 1.00E-01 | 0.0014 | 0.003 | 6.60E-01 | 0.1026 | 0.014 | 4.34E-13 |
| rs73301698 | G | A | 0.0129 | 0.002 | 1.00E-10 | 0.0082 | 0.003 | 1.20E-02 | -0.0039 | 0.003 | 1.20E-01 | -0.0046 | 0.011 | 6.64E-01 |
| rs7332724 | C | T | 0.0115 | 0.002 | 1.00E-10 | 0.0056 | 0.003 | 5.70E-02 | -0.0077 | 0.002 | 7.20E-04 | -0.0277 | 0.010 | 3.85E-03 |
| rs736282 | T | C | 0.0108 | 0.002 | 1.00E-10 | 0.0039 | 0.003 | 1.40E-01 | -0.0016 | 0.002 | 4.40E-01 | 0.0060 | 0.009 | 4.85E-01 |
| rs73874335 | C | T | 0.0199 | 0.004 | 1.00E-10 | 0.0123 | 0.006 | 3.40E-02 | -0.0053 | 0.004 | 2.30E-01 | -0.0251 | 0.018 | 1.64E-01 |
| rs743316 | T | C | 0.0119 | 0.002 | 1.00E-10 | 0.0098 | 0.003 | 2.70E-03 | -0.0008 | 0.002 | 7.60E-01 | -0.0207 | 0.011 | 5.04E-02 |
| rs74643044 | T | C | -0.0232 | 0.004 | 1.00E-10 | -0.0110 | 0.006 | 6.40E-02 | 0.0095 | 0.005 | 3.60E-02 | 0.0042 | 0.021 | 8.46E-01 |
| rs74701752 | G | T | -0.0159 | 0.003 | 1.00E-10 | -0.0216 | 0.004 | 1.20E-06 | 0.0047 | 0.003 | 1.70E-01 | 0.0302 | 0.015 | 4.35E-02 |
| rs7515363 | C | T | 0.0003 | 0.002 | 4.25E-02 | -0.0014 | 0.003 | 6.00E-01 | 0.0018 | 0.002 | 3.90E-01 | 0.0535 | 0.009 | 1.84E-09 |
| rs7603132 | G | A | -0.0132 | 0.002 | 1.00E-10 | -0.0111 | 0.003 | 9.00E-04 | 0.0068 | 0.003 | 8.10E-03 | 0.0210 | 0.011 | 5.83E-02 |
| rs76076331 | C | T | -0.0187 | 0.002 | 1.00E-10 | -0.0182 | 0.004 | 6.30E-06 | 0.0098 | 0.003 | 1.60E-03 | 0.0481 | 0.013 | 1.86E-04 |
| rs7634476 | A | G | 0.0016 | 0.002 | 1.96E-03 | 0.0059 | 0.003 | 2.90E-02 | 0.0022 | 0.002 | 2.80E-01 | -0.0577 | 0.009 | 5.46E-11 |
| rs7647398 | C | T | 0.0003 | 0.002 | 4.38E-02 | -0.0024 | 0.003 | 4.70E-01 | -0.0035 | 0.003 | 1.70E-01 | 0.0775 | 0.011 | 1.07E-12 |
| rs76608582 | C | A | -0.0280 | 0.004 | 1.00E-10 | -0.0291 | 0.007 | 9.30E-06 | 0.0121 | 0.005 | 1.60E-02 | -0.0186 | 0.026 | 4.67E-01 |
| rs77835879 | A | G | 0.0160 | 0.003 | 1.00E-10 | 0.0141 | 0.004 | 1.40E-03 | -0.0014 | 0.003 | 6.80E-01 | 0.0230 | 0.015 | 1.25E-01 |
| rs778371 | A | G | -0.0100 | 0.002 | 1.00E-10 | -0.0011 | 0.003 | 7.00E-01 | -0.0005 | 0.002 | 8.20E-01 | -0.0806 | 0.010 | 1.50E-17 |
| rs7798283 | T | G | -0.0059 | 0.003 | 1.21E-09 | -0.0053 | 0.004 | 1.90E-01 | 0.0020 | 0.003 | 5.10E-01 | 0.0740 | 0.013 | 3.49E-08 |
| rs7803932 | G | A | -0.0143 | 0.002 | 1.00E-10 | -0.0095 | 0.004 | 7.00E-03 | 0.0058 | 0.003 | 3.30E-02 | 0.0366 | 0.012 | 1.89E-03 |
| rs7808399 | A | G | -0.0107 | 0.002 | 1.00E-10 | -0.0160 | 0.003 | 2.20E-09 | 0.0011 | 0.002 | 5.70E-01 | -0.0150 | 0.009 | 8.38E-02 |
| rs78257128 | C | T | -0.0026 | 0.002 | 4.78E-04 | -0.0063 | 0.004 | 8.70E-02 | 0.0167 | 0.003 | 2.40E-09 | 0.0114 | 0.013 | 3.86E-01 |
| rs78721320 | G | A | -0.0131 | 0.002 | 1.00E-10 | -0.0127 | 0.003 | 1.50E-04 | 0.0011 | 0.003 | 6.70E-01 | 0.0032 | 0.012 | 7.81E-01 |
| rs7896518 | A | G | -0.0142 | 0.002 | 1.00E-10 | -0.0148 | 0.003 | 4.10E-08 | 0.0035 | 0.002 | 9.30E-02 | -0.0019 | 0.009 | 8.33E-01 |
| rs79265434 | A | G | -0.0233 | 0.003 | 1.00E-10 | -0.0021 | 0.004 | 6.10E-01 | -0.0043 | 0.003 | 1.70E-01 | -0.0840 | 0.014 | 6.63E-10 |
| rs79269403 | G | A | -0.0145 | 0.002 | 1.00E-10 | -0.0036 | 0.003 | 2.60E-01 | 0.0030 | 0.002 | 2.20E-01 | 0.0055 | 0.011 | 6.02E-01 |
| rs79445414 | T | C | 0.0027 | 0.004 | 8.69E-03 | 0.0088 | 0.006 | 1.70E-01 | -0.0112 | 0.005 | 2.10E-02 | -0.1234 | 0.022 | 2.80E-08 |
| rs795230 | C | T | -0.0095 | 0.002 | 1.00E-10 | -0.0035 | 0.003 | 1.90E-01 | -0.0034 | 0.002 | 9.30E-02 | 0.0097 | 0.009 | 2.72E-01 |
| rs7993663 | T | C | -0.0118 | 0.002 | 1.00E-10 | -0.0067 | 0.003 | 1.50E-02 | 0.0034 | 0.002 | 1.00E-01 | 0.0168 | 0.009 | 6.03E-02 |
| rs80171383 | G | A | -0.0145 | 0.002 | 1.00E-10 | -0.0127 | 0.004 | 7.80E-04 | 0.0070 | 0.003 | 1.60E-02 | 0.0227 | 0.013 | 7.71E-02 |
| rs8055219 | G | A | -0.0084 | 0.002 | 1.00E-10 | 0.0065 | 0.003 | 4.00E-02 | -0.0065 | 0.002 | 7.10E-03 | -0.0665 | 0.010 | 5.69E-11 |
| rs818415 | T | G | -0.0124 | 0.002 | 1.00E-10 | -0.0072 | 0.003 | 3.30E-02 | -0.0022 | 0.003 | 3.90E-01 | -0.0280 | 0.011 | 1.03E-02 |
| rs837080 | T | C | -0.0109 | 0.002 | 1.00E-10 | -0.0086 | 0.003 | 1.20E-03 | 0.0029 | 0.002 | 1.50E-01 | 0.0440 | 0.009 | 4.05E-07 |
| rs892612 | A | C | -0.0146 | 0.002 | 1.00E-10 | -0.0125 | 0.004 | 6.50E-04 | 0.0040 | 0.003 | 1.50E-01 | -0.0290 | 0.012 | 1.63E-02 |
| rs894067 | G | A | -0.0104 | 0.002 | 1.00E-10 | -0.0076 | 0.003 | 5.30E-03 | 0.0007 | 0.002 | 7.40E-01 | -0.0171 | 0.009 | 5.31E-02 |
| rs9318627 | A | C | -0.0001 | 0.002 | 5.02E-02 | 0.0028 | 0.003 | 3.00E-01 | 0.0009 | 0.002 | 6.50E-01 | 0.0612 | 0.009 | 4.35E-12 |
| rs9342482 | G | T | -0.0126 | 0.002 | 1.00E-10 | -0.0068 | 0.003 | 2.60E-02 | 0.0015 | 0.002 | 5.20E-01 | 0.0128 | 0.010 | 1.99E-01 |
| rs9349956 | A | C | -0.0188 | 0.002 | 1.00E-10 | -0.0114 | 0.003 | 7.90E-04 | 0.0006 | 0.003 | 8.20E-01 | 0.0049 | 0.011 | 6.67E-01 |
| rs9384679 | C | T | 0.0096 | 0.002 | 1.00E-10 | 0.0109 | 0.003 | 6.40E-05 | -0.0030 | 0.002 | 1.50E-01 | -0.0203 | 0.009 | 2.22E-02 |
| rs9386787 | A | G | -0.0096 | 0.002 | 1.00E-10 | -0.0021 | 0.003 | 4.30E-01 | 0.0027 | 0.002 | 1.90E-01 | -0.0056 | 0.009 | 5.16E-01 |
| rs9436866 | A | C | -0.0188 | 0.003 | 1.00E-10 | -0.0050 | 0.004 | 2.70E-01 | 0.0004 | 0.003 | 9.10E-01 | -0.0180 | 0.014 | 2.06E-01 |
| rs9461916 | T | C | -0.0026 | 0.002 | 4.99E-06 | 0.0035 | 0.003 | 2.00E-01 | -0.0003 | 0.002 | 8.80E-01 | -0.0533 | 0.009 | 1.64E-09 |
| rs9556958 | C | T | 0.0108 | 0.002 | 1.00E-10 | 0.0153 | 0.003 | 8.50E-09 | -0.0061 | 0.002 | 2.90E-03 | 0.0181 | 0.009 | 3.59E-02 |
| rs9616906 | G | A | -0.0150 | 0.002 | 1.00E-10 | -0.0074 | 0.003 | 5.60E-03 | 0.0008 | 0.002 | 6.80E-01 | -0.0244 | 0.009 | 4.82E-03 |
| rs9636107 | A | G | 0.0037 | 0.002 | 2.34E-09 | 0.0065 | 0.003 | 1.50E-02 | -0.0088 | 0.002 | 1.70E-05 | -0.0699 | 0.009 | 5.12E-16 |
| rs9679654 | T | C | -0.0104 | 0.002 | 1.00E-10 | -0.0128 | 0.003 | 1.80E-06 | 0.0030 | 0.002 | 1.40E-01 | -0.0085 | 0.009 | 3.33E-01 |
| rs968050 | C | T | -0.0224 | 0.002 | 1.00E-10 | -0.0224 | 0.003 | 3.10E-17 | 0.0100 | 0.002 | 9.50E-07 | -0.0319 | 0.009 | 2.12E-04 |
| rs9704097 | C | A | 0.0103 | 0.002 | 1.00E-10 | 0.0100 | 0.003 | 1.60E-04 | -0.0045 | 0.002 | 2.50E-02 | 0.0073 | 0.009 | 3.94E-01 |
| rs9876421 | C | T | -0.0052 | 0.002 | 1.56E-10 | 0.0009 | 0.003 | 7.60E-01 | -0.0013 | 0.002 | 5.40E-01 | -0.0625 | 0.009 | 9.19E-12 |
| rs989532 | A | G | 0.0071 | 0.002 | 1.01E-10 | 0.0132 | 0.003 | 2.00E-06 | -0.0139 | 0.002 | 7.40E-11 | -0.0245 | 0.009 | 6.32E-03 |
| rs9914918 | G | A | -0.0116 | 0.002 | 1.00E-10 | -0.0119 | 0.003 | 4.20E-05 | -0.0032 | 0.002 | 1.60E-01 | 0.0329 | 0.010 | 5.37E-04 |
| rs9933256 | A | G | 0.0113 | 0.002 | 1.00E-10 | 0.0128 | 0.003 | 1.30E-06 | -0.0040 | 0.002 | 4.70E-02 | 0.0078 | 0.009 | 3.98E-01 |
| rs9995567 | G | A | -0.0100 | 0.002 | 1.00E-10 | -0.0089 | 0.003 | 1.30E-03 | 0.0051 | 0.002 | 1.60E-02 | 0.0110 | 0.009 | 2.25E-01 |

Abbreviations: GWAS, genome-wide association studies; MDD, major depressive disorder; MR, Mendelian randomisation; SE, standard error; SES, socioeconomic status; SNP, single nucleotide polymorphism; TDI, Townsend deprivation index.

## Table S13. Summary information on genetic instruments for SES-MDD multivariable MR analysis in MDD GWAS dataset.

| **SNP** | **Effect Allele** | **Alternate Allele** | **Beta** | **SE** | ***p* value** |
| --- | --- | --- | --- | --- | --- |
| rs10035564 | A | G | 0.0161 | 0.010 | 9.12E-02 |
| rs10073890 | A | G | 0.0113 | 0.010 | 2.72E-01 |
| rs10086619 | A | G | -0.0139 | 0.012 | 2.41E-01 |
| rs10108980 | C | T | -0.0044 | 0.011 | 6.91E-01 |
| rs10189857 | A | G | 0.0041 | 0.009 | 6.45E-01 |
| rs10215082 | A | G | 0.0047 | 0.009 | 6.04E-01 |
| rs10240905 | T | C | 0.0138 | 0.009 | 1.36E-01 |
| rs10429582 | T | C | 0.0071 | 0.009 | 4.37E-01 |
| rs10456918 | A | C | 0.0100 | 0.012 | 3.92E-01 |
| rs10460095 | G | A | -0.0048 | 0.009 | 5.95E-01 |
| rs1051474 | T | C | 0.0102 | 0.010 | 3.04E-01 |
| rs10765775 | G | A | 0.0107 | 0.009 | 2.41E-01 |
| rs10798418 | C | T | -0.0141 | 0.009 | 1.18E-01 |
| rs10861176 | G | A | 0.0111 | 0.010 | 2.76E-01 |
| rs10887801 | G | T | -0.0094 | 0.009 | 2.94E-01 |
| rs10940921 | T | G | -0.0079 | 0.009 | 3.80E-01 |
| rs10994777 | G | A | -0.0016 | 0.012 | 8.96E-01 |
| rs11023749 | G | A | -0.0146 | 0.009 | 1.18E-01 |
| rs1105307 | G | A | 0.0026 | 0.010 | 8.02E-01 |
| rs11081529 | T | C | -0.0071 | 0.010 | 4.76E-01 |
| rs11136325 | G | A | 0.0168 | 0.010 | 9.16E-02 |
| rs111821073 | C | T | 0.0235 | 0.012 | 5.60E-02 |
| rs11191580 | T | C | 0.0448 | 0.016 | 4.45E-03 |
| rs11210892 | G | A | 0.0042 | 0.010 | 6.56E-01 |
| rs112687095 | G | A | 0.0123 | 0.015 | 4.21E-01 |
| rs113615161 | C | T | -0.0050 | 0.013 | 7.08E-01 |
| rs1143770 | C | T | 0.0161 | 0.009 | 7.49E-02 |
| rs11534045 | G | A | 0.0028 | 0.010 | 7.73E-01 |
| rs115454970 | G | T | -0.0152 | 0.011 | 1.56E-01 |
| rs11588857 | G | A | 0.0018 | 0.011 | 8.68E-01 |
| rs11601122 | A | G | -0.0114 | 0.012 | 3.53E-01 |
| rs11620355 | G | A | 0.0377 | 0.016 | 1.73E-02 |
| rs11627087 | A | G | 0.0172 | 0.016 | 2.90E-01 |
| rs11635092 | G | A | 0.0170 | 0.010 | 7.65E-02 |
| rs11657342 | G | A | -0.0084 | 0.011 | 4.41E-01 |
| rs11664298 | G | A | -0.0393 | 0.011 | 4.80E-04 |
| rs11678980 | G | A | -0.0037 | 0.010 | 7.03E-01 |
| rs11732657 | G | A | -0.0059 | 0.010 | 5.70E-01 |
| rs11752914 | T | C | -0.0104 | 0.011 | 3.65E-01 |
| rs11772580 | G | T | 0.0008 | 0.011 | 9.41E-01 |
| rs11855821 | G | A | 0.0171 | 0.010 | 9.69E-02 |
| rs11877758 | T | G | -0.0244 | 0.010 | 1.10E-02 |
| rs11941714 | G | A | 0.0155 | 0.010 | 1.09E-01 |
| rs12028010 | T | C | 0.0090 | 0.011 | 3.91E-01 |
| rs12129573 | C | A | -0.0486 | 0.009 | 1.34E-07 |
| rs12151767 | G | A | 0.0142 | 0.009 | 1.12E-01 |
| rs12203592 | C | T | -0.0059 | 0.014 | 6.67E-01 |
| rs12285419 | C | A | -0.0153 | 0.011 | 1.78E-01 |
| rs12293670 | A | G | 0.0087 | 0.009 | 3.54E-01 |
| rs12375949 | T | C | 0.0028 | 0.009 | 7.53E-01 |
| rs12468040 | T | G | -0.0181 | 0.009 | 4.96E-02 |
| rs12489270 | T | C | 0.0019 | 0.009 | 8.32E-01 |
| rs12574281 | A | C | 0.0043 | 0.009 | 6.44E-01 |
| rs12652777 | T | C | 0.0018 | 0.009 | 8.39E-01 |
| rs12682775 | T | C | -0.0138 | 0.011 | 1.98E-01 |
| rs12712510 | T | C | 0.0219 | 0.009 | 1.53E-02 |
| rs12771371 | G | A | -0.0014 | 0.010 | 8.88E-01 |
| rs12833624 | C | T | -0.0089 | 0.009 | 3.44E-01 |
| rs12883788 | C | T | -0.0048 | 0.009 | 6.10E-01 |
| rs12940014 | T | C | 0.0010 | 0.009 | 9.08E-01 |
| rs13010566 | A | C | 0.0192 | 0.009 | 3.18E-02 |
| rs13016542 | T | C | -0.0058 | 0.013 | 6.54E-01 |
| rs13029509 | G | A | -0.0071 | 0.009 | 4.31E-01 |
| rs13107325 | C | T | 0.0269 | 0.019 | 1.62E-01 |
| rs13141210 | C | T | 0.0200 | 0.009 | 2.70E-02 |
| rs13145650 | C | T | -0.0249 | 0.017 | 1.33E-01 |
| rs13195636 | A | C | 0.0626 | 0.016 | 5.82E-05 |
| rs132582 | C | T | 0.0104 | 0.009 | 2.49E-01 |
| rs1334297 | G | A | 0.0196 | 0.010 | 4.91E-02 |
| rs13422673 | C | T | 0.0091 | 0.009 | 3.06E-01 |
| rs1381247 | T | C | 0.0145 | 0.010 | 1.62E-01 |
| rs1391438 | T | C | 0.0114 | 0.009 | 2.26E-01 |
| rs1430894 | C | T | -0.0058 | 0.009 | 5.12E-01 |
| rs145071536 | T | C | 0.0015 | 0.013 | 9.08E-01 |
| rs1450782 | T | G | -0.0001 | 0.009 | 9.89E-01 |
| rs1451488 | A | G | -0.0056 | 0.009 | 5.32E-01 |
| rs152603 | A | G | -0.0031 | 0.009 | 7.41E-01 |
| rs1558727 | C | T | 0.0092 | 0.009 | 3.11E-01 |
| rs1584469 | C | T | -0.0189 | 0.010 | 5.22E-02 |
| rs1595973 | C | T | -0.0076 | 0.009 | 4.02E-01 |
| rs1615350 | C | T | -0.0237 | 0.010 | 2.12E-02 |
| rs1618725 | C | T | -0.0224 | 0.009 | 1.16E-02 |
| rs1620977 | A | G | 0.0285 | 0.011 | 6.99E-03 |
| rs16846463 | A | G | -0.0031 | 0.015 | 8.33E-01 |
| rs16851048 | T | C | 0.0000 | 0.011 | 9.97E-01 |
| rs16854920 | T | C | 0.0147 | 0.010 | 1.41E-01 |
| rs16867571 | A | G | 0.0056 | 0.011 | 5.97E-01 |
| rs16995054 | C | T | -0.0166 | 0.011 | 1.21E-01 |
| rs17194490 | G | T | -0.0115 | 0.013 | 3.55E-01 |
| rs17425572 | A | G | -0.0033 | 0.009 | 7.14E-01 |
| rs17489649 | A | G | -0.0157 | 0.010 | 9.87E-02 |
| rs17551064 | A | G | -0.0372 | 0.012 | 2.14E-03 |
| rs17563464 | C | A | 0.0133 | 0.012 | 2.68E-01 |
| rs17565975 | G | A | 0.0048 | 0.009 | 5.95E-01 |
| rs17598675 | T | C | 0.0102 | 0.009 | 2.52E-01 |
| rs176218 | G | T | 0.0092 | 0.011 | 4.14E-01 |
| rs17731 | G | A | 0.0001 | 0.009 | 9.87E-01 |
| rs1866823 | G | A | 0.0006 | 0.009 | 9.44E-01 |
| rs1915019 | A | G | -0.0168 | 0.010 | 1.06E-01 |
| rs192436652 | C | T | 0.0069 | 0.030 | 8.19E-01 |
| rs1925576 | A | G | -0.0062 | 0.009 | 4.89E-01 |
| rs1964927 | A | G | -0.0149 | 0.009 | 1.08E-01 |
| rs215412 | G | A | -0.0015 | 0.009 | 8.72E-01 |
| rs217336 | C | A | -0.0092 | 0.009 | 3.02E-01 |
| rs2182505 | T | C | 0.0027 | 0.010 | 7.85E-01 |
| rs2238057 | T | G | -0.0087 | 0.009 | 3.33E-01 |
| rs2252074 | T | G | -0.0060 | 0.009 | 5.07E-01 |
| rs225291 | A | G | -0.0187 | 0.011 | 9.41E-02 |
| rs2283076 | A | G | 0.0002 | 0.011 | 9.83E-01 |
| rs2287838 | G | A | 0.0087 | 0.009 | 3.32E-01 |
| rs2302761 | C | T | 0.0332 | 0.011 | 2.80E-03 |
| rs2332719 | A | G | -0.0021 | 0.010 | 8.36E-01 |
| rs2333321 | A | G | 0.0207 | 0.011 | 5.50E-02 |
| rs2347526 | T | C | 0.0092 | 0.010 | 3.33E-01 |
| rs2381411 | T | C | -0.0006 | 0.009 | 9.49E-01 |
| rs242093 | G | A | -0.0094 | 0.009 | 3.01E-01 |
| rs2422859 | T | G | 0.0064 | 0.009 | 4.79E-01 |
| rs2441111 | G | A | -0.0188 | 0.009 | 3.64E-02 |
| rs2447535 | A | G | 0.0052 | 0.010 | 5.97E-01 |
| rs2456020 | C | T | 0.0019 | 0.011 | 8.59E-01 |
| rs2514218 | C | T | 0.0269 | 0.009 | 4.16E-03 |
| rs2554835 | G | A | -0.0099 | 0.009 | 2.90E-01 |
| rs2570497 | C | T | 0.0037 | 0.009 | 6.88E-01 |
| rs2710323 | T | C | 0.0243 | 0.009 | 6.53E-03 |
| rs2725370 | T | C | 0.0061 | 0.010 | 5.34E-01 |
| rs277828 | C | A | -0.0085 | 0.011 | 4.19E-01 |
| rs2787101 | C | T | 0.0161 | 0.009 | 7.77E-02 |
| rs2815731 | C | A | 0.0238 | 0.009 | 1.08E-02 |
| rs2820314 | A | C | -0.0081 | 0.010 | 3.99E-01 |
| rs28513670 | A | G | 0.0238 | 0.012 | 4.21E-02 |
| rs2885198 | A | G | -0.0202 | 0.009 | 2.83E-02 |
| rs2998315 | A | G | 0.0105 | 0.009 | 2.51E-01 |
| rs301800 | T | C | 0.0139 | 0.012 | 2.35E-01 |
| rs3026996 | A | C | -0.0078 | 0.011 | 4.65E-01 |
| rs31940 | G | A | 0.0113 | 0.013 | 3.81E-01 |
| rs32940 | T | C | -0.0094 | 0.010 | 3.41E-01 |
| rs337637 | G | A | 0.0162 | 0.009 | 8.39E-02 |
| rs34316 | A | C | 0.0265 | 0.009 | 4.54E-03 |
| rs34473884 | G | A | 0.0128 | 0.011 | 2.31E-01 |
| rs34485537 | C | T | 0.0175 | 0.009 | 6.03E-02 |
| rs35039375 | A | G | -0.0194 | 0.016 | 2.22E-01 |
| rs35309068 | T | G | -0.0086 | 0.009 | 3.32E-01 |
| rs35316276 | C | T | -0.0201 | 0.011 | 6.10E-02 |
| rs35351411 | A | C | -0.0047 | 0.009 | 6.02E-01 |
| rs35417702 | C | T | -0.0113 | 0.009 | 2.11E-01 |
| rs35475880 | G | T | -0.0104 | 0.011 | 3.43E-01 |
| rs36119825 | G | A | 0.0116 | 0.009 | 1.95E-01 |
| rs363096 | T | C | 0.0209 | 0.009 | 2.00E-02 |
| rs3739118 | G | A | 0.0272 | 0.010 | 5.81E-03 |
| rs3791710 | T | C | -0.0160 | 0.011 | 1.51E-01 |
| rs3802924 | A | C | 0.0116 | 0.011 | 2.87E-01 |
| rs3814883 | C | T | 0.0172 | 0.009 | 5.92E-02 |
| rs3824451 | T | C | -0.0091 | 0.012 | 4.53E-01 |
| rs4115668 | G | A | 0.0092 | 0.010 | 3.42E-01 |
| rs4129585 | A | C | 0.0108 | 0.009 | 2.38E-01 |
| rs4382592 | T | G | 0.0092 | 0.010 | 3.36E-01 |
| rs4384309 | G | A | -0.0065 | 0.009 | 4.90E-01 |
| rs4392737 | A | G | -0.0217 | 0.009 | 1.67E-02 |
| rs4442732 | A | G | 0.0074 | 0.009 | 4.14E-01 |
| rs4632195 | C | T | -0.0343 | 0.009 | 1.24E-04 |
| rs4667025 | G | A | -0.0045 | 0.009 | 6.20E-01 |
| rs4702 | G | A | 0.0263 | 0.010 | 5.68E-03 |
| rs4726070 | G | A | 0.0059 | 0.009 | 5.14E-01 |
| rs4766428 | C | T | -0.0199 | 0.009 | 2.83E-02 |
| rs4779050 | T | G | 0.0079 | 0.009 | 3.96E-01 |
| rs4785187 | G | A | 0.0100 | 0.011 | 3.62E-01 |
| rs4810227 | G | A | 0.0062 | 0.009 | 5.01E-01 |
| rs4812325 | G | A | -0.0128 | 0.009 | 1.70E-01 |
| rs4839155 | T | G | -0.0116 | 0.010 | 2.68E-01 |
| rs4846724 | G | A | -0.0017 | 0.009 | 8.53E-01 |
| rs4888746 | A | G | 0.0006 | 0.009 | 9.51E-01 |
| rs4904523 | G | A | 0.0003 | 0.009 | 9.71E-01 |
| rs4945424 | C | A | -0.0219 | 0.009 | 1.65E-02 |
| rs4964046 | A | G | -0.0032 | 0.009 | 7.28E-01 |
| rs4972400 | G | A | -0.0134 | 0.010 | 1.68E-01 |
| rs56335113 | A | G | 0.0209 | 0.010 | 3.23E-02 |
| rs575113 | G | A | -0.0308 | 0.010 | 1.83E-03 |
| rs5751191 | T | C | -0.0069 | 0.009 | 4.38E-01 |
| rs58120505 | T | C | -0.0116 | 0.009 | 2.11E-01 |
| rs59123361 | G | A | -0.0234 | 0.016 | 1.46E-01 |
| rs60135207 | G | T | 0.0067 | 0.009 | 4.55E-01 |
| rs6122735 | C | T | -0.0067 | 0.009 | 4.61E-01 |
| rs6123924 | A | G | -0.0017 | 0.013 | 8.92E-01 |
| rs61747885 | G | T | -0.0184 | 0.012 | 1.36E-01 |
| rs61937595 | C | T | -0.0047 | 0.016 | 7.73E-01 |
| rs62183028 | G | T | -0.0117 | 0.010 | 2.28E-01 |
| rs62183776 | C | T | 0.0129 | 0.012 | 2.63E-01 |
| rs622169 | C | T | -0.0162 | 0.010 | 1.05E-01 |
| rs62439690 | G | A | 0.0036 | 0.011 | 7.37E-01 |
| rs634940 | G | T | -0.0170 | 0.010 | 9.61E-02 |
| rs6482437 | A | C | -0.0292 | 0.015 | 4.68E-02 |
| rs6493265 | C | T | -0.0342 | 0.009 | 1.57E-04 |
| rs6520064 | A | G | -0.0051 | 0.011 | 6.47E-01 |
| rs6538539 | G | T | 0.0047 | 0.009 | 5.97E-01 |
| rs6546857 | A | G | -0.0117 | 0.011 | 2.69E-01 |
| rs6549963 | T | C | 0.0049 | 0.009 | 5.93E-01 |
| rs6557171 | T | C | 0.0344 | 0.010 | 3.05E-04 |
| rs66568921 | T | G | -0.0030 | 0.009 | 7.51E-01 |
| rs6673880 | A | G | -0.0078 | 0.011 | 4.84E-01 |
| rs6699397 | A | G | -0.0057 | 0.009 | 5.33E-01 |
| rs6731373 | G | A | 0.0022 | 0.010 | 8.19E-01 |
| rs6798742 | A | G | -0.0102 | 0.010 | 2.92E-01 |
| rs6803651 | G | T | -0.0069 | 0.009 | 4.52E-01 |
| rs6805241 | T | C | 0.0147 | 0.011 | 1.69E-01 |
| rs6938002 | G | A | -0.0165 | 0.009 | 7.33E-02 |
| rs6943762 | T | C | -0.0287 | 0.013 | 3.14E-02 |
| rs6959891 | A | G | -0.0062 | 0.010 | 5.30E-01 |
| rs7012546 | C | T | -0.0036 | 0.009 | 6.89E-01 |
| rs7031698 | T | C | 0.0299 | 0.011 | 5.65E-03 |
| rs708228 | C | T | -0.0318 | 0.009 | 7.27E-04 |
| rs710629 | G | A | 0.0040 | 0.009 | 6.69E-01 |
| rs7112616 | T | C | -0.0046 | 0.009 | 6.06E-01 |
| rs71646142 | C | T | 0.0167 | 0.012 | 1.45E-01 |
| rs7257460 | T | C | -0.0145 | 0.010 | 1.43E-01 |
| rs72802868 | G | T | -0.0027 | 0.010 | 7.80E-01 |
| rs72840994 | T | G | -0.0128 | 0.012 | 2.64E-01 |
| rs730384 | G | A | 0.0041 | 0.009 | 6.47E-01 |
| rs7321274 | A | G | -0.0034 | 0.011 | 7.57E-01 |
| rs73229090 | C | A | 0.0148 | 0.014 | 2.99E-01 |
| rs73301698 | G | A | -0.0100 | 0.011 | 3.62E-01 |
| rs7332724 | C | T | -0.0169 | 0.010 | 8.60E-02 |
| rs736282 | T | C | -0.0143 | 0.009 | 1.11E-01 |
| rs73874335 | C | T | 0.0408 | 0.019 | 2.97E-02 |
| rs743316 | T | C | -0.0345 | 0.011 | 1.56E-03 |
| rs74643044 | T | C | 0.0308 | 0.021 | 1.45E-01 |
| rs74701752 | G | T | 0.0014 | 0.015 | 9.28E-01 |
| rs7515363 | C | T | 0.0091 | 0.009 | 3.24E-01 |
| rs7603132 | G | A | 0.0082 | 0.011 | 4.71E-01 |
| rs76076331 | C | T | 0.0145 | 0.013 | 2.74E-01 |
| rs7634476 | A | G | -0.0116 | 0.009 | 2.09E-01 |
| rs7647398 | C | T | 0.0059 | 0.011 | 5.96E-01 |
| rs76608582 | C | A | 0.0168 | 0.026 | 5.20E-01 |
| rs77835879 | A | G | 0.0047 | 0.016 | 7.71E-01 |
| rs778371 | A | G | 0.0199 | 0.010 | 4.42E-02 |
| rs7798283 | T | G | 0.0186 | 0.013 | 1.64E-01 |
| rs7803932 | G | A | 0.0217 | 0.013 | 8.74E-02 |
| rs7808399 | A | G | -0.0061 | 0.009 | 4.99E-01 |
| rs78257128 | C | T | -0.0152 | 0.013 | 2.35E-01 |
| rs78721320 | G | A | -0.0273 | 0.012 | 2.27E-02 |
| rs7896518 | A | G | 0.0100 | 0.009 | 2.65E-01 |
| rs79265434 | A | G | -0.0262 | 0.014 | 6.24E-02 |
| rs79269403 | G | A | -0.0031 | 0.011 | 7.77E-01 |
| rs79445414 | T | C | -0.0510 | 0.021 | 1.64E-02 |
| rs795230 | C | T | 0.0118 | 0.009 | 1.90E-01 |
| rs7993663 | T | C | 0.0189 | 0.009 | 4.31E-02 |
| rs80171383 | G | A | 0.0001 | 0.013 | 9.96E-01 |
| rs8055219 | G | A | -0.0335 | 0.011 | 1.56E-03 |
| rs818415 | T | G | 0.0014 | 0.011 | 9.02E-01 |
| rs837080 | T | C | 0.0007 | 0.009 | 9.40E-01 |
| rs892612 | A | C | -0.0068 | 0.012 | 5.87E-01 |
| rs894067 | G | A | 0.0104 | 0.009 | 2.55E-01 |
| rs9318627 | A | C | 0.0091 | 0.009 | 3.18E-01 |
| rs9342482 | G | T | 0.0000 | 0.010 | 9.97E-01 |
| rs9349956 | A | C | -0.0054 | 0.012 | 6.44E-01 |
| rs9384679 | C | T | 0.0143 | 0.009 | 1.27E-01 |
| rs9386787 | A | G | 0.0059 | 0.009 | 5.05E-01 |
| rs9436866 | A | C | 0.0175 | 0.015 | 2.50E-01 |
| rs9461916 | T | C | -0.0243 | 0.009 | 7.90E-03 |
| rs9556958 | C | T | -0.0110 | 0.009 | 2.19E-01 |
| rs9616906 | G | A | -0.0135 | 0.009 | 1.42E-01 |
| rs9636107 | A | G | -0.0187 | 0.009 | 3.64E-02 |
| rs9679654 | T | C | 0.0066 | 0.009 | 4.67E-01 |
| rs968050 | C | T | -0.0121 | 0.009 | 1.74E-01 |
| rs9704097 | C | A | -0.0038 | 0.009 | 6.71E-01 |
| rs9876421 | C | T | 0.0035 | 0.009 | 7.05E-01 |
| rs989532 | A | G | 0.0094 | 0.009 | 3.08E-01 |
| rs9914918 | G | A | 0.0141 | 0.010 | 1.56E-01 |
| rs9933256 | A | G | -0.0064 | 0.011 | 5.74E-01 |
| rs9995567 | G | A | 0.0062 | 0.009 | 5.04E-01 |

Abbreviations: GWAS, genome-wide association studies; MDD, major depressive disorder; MR, Mendelian randomisation; SE, standard error; SES, socioeconomic status; SNP, single nucleotide polymorphism; TDI, Townsend deprivation index.

## Table S14. Summary information on genetic instruments for MDD-schizophrenia MR analysis in MDD GWAS dataset.

| **SNP** | **Effect Allele** | **Alternate Allele** | **EAF** | **Beta** | **SE** | ***p* value** | ***R*^2^** | ***F* statistics** | **Overall *R*^2^** | **Mean *F* statistics** |
| --- | --- | --- | --- | --- | --- | --- | --- | --- | --- | --- |
| rs11135349 | A | C | 0.468 | -0.029 | 0.005 | 1.09E-09 | 0.0001 | 37.510 | 0.002 | 38.405 |
| rs11643192 | A | C | 0.404 | 0.027 | 0.005 | 3.36E-08 | 0.0001 | 30.367 |  |  |
| rs1226412 | T | C | 0.792 | 0.033 | 0.006 | 2.38E-08 | 0.0001 | 31.670 |  |  |
| rs12552 | A | G | 0.445 | 0.043 | 0.005 | 6.07E-19 | 0.0002 | 79.866 |  |  |
| rs12666117 | A | G | 0.472 | 0.027 | 0.005 | 1.35E-08 | 0.0001 | 32.588 |  |  |
| rs1354115 | A | C | 0.623 | 0.028 | 0.005 | 2.37E-08 | 0.0001 | 31.717 |  |  |
| rs1432639 | A | C | 0.626 | 0.039 | 0.005 | 4.55E-15 | 0.0002 | 60.839 |  |  |
| rs17727765 | T | C | 0.916 | -0.051 | 0.009 | 8.51E-09 | 0.0001 | 33.323 |  |  |
| rs1806153 | T | G | 0.224 | 0.036 | 0.006 | 1.18E-09 | 0.0001 | 37.439 |  |  |
| rs2389016 | T | C | 0.281 | 0.031 | 0.005 | 1.02E-08 | 0.0001 | 33.117 |  |  |
| rs34215985 | C | G | 0.236 | -0.037 | 0.006 | 3.13E-09 | 0.0001 | 35.048 |  |  |
| rs4904738 | T | C | 0.563 | -0.029 | 0.005 | 2.57E-09 | 0.0001 | 34.795 |  |  |
| rs7198928 | T | C | 0.619 | 0.028 | 0.005 | 1.00E-08 | 0.0001 | 32.269 |  |  |
| rs7430565 | A | G | 0.573 | -0.029 | 0.005 | 2.87E-09 | 0.0001 | 36.002 |  |  |
| rs76485002 | A | G | 0.970 | 0.109 | 0.018 | 1.60E-09 | 0.0001 | 36.469 |  |  |
| rs7856424 | T | C | 0.285 | -0.031 | 0.005 | 8.48E-09 | 0.0001 | 33.342 |  |  |
| rs8025231 | A | C | 0.564 | -0.034 | 0.005 | 2.36E-12 | 0.0001 | 49.873 |  |  |
| rs8063603 | A | G | 0.654 | -0.031 | 0.005 | 6.87E-09 | 0.0001 | 33.770 |  |  |
| rs915057 | A | G | 0.422 | -0.030 | 0.005 | 7.61E-10 | 0.0001 | 37.473 |  |  |
| rs9427672 | A | G | 0.242 | -0.032 | 0.006 | 3.12E-08 | 0.0001 | 30.630 |  |  |

Abbreviations: EAF, effect allele frequency; GWAS, genome-wide association studies; MDD, major depressive disorder; MR, Mendelian randomisation; SE, standard error; SNP, single nucleotide polymorphism.

## Table S15. Summary information on genetic instruments for MDD-schizophrenia MR analysis in schizophrenia GWAS dataset.

| **SNP** | **Effect Allele** | **Alternate Allele** | **EAF** | **Beta** | **SE** | ***p* value** |
| --- | --- | --- | --- | --- | --- | --- |
| rs11135349 | A | C | 0.465 | -0.003 | 0.009 | 0.729 |
| rs11643192 | A | C | 0.405 | 0.019 | 0.009 | 0.035 |
| rs1226412 | T | C | 0.789 | 0.021 | 0.011 | 0.052 |
| rs12552 | A | G | 0.437 | -0.008 | 0.009 | 0.360 |
| rs12666117 | A | G | 0.476 | 0.025 | 0.009 | 0.004 |
| rs1354115 | A | C | 0.623 | 0.009 | 0.009 | 0.295 |
| rs1432639 | A | C | 0.626 | 0.022 | 0.009 | 0.012 |
| rs17727765 | T | C | 0.920 | -0.003 | 0.017 | 0.876 |
| rs1806153 | T | G | 0.224 | 0.021 | 0.011 | 0.045 |
| rs2389016 | T | C | 0.279 | 0.015 | 0.010 | 0.123 |
| rs34215985 | C | G | 0.212 | -0.023 | 0.011 | 0.032 |
| rs4904738 | T | C | 0.553 | -0.015 | 0.009 | 0.075 |
| rs7198928 | T | C | 0.612 | 0.003 | 0.009 | 0.731 |
| rs7430565 | A | G | 0.578 | -0.002 | 0.009 | 0.783 |
| rs76485002 | A | G | 0.973 | 0.007 | 0.030 | 0.826 |
| rs7856424 | T | C | 0.288 | -0.017 | 0.010 | 0.067 |
| rs8025231 | A | C | 0.556 | -0.007 | 0.009 | 0.384 |
| rs8063603 | A | G | 0.654 | -0.014 | 0.009 | 0.142 |
| rs915057 | A | G | 0.424 | -0.001 | 0.009 | 0.910 |
| rs9427672 | A | G | 0.243 | 0.027 | 0.010 | 0.008 |

Abbreviations: EAF, effect allele frequency; GWAS, genome-wide association studies; MDD, major depressive disorder; MR, Mendelian randomisation; SE, standard error; SNP, single nucleotide polymorphism.

## Table S16. Summary information on genetic instruments for MDD-educational years MR analysis in MDD GWAS dataset.

| **SNP** | **Effect Allele** | **Alternate Allele** | **EAF** | **Beta** | **SE** | ***p* value** | ***R*^2^** | ***F* statistics** | **Overall *R*^2^** | **Mean *F* statistics** |
| --- | --- | --- | --- | --- | --- | --- | --- | --- | --- | --- |
| rs10149470 | A | G | 0.484 | -0.029 | 0.005 | 3.05E-09 | 0.0001 | 35.018 | 0.003 | 37.160 |
| rs10950398 | A | G | 0.408 | 0.027 | 0.005 | 2.55E-08 | 0.0001 | 31.494 |  |  |
| rs10959913 | T | G | 0.762 | 0.033 | 0.006 | 5.06E-09 | 0.0001 | 34.327 |  |  |
| rs11135349 | A | C | 0.468 | -0.029 | 0.005 | 1.09E-09 | 0.0001 | 37.510 |  |  |
| rs11643192 | A | C | 0.404 | 0.027 | 0.005 | 3.36E-08 | 0.0001 | 30.367 |  |  |
| rs11663393 | A | G | 0.459 | 0.028 | 0.005 | 1.65E-08 | 0.0001 | 32.188 |  |  |
| rs11682175 | T | C | 0.522 | -0.028 | 0.005 | 4.68E-09 | 0.0001 | 34.274 |  |  |
| rs1226412 | T | C | 0.792 | 0.033 | 0.006 | 2.38E-08 | 0.0001 | 31.670 |  |  |
| rs12552 | A | G | 0.445 | 0.043 | 0.005 | 6.07E-19 | 0.0002 | 79.866 |  |  |
| rs12666117 | A | G | 0.472 | 0.027 | 0.005 | 1.35E-08 | 0.0001 | 32.588 |  |  |
| rs12958048 | A | G | 0.334 | 0.034 | 0.005 | 3.61E-11 | 0.0001 | 43.929 |  |  |
| rs1354115 | A | C | 0.623 | 0.028 | 0.005 | 2.37E-08 | 0.0001 | 31.717 |  |  |
| rs1432639 | A | C | 0.626 | 0.039 | 0.005 | 4.55E-15 | 0.0002 | 60.839 |  |  |
| rs159963 | A | C | 0.556 | -0.027 | 0.005 | 3.19E-08 | 0.0001 | 30.365 |  |  |
| rs17727765 | T | C | 0.916 | -0.051 | 0.009 | 8.51E-09 | 0.0001 | 33.323 |  |  |
| rs1806153 | T | G | 0.224 | 0.036 | 0.006 | 1.18E-09 | 0.0001 | 37.439 |  |  |
| rs2005864 | T | C | 0.441 | 0.028 | 0.005 | 6.73E-09 | 0.0001 | 33.118 |  |  |
| rs2389016 | T | C | 0.281 | 0.031 | 0.005 | 1.02E-08 | 0.0001 | 33.117 |  |  |
| rs247910 | A | G | 0.535 | -0.032 | 0.005 | 1.07E-10 | 0.0001 | 41.329 |  |  |
| rs34215985 | C | G | 0.236 | -0.037 | 0.006 | 3.13E-09 | 0.0001 | 35.048 |  |  |
| rs4074723 | A | C | 0.407 | -0.027 | 0.005 | 3.12E-08 | 0.0001 | 30.365 |  |  |
| rs4904738 | T | C | 0.563 | -0.029 | 0.005 | 2.57E-09 | 0.0001 | 34.795 |  |  |
| rs5758265 | A | G | 0.284 | 0.031 | 0.005 | 7.55E-09 | 0.0001 | 32.965 |  |  |
| rs61867293 | T | C | 0.200 | -0.037 | 0.006 | 6.97E-10 | 0.0001 | 37.593 |  |  |
| rs6905391 | A | G | 0.140 | -0.044 | 0.007 | 1.35E-10 | 0.0001 | 41.214 |  |  |
| rs7198928 | T | C | 0.619 | 0.028 | 0.005 | 1.00E-08 | 0.0001 | 32.269 |  |  |
| rs7430565 | A | G | 0.573 | -0.029 | 0.005 | 2.87E-09 | 0.0001 | 36.002 |  |  |
| rs76485002 | A | G | 0.970 | 0.109 | 0.018 | 1.60E-09 | 0.0001 | 36.469 |  |  |
| rs7856424 | T | C | 0.285 | -0.031 | 0.005 | 8.48E-09 | 0.0001 | 33.342 |  |  |
| rs8025231 | A | C | 0.564 | -0.034 | 0.005 | 2.36E-12 | 0.0001 | 49.873 |  |  |
| rs8063603 | A | G | 0.654 | -0.031 | 0.005 | 6.87E-09 | 0.0001 | 33.770 |  |  |
| rs915057 | A | G | 0.422 | -0.030 | 0.005 | 7.61E-10 | 0.0001 | 37.473 |  |  |
| rs9427672 | A | G | 0.242 | -0.032 | 0.006 | 3.12E-08 | 0.0001 | 30.630 |  |  |

Abbreviations: EAF, effect allele frequency; GWAS, genome-wide association studies; MDD, major depressive disorder; MR, Mendelian randomisation; SE, standard error; SNP, single nucleotide polymorphism.

## Table S17. Summary information on genetic instruments for MDD-educational years MR analysis in educational years GWAS dataset.

| **SNP** | **Effect Allele** | **Alternate Allele** | **EAF** | **Beta** | **SE** | ***p* value** |
| --- | --- | --- | --- | --- | --- | --- |
| rs10149470 | A | G | 0.485 | -0.007 | 0.002 | 4.51E-05 |
| rs10950398 | A | G | 0.418 | -0.004 | 0.002 | 3.30E-02 |
| rs10959913 | T | G | 0.733 | -0.009 | 0.002 | 2.62E-06 |
| rs11135349 | A | C | 0.478 | -0.002 | 0.002 | 3.54E-01 |
| rs11643192 | A | C | 0.391 | 0.009 | 0.002 | 1.76E-07 |
| rs11663393 | A | G | 0.493 | -0.011 | 0.002 | 3.38E-11 |
| rs11682175 | T | C | 0.553 | -0.009 | 0.002 | 3.21E-08 |
| rs1226412 | T | C | 0.793 | 0.002 | 0.002 | 2.61E-01 |
| rs12552 | A | G | 0.449 | 0.008 | 0.002 | 1.03E-05 |
| rs12666117 | A | G | 0.454 | -0.005 | 0.002 | 2.85E-03 |
| rs12958048 | A | G | 0.366 | -0.001 | 0.002 | 6.32E-01 |
| rs1354115 | A | C | 0.640 | -0.002 | 0.002 | 1.71E-01 |
| rs1432639 | A | C | 0.633 | 0.014 | 0.002 | 9.17E-16 |
| rs159963 | A | C | 0.534 | -0.004 | 0.002 | 2.32E-02 |
| rs17727765 | T | C | 0.920 | 0.008 | 0.003 | 1.10E-02 |
| rs1806153 | T | G | 0.194 | -0.001 | 0.002 | 5.54E-01 |
| rs2005864 | T | C | 0.469 | -0.003 | 0.002 | 1.27E-01 |
| rs2389016 | T | C | 0.320 | -0.006 | 0.002 | 3.11E-03 |
| rs247910 | A | G | 0.539 | -0.011 | 0.002 | 6.08E-11 |
| rs34215985 | C | G | 0.218 | -0.008 | 0.002 | 2.16E-04 |
| rs4074723 | A | C | 0.425 | -0.004 | 0.002 | 3.52E-02 |
| rs4904738 | T | C | 0.549 | 0.005 | 0.002 | 2.23E-03 |
| rs5758265 | A | G | 0.313 | 0.001 | 0.002 | 6.04E-01 |
| rs61867293 | T | C | 0.201 | 0.006 | 0.002 | 2.53E-03 |
| rs6905391 | A | G | 0.129 | -0.002 | 0.002 | 3.35E-01 |
| rs7198928 | T | C | 0.604 | 0.010 | 0.002 | 2.82E-09 |
| rs7430565 | A | G | 0.573 | 0.002 | 0.002 | 2.39E-01 |
| rs76485002 | A | G | 0.974 | -0.004 | 0.005 | 4.74E-01 |
| rs7856424 | T | C | 0.284 | 0.006 | 0.002 | 2.59E-03 |
| rs8025231 | A | C | 0.536 | 0.005 | 0.002 | 4.97E-03 |
| rs8063603 | A | G | 0.667 | -0.002 | 0.002 | 2.78E-01 |
| rs915057 | A | G | 0.410 | -0.009 | 0.002 | 1.47E-07 |
| rs9427672 | A | G | 0.209 | -0.010 | 0.002 | 1.17E-06 |

Abbreviations: EAF, effect allele frequency; GWAS, genome-wide association studies; MDD, major depressive disorder; MR, Mendelian randomisation; SE, standard error; SNP, single nucleotide polymorphism.

## Table S18. Summary information on genetic instruments for MDD-income MR analysis in MDD GWAS dataset.

| **SNP** | **Effect Allele** | **Alternate Allele** | **EAF** | **Beta** | **SE** | ***p* value** | ***R*^2^** | ***F* statistics** | **Overall *R*^2^** | **Mean *F* statistics** |
| --- | --- | --- | --- | --- | --- | --- | --- | --- | --- | --- |
| rs10149470 | A | G | 0.484 | -0.029 | 0.005 | 3.05E-09 | 0.0001 | 35.018 | 0.003 | 37.160 |
| rs10950398 | A | G | 0.408 | 0.027 | 0.005 | 2.55E-08 | 0.0001 | 31.494 |  |  |
| rs10959913 | T | G | 0.762 | 0.033 | 0.006 | 5.06E-09 | 0.0001 | 34.327 |  |  |
| rs11135349 | A | C | 0.468 | -0.029 | 0.005 | 1.09E-09 | 0.0001 | 37.510 |  |  |
| rs11643192 | A | C | 0.404 | 0.027 | 0.005 | 3.36E-08 | 0.0001 | 30.367 |  |  |
| rs11663393 | A | G | 0.459 | 0.028 | 0.005 | 1.65E-08 | 0.0001 | 32.188 |  |  |
| rs11682175 | T | C | 0.522 | -0.028 | 0.005 | 4.68E-09 | 0.0001 | 34.274 |  |  |
| rs1226412 | T | C | 0.792 | 0.033 | 0.006 | 2.38E-08 | 0.0001 | 31.670 |  |  |
| rs12552 | A | G | 0.445 | 0.043 | 0.005 | 6.07E-19 | 0.0002 | 79.866 |  |  |
| rs12666117 | A | G | 0.472 | 0.027 | 0.005 | 1.35E-08 | 0.0001 | 32.588 |  |  |
| rs12958048 | A | G | 0.334 | 0.034 | 0.005 | 3.61E-11 | 0.0001 | 43.929 |  |  |
| rs1354115 | A | C | 0.623 | 0.028 | 0.005 | 2.37E-08 | 0.0001 | 31.717 |  |  |
| rs1432639 | A | C | 0.626 | 0.039 | 0.005 | 4.55E-15 | 0.0002 | 60.839 |  |  |
| rs159963 | A | C | 0.556 | -0.027 | 0.005 | 3.19E-08 | 0.0001 | 30.365 |  |  |
| rs17727765 | T | C | 0.916 | -0.051 | 0.009 | 8.51E-09 | 0.0001 | 33.323 |  |  |
| rs1806153 | T | G | 0.224 | 0.036 | 0.006 | 1.18E-09 | 0.0001 | 37.439 |  |  |
| rs2005864 | T | C | 0.441 | 0.028 | 0.005 | 6.73E-09 | 0.0001 | 33.118 |  |  |
| rs2389016 | T | C | 0.281 | 0.031 | 0.005 | 1.02E-08 | 0.0001 | 33.117 |  |  |
| rs247910 | A | G | 0.535 | -0.032 | 0.005 | 1.07E-10 | 0.0001 | 41.329 |  |  |
| rs34215985 | C | G | 0.236 | -0.037 | 0.006 | 3.13E-09 | 0.0001 | 35.048 |  |  |
| rs4074723 | A | C | 0.407 | -0.027 | 0.005 | 3.12E-08 | 0.0001 | 30.365 |  |  |
| rs4904738 | T | C | 0.563 | -0.029 | 0.005 | 2.57E-09 | 0.0001 | 34.795 |  |  |
| rs5758265 | A | G | 0.284 | 0.031 | 0.005 | 7.55E-09 | 0.0001 | 32.965 |  |  |
| rs61867293 | T | C | 0.200 | -0.037 | 0.006 | 6.97E-10 | 0.0001 | 37.593 |  |  |
| rs6905391 | A | G | 0.140 | -0.044 | 0.007 | 1.35E-10 | 0.0001 | 41.214 |  |  |
| rs7198928 | T | C | 0.619 | 0.028 | 0.005 | 1.00E-08 | 0.0001 | 32.269 |  |  |
| rs7430565 | A | G | 0.573 | -0.029 | 0.005 | 2.87E-09 | 0.0001 | 36.002 |  |  |
| rs76485002 | A | G | 0.970 | 0.109 | 0.018 | 1.60E-09 | 0.0001 | 36.469 |  |  |
| rs7856424 | T | C | 0.285 | -0.031 | 0.005 | 8.48E-09 | 0.0001 | 33.342 |  |  |
| rs8025231 | A | C | 0.564 | -0.034 | 0.005 | 2.36E-12 | 0.0001 | 49.873 |  |  |
| rs8063603 | A | G | 0.654 | -0.031 | 0.005 | 6.87E-09 | 0.0001 | 33.770 |  |  |
| rs915057 | A | G | 0.422 | -0.030 | 0.005 | 7.61E-10 | 0.0001 | 37.473 |  |  |
| rs9427672 | A | G | 0.242 | -0.032 | 0.006 | 3.12E-08 | 0.0001 | 30.630 |  |  |

Abbreviations: EAF, effect allele frequency; GWAS, genome-wide association studies; MDD, major depressive disorder; MR, Mendelian randomisation; SE, standard error; SNP, single nucleotide polymorphism.

## Table S19. Summary information on genetic instruments for MDD-income MR analysis in income GWAS dataset.

| **SNP** | **Effect Allele** | **Alternate Allele** | **EAF** | **Beta** | **SE** | ***p* value** |
| --- | --- | --- | --- | --- | --- | --- |
| rs10149470 | A | G | 0.485 | 0.000 | 0.003 | 9.30E-01 |
| rs10950398 | A | G | 0.419 | -0.009 | 0.003 | 7.40E-04 |
| rs10959913 | T | G | 0.742 | -0.009 | 0.003 | 4.40E-03 |
| rs11135349 | A | C | 0.467 | -0.005 | 0.003 | 7.10E-02 |
| rs11643192 | A | C | 0.390 | 0.007 | 0.003 | 1.40E-02 |
| rs11663393 | A | G | 0.460 | -0.012 | 0.003 | 3.10E-06 |
| rs11682175 | T | C | 0.526 | -0.002 | 0.003 | 4.30E-01 |
| rs1226412 | T | C | 0.795 | -0.005 | 0.003 | 1.10E-01 |
| rs12552 | A | G | 0.437 | 0.006 | 0.003 | 3.70E-02 |
| rs12666117 | A | G | 0.465 | -0.003 | 0.003 | 3.00E-01 |
| rs12958048 | A | G | 0.335 | -0.004 | 0.003 | 1.60E-01 |
| rs1354115 | A | C | 0.630 | 0.000 | 0.003 | 9.80E-01 |
| rs1432639 | A | C | 0.604 | 0.009 | 0.003 | 1.40E-03 |
| rs159963 | A | C | 0.582 | -0.007 | 0.003 | 6.20E-03 |
| rs17727765 | T | C | 0.919 | -0.004 | 0.005 | 4.00E-01 |
| rs1806153 | T | G | 0.231 | -0.007 | 0.003 | 1.80E-02 |
| rs2005864 | T | C | 0.437 | -0.006 | 0.003 | 1.70E-02 |
| rs2389016 | T | C | 0.294 | -0.006 | 0.003 | 4.50E-02 |
| rs247910 | A | G | 0.538 | -0.003 | 0.003 | 2.20E-01 |
| rs34215985 | C | G | 0.208 | 0.002 | 0.003 | 5.80E-01 |
| rs4074723 | A | C | 0.411 | -0.001 | 0.003 | 6.70E-01 |
| rs4904738 | T | C | 0.571 | 0.004 | 0.003 | 1.70E-01 |
| rs5758265 | A | G | 0.287 | 0.005 | 0.003 | 6.30E-02 |
| rs61867293 | T | C | 0.193 | 0.016 | 0.003 | 9.30E-07 |
| rs6905391 | A | G | 0.162 | 0.001 | 0.004 | 7.40E-01 |
| rs7198928 | T | C | 0.615 | 0.005 | 0.003 | 7.50E-02 |
| rs7430565 | A | G | 0.585 | 0.002 | 0.003 | 5.40E-01 |
| rs76485002 | A | G | 0.970 | 0.011 | 0.008 | 1.80E-01 |
| rs7856424 | T | C | 0.284 | 0.007 | 0.003 | 1.80E-02 |
| rs8025231 | A | C | 0.554 | 0.010 | 0.003 | 1.40E-04 |
| rs8063603 | A | G | 0.677 | 0.001 | 0.003 | 7.00E-01 |
| rs915057 | A | G | 0.432 | -0.008 | 0.003 | 2.90E-03 |
| rs9427672 | A | G | 0.240 | -0.008 | 0.003 | 1.40E-02 |

Abbreviations: EAF, effect allele frequency; GWAS, genome-wide association studies; MDD, major depressive disorder; MR, Mendelian randomisation; SE, standard error; SNP, single nucleotide polymorphism.

## Table S20. Summary information on genetic instruments for MDD-employment status MR analysis in MDD GWAS dataset.

| **SNP** | **Effect Allele** | **Alternate Allele** | **EAF** | **Beta** | **SE** | ***p* value** | ***R*^2^** | ***F* statistics** | **Overall *R*^2^** | **Mean *F* statistics** |
| --- | --- | --- | --- | --- | --- | --- | --- | --- | --- | --- |
| rs10149470 | A | G | 0.484 | -0.029 | 0.005 | 3.05E-09 | 0.0001 | 35.018 | 0.003 | 37.160 |
| rs10950398 | A | G | 0.408 | 0.027 | 0.005 | 2.55E-08 | 0.0001 | 31.494 |  |  |
| rs10959913 | T | G | 0.762 | 0.033 | 0.006 | 5.06E-09 | 0.0001 | 34.327 |  |  |
| rs11135349 | A | C | 0.468 | -0.029 | 0.005 | 1.09E-09 | 0.0001 | 37.510 |  |  |
| rs11643192 | A | C | 0.404 | 0.027 | 0.005 | 3.36E-08 | 0.0001 | 30.367 |  |  |
| rs11663393 | A | G | 0.459 | 0.028 | 0.005 | 1.65E-08 | 0.0001 | 32.188 |  |  |
| rs11682175 | T | C | 0.522 | -0.028 | 0.005 | 4.68E-09 | 0.0001 | 34.274 |  |  |
| rs1226412 | T | C | 0.792 | 0.033 | 0.006 | 2.38E-08 | 0.0001 | 31.670 |  |  |
| rs12552 | A | G | 0.445 | 0.043 | 0.005 | 6.07E-19 | 0.0002 | 79.866 |  |  |
| rs12666117 | A | G | 0.472 | 0.027 | 0.005 | 1.35E-08 | 0.0001 | 32.588 |  |  |
| rs12958048 | A | G | 0.334 | 0.034 | 0.005 | 3.61E-11 | 0.0001 | 43.929 |  |  |
| rs1354115 | A | C | 0.623 | 0.028 | 0.005 | 2.37E-08 | 0.0001 | 31.717 |  |  |
| rs1432639 | A | C | 0.626 | 0.039 | 0.005 | 4.55E-15 | 0.0002 | 60.839 |  |  |
| rs159963 | A | C | 0.556 | -0.027 | 0.005 | 3.19E-08 | 0.0001 | 30.365 |  |  |
| rs17727765 | T | C | 0.916 | -0.051 | 0.009 | 8.51E-09 | 0.0001 | 33.323 |  |  |
| rs1806153 | T | G | 0.224 | 0.036 | 0.006 | 1.18E-09 | 0.0001 | 37.439 |  |  |
| rs2005864 | T | C | 0.441 | 0.028 | 0.005 | 6.73E-09 | 0.0001 | 33.118 |  |  |
| rs2389016 | T | C | 0.281 | 0.031 | 0.005 | 1.02E-08 | 0.0001 | 33.117 |  |  |
| rs247910 | A | G | 0.535 | -0.032 | 0.005 | 1.07E-10 | 0.0001 | 41.329 |  |  |
| rs34215985 | C | G | 0.236 | -0.037 | 0.006 | 3.13E-09 | 0.0001 | 35.048 |  |  |
| rs4074723 | A | C | 0.407 | -0.027 | 0.005 | 3.12E-08 | 0.0001 | 30.365 |  |  |
| rs4904738 | T | C | 0.563 | -0.029 | 0.005 | 2.57E-09 | 0.0001 | 34.795 |  |  |
| rs5758265 | A | G | 0.284 | 0.031 | 0.005 | 7.55E-09 | 0.0001 | 32.965 |  |  |
| rs61867293 | T | C | 0.200 | -0.037 | 0.006 | 6.97E-10 | 0.0001 | 37.593 |  |  |
| rs6905391 | A | G | 0.140 | -0.044 | 0.007 | 1.35E-10 | 0.0001 | 41.214 |  |  |
| rs7198928 | T | C | 0.619 | 0.028 | 0.005 | 1.00E-08 | 0.0001 | 32.269 |  |  |
| rs7430565 | A | G | 0.573 | -0.029 | 0.005 | 2.87E-09 | 0.0001 | 36.002 |  |  |
| rs76485002 | A | G | 0.970 | 0.109 | 0.018 | 1.60E-09 | 0.0001 | 36.469 |  |  |
| rs7856424 | T | C | 0.285 | -0.031 | 0.005 | 8.48E-09 | 0.0001 | 33.342 |  |  |
| rs8025231 | A | C | 0.564 | -0.034 | 0.005 | 2.36E-12 | 0.0001 | 49.873 |  |  |
| rs8063603 | A | G | 0.654 | -0.031 | 0.005 | 6.87E-09 | 0.0001 | 33.770 |  |  |
| rs915057 | A | G | 0.422 | -0.030 | 0.005 | 7.61E-10 | 0.0001 | 37.473 |  |  |
| rs9427672 | A | G | 0.242 | -0.032 | 0.006 | 3.12E-08 | 0.0001 | 30.630 |  |  |

Abbreviations: EAF, effect allele frequency; GWAS, genome-wide association studies; MDD, major depressive disorder; MR, Mendelian randomisation; SE, standard error; SNP, single nucleotide polymorphism.

## Table S21. Summary information on genetic instruments for MDD-employment status MR analysis in employment status GWAS dataset.

| **SNP** | **Effect Allele** | **Alternate Allele** | **EAF** | **Beta** | **SE** | ***p* value** |
| --- | --- | --- | --- | --- | --- | --- |
| rs10149470 | A | G | 0.486 | -0.002 | 0.001 | 0.140 |
| rs10950398 | A | G | 0.415 | -0.002 | 0.001 | 0.031 |
| rs10959913 | T | G | 0.742 | -0.001 | 0.001 | 0.460 |
| rs11135349 | A | C | 0.467 | 0.000 | 0.001 | 0.900 |
| rs11643192 | A | C | 0.390 | 0.001 | 0.001 | 0.490 |
| rs11663393 | A | G | 0.460 | -0.002 | 0.001 | 0.084 |
| rs11682175 | T | C | 0.527 | 0.004 | 0.001 | 0.001 |
| rs1226412 | T | C | 0.795 | -0.003 | 0.001 | 0.035 |
| rs12552 | A | G | 0.437 | 0.000 | 0.001 | 0.920 |
| rs12666117 | A | G | 0.466 | 0.001 | 0.001 | 0.540 |
| rs12958048 | A | G | 0.335 | 0.000 | 0.001 | 0.930 |
| rs1354115 | A | C | 0.630 | 0.001 | 0.001 | 0.270 |
| rs1432639 | A | C | 0.603 | 0.002 | 0.001 | 0.039 |
| rs159963 | A | C | 0.582 | -0.001 | 0.001 | 0.410 |
| rs17727765 | T | C | 0.919 | 0.000 | 0.002 | 0.830 |
| rs1806153 | T | G | 0.231 | -0.001 | 0.001 | 0.400 |
| rs2005864 | T | C | 0.437 | 0.000 | 0.001 | 0.810 |
| rs2389016 | T | C | 0.294 | -0.001 | 0.001 | 0.260 |
| rs247910 | A | G | 0.533 | 0.003 | 0.001 | 0.002 |
| rs34215985 | C | G | 0.208 | 0.001 | 0.001 | 0.690 |
| rs4074723 | A | C | 0.411 | -0.001 | 0.001 | 0.610 |
| rs4904738 | T | C | 0.571 | 0.000 | 0.001 | 0.680 |
| rs5758265 | A | G | 0.287 | -0.001 | 0.001 | 0.310 |
| rs61867293 | T | C | 0.193 | 0.002 | 0.001 | 0.160 |
| rs6905391 | A | G | 0.162 | -0.001 | 0.001 | 0.660 |
| rs7198928 | T | C | 0.615 | 0.001 | 0.001 | 0.480 |
| rs7430565 | A | G | 0.584 | 0.000 | 0.001 | 0.690 |
| rs76485002 | A | G | 0.970 | -0.001 | 0.003 | 0.800 |
| rs7856424 | T | C | 0.284 | 0.002 | 0.001 | 0.110 |
| rs8025231 | A | C | 0.554 | 0.003 | 0.001 | 0.006 |
| rs8063603 | A | G | 0.676 | -0.001 | 0.001 | 0.370 |
| rs915057 | A | G | 0.433 | -0.001 | 0.001 | 0.220 |
| rs9427672 | A | G | 0.240 | -0.001 | 0.001 | 0.350 |

Abbreviations: EAF, effect allele frequency; GWAS, genome-wide association studies; MDD, major depressive disorder; MR, Mendelian randomisation; SE, standard error; SNP, single nucleotide polymorphism.

## Table S22. Summary information on genetic instruments for MDD-TDI MR analysis in MDD GWAS dataset.

| **SNP** | **Effect Allele** | **Alternate Allele** | **EAF** | **Beta** | **SE** | ***p* value** | ***R*^2^** | ***F* statistics** | **Overall *R*^2^** | **Mean *F* statistics** |
| --- | --- | --- | --- | --- | --- | --- | --- | --- | --- | --- |
| rs10149470 | A | G | 0.484 | -0.029 | 0.005 | 3.05E-09 | 0.0001 | 35.018 | 0.003 | 37.160 |
| rs10950398 | A | G | 0.408 | 0.027 | 0.005 | 2.55E-08 | 0.0001 | 31.494 |  |  |
| rs10959913 | T | G | 0.762 | 0.033 | 0.006 | 5.06E-09 | 0.0001 | 34.327 |  |  |
| rs11135349 | A | C | 0.468 | -0.029 | 0.005 | 1.09E-09 | 0.0001 | 37.510 |  |  |
| rs11643192 | A | C | 0.404 | 0.027 | 0.005 | 3.36E-08 | 0.0001 | 30.367 |  |  |
| rs11663393 | A | G | 0.459 | 0.028 | 0.005 | 1.65E-08 | 0.0001 | 32.188 |  |  |
| rs11682175 | T | C | 0.522 | -0.028 | 0.005 | 4.68E-09 | 0.0001 | 34.274 |  |  |
| rs1226412 | T | C | 0.792 | 0.033 | 0.006 | 2.38E-08 | 0.0001 | 31.670 |  |  |
| rs12552 | A | G | 0.445 | 0.043 | 0.005 | 6.07E-19 | 0.0002 | 79.866 |  |  |
| rs12666117 | A | G | 0.472 | 0.027 | 0.005 | 1.35E-08 | 0.0001 | 32.588 |  |  |
| rs12958048 | A | G | 0.334 | 0.034 | 0.005 | 3.61E-11 | 0.0001 | 43.929 |  |  |
| rs1354115 | A | C | 0.623 | 0.028 | 0.005 | 2.37E-08 | 0.0001 | 31.717 |  |  |
| rs1432639 | A | C | 0.626 | 0.039 | 0.005 | 4.55E-15 | 0.0002 | 60.839 |  |  |
| rs159963 | A | C | 0.556 | -0.027 | 0.005 | 3.19E-08 | 0.0001 | 30.365 |  |  |
| rs17727765 | T | C | 0.916 | -0.051 | 0.009 | 8.51E-09 | 0.0001 | 33.323 |  |  |
| rs1806153 | T | G | 0.224 | 0.036 | 0.006 | 1.18E-09 | 0.0001 | 37.439 |  |  |
| rs2005864 | T | C | 0.441 | 0.028 | 0.005 | 6.73E-09 | 0.0001 | 33.118 |  |  |
| rs2389016 | T | C | 0.281 | 0.031 | 0.005 | 1.02E-08 | 0.0001 | 33.117 |  |  |
| rs247910 | A | G | 0.535 | -0.032 | 0.005 | 1.07E-10 | 0.0001 | 41.329 |  |  |
| rs34215985 | C | G | 0.236 | -0.037 | 0.006 | 3.13E-09 | 0.0001 | 35.048 |  |  |
| rs4074723 | A | C | 0.407 | -0.027 | 0.005 | 3.12E-08 | 0.0001 | 30.365 |  |  |
| rs4904738 | T | C | 0.563 | -0.029 | 0.005 | 2.57E-09 | 0.0001 | 34.795 |  |  |
| rs5758265 | A | G | 0.284 | 0.031 | 0.005 | 7.55E-09 | 0.0001 | 32.965 |  |  |
| rs61867293 | T | C | 0.200 | -0.037 | 0.006 | 6.97E-10 | 0.0001 | 37.593 |  |  |
| rs6905391 | A | G | 0.140 | -0.044 | 0.007 | 1.35E-10 | 0.0001 | 41.214 |  |  |
| rs7198928 | T | C | 0.619 | 0.028 | 0.005 | 1.00E-08 | 0.0001 | 32.269 |  |  |
| rs7430565 | A | G | 0.573 | -0.029 | 0.005 | 2.87E-09 | 0.0001 | 36.002 |  |  |
| rs76485002 | A | G | 0.970 | 0.109 | 0.018 | 1.60E-09 | 0.0001 | 36.469 |  |  |
| rs7856424 | T | C | 0.285 | -0.031 | 0.005 | 8.48E-09 | 0.0001 | 33.342 |  |  |
| rs8025231 | A | C | 0.564 | -0.034 | 0.005 | 2.36E-12 | 0.0001 | 49.873 |  |  |
| rs8063603 | A | G | 0.654 | -0.031 | 0.005 | 6.87E-09 | 0.0001 | 33.770 |  |  |
| rs915057 | A | G | 0.422 | -0.030 | 0.005 | 7.61E-10 | 0.0001 | 37.473 |  |  |
| rs9427672 | A | G | 0.242 | -0.032 | 0.006 | 3.12E-08 | 0.0001 | 30.630 |  |  |

Abbreviations: EAF, effect allele frequency; GWAS, genome-wide association studies; MDD, major depressive disorder; MR, Mendelian randomisation; SE, standard error; SNP, single nucleotide polymorphism; TDI, Townsend deprivation index.

## Table S23. Summary information on genetic instruments for MDD-TDI MR analysis in TDI GWAS dataset.

| **SNP** | **Effect Allele** | **Alternate Allele** | **EAF** | **Beta** | **SE** | ***p* value** |
| --- | --- | --- | --- | --- | --- | --- |
| rs10149470 | A | G | 0.486 | -0.004 | 0.002 | 0.045 |
| rs10950398 | A | G | 0.419 | 0.001 | 0.002 | 0.670 |
| rs10959913 | T | G | 0.742 | 0.002 | 0.002 | 0.370 |
| rs11135349 | A | C | 0.467 | 0.005 | 0.002 | 0.009 |
| rs11643192 | A | C | 0.390 | 0.005 | 0.002 | 0.026 |
| rs11663393 | A | G | 0.460 | 0.001 | 0.002 | 0.760 |
| rs11682175 | T | C | 0.527 | 0.003 | 0.002 | 0.150 |
| rs1226412 | T | C | 0.795 | -0.003 | 0.003 | 0.310 |
| rs12552 | A | G | 0.437 | 0.002 | 0.002 | 0.360 |
| rs12666117 | A | G | 0.465 | 0.004 | 0.002 | 0.030 |
| rs12958048 | A | G | 0.335 | 0.007 | 0.002 | 0.002 |
| rs1354115 | A | C | 0.630 | 0.006 | 0.002 | 0.007 |
| rs1432639 | A | C | 0.603 | 0.001 | 0.002 | 0.570 |
| rs159963 | A | C | 0.582 | 0.004 | 0.002 | 0.073 |
| rs17727765 | T | C | 0.919 | 0.004 | 0.004 | 0.300 |
| rs1806153 | T | G | 0.231 | 0.008 | 0.002 | 0.001 |
| rs2005864 | T | C | 0.437 | 0.006 | 0.002 | 0.007 |
| rs2389016 | T | C | 0.294 | 0.004 | 0.002 | 0.074 |
| rs247910 | A | G | 0.538 | 0.008 | 0.002 | 4.80E-05 |
| rs34215985 | C | G | 0.208 | 0.003 | 0.003 | 0.170 |
| rs4074723 | A | C | 0.411 | -0.004 | 0.002 | 0.056 |
| rs4904738 | T | C | 0.571 | -0.002 | 0.002 | 0.320 |
| rs5758265 | A | G | 0.287 | -0.004 | 0.002 | 0.093 |
| rs61867293 | T | C | 0.193 | -0.008 | 0.003 | 0.002 |
| rs6905391 | A | G | 0.162 | -0.008 | 0.003 | 0.003 |
| rs7198928 | T | C | 0.615 | -0.002 | 0.002 | 0.250 |
| rs7430565 | A | G | 0.584 | -0.002 | 0.002 | 0.410 |
| rs76485002 | A | G | 0.970 | -0.009 | 0.006 | 0.150 |
| rs7856424 | T | C | 0.284 | -0.001 | 0.002 | 0.620 |
| rs8025231 | A | C | 0.554 | -0.005 | 0.002 | 0.011 |
| rs8063603 | A | G | 0.676 | -0.009 | 0.002 | 3.50E-05 |
| rs915057 | A | G | 0.433 | 0.000 | 0.002 | 0.860 |
| rs9427672 | A | G | 0.240 | 0.006 | 0.002 | 0.010 |

Abbreviations: EAF, effect allele frequency; GWAS, genome-wide association studies; MDD, major depressive disorder; MR, Mendelian randomisation; SE, standard error; SNP, single nucleotide polymorphism; TDI, Townsend deprivation index.

## Table S24. *I*^2^ values for the evaluation of NOME assumption in MR analyses.

| **Exposure** | **Outcome** | ***I*^2^ value** |
| --- | --- | --- |
| Schizophrenia | MDD | 0.632 |
|  | Educational years | 0.630 |
|  | Household income | 0.630 |
|  | Employment status | 0.630 |
|  | TDI | 0.630 |
|  |  |  |
| MDD | Schizophrenia | 0.471 |
|  | Educational years | 0.293 |
|  | Household income | 0.293 |
|  | Employment status | 0.293 |
|  | TDI | 0.293 |

Note: We performed SIMEX corrections to replace the MR-Egger results for those *I*^2^ values less than 0.9.

Abbreviations: MDD, major depressive disorder; MR, Mendelian randomisation; NOME, no measurement error; SIMEX, simulation extrapolation; TDI, Townsend deprivation index.

## Table S25. Results for sensitivity analyses and Steiger tests in univariable MR analyses.

| **Exposure** | **Outcome** | **Heterogeneity test** | | | | **Pleiotropy test** | | | **Steiger test** | | | |
| --- | --- | --- | --- | --- | --- | --- | --- | --- | --- | --- | --- | --- |
|  |  | **Method** | ***Q*** | ***Q*_df** | ***P* value** | **Egger intercept** | **SE** | ***P* value** | ***r*^2^ (exposure)** | ***r*^2^ (outcome)** | **Correct causal direction** | ***P* value** |
| Schizophrenia | MDD | MR Egger | 323.113 | 144 | 8.83E-16 | -0.001 | 0.005 | 0.772 | 0.050 | 0.003 | TRUE | ~0 |
|  |  | Inverse variance weighted | 323.303 | 145 | 1.26E-15 |  |  |  |  |  |  |  |
|  | Educational years | MR Egger | 1327.047 | 145 | 6.17E-190 | -0.002 | 0.002 | 0.226 | 0.050 | 0.002 | TRUE | ~0 |
|  |  | Inverse variance weighted | 1340.598 | 146 | 4.43E-192 |  |  |  |  |  |  |  |
|  | Household income | MR Egger | 464.068 | 144 | 2.58E-35 | -0.002 | 0.002 | 0.284 | 0.050 | 0.002 | TRUE | ~0 |
|  |  | Inverse variance weighted | 467.788 | 145 | 1.28E-35 |  |  |  |  |  |  |  |
|  | Employment status | MR Egger | 216.608 | 144 | 8.67E-05 | 2.32E-04 | 4.65E-04 | 0.618 | 0.050 | 0.001 | TRUE | ~0 |
|  |  | Inverse variance weighted | 216.983 | 145 | 1.01E-04 |  |  |  |  |  |  |  |
|  | TDI | MR Egger | 356.192 | 144 | 5.48E-20 | 0.003 | 0.001 | 0.016 | 0.050 | 0.001 | TRUE | ~0 |
|  |  | Inverse variance weighted | 371.031 | 145 | 9.38E-22 |  |  |  |  |  |  |  |
|  |  |  |  |  |  |  |  |  |  |  |  |  |
| MDD | Schizophrenia | MR Egger | 31.073 | 18 | 0.028 | 0.015 | 0.013 | 0.242 | 0.002 | 4.02E-04 | TRUE | 5.24E-16 |
|  |  | Inverse variance weighted | 33.601 | 19 | 0.020 |  |  |  |  |  |  |  |
|  | Educational years | MR Egger | 431.544 | 31 | 4.36E-72 | 1.03E-04 | 0.006 | 0.986 | 0.003 | 0.001 | TRUE | 3.78E-64 |
|  |  | Inverse variance weighted | 431.548 | 32 | 1.64E-71 |  |  |  |  |  |  |  |
|  | Household income | MR Egger | 161.978 | 31 | 1.14E-19 | -0.004 | 0.006 | 0.478 | 0.003 | 4.19E-04 | TRUE | 2.80E-61 |
|  |  | Inverse variance weighted | 164.674 | 32 | 8.81E-20 |  |  |  |  |  |  |  |
|  | Employment status | MR Egger | 58.878 | 31 | 0.002 | -4.11E-04 | 0.001 | 0.753 | 0.003 | 1.36E-04 | TRUE | 2.53E-98 |
|  |  | Inverse variance weighted | 59.069 | 32 | 0.002 |  |  |  |  |  |  |  |
|  | TDI | MR Egger | 133.799 | 31 | 9.86E-15 | 0.002 | 0.004 | 0.565 | 0.003 | 3.19E-04 | TRUE | 1.27E-74 |
|  |  | Inverse variance weighted | 135.258 | 32 | 1.18E-14 |  |  |  |  |  |  |  |

Abbreviations: MDD, major depressive disorder; MR, Mendelian randomisation; SE, standard error; TDI, Townsend deprivation index.

## Table S26. MR leave-one-out sensitivity analysis for causal effect of schizophrenia on MDD.

| **Exposure** | **Outcome** | **SNP** | **Beta** | **SE** | ***p* value** |
| --- | --- | --- | --- | --- | --- |
| Schizophrenia | MDD | rs1000237 | 0.132 | 0.019 | 7.660E-12 |
|  |  | rs10035564 | 0.132 | 0.019 | 6.840E-12 |
|  |  | rs10086619 | 0.128 | 0.019 | 3.620E-11 |
|  |  | rs10108980 | 0.129 | 0.019 | 2.780E-11 |
|  |  | rs10117 | 0.129 | 0.019 | 2.460E-11 |
|  |  | rs10861176 | 0.130 | 0.019 | 1.370E-11 |
|  |  | rs10876446 | 0.127 | 0.019 | 4.330E-11 |
|  |  | rs11027839 | 0.128 | 0.019 | 3.240E-11 |
|  |  | rs11136325 | 0.128 | 0.019 | 3.970E-11 |
|  |  | rs11165867 | 0.129 | 0.019 | 2.190E-11 |
|  |  | rs11191580 | 0.126 | 0.019 | 6.920E-11 |
|  |  | rs11210892 | 0.129 | 0.019 | 2.750E-11 |
|  |  | rs11223774 | 0.131 | 0.019 | 4.180E-12 |
|  |  | rs113264400 | 0.127 | 0.019 | 3.900E-11 |
|  |  | rs11534045 | 0.129 | 0.019 | 2.590E-11 |
|  |  | rs11587347 | 0.127 | 0.019 | 4.450E-11 |
|  |  | rs11664298 | 0.126 | 0.019 | 5.840E-11 |
|  |  | rs11693094 | 0.128 | 0.019 | 3.540E-11 |
|  |  | rs117178087 | 0.130 | 0.019 | 1.970E-11 |
|  |  | rs11941714 | 0.128 | 0.019 | 3.870E-11 |
|  |  | rs1198588 | 0.127 | 0.019 | 6.640E-11 |
|  |  | rs12129573 | 0.123 | 0.019 | 8.420E-11 |
|  |  | rs12138231 | 0.129 | 0.019 | 2.890E-11 |
|  |  | rs12151767 | 0.128 | 0.019 | 4.260E-11 |
|  |  | rs12285419 | 0.128 | 0.019 | 4.000E-11 |
|  |  | rs12293670 | 0.129 | 0.019 | 3.370E-11 |
|  |  | rs12303743 | 0.130 | 0.019 | 2.000E-11 |
|  |  | rs12489270 | 0.130 | 0.019 | 2.060E-11 |
|  |  | rs12652777 | 0.129 | 0.019 | 2.600E-11 |
|  |  | rs12712510 | 0.127 | 0.019 | 4.920E-11 |
|  |  | rs12771371 | 0.129 | 0.019 | 2.260E-11 |
|  |  | rs12833624 | 0.128 | 0.019 | 3.330E-11 |
|  |  | rs12877581 | 0.129 | 0.019 | 2.150E-11 |
|  |  | rs12883788 | 0.129 | 0.019 | 2.850E-11 |
|  |  | rs13016542 | 0.130 | 0.019 | 1.690E-11 |
|  |  | rs13107325 | 0.132 | 0.019 | 6.810E-12 |
|  |  | rs13195636 | 0.123 | 0.019 | 2.280E-10 |
|  |  | rs13233308 | 0.129 | 0.019 | 2.420E-11 |
|  |  | rs132582 | 0.128 | 0.019 | 3.560E-11 |
|  |  | rs1427633 | 0.128 | 0.019 | 3.130E-11 |
|  |  | rs1430894 | 0.129 | 0.019 | 3.040E-11 |
|  |  | rs145071536 | 0.130 | 0.019 | 2.100E-11 |
|  |  | rs1451488 | 0.129 | 0.019 | 2.890E-11 |
|  |  | rs149165 | 0.130 | 0.019 | 1.370E-11 |
|  |  | rs1593304 | 0.129 | 0.019 | 2.850E-11 |
|  |  | rs1604060 | 0.127 | 0.019 | 4.310E-11 |
|  |  | rs1615350 | 0.132 | 0.019 | 3.880E-12 |
|  |  | rs167924 | 0.129 | 0.019 | 2.400E-11 |
|  |  | rs16851048 | 0.129 | 0.019 | 2.220E-11 |
|  |  | rs16867571 | 0.129 | 0.019 | 2.900E-11 |
|  |  | rs17016552 | 0.128 | 0.019 | 4.030E-11 |
|  |  | rs17194490 | 0.128 | 0.019 | 3.350E-11 |
|  |  | rs17731 | 0.129 | 0.019 | 2.350E-11 |
|  |  | rs187557 | 0.127 | 0.019 | 4.120E-11 |
|  |  | rs1881046 | 0.130 | 0.019 | 1.930E-11 |
|  |  | rs1901512 | 0.129 | 0.019 | 2.630E-11 |
|  |  | rs1915019 | 0.131 | 0.019 | 9.870E-12 |
|  |  | rs2053079 | 0.130 | 0.019 | 1.200E-11 |
|  |  | rs2078266 | 0.129 | 0.019 | 2.100E-11 |
|  |  | rs215412 | 0.129 | 0.019 | 2.480E-11 |
|  |  | rs217336 | 0.130 | 0.019 | 1.390E-11 |
|  |  | rs2238057 | 0.129 | 0.019 | 3.380E-11 |
|  |  | rs2252074 | 0.129 | 0.019 | 2.980E-11 |
|  |  | rs2332700 | 0.125 | 0.019 | 6.490E-11 |
|  |  | rs2333321 | 0.127 | 0.019 | 4.550E-11 |
|  |  | rs2381411 | 0.129 | 0.019 | 2.460E-11 |
|  |  | rs2455415 | 0.129 | 0.019 | 2.460E-11 |
|  |  | rs2456020 | 0.130 | 0.019 | 2.290E-11 |
|  |  | rs2514218 | 0.126 | 0.019 | 6.120E-11 |
|  |  | rs2710323 | 0.126 | 0.019 | 7.060E-11 |
|  |  | rs2815731 | 0.127 | 0.019 | 5.050E-11 |
|  |  | rs2909457 | 0.130 | 0.019 | 2.000E-11 |
|  |  | rs2999392 | 0.129 | 0.019 | 3.090E-11 |
|  |  | rs308697 | 0.131 | 0.019 | 1.000E-11 |
|  |  | rs35351411 | 0.129 | 0.019 | 2.830E-11 |
|  |  | rs35734242 | 0.129 | 0.019 | 2.740E-11 |
|  |  | rs3739118 | 0.127 | 0.019 | 4.660E-11 |
|  |  | rs3770754 | 0.129 | 0.019 | 3.000E-11 |
|  |  | rs3791710 | 0.130 | 0.019 | 1.140E-11 |
|  |  | rs3795310 | 0.132 | 0.019 | 2.300E-12 |
|  |  | rs3802924 | 0.128 | 0.019 | 3.550E-11 |
|  |  | rs3814883 | 0.127 | 0.019 | 4.800E-11 |
|  |  | rs3824451 | 0.128 | 0.019 | 3.150E-11 |
|  |  | rs4129585 | 0.128 | 0.019 | 3.780E-11 |
|  |  | rs4575535 | 0.127 | 0.019 | 4.500E-11 |
|  |  | rs4632195 | 0.126 | 0.019 | 4.070E-11 |
|  |  | rs4636654 | 0.127 | 0.019 | 4.120E-11 |
|  |  | rs4653164 | 0.126 | 0.019 | 4.050E-11 |
|  |  | rs4702 | 0.126 | 0.019 | 7.200E-11 |
|  |  | rs4766428 | 0.127 | 0.019 | 5.640E-11 |
|  |  | rs4779050 | 0.128 | 0.019 | 3.270E-11 |
|  |  | rs4812325 | 0.128 | 0.019 | 4.090E-11 |
|  |  | rs4921741 | 0.129 | 0.019 | 2.780E-11 |
|  |  | rs498591 | 0.127 | 0.019 | 4.390E-11 |
|  |  | rs500102 | 0.128 | 0.019 | 3.810E-11 |
|  |  | rs505061 | 0.127 | 0.019 | 4.630E-11 |
|  |  | rs56205728 | 0.128 | 0.019 | 3.280E-11 |
|  |  | rs56335113 | 0.127 | 0.019 | 4.780E-11 |
|  |  | rs57433322 | 0.130 | 0.019 | 1.860E-11 |
|  |  | rs5751191 | 0.129 | 0.019 | 3.150E-11 |
|  |  | rs58120505 | 0.133 | 0.019 | 5.940E-12 |
|  |  | rs60135207 | 0.128 | 0.019 | 3.150E-11 |
|  |  | rs6125656 | 0.127 | 0.019 | 4.140E-11 |
|  |  | rs61857878 | 0.129 | 0.019 | 2.990E-11 |
|  |  | rs61937595 | 0.130 | 0.019 | 1.670E-11 |
|  |  | rs62018952 | 0.126 | 0.019 | 4.840E-11 |
|  |  | rs62183855 | 0.129 | 0.019 | 2.420E-11 |
|  |  | rs634940 | 0.128 | 0.019 | 4.250E-11 |
|  |  | rs6482437 | 0.127 | 0.019 | 4.680E-11 |
|  |  | rs6520064 | 0.129 | 0.019 | 2.870E-11 |
|  |  | rs6538539 | 0.129 | 0.019 | 2.880E-11 |
|  |  | rs6546857 | 0.128 | 0.019 | 3.510E-11 |
|  |  | rs6549963 | 0.129 | 0.019 | 2.950E-11 |
|  |  | rs6673880 | 0.129 | 0.019 | 3.100E-11 |
|  |  | rs6715366 | 0.126 | 0.019 | 4.360E-11 |
|  |  | rs6798742 | 0.128 | 0.019 | 3.510E-11 |
|  |  | rs6943762 | 0.133 | 0.019 | 3.640E-12 |
|  |  | rs6974218 | 0.127 | 0.019 | 4.330E-11 |
|  |  | rs6984242 | 0.131 | 0.019 | 6.800E-12 |
|  |  | rs708228 | 0.126 | 0.019 | 4.560E-11 |
|  |  | rs7112616 | 0.130 | 0.019 | 1.820E-11 |
|  |  | rs713692 | 0.129 | 0.019 | 2.980E-11 |
|  |  | rs7251 | 0.129 | 0.019 | 2.540E-11 |
|  |  | rs72802868 | 0.130 | 0.019 | 1.840E-11 |
|  |  | rs728055 | 0.131 | 0.019 | 1.160E-11 |
|  |  | rs72943392 | 0.127 | 0.019 | 4.080E-11 |
|  |  | rs72986630 | 0.130 | 0.019 | 1.130E-11 |
|  |  | rs73229090 | 0.128 | 0.019 | 3.530E-11 |
|  |  | rs73292401 | 0.128 | 0.019 | 4.080E-11 |
|  |  | rs7515363 | 0.128 | 0.019 | 3.410E-11 |
|  |  | rs7575796 | 0.130 | 0.019 | 1.870E-11 |
|  |  | rs7634476 | 0.128 | 0.019 | 3.750E-11 |
|  |  | rs7647398 | 0.129 | 0.019 | 2.840E-11 |
|  |  | rs76838079 | 0.131 | 0.019 | 1.010E-11 |
|  |  | rs778371 | 0.133 | 0.019 | 3.960E-12 |
|  |  | rs7798283 | 0.128 | 0.019 | 3.750E-11 |
|  |  | rs7830315 | 0.127 | 0.019 | 4.390E-11 |
|  |  | rs79210963 | 0.128 | 0.019 | 4.220E-11 |
|  |  | rs79445414 | 0.127 | 0.019 | 4.570E-11 |
|  |  | rs8055219 | 0.126 | 0.019 | 5.180E-11 |
|  |  | rs9304548 | 0.129 | 0.019 | 3.120E-11 |
|  |  | rs9318627 | 0.128 | 0.019 | 3.460E-11 |
|  |  | rs9461916 | 0.127 | 0.019 | 4.650E-11 |
|  |  | rs9636107 | 0.127 | 0.019 | 5.230E-11 |
|  |  | rs9687282 | 0.130 | 0.019 | 1.130E-11 |
|  |  | rs9876421 | 0.130 | 0.019 | 1.800E-11 |
|  |  | **All** | 0.129 | 0.019 | 2.370E-11 |

Abbreviations: MDD, major depressive disorder; MR, Mendelian randomization; SE, standard error; SNP, single nucleotide polymorphism.

## Table S27. Univariable MR results with MDD as the exposure.

| **Outcome** | **Method** | **SNPs** | **Beta** | **SE** | **Lower CI** | **Upper CI** | **OR** | **Lower CI** | **Upper CI** | ***p* value** |
| --- | --- | --- | --- | --- | --- | --- | --- | --- | --- | --- |
| Schizophrenia | MR Egger (SIMEX) | 20 | -0.072 | 0.166 | -0.398 | 0.253 | 0.930 | 0.672 | 1.288 | 0.669 |
|  | Weighted median | 20 | 0.287 | 0.096 | 0.100 | 0.475 | 1.333 | 1.105 | 1.608 | 0.003 |
|  | Inverse variance weighted | 20 | 0.280 | 0.086 | 0.112 | 0.448 | 1.323 | 1.118 | 1.565 | 0.001 |
|  | MR-PRESSO outlier-corrected | 19 | 0.327 | 0.071 | 0.188 | 0.466 | 1.387 | 1.207 | 1.593 | 3.74E-06 |
|  |  |  |  |  |  |  |  |  |  |  |
| Educational years | MR Egger (SIMEX) | 33 | -0.064 | 0.088 | -0.236 | 0.109 | 0.938 | 0.790 | 1.115 | 0.475 |
|  | Weighted median | 33 | -0.002 | 0.023 | -0.048 | 0.043 | 0.998 | 0.954 | 1.044 | 0.918 |
|  | Inverse variance weighted | 33 | 0.045 | 0.037 | -0.027 | 0.117 | 1.046 | 0.974 | 1.125 | 0.216 |
|  | MR-PRESSO outlier-corrected | - | - | - | - | - | - | - | - | - |
|  |  |  |  |  |  |  |  |  |  |  |
| Household income | MR Egger (SIMEX) | 33 | 0.156 | 0.087 | -0.016 | 0.327 | 1.169 | 0.984 | 1.387 | 0.085 |
|  | Weighted median | 33 | -0.008 | 0.032 | -0.071 | 0.054 | 0.992 | 0.932 | 1.056 | 0.800 |
|  | Inverse variance weighted | 33 | -0.023 | 0.035 | -0.091 | 0.046 | 0.978 | 0.913 | 1.047 | 0.520 |
|  | MR-PRESSO outlier-corrected | - | - | - | - | - | - | - | - | - |
|  |  |  |  |  |  |  |  |  |  |  |
| Employment status | MR Egger (SIMEX) | 33 | -0.003 | 0.019 | -0.041 | 0.035 | 0.997 | 0.960 | 1.036 | 0.883 |
|  | Weighted median | 33 | -0.002 | 0.009 | -0.020 | 0.016 | 0.998 | 0.980 | 1.016 | 0.830 |
|  | Inverse variance weighted | 33 | -0.012 | 0.008 | -0.028 | 0.005 | 0.989 | 0.973 | 1.005 | 0.160 |
|  | MR-PRESSO outlier-corrected | - | - | - | - | - | - | - | - | - |
|  |  |  |  |  |  |  |  |  |  |  |
| TDI | MR Egger (SIMEX) | 33 | -0.123 | 0.063 | -0.248 | 0.001 | 0.884 | 0.781 | 1.001 | 0.061 |
|  | Weighted median | 33 | 0.040 | 0.023 | -0.005 | 0.085 | 1.041 | 0.995 | 1.089 | 0.082 |
|  | Inverse variance weighted | 33 | 0.041 | 0.024 | -0.007 | 0.089 | 1.042 | 0.993 | 1.093 | 0.091 |
|  | MR-PRESSO outlier-corrected | - | - | - | - | - | - | - | - | - |

Note: When investigating the causal effect of schizophrenia on education years, MR-PRESSO outlier correction was not performed due to the non-significant MR results found by other MR methods.

Abbreviations: CI, confidence interval; MDD, major depressive disorder; MR-PRESSO, Mendelian randomisation pleiotropy residual sum and outlier; OR, odds ratio; SE, standard error; SIMEX, simulation extrapolation; SNP, single nucleotide polymorphism; TDI, Townsend deprivation index.

## Table S28. MR leave-one-out sensitivity analysis for causal effect of MDD on schizophrenia.

| **Exposure** | **Outcome** | **SNP** | **Beta** | **SE** | ***p value*** |
| --- | --- | --- | --- | --- | --- |
| MDD | Schizophrenia | rs11135349 | 0.289 | 0.090 | 1.280E-03 |
|  |  | rs11643192 | 0.263 | 0.088 | 2.645E-03 |
|  |  | rs1226412 | 0.265 | 0.088 | 2.639E-03 |
|  |  | rs12552 | 0.332 | 0.084 | 8.150E-05 |
|  |  | rs12666117 | 0.252 | 0.084 | 2.731E-03 |
|  |  | rs1354115 | 0.277 | 0.090 | 1.998E-03 |
|  |  | rs1432639 | 0.255 | 0.089 | 4.353E-03 |
|  |  | rs17727765 | 0.289 | 0.089 | 1.172E-03 |
|  |  | rs1806153 | 0.264 | 0.089 | 2.906E-03 |
|  |  | rs2389016 | 0.271 | 0.089 | 2.424E-03 |
|  |  | rs34215985 | 0.262 | 0.088 | 3.061E-03 |
|  |  | rs4904738 | 0.268 | 0.089 | 2.668E-03 |
|  |  | rs7198928 | 0.288 | 0.089 | 1.315E-03 |
|  |  | rs7430565 | 0.289 | 0.089 | 1.230E-03 |
|  |  | rs76485002 | 0.292 | 0.090 | 1.101E-03 |
|  |  | rs7856424 | 0.267 | 0.089 | 2.634E-03 |
|  |  | rs8025231 | 0.284 | 0.091 | 1.785E-03 |
|  |  | rs8063603 | 0.272 | 0.090 | 2.410E-03 |
|  |  | rs915057 | 0.292 | 0.089 | 1.050E-03 |
|  |  | rs9427672 | 0.327 | 0.071 | 3.740E-06 |
|  |  | **All** | 0.280 | 0.086 | 1.088E-03 |

Abbreviations: MDD, major depressive disorder; MR, Mendelian randomization; SE, standard error; SNP, single nucleotide polymorphism.

## Table S29. MR leave-one-out sensitivity analysis for causal effect of schizophrenia on employment status.

| **Exposure** | **Outcome** | **SNP** | **Beta** | **SE** | ***p* value** |
| --- | --- | --- | --- | --- | --- |
| Schizophrenia | Employment status | rs1000237 | -0.008 | 0.002 | 7.743E-06 |
|  |  | rs10035564 | -0.008 | 0.002 | 2.304E-06 |
|  |  | rs10086619 | -0.008 | 0.002 | 5.013E-06 |
|  |  | rs10108980 | -0.008 | 0.002 | 3.071E-06 |
|  |  | rs10117 | -0.008 | 0.002 | 7.233E-06 |
|  |  | rs10861176 | -0.008 | 0.002 | 1.626E-06 |
|  |  | rs10873538 | -0.008 | 0.002 | 3.196E-06 |
|  |  | rs10876446 | -0.008 | 0.002 | 6.667E-06 |
|  |  | rs11027839 | -0.008 | 0.002 | 3.261E-06 |
|  |  | rs11136325 | -0.008 | 0.002 | 3.014E-06 |
|  |  | rs11165867 | -0.009 | 0.002 | 1.602E-06 |
|  |  | rs11191580 | -0.008 | 0.002 | 7.714E-06 |
|  |  | rs11210892 | -0.008 | 0.002 | 5.263E-06 |
|  |  | rs11223774 | -0.008 | 0.002 | 4.286E-06 |
|  |  | rs113264400 | -0.008 | 0.002 | 3.044E-06 |
|  |  | rs11534045 | -0.008 | 0.002 | 6.822E-06 |
|  |  | rs11587347 | -0.008 | 0.002 | 2.674E-06 |
|  |  | rs11664298 | -0.008 | 0.002 | 4.451E-06 |
|  |  | rs11693094 | -0.008 | 0.002 | 5.157E-06 |
|  |  | rs117178087 | -0.008 | 0.002 | 3.833E-06 |
|  |  | rs11941714 | -0.008 | 0.002 | 5.178E-06 |
|  |  | rs1198588 | -0.008 | 0.002 | 4.733E-06 |
|  |  | rs12129573 | -0.008 | 0.002 | 3.577E-06 |
|  |  | rs12138231 | -0.008 | 0.002 | 4.792E-06 |
|  |  | rs12151767 | -0.008 | 0.002 | 5.604E-06 |
|  |  | rs12285419 | -0.008 | 0.002 | 4.987E-06 |
|  |  | rs12293670 | -0.008 | 0.002 | 6.814E-06 |
|  |  | rs12303743 | -0.008 | 0.002 | 2.796E-06 |
|  |  | rs12489270 | -0.008 | 0.002 | 3.592E-06 |
|  |  | rs12652777 | -0.008 | 0.002 | 3.698E-06 |
|  |  | rs12712510 | -0.008 | 0.002 | 3.783E-06 |
|  |  | rs12771371 | -0.008 | 0.002 | 2.876E-06 |
|  |  | rs12833624 | -0.008 | 0.002 | 6.163E-06 |
|  |  | rs12877581 | -0.008 | 0.002 | 3.875E-06 |
|  |  | rs12883788 | -0.008 | 0.002 | 6.993E-06 |
|  |  | rs13016542 | -0.008 | 0.002 | 2.660E-06 |
|  |  | rs13107325 | -0.008 | 0.002 | 1.377E-05 |
|  |  | rs13195636 | -0.009 | 0.002 | 2.273E-06 |
|  |  | rs13233308 | -0.008 | 0.002 | 6.342E-06 |
|  |  | rs132582 | -0.008 | 0.002 | 3.452E-06 |
|  |  | rs1430894 | -0.008 | 0.002 | 4.432E-06 |
|  |  | rs145071536 | -0.008 | 0.002 | 5.902E-06 |
|  |  | rs1451488 | -0.008 | 0.002 | 4.779E-06 |
|  |  | rs149165 | -0.008 | 0.002 | 2.549E-06 |
|  |  | rs1593304 | -0.008 | 0.002 | 3.630E-06 |
|  |  | rs1604060 | -0.008 | 0.002 | 6.305E-06 |
|  |  | rs1615350 | -0.008 | 0.002 | 6.021E-06 |
|  |  | rs167924 | -0.008 | 0.002 | 5.392E-06 |
|  |  | rs16851048 | -0.008 | 0.002 | 3.972E-06 |
|  |  | rs16867571 | -0.008 | 0.002 | 5.062E-06 |
|  |  | rs17016552 | -0.008 | 0.002 | 5.130E-06 |
|  |  | rs17194490 | -0.008 | 0.002 | 3.661E-06 |
|  |  | rs17731 | -0.008 | 0.002 | 5.554E-06 |
|  |  | rs187557 | -0.008 | 0.002 | 4.534E-06 |
|  |  | rs1881046 | -0.008 | 0.002 | 6.241E-06 |
|  |  | rs1901512 | -0.008 | 0.002 | 4.224E-06 |
|  |  | rs1915019 | -0.008 | 0.002 | 4.925E-06 |
|  |  | rs2053079 | -0.008 | 0.002 | 5.648E-06 |
|  |  | rs2078266 | -0.008 | 0.002 | 4.760E-06 |
|  |  | rs215412 | -0.008 | 0.002 | 5.297E-06 |
|  |  | rs217336 | -0.008 | 0.002 | 3.390E-06 |
|  |  | rs2238057 | -0.008 | 0.002 | 2.734E-06 |
|  |  | rs2252074 | -0.008 | 0.002 | 5.198E-06 |
|  |  | rs2332700 | -0.008 | 0.002 | 3.627E-06 |
|  |  | rs2333321 | -0.008 | 0.002 | 5.334E-06 |
|  |  | rs2381411 | -0.008 | 0.002 | 6.255E-06 |
|  |  | rs2455415 | -0.008 | 0.002 | 4.991E-06 |
|  |  | rs2456020 | -0.008 | 0.002 | 8.909E-06 |
|  |  | rs2514218 | -0.008 | 0.002 | 9.516E-06 |
|  |  | rs2710323 | -0.008 | 0.002 | 5.945E-06 |
|  |  | rs2815731 | -0.008 | 0.002 | 4.025E-06 |
|  |  | rs2909457 | -0.008 | 0.002 | 3.813E-06 |
|  |  | rs2999392 | -0.008 | 0.002 | 2.900E-06 |
|  |  | rs308697 | -0.008 | 0.002 | 2.650E-06 |
|  |  | rs35351411 | -0.008 | 0.002 | 3.542E-06 |
|  |  | rs35734242 | -0.008 | 0.002 | 5.324E-06 |
|  |  | rs3739118 | -0.008 | 0.002 | 4.201E-06 |
|  |  | rs3770754 | -0.008 | 0.002 | 3.681E-06 |
|  |  | rs3791710 | -0.008 | 0.002 | 6.037E-06 |
|  |  | rs3795310 | -0.008 | 0.002 | 5.075E-06 |
|  |  | rs3802924 | -0.008 | 0.002 | 3.250E-06 |
|  |  | rs3814883 | -0.008 | 0.002 | 9.512E-06 |
|  |  | rs3824451 | -0.008 | 0.002 | 2.955E-06 |
|  |  | rs4129585 | -0.008 | 0.002 | 9.812E-06 |
|  |  | rs4575535 | -0.008 | 0.002 | 4.913E-06 |
|  |  | rs4632195 | -0.008 | 0.002 | 5.126E-06 |
|  |  | rs4636654 | -0.008 | 0.002 | 6.294E-06 |
|  |  | rs4653164 | -0.008 | 0.002 | 5.217E-06 |
|  |  | rs4702 | -0.008 | 0.002 | 5.104E-06 |
|  |  | rs4766428 | -0.008 | 0.002 | 2.260E-06 |
|  |  | rs4779050 | -0.008 | 0.002 | 4.536E-06 |
|  |  | rs4812325 | -0.008 | 0.002 | 3.183E-06 |
|  |  | rs4921741 | -0.008 | 0.002 | 2.146E-06 |
|  |  | rs498591 | -0.008 | 0.002 | 4.244E-06 |
|  |  | rs500102 | -0.008 | 0.002 | 3.214E-06 |
|  |  | rs505061 | -0.009 | 0.002 | 1.317E-06 |
|  |  | rs56205728 | -0.008 | 0.002 | 4.656E-06 |
|  |  | rs56335113 | -0.008 | 0.002 | 5.258E-06 |
|  |  | rs57433322 | -0.008 | 0.002 | 6.549E-06 |
|  |  | rs5751191 | -0.008 | 0.002 | 5.269E-06 |
|  |  | rs58120505 | -0.008 | 0.002 | 7.136E-06 |
|  |  | rs60135207 | -0.008 | 0.002 | 5.534E-06 |
|  |  | rs6125656 | -0.008 | 0.002 | 4.857E-06 |
|  |  | rs61857878 | -0.008 | 0.002 | 2.708E-06 |
|  |  | rs61937595 | -0.008 | 0.002 | 8.374E-06 |
|  |  | rs62018952 | -0.008 | 0.002 | 5.738E-06 |
|  |  | rs62183855 | -0.008 | 0.002 | 3.704E-06 |
|  |  | rs634940 | -0.008 | 0.002 | 3.176E-06 |
|  |  | rs6482437 | -0.008 | 0.002 | 4.699E-06 |
|  |  | rs6520064 | -0.008 | 0.002 | 3.824E-06 |
|  |  | rs6538539 | -0.008 | 0.002 | 5.914E-06 |
|  |  | rs6546857 | -0.008 | 0.002 | 6.010E-06 |
|  |  | rs6549963 | -0.008 | 0.002 | 2.521E-06 |
|  |  | rs6673880 | -0.008 | 0.002 | 3.859E-06 |
|  |  | rs6715366 | -0.008 | 0.002 | 4.717E-06 |
|  |  | rs6798742 | -0.008 | 0.002 | 4.002E-06 |
|  |  | rs6943762 | -0.008 | 0.002 | 8.021E-06 |
|  |  | rs6974218 | -0.008 | 0.002 | 4.306E-06 |
|  |  | rs6984242 | -0.008 | 0.002 | 5.431E-06 |
|  |  | rs708228 | -0.008 | 0.002 | 5.988E-06 |
|  |  | rs7112616 | -0.008 | 0.002 | 4.367E-06 |
|  |  | rs713692 | -0.008 | 0.002 | 2.859E-06 |
|  |  | rs7251 | -0.008 | 0.002 | 5.778E-06 |
|  |  | rs72802868 | -0.008 | 0.002 | 3.184E-06 |
|  |  | rs728055 | -0.008 | 0.002 | 4.943E-06 |
|  |  | rs72943392 | -0.008 | 0.002 | 4.588E-06 |
|  |  | rs72986630 | -0.008 | 0.002 | 3.747E-06 |
|  |  | rs73229090 | -0.008 | 0.002 | 5.590E-06 |
|  |  | rs73292401 | -0.008 | 0.002 | 6.259E-06 |
|  |  | rs7515363 | -0.008 | 0.002 | 4.344E-06 |
|  |  | rs7575796 | -0.008 | 0.002 | 4.210E-06 |
|  |  | rs7634476 | -0.008 | 0.002 | 6.410E-06 |
|  |  | rs7647398 | -0.008 | 0.002 | 2.240E-06 |
|  |  | rs76838079 | -0.008 | 0.002 | 3.047E-06 |
|  |  | rs778371 | -0.008 | 0.002 | 6.035E-06 |
|  |  | rs7798283 | -0.008 | 0.002 | 2.498E-06 |
|  |  | rs7830315 | -0.008 | 0.002 | 2.707E-06 |
|  |  | rs79210963 | -0.008 | 0.002 | 3.354E-06 |
|  |  | rs79445414 | -0.008 | 0.002 | 2.855E-06 |
|  |  | rs8055219 | -0.008 | 0.002 | 4.428E-06 |
|  |  | rs9304548 | -0.008 | 0.002 | 2.741E-06 |
|  |  | rs9318627 | -0.008 | 0.002 | 2.772E-06 |
|  |  | rs9461916 | -0.008 | 0.002 | 5.391E-06 |
|  |  | rs9636107 | -0.008 | 0.002 | 5.333E-06 |
|  |  | rs9687282 | -0.008 | 0.002 | 3.873E-06 |
|  |  | rs9876421 | -0.008 | 0.002 | 2.467E-06 |
|  |  | **All** | -0.008 | 0.002 | 4.046E-06 |

Abbreviations: MR, Mendelian randomization; SE, standard error; SNP, single nucleotide polymorphism.

## Table S30. MR leave-one-out sensitivity analysis for causal effect of schizophrenia on household income.

| **Exposure** | **Outcome** | **SNP** | **Beta** | **SE** | ***p value*** |
| --- | --- | --- | --- | --- | --- |
| Schizophrenia | Household income | rs1000237 | -0.036 | 0.007 | 8.503E-08 |
|  |  | rs10035564 | -0.036 | 0.007 | 9.617E-08 |
|  |  | rs10086619 | -0.037 | 0.007 | 4.983E-08 |
|  |  | rs10108980 | -0.037 | 0.007 | 6.522E-08 |
|  |  | rs10117 | -0.035 | 0.007 | 1.151E-07 |
|  |  | rs10861176 | -0.037 | 0.007 | 4.171E-08 |
|  |  | rs10873538 | -0.037 | 0.007 | 7.501E-08 |
|  |  | rs10876446 | -0.036 | 0.007 | 8.224E-08 |
|  |  | rs11027839 | -0.037 | 0.007 | 6.845E-08 |
|  |  | rs11136325 | -0.037 | 0.007 | 5.547E-08 |
|  |  | rs11165867 | -0.037 | 0.007 | 5.505E-08 |
|  |  | rs11191580 | -0.036 | 0.007 | 9.143E-08 |
|  |  | rs11210892 | -0.035 | 0.007 | 1.287E-07 |
|  |  | rs11223774 | -0.037 | 0.007 | 5.934E-08 |
|  |  | rs113264400 | -0.036 | 0.007 | 8.216E-08 |
|  |  | rs11534045 | -0.037 | 0.007 | 7.101E-08 |
|  |  | rs11587347 | -0.036 | 0.007 | 8.539E-08 |
|  |  | rs11664298 | -0.036 | 0.007 | 1.207E-07 |
|  |  | rs11693094 | -0.037 | 0.007 | 5.299E-08 |
|  |  | rs117178087 | -0.037 | 0.007 | 6.584E-08 |
|  |  | rs11941714 | -0.036 | 0.007 | 8.118E-08 |
|  |  | rs1198588 | -0.038 | 0.007 | 2.094E-08 |
|  |  | rs12129573 | -0.036 | 0.007 | 9.085E-08 |
|  |  | rs12138231 | -0.037 | 0.007 | 7.079E-08 |
|  |  | rs12151767 | -0.038 | 0.007 | 2.415E-08 |
|  |  | rs12285419 | -0.037 | 0.007 | 5.480E-08 |
|  |  | rs12293670 | -0.037 | 0.007 | 4.574E-08 |
|  |  | rs12303743 | -0.036 | 0.007 | 8.642E-08 |
|  |  | rs12489270 | -0.036 | 0.007 | 1.119E-07 |
|  |  | rs12652777 | -0.036 | 0.007 | 1.026E-07 |
|  |  | rs12712510 | -0.036 | 0.007 | 8.448E-08 |
|  |  | rs12771371 | -0.037 | 0.007 | 5.504E-08 |
|  |  | rs12833624 | -0.036 | 0.007 | 8.584E-08 |
|  |  | rs12877581 | -0.037 | 0.007 | 6.190E-08 |
|  |  | rs12883788 | -0.034 | 0.007 | 1.155E-07 |
|  |  | rs13016542 | -0.037 | 0.007 | 7.532E-08 |
|  |  | rs13107325 | -0.035 | 0.007 | 2.150E-07 |
|  |  | rs13195636 | -0.037 | 0.007 | 9.263E-08 |
|  |  | rs13233308 | -0.037 | 0.007 | 6.599E-08 |
|  |  | rs132582 | -0.036 | 0.007 | 1.052E-07 |
|  |  | rs1430894 | -0.036 | 0.007 | 1.050E-07 |
|  |  | rs145071536 | -0.036 | 0.007 | 8.362E-08 |
|  |  | rs1451488 | -0.037 | 0.007 | 5.051E-08 |
|  |  | rs149165 | -0.037 | 0.007 | 7.190E-08 |
|  |  | rs1593304 | -0.037 | 0.007 | 3.667E-08 |
|  |  | rs1604060 | -0.036 | 0.007 | 9.999E-08 |
|  |  | rs1615350 | -0.038 | 0.007 | 1.832E-08 |
|  |  | rs167924 | -0.036 | 0.007 | 8.958E-08 |
|  |  | rs16851048 | -0.037 | 0.007 | 7.003E-08 |
|  |  | rs16867571 | -0.037 | 0.007 | 3.750E-08 |
|  |  | rs17016552 | -0.037 | 0.007 | 7.189E-08 |
|  |  | rs17194490 | -0.037 | 0.007 | 6.035E-08 |
|  |  | rs17731 | -0.036 | 0.007 | 8.677E-08 |
|  |  | rs187557 | -0.037 | 0.007 | 2.449E-08 |
|  |  | rs1881046 | -0.036 | 0.007 | 8.068E-08 |
|  |  | rs1901512 | -0.036 | 0.007 | 1.011E-07 |
|  |  | rs1915019 | -0.037 | 0.007 | 6.142E-08 |
|  |  | rs2053079 | -0.036 | 0.007 | 1.044E-07 |
|  |  | rs2078266 | -0.037 | 0.007 | 6.839E-08 |
|  |  | rs215412 | -0.036 | 0.007 | 1.111E-07 |
|  |  | rs217336 | -0.036 | 0.007 | 8.965E-08 |
|  |  | rs2238057 | -0.037 | 0.007 | 3.631E-08 |
|  |  | rs2252074 | -0.036 | 0.007 | 1.130E-07 |
|  |  | rs2332700 | -0.037 | 0.007 | 3.881E-08 |
|  |  | rs2333321 | -0.036 | 0.007 | 9.004E-08 |
|  |  | rs2381411 | -0.037 | 0.007 | 7.120E-08 |
|  |  | rs2455415 | -0.037 | 0.007 | 6.251E-08 |
|  |  | rs2456020 | -0.035 | 0.007 | 1.470E-07 |
|  |  | rs2514218 | -0.037 | 0.007 | 7.626E-08 |
|  |  | rs2710323 | -0.036 | 0.007 | 9.614E-08 |
|  |  | rs2815731 | -0.037 | 0.007 | 4.622E-08 |
|  |  | rs2909457 | -0.036 | 0.007 | 8.876E-08 |
|  |  | rs2999392 | -0.037 | 0.007 | 4.927E-08 |
|  |  | rs308697 | -0.037 | 0.007 | 5.245E-08 |
|  |  | rs35351411 | -0.037 | 0.007 | 6.253E-08 |
|  |  | rs35734242 | -0.037 | 0.007 | 6.699E-08 |
|  |  | rs3739118 | -0.036 | 0.007 | 9.800E-08 |
|  |  | rs3770754 | -0.036 | 0.007 | 8.435E-08 |
|  |  | rs3791710 | -0.037 | 0.007 | 5.667E-08 |
|  |  | rs3795310 | -0.036 | 0.007 | 1.013E-07 |
|  |  | rs3802924 | -0.036 | 0.007 | 1.026E-07 |
|  |  | rs3814883 | -0.037 | 0.007 | 5.446E-08 |
|  |  | rs3824451 | -0.036 | 0.007 | 7.709E-08 |
|  |  | rs4129585 | -0.036 | 0.007 | 1.491E-07 |
|  |  | rs4575535 | -0.037 | 0.007 | 4.465E-08 |
|  |  | rs4632195 | -0.035 | 0.007 | 9.754E-08 |
|  |  | rs4636654 | -0.036 | 0.007 | 1.025E-07 |
|  |  | rs4653164 | -0.037 | 0.007 | 5.343E-08 |
|  |  | rs4702 | -0.038 | 0.007 | 1.711E-08 |
|  |  | rs4766428 | -0.037 | 0.007 | 3.458E-08 |
|  |  | rs4779050 | -0.037 | 0.007 | 6.400E-08 |
|  |  | rs4812325 | -0.037 | 0.007 | 4.418E-08 |
|  |  | rs4921741 | -0.037 | 0.007 | 3.328E-08 |
|  |  | rs498591 | -0.036 | 0.007 | 1.006E-07 |
|  |  | rs500102 | -0.037 | 0.007 | 4.937E-08 |
|  |  | rs505061 | -0.037 | 0.007 | 5.205E-08 |
|  |  | rs56205728 | -0.037 | 0.007 | 5.076E-08 |
|  |  | rs56335113 | -0.037 | 0.007 | 6.307E-08 |
|  |  | rs57433322 | -0.037 | 0.007 | 5.327E-08 |
|  |  | rs5751191 | -0.036 | 0.007 | 1.026E-07 |
|  |  | rs58120505 | -0.036 | 0.007 | 1.059E-07 |
|  |  | rs60135207 | -0.035 | 0.007 | 1.013E-07 |
|  |  | rs6125656 | -0.036 | 0.007 | 8.218E-08 |
|  |  | rs61857878 | -0.036 | 0.007 | 8.416E-08 |
|  |  | rs61937595 | -0.037 | 0.007 | 7.319E-08 |
|  |  | rs62018952 | -0.036 | 0.007 | 1.037E-07 |
|  |  | rs62183855 | -0.037 | 0.007 | 6.790E-08 |
|  |  | rs634940 | -0.037 | 0.007 | 2.634E-08 |
|  |  | rs6482437 | -0.037 | 0.007 | 2.613E-08 |
|  |  | rs6520064 | -0.037 | 0.007 | 6.555E-08 |
|  |  | rs6538539 | -0.036 | 0.007 | 1.091E-07 |
|  |  | rs6546857 | -0.035 | 0.007 | 1.105E-07 |
|  |  | rs6549963 | -0.037 | 0.007 | 3.341E-08 |
|  |  | rs6673880 | -0.036 | 0.007 | 7.976E-08 |
|  |  | rs6715366 | -0.037 | 0.007 | 5.437E-08 |
|  |  | rs6798742 | -0.037 | 0.007 | 6.748E-08 |
|  |  | rs6943762 | -0.036 | 0.007 | 1.017E-07 |
|  |  | rs6974218 | -0.036 | 0.007 | 1.003E-07 |
|  |  | rs6984242 | -0.036 | 0.007 | 8.064E-08 |
|  |  | rs708228 | -0.035 | 0.007 | 1.022E-07 |
|  |  | rs7112616 | -0.037 | 0.007 | 3.217E-08 |
|  |  | rs713692 | -0.037 | 0.007 | 7.359E-08 |
|  |  | rs7251 | -0.037 | 0.007 | 3.157E-08 |
|  |  | rs72802868 | -0.037 | 0.007 | 7.720E-08 |
|  |  | rs728055 | -0.037 | 0.007 | 4.434E-08 |
|  |  | rs72943392 | -0.037 | 0.007 | 7.148E-08 |
|  |  | rs72986630 | -0.037 | 0.007 | 5.920E-08 |
|  |  | rs73229090 | -0.036 | 0.007 | 9.506E-08 |
|  |  | rs73292401 | -0.036 | 0.007 | 9.952E-08 |
|  |  | rs7515363 | -0.037 | 0.007 | 7.151E-08 |
|  |  | rs7575796 | -0.037 | 0.007 | 6.187E-08 |
|  |  | rs7634476 | -0.036 | 0.007 | 1.008E-07 |
|  |  | rs7647398 | -0.037 | 0.007 | 7.445E-08 |
|  |  | rs76838079 | -0.037 | 0.007 | 5.803E-08 |
|  |  | rs778371 | -0.037 | 0.007 | 4.869E-08 |
|  |  | rs7798283 | -0.036 | 0.007 | 8.385E-08 |
|  |  | rs7830315 | -0.037 | 0.007 | 4.570E-08 |
|  |  | rs79210963 | -0.037 | 0.007 | 5.325E-08 |
|  |  | rs79445414 | -0.036 | 0.007 | 8.559E-08 |
|  |  | rs8055219 | -0.036 | 0.007 | 9.811E-08 |
|  |  | rs9304548 | -0.037 | 0.007 | 5.556E-08 |
|  |  | rs9318627 | -0.037 | 0.007 | 4.171E-08 |
|  |  | rs9461916 | -0.036 | 0.007 | 8.404E-08 |
|  |  | rs9636107 | -0.036 | 0.007 | 1.164E-07 |
|  |  | rs9687282 | -0.037 | 0.007 | 6.706E-08 |
|  |  | rs9876421 | -0.037 | 0.007 | 6.657E-08 |
|  |  | **All** | -0.037 | 0.007 | 6.205E-08 |

Abbreviations: MR, Mendelian randomization; SE, standard error; SNP, single nucleotide polymorphism.

## Table S31. MR leave-one-out sensitivity analysis for causal effect of schizophrenia on TDI.

| **Exposure** | **Outcome** | **SNP** | **Beta** | **SE** | ***p* value** |
| --- | --- | --- | --- | --- | --- |
| Schizophrenia | TDI | rs1000237 | 0.022 | 0.005 | 8.059E-07 |
|  |  | rs10035564 | 0.021 | 0.005 | 7.190E-06 |
|  |  | rs10086619 | 0.021 | 0.005 | 4.775E-06 |
|  |  | rs10108980 | 0.021 | 0.005 | 3.880E-06 |
|  |  | rs10117 | 0.021 | 0.005 | 7.074E-06 |
|  |  | rs10861176 | 0.022 | 0.005 | 3.088E-06 |
|  |  | rs10873538 | 0.021 | 0.005 | 8.044E-06 |
|  |  | rs10876446 | 0.021 | 0.005 | 5.453E-06 |
|  |  | rs11027839 | 0.021 | 0.005 | 5.360E-06 |
|  |  | rs11136325 | 0.021 | 0.005 | 5.890E-06 |
|  |  | rs11165867 | 0.021 | 0.005 | 4.312E-06 |
|  |  | rs11191580 | 0.023 | 0.005 | 5.513E-07 |
|  |  | rs11210892 | 0.020 | 0.005 | 7.935E-06 |
|  |  | rs11223774 | 0.021 | 0.005 | 3.961E-06 |
|  |  | rs113264400 | 0.021 | 0.005 | 4.425E-06 |
|  |  | rs11534045 | 0.022 | 0.005 | 1.230E-06 |
|  |  | rs11587347 | 0.022 | 0.005 | 2.960E-06 |
|  |  | rs11664298 | 0.020 | 0.005 | 8.682E-06 |
|  |  | rs11693094 | 0.021 | 0.005 | 5.086E-06 |
|  |  | rs117178087 | 0.021 | 0.005 | 4.696E-06 |
|  |  | rs11941714 | 0.021 | 0.005 | 6.293E-06 |
|  |  | rs1198588 | 0.021 | 0.005 | 3.855E-06 |
|  |  | rs12129573 | 0.021 | 0.005 | 7.756E-06 |
|  |  | rs12138231 | 0.021 | 0.005 | 5.827E-06 |
|  |  | rs12151767 | 0.021 | 0.005 | 4.555E-06 |
|  |  | rs12285419 | 0.022 | 0.005 | 3.432E-06 |
|  |  | rs12293670 | 0.021 | 0.005 | 4.852E-06 |
|  |  | rs12303743 | 0.021 | 0.005 | 5.951E-06 |
|  |  | rs12489270 | 0.021 | 0.005 | 6.563E-06 |
|  |  | rs12652777 | 0.021 | 0.005 | 6.672E-06 |
|  |  | rs12712510 | 0.021 | 0.005 | 6.473E-06 |
|  |  | rs12771371 | 0.021 | 0.005 | 5.535E-06 |
|  |  | rs12833624 | 0.021 | 0.005 | 5.383E-06 |
|  |  | rs12877581 | 0.021 | 0.005 | 5.437E-06 |
|  |  | rs12883788 | 0.020 | 0.005 | 8.497E-06 |
|  |  | rs13016542 | 0.021 | 0.005 | 4.366E-06 |
|  |  | rs13107325 | 0.022 | 0.005 | 1.759E-06 |
|  |  | rs13195636 | 0.020 | 0.005 | 1.759E-05 |
|  |  | rs13233308 | 0.021 | 0.005 | 3.644E-06 |
|  |  | rs132582 | 0.021 | 0.005 | 5.169E-06 |
|  |  | rs1430894 | 0.021 | 0.005 | 6.587E-06 |
|  |  | rs145071536 | 0.021 | 0.005 | 3.627E-06 |
|  |  | rs1451488 | 0.021 | 0.005 | 4.123E-06 |
|  |  | rs149165 | 0.021 | 0.005 | 5.820E-06 |
|  |  | rs1593304 | 0.021 | 0.005 | 3.739E-06 |
|  |  | rs1604060 | 0.020 | 0.005 | 6.690E-06 |
|  |  | rs1615350 | 0.021 | 0.005 | 3.565E-06 |
|  |  | rs167924 | 0.021 | 0.005 | 5.533E-06 |
|  |  | rs16851048 | 0.022 | 0.005 | 2.533E-06 |
|  |  | rs16867571 | 0.021 | 0.005 | 3.518E-06 |
|  |  | rs17016552 | 0.021 | 0.005 | 3.773E-06 |
|  |  | rs17194490 | 0.021 | 0.005 | 4.717E-06 |
|  |  | rs17731 | 0.021 | 0.005 | 5.126E-06 |
|  |  | rs187557 | 0.021 | 0.005 | 5.447E-06 |
|  |  | rs1881046 | 0.021 | 0.005 | 3.942E-06 |
|  |  | rs1901512 | 0.021 | 0.005 | 6.982E-06 |
|  |  | rs1915019 | 0.021 | 0.005 | 4.734E-06 |
|  |  | rs2053079 | 0.022 | 0.005 | 3.035E-06 |
|  |  | rs2078266 | 0.021 | 0.005 | 5.647E-06 |
|  |  | rs215412 | 0.021 | 0.005 | 5.431E-06 |
|  |  | rs217336 | 0.021 | 0.005 | 4.268E-06 |
|  |  | rs2238057 | 0.022 | 0.005 | 2.485E-06 |
|  |  | rs2252074 | 0.021 | 0.005 | 5.630E-06 |
|  |  | rs2332700 | 0.021 | 0.005 | 3.522E-06 |
|  |  | rs2333321 | 0.021 | 0.005 | 4.898E-06 |
|  |  | rs2381411 | 0.021 | 0.005 | 3.791E-06 |
|  |  | rs2455415 | 0.021 | 0.005 | 4.376E-06 |
|  |  | rs2456020 | 0.021 | 0.005 | 5.887E-06 |
|  |  | rs2514218 | 0.021 | 0.005 | 5.245E-06 |
|  |  | rs2710323 | 0.022 | 0.005 | 1.800E-06 |
|  |  | rs2815731 | 0.021 | 0.005 | 3.627E-06 |
|  |  | rs2909457 | 0.021 | 0.005 | 3.807E-06 |
|  |  | rs2999392 | 0.021 | 0.005 | 4.336E-06 |
|  |  | rs308697 | 0.021 | 0.005 | 4.315E-06 |
|  |  | rs35351411 | 0.022 | 0.005 | 2.438E-06 |
|  |  | rs35734242 | 0.021 | 0.005 | 4.807E-06 |
|  |  | rs3739118 | 0.021 | 0.005 | 5.500E-06 |
|  |  | rs3770754 | 0.021 | 0.005 | 4.517E-06 |
|  |  | rs3791710 | 0.021 | 0.005 | 5.152E-06 |
|  |  | rs3795310 | 0.021 | 0.005 | 6.130E-06 |
|  |  | rs3802924 | 0.021 | 0.005 | 6.265E-06 |
|  |  | rs3814883 | 0.022 | 0.005 | 2.699E-06 |
|  |  | rs3824451 | 0.021 | 0.005 | 4.718E-06 |
|  |  | rs4129585 | 0.021 | 0.005 | 7.213E-06 |
|  |  | rs4575535 | 0.021 | 0.005 | 4.656E-06 |
|  |  | rs4632195 | 0.021 | 0.005 | 5.150E-06 |
|  |  | rs4636654 | 0.021 | 0.005 | 5.143E-06 |
|  |  | rs4653164 | 0.021 | 0.005 | 5.458E-06 |
|  |  | rs4702 | 0.021 | 0.005 | 4.012E-06 |
|  |  | rs4766428 | 0.022 | 0.005 | 1.883E-06 |
|  |  | rs4779050 | 0.021 | 0.005 | 3.575E-06 |
|  |  | rs4812325 | 0.021 | 0.005 | 4.717E-06 |
|  |  | rs4921741 | 0.022 | 0.005 | 3.040E-06 |
|  |  | rs498591 | 0.021 | 0.005 | 5.507E-06 |
|  |  | rs500102 | 0.021 | 0.005 | 6.806E-06 |
|  |  | rs505061 | 0.021 | 0.005 | 5.663E-06 |
|  |  | rs56205728 | 0.021 | 0.005 | 4.002E-06 |
|  |  | rs56335113 | 0.022 | 0.005 | 2.996E-06 |
|  |  | rs57433322 | 0.022 | 0.005 | 2.561E-06 |
|  |  | rs5751191 | 0.022 | 0.005 | 1.165E-06 |
|  |  | rs58120505 | 0.021 | 0.005 | 5.562E-06 |
|  |  | rs60135207 | 0.021 | 0.005 | 5.808E-06 |
|  |  | rs6125656 | 0.021 | 0.005 | 4.892E-06 |
|  |  | rs61857878 | 0.021 | 0.005 | 5.546E-06 |
|  |  | rs61937595 | 0.022 | 0.005 | 8.695E-07 |
|  |  | rs62018952 | 0.021 | 0.005 | 6.175E-06 |
|  |  | rs62183855 | 0.021 | 0.005 | 4.090E-06 |
|  |  | rs634940 | 0.021 | 0.005 | 4.783E-06 |
|  |  | rs6482437 | 0.022 | 0.005 | 2.573E-06 |
|  |  | rs6520064 | 0.021 | 0.005 | 5.870E-06 |
|  |  | rs6538539 | 0.021 | 0.005 | 5.912E-06 |
|  |  | rs6546857 | 0.021 | 0.005 | 5.523E-06 |
|  |  | rs6549963 | 0.021 | 0.005 | 3.598E-06 |
|  |  | rs6673880 | 0.021 | 0.005 | 3.903E-06 |
|  |  | rs6715366 | 0.021 | 0.005 | 3.415E-06 |
|  |  | rs6798742 | 0.021 | 0.005 | 3.781E-06 |
|  |  | rs6943762 | 0.022 | 0.005 | 2.755E-06 |
|  |  | rs6974218 | 0.021 | 0.005 | 5.884E-06 |
|  |  | rs6984242 | 0.022 | 0.005 | 3.189E-06 |
|  |  | rs708228 | 0.021 | 0.005 | 6.830E-06 |
|  |  | rs7112616 | 0.021 | 0.005 | 3.879E-06 |
|  |  | rs713692 | 0.021 | 0.005 | 5.991E-06 |
|  |  | rs7251 | 0.022 | 0.005 | 2.945E-06 |
|  |  | rs72802868 | 0.020 | 0.005 | 8.466E-06 |
|  |  | rs728055 | 0.021 | 0.005 | 5.006E-06 |
|  |  | rs72943392 | 0.021 | 0.005 | 4.658E-06 |
|  |  | rs72986630 | 0.021 | 0.005 | 4.348E-06 |
|  |  | rs73229090 | 0.021 | 0.005 | 4.563E-06 |
|  |  | rs73292401 | 0.021 | 0.005 | 4.430E-06 |
|  |  | rs7515363 | 0.021 | 0.005 | 5.060E-06 |
|  |  | rs7575796 | 0.021 | 0.005 | 4.572E-06 |
|  |  | rs7634476 | 0.022 | 0.005 | 2.931E-06 |
|  |  | rs7647398 | 0.022 | 0.005 | 2.497E-06 |
|  |  | rs76838079 | 0.021 | 0.005 | 6.729E-06 |
|  |  | rs778371 | 0.021 | 0.005 | 4.211E-06 |
|  |  | rs7798283 | 0.021 | 0.005 | 4.842E-06 |
|  |  | rs7830315 | 0.021 | 0.005 | 3.711E-06 |
|  |  | rs79210963 | 0.021 | 0.005 | 5.933E-06 |
|  |  | rs79445414 | 0.021 | 0.005 | 6.315E-06 |
|  |  | rs8055219 | 0.021 | 0.005 | 6.867E-06 |
|  |  | rs9304548 | 0.021 | 0.005 | 4.399E-06 |
|  |  | rs9318627 | 0.021 | 0.005 | 4.619E-06 |
|  |  | rs9461916 | 0.021 | 0.005 | 4.320E-06 |
|  |  | rs9636107 | 0.020 | 0.005 | 9.746E-06 |
|  |  | rs9687282 | 0.021 | 0.005 | 4.819E-06 |
|  |  | rs9876421 | 0.021 | 0.005 | 4.817E-06 |
|  |  | **All** | 0.021 | 0.005 | 4.128E-06 |

Abbreviations: MR, Mendelian randomisation; TDI, Townsend deprivation index; SE, standard error; SNP, single nucleotide polymorphism.

## Table S32. Results for sensitivity analyses in multivariable MR analysis.

| **Outcome** | **Exposures** | **Heterogeneity test** | | | | **Pleiotropy test** | | |
| --- | --- | --- | --- | --- | --- | --- | --- | --- |
|  |  | **IVW *Q*** | ***p value*** | **Egger *Q*** | ***p*** | **Egger intercept** | **SE** | ***p value*** |
| MDD | Schizophrenia | 459.34 | 1.31E-12 | 458.52 | 1.18E-12 | -0.001 | 0.002 | 0.491 |
|  | Educational years |  |  |  |  |  |  |  |
|  | Household income |  |  |  |  |  |  |  |
|  | TDI |  |  |  |  |  |  |  |

Abbreviations: IVW, inverse variance weighted; MDD, major depressive disorder; MR, Mendelian randomisation; SE, standard error; TDI, Townsend deprivation index.

## Table S33. The statistical power for the univariable MR analyses.

| **Exposure** | ***R*^2^** | **Outcome** | **Sample size (case/control)** | **Causal effect*** | **Statistical power** |
| --- | --- | --- | --- | --- | --- |
| Schizophrenia | 0.050 | MDD | 142,646 (0.474) | 1.137 (OR) | 99.9% |
|  | 0.050 | Educational years | 766,345 | 0.003 (*β*) | 8.5% |
|  | 0.050 | Household income | 397,751 | -0.037 (*β*) | 99.9% |
|  | 0.050 | Employment status | 461,242 | -0.008 (*β*) | 22.8% |
|  | 0.050 | TDI | 462,464 | 0.021 (*β*) | 89.1% |
|  |  |  |  |  |  |
| MDD | 0.002 | Schizophrenia | 130,644 (0.691) | 1.323 (OR) | 60.4% |
|  | 0.003 | Educational years | 766,345 | 0.045 (*β*) | 57.8% |
|  | 0.003 | Household income | 397,751 | -0.022 (*β*) | 11.5% |
|  | 0.003 | Employment status | 461,242 | -0.012 (*β*) | 6.5% |
|  | 0.003 | TDI | 462,464 | 0.041 (*β*) | 33.3% |

Note: *for binary outcome, we used OR for causal effect; for continuous outcome, we used *β* for causal effect.

Abbreviations: MDD, major depressive disorder; MR, Mendelian randomisation; OR, odds ratio; TDI, Townsend deprivation index.

# Supplementary Figures


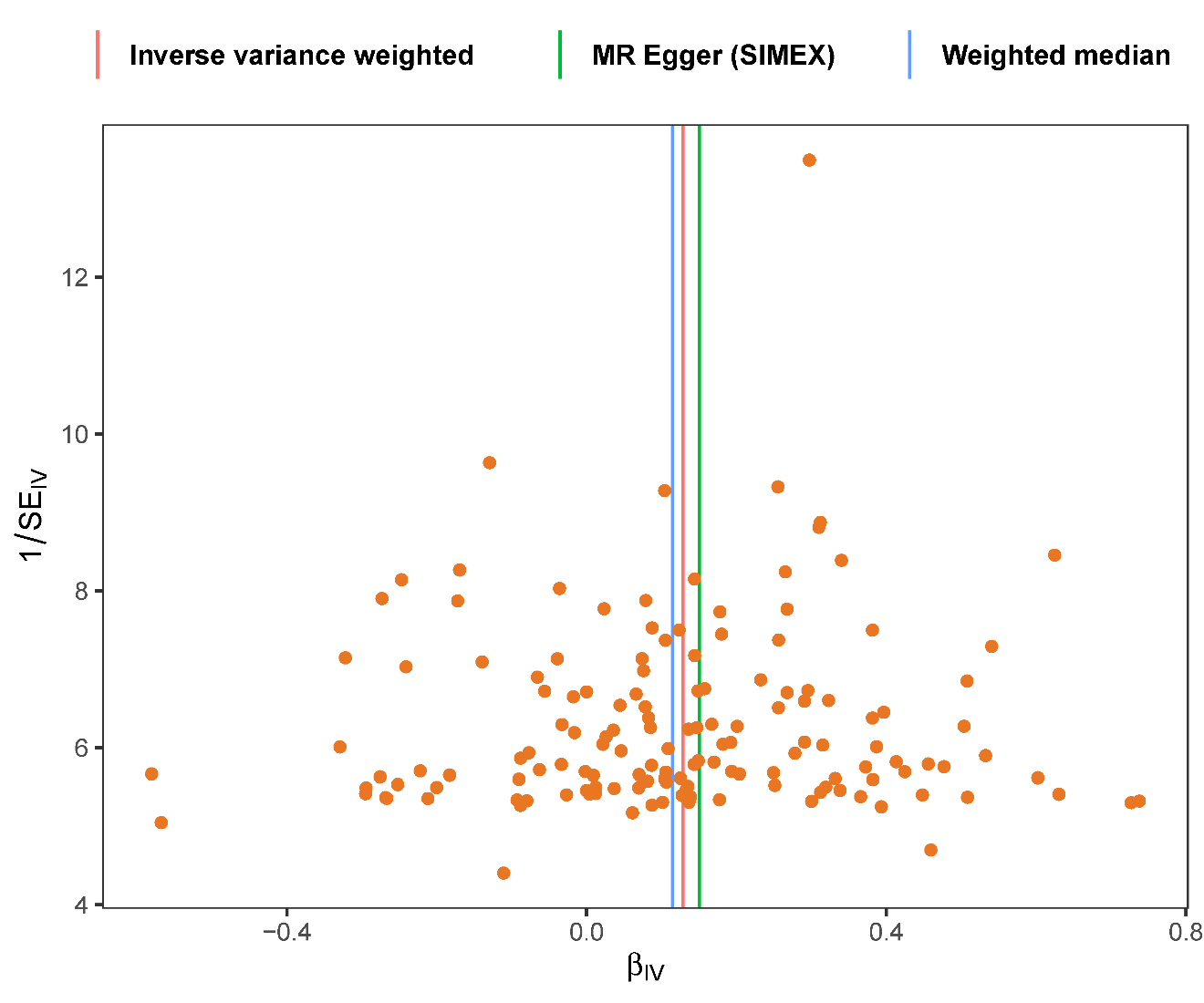


## **Fig. S1. Funnel plot for the causal effect of schizophrenia on MDD.**

In the funnel plot, each dot (orange) represents a SNP, the horizontal axis denotes the MR estimate for each SNP, and the vertical axis denotes the inverse standard error for MR estimate of each SNP. The three vertical lines (colors) represent the causal effect estimated by IVW, MR-Egger (SIMEX) and weighted median methods.

Abbreviations: IV, instrumental variable; IVW, inverse variance weighted; MDD, major depressive disorder; MR, Mendelian randomisation; SIMEX, simulation extrapolation; SE, standard error; SNP, single nucleotide polymorphism.


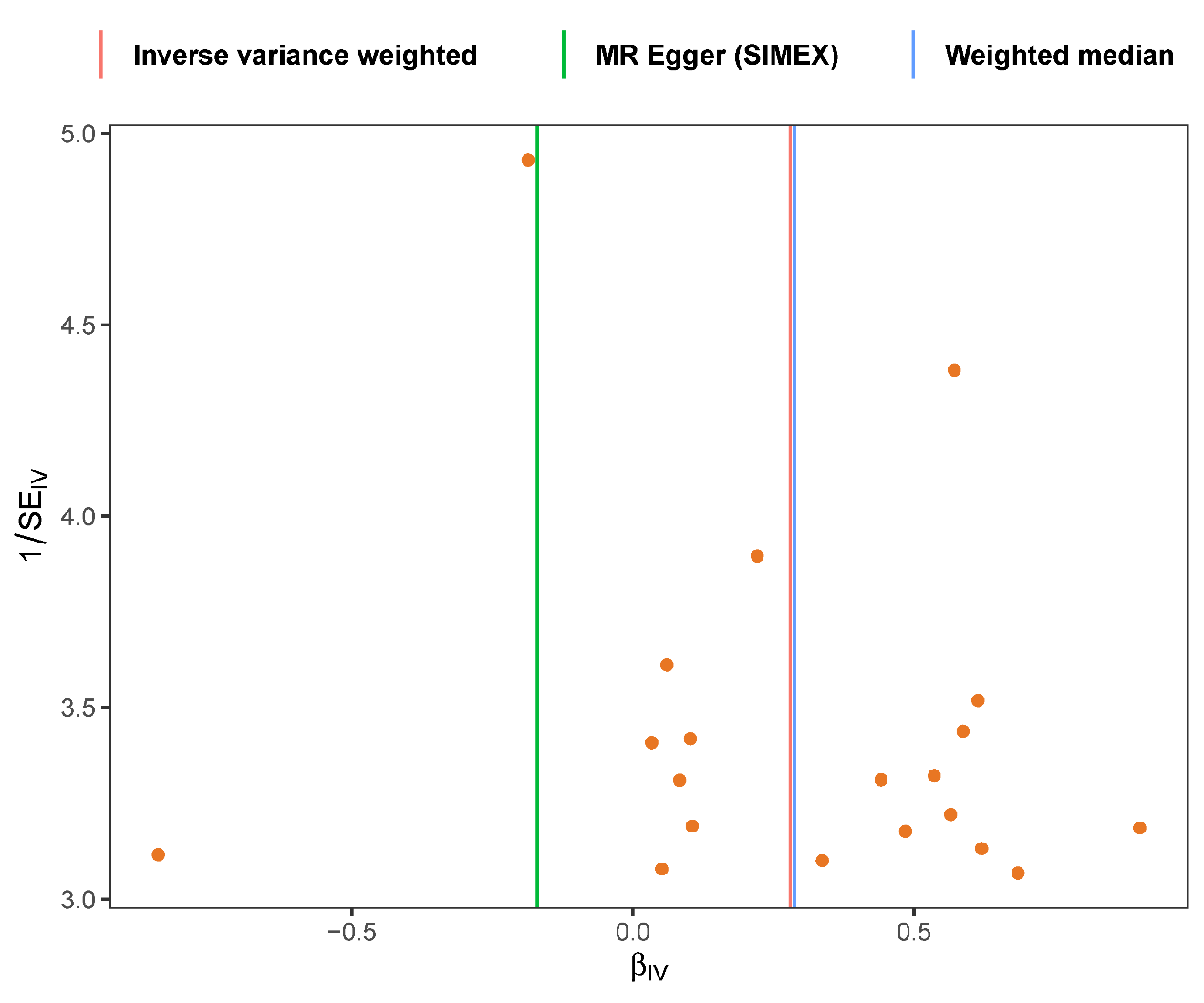


## **Fig. S**2. Funnel plot for the causal effect of MDD on schizophrenia.

In the funnel plot, each dot (orange) represents a SNP, the horizontal axis denotes the MR estimate for each SNP, and the vertical axis denotes the inverse standard error for MR estimate of each SNP. The three vertical lines (colors) represent the causal effect estimated by IVW, MR-Egger (SIMEX) and weighted median methods.

Abbreviations: IV, instrumental variable; IVW, inverse variance weighted; MDD, major depressive disorder; MR, Mendelian randomisation; SIMEX, simulation extrapolation; SE, standard error; SNP, single nucleotide polymorphism.


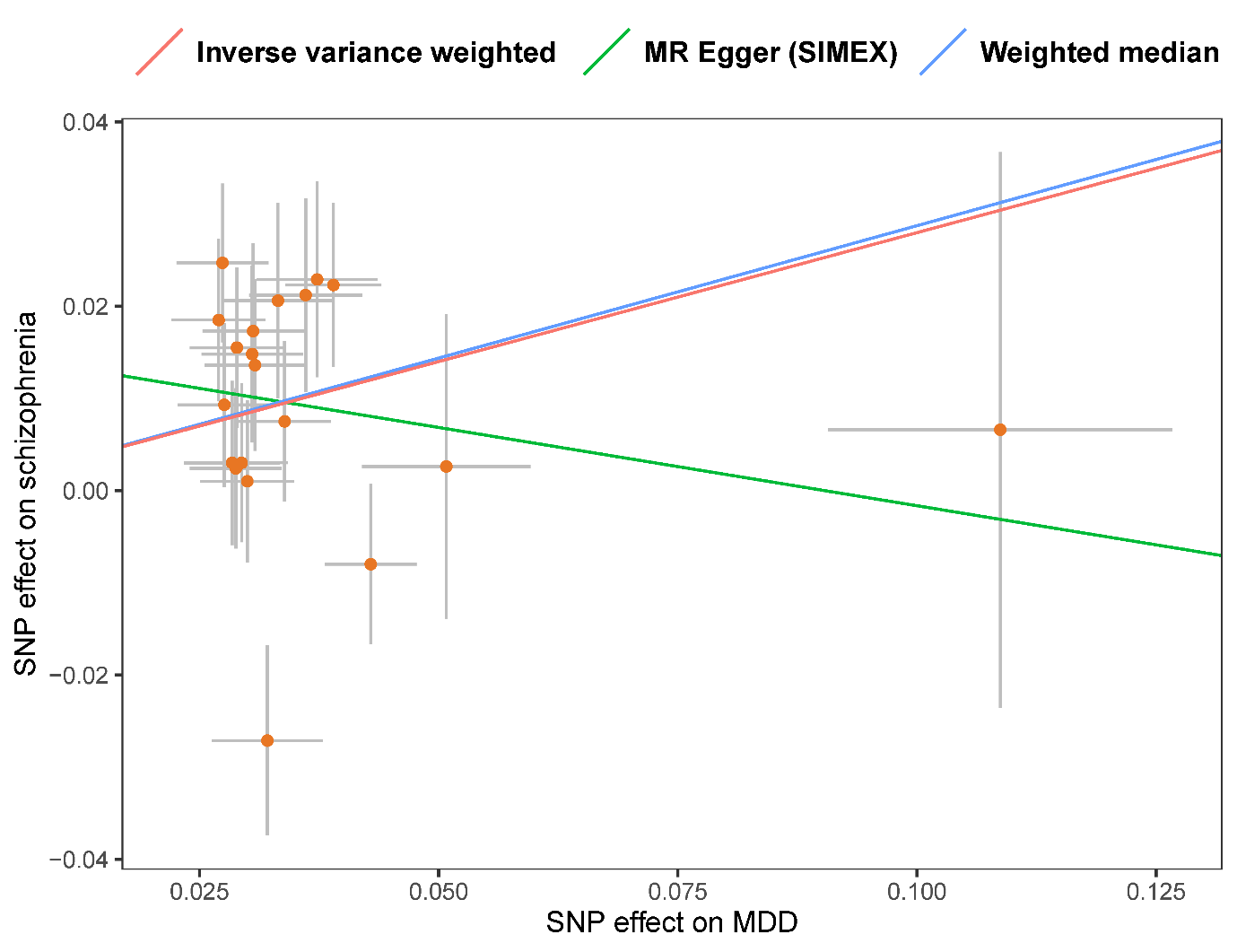


## **Fig. S**3. Scatter plot for the causal effect of MDD on schizophrenia.

In the scatter plot, each dot (orange) represents a SNP, the error bars (grey) at each dot represent the 95% confidence intervals. The horizontal axis is the SNP effect on exposure (MDD), while the vertical axis is the SNP effect on outcome (schizophrenia). The three fitted lines (colors) represent the results of MR under three methods (shown in the top panel), with the slope of each line corresponding to the estimated causal effect for each method.

Abbreviations: IVW, inverse variance weighted; MDD, major depressive disorder; MR, Mendelian randomisation; SIMEX, simulation extrapolation; SNP, single nucleotide polymorphism.


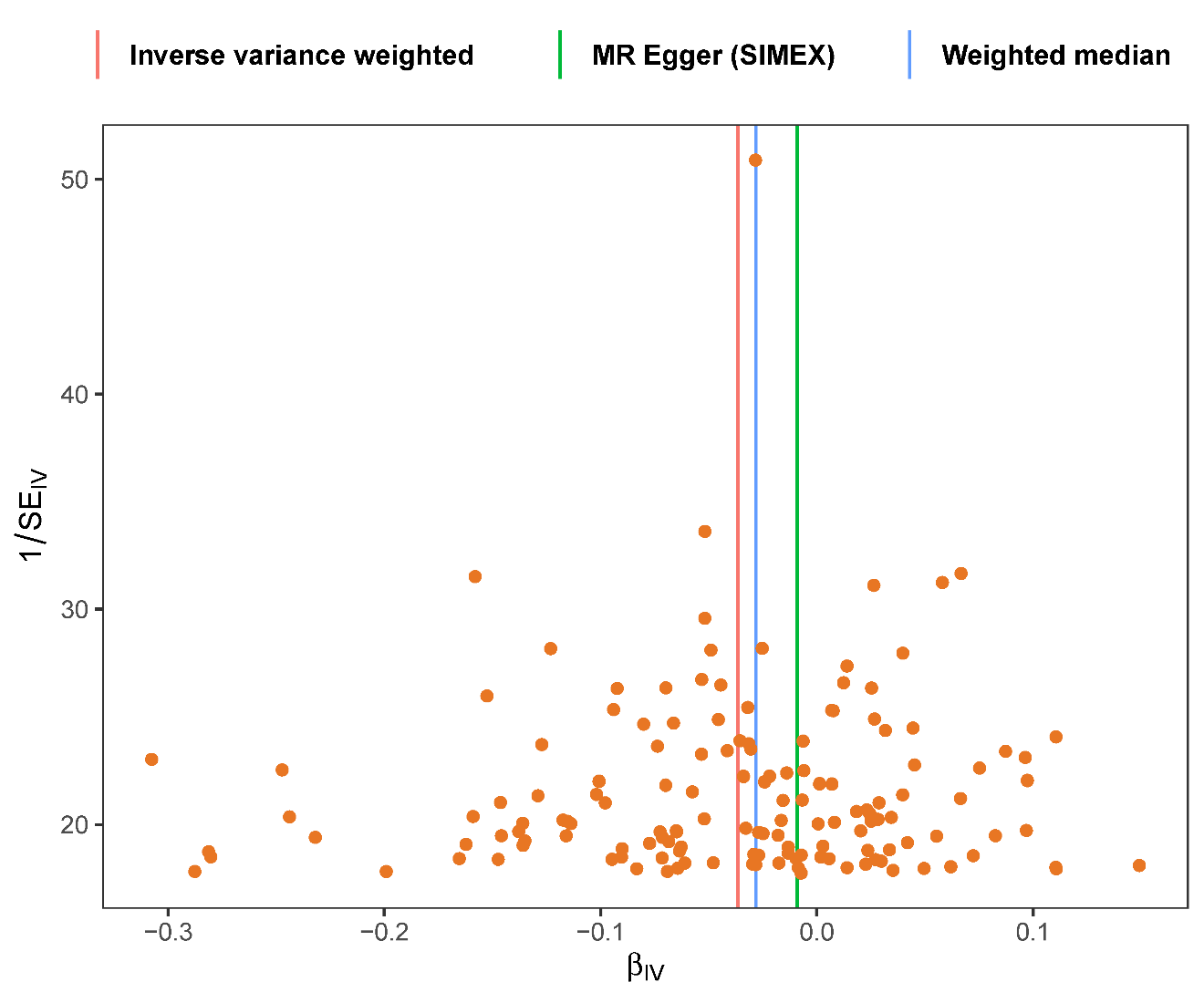


## **Fig. S**4. Funnel plot for the causal effect of schizophrenia on household income.

In the funnel plot, each dot (orange) represents a SNP, the horizontal axis denotes the MR estimate for each SNP, and the vertical axis denotes the inverse standard error for MR estimate of each SNP. The three vertical lines (colors) represent the causal effect estimated by IVW, MR-Egger (SIMEX) and weighted median methods.

Abbreviations: IV, instrumental variable; IVW, inverse variance weighted; MR, Mendelian randomisation; SIMEX, simulation extrapolation; SE, standard error; SNP, single nucleotide polymorphism.


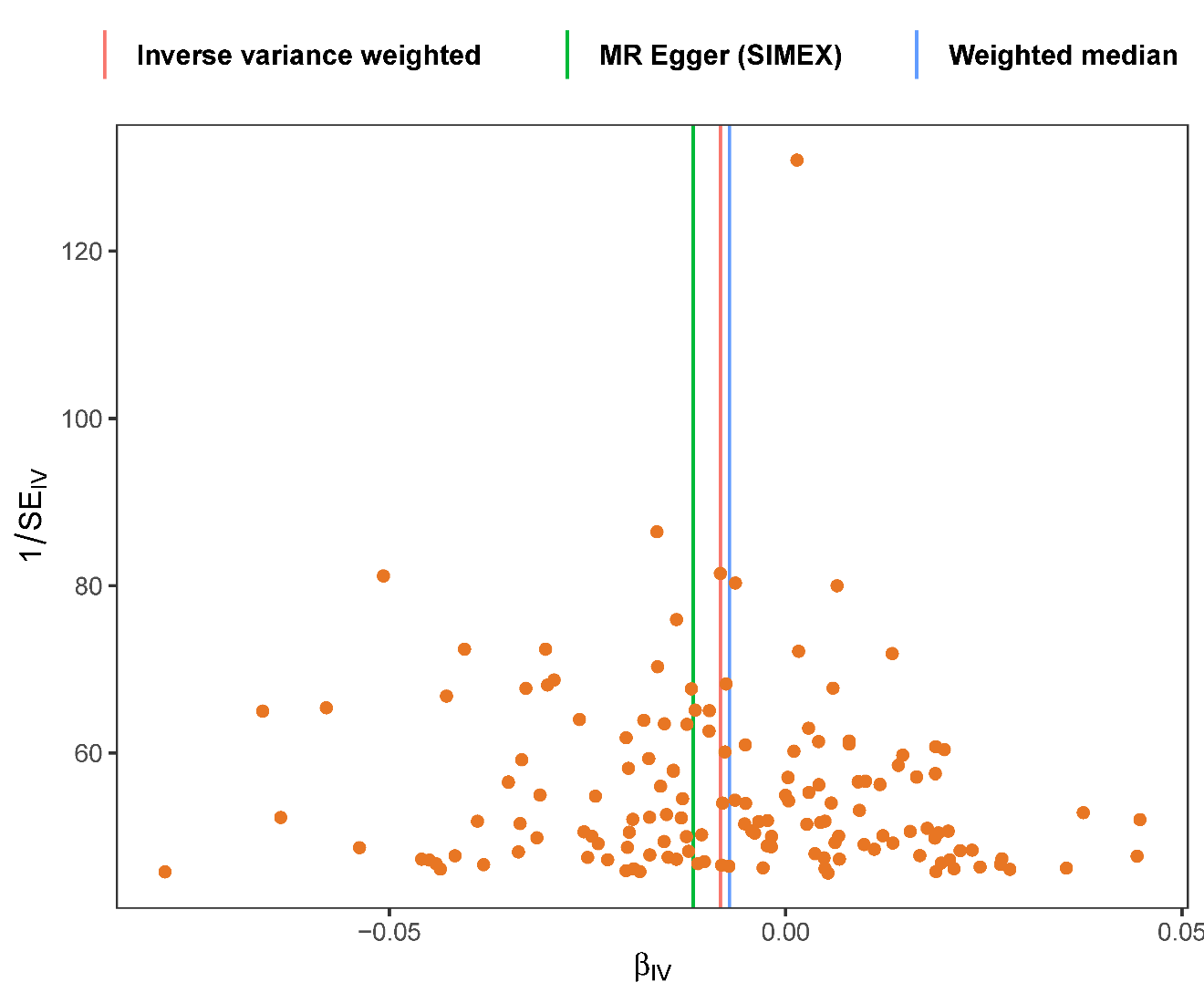


## **Fig. S**5. Funnel plot for the causal effect of schizophrenia on employment status.

In the funnel plot, each dot (orange) represents a SNP, the horizontal axis denotes the MR estimate for each SNP, and the vertical axis denotes the inverse standard error for MR estimate of each SNP. The three vertical lines (colors) represent the causal effect estimated by IVW, MR-Egger (SIMEX) and weighted median methods.

Abbreviations: IV, instrumental variable; IVW, inverse variance weighted; MR, Mendelian randomisation; SIMEX, simulation extrapolation; SE, standard error; SNP, single nucleotide polymorphism.


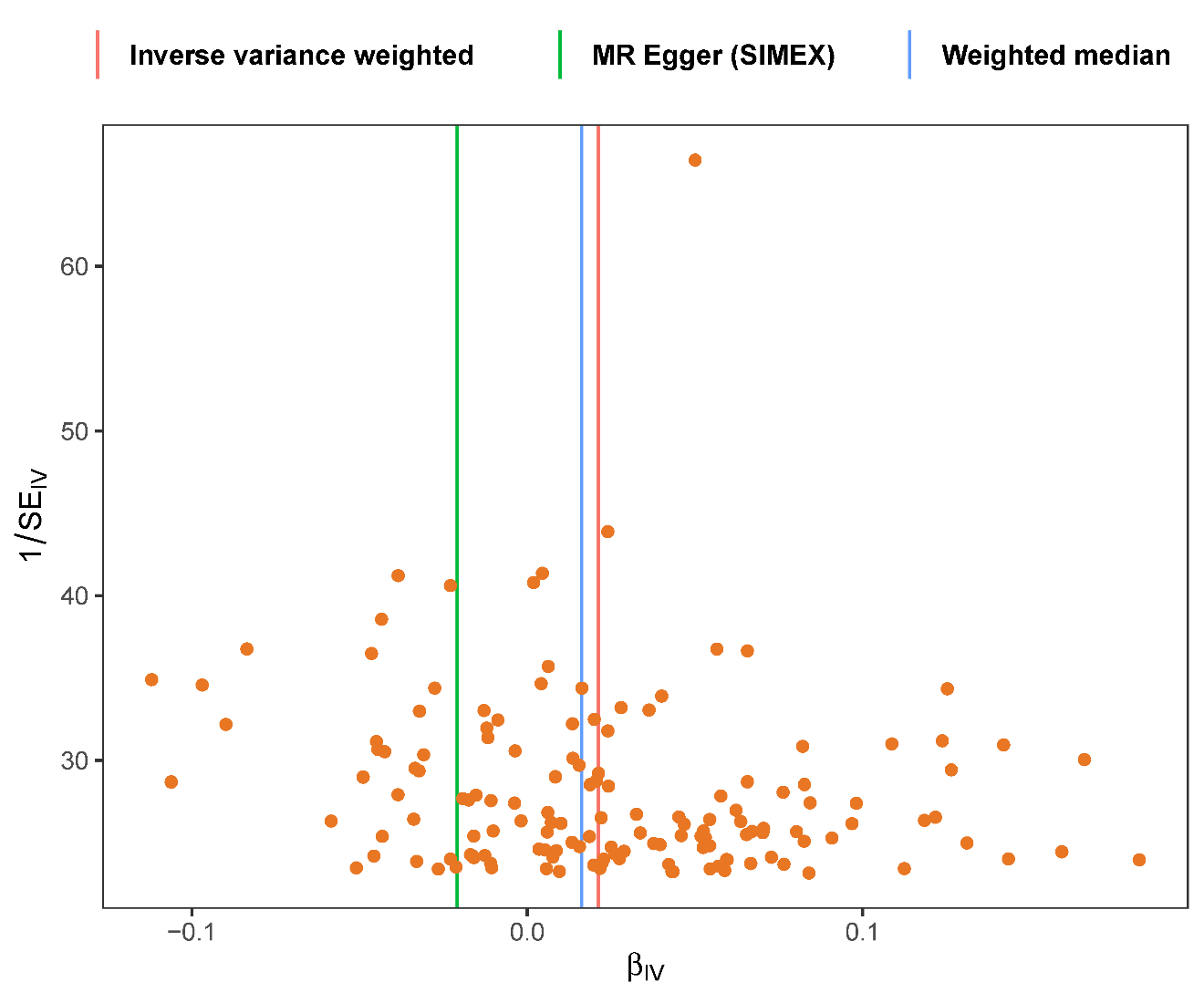


## **Fig. S6. Funnel plot for the causal effect of schizophrenia on TDI.**

In the funnel plot, each dot (orange) represents a SNP, the horizontal axis denotes the MR estimate for each SNP, and the vertical axis denotes the inverse standard error for MR estimate of each SNP. The three vertical lines (colors) represent the causal effect estimated by, MR-Egger (SIMEX) and weighted median methods.

Abbreviations: IV, instrumental variable; IVW, inverse variance weighted; MR, Mendelian randomisation; SIMEX, simulation extrapolation; SE, standard error; SNP, single nucleotide polymorphism; TDI, Townsend deprivation index.


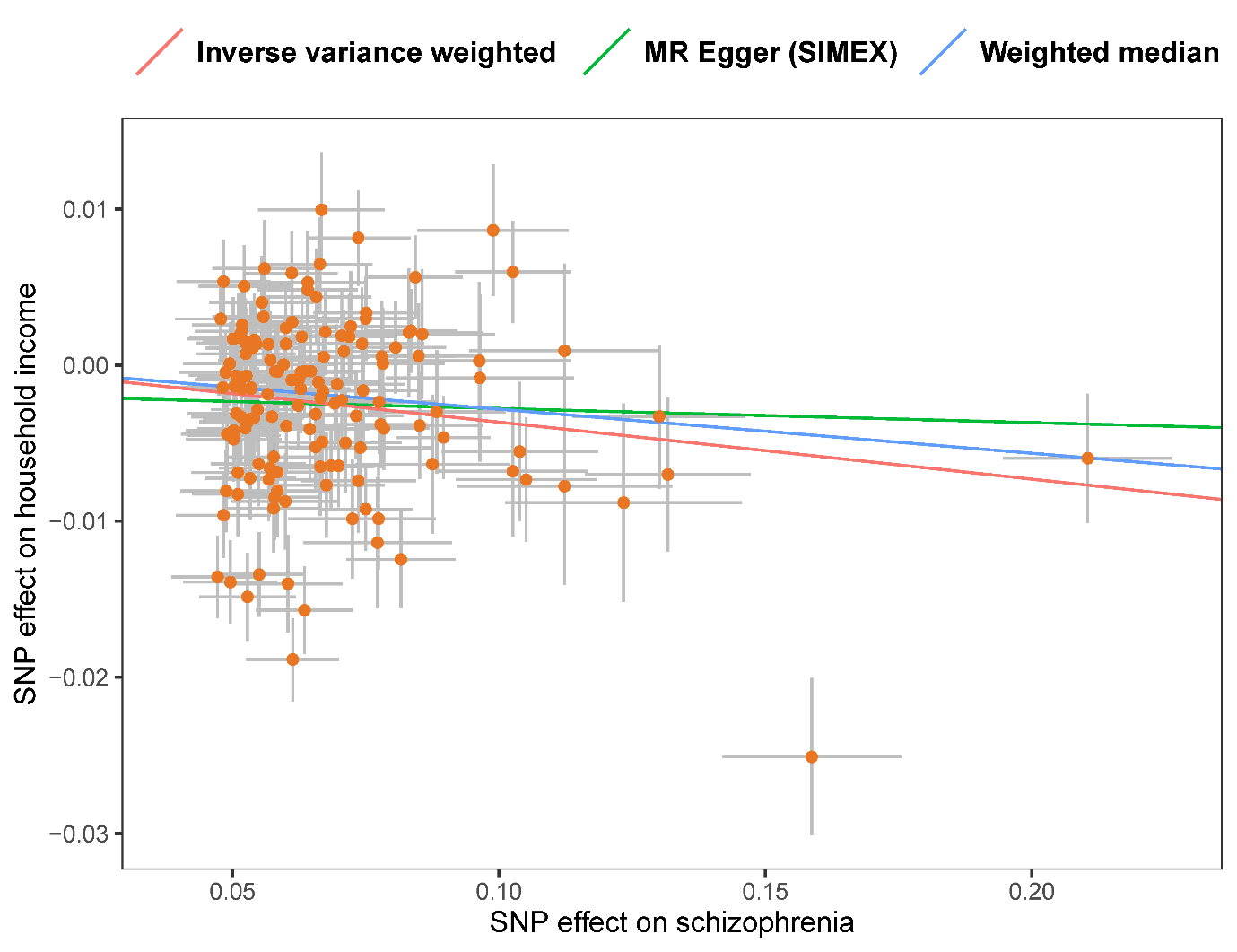


## **Fig. S**7. Scatter plot for the causal effect of schizophrenia on household income.

In the scatter plot, each dot (orange) represents a SNP, the error bars (grey) at each dot represent the 95% confidence intervals. The horizontal axis is the SNP effect on exposure (schizophrenia), while the vertical axis is the SNP effect on outcome (household income). The three fitted lines (colors) represent the results of MR under three methods (shown in the top panel), with the slope of each line corresponding to the estimated causal effect for each method.

Abbreviations: IVW, inverse variance weighted; MR, Mendelian randomisation; SIMEX, simulation extrapolation; SNP, single nucleotide polymorphism.


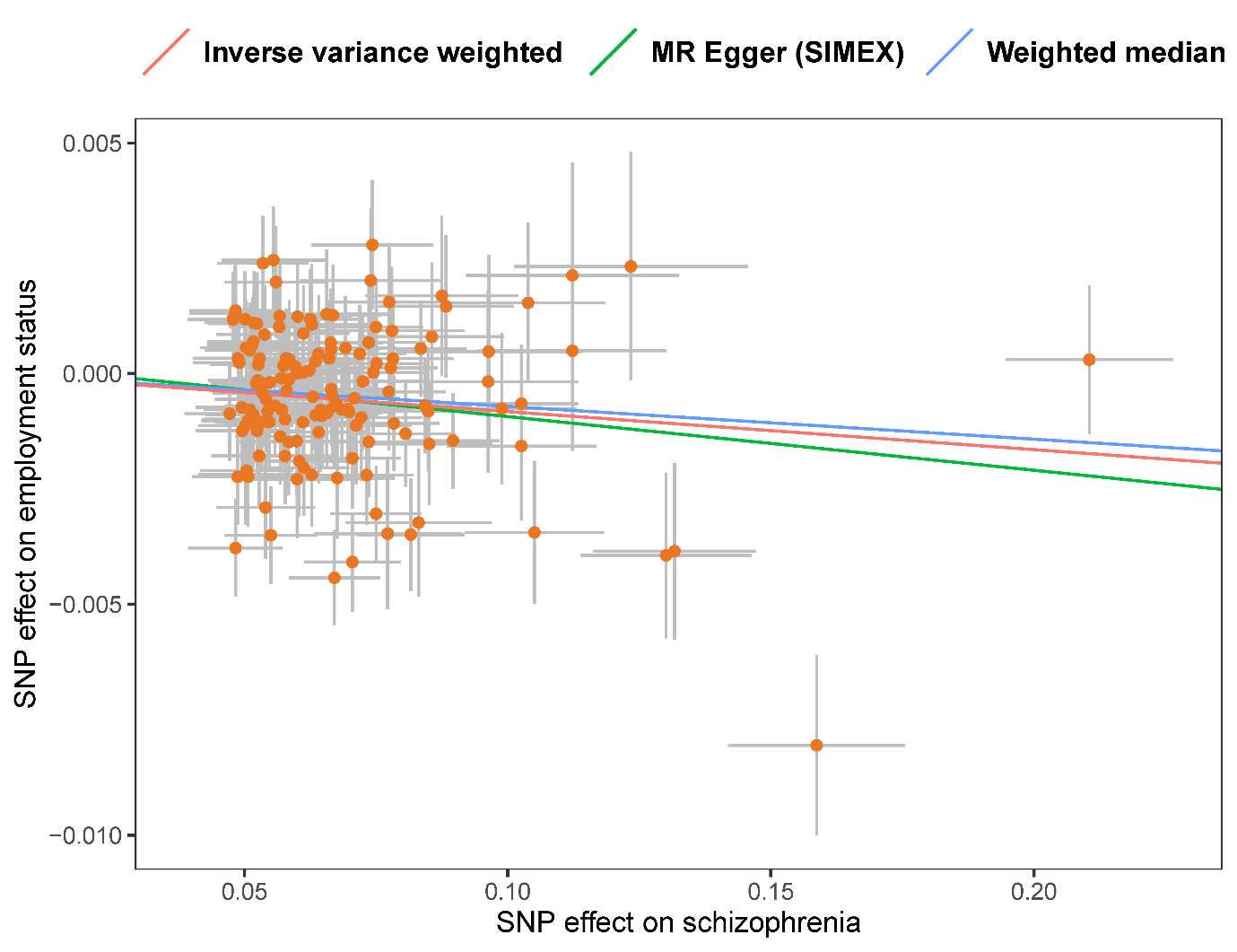


## **Fig. S**8. Scatter plot for the causal effect of schizophrenia on employment status.

In the scatter plot, each dot (orange) represents a SNP, the error bars (grey) at each dot represent the 95% confidence intervals. The horizontal axis is the SNP effect on exposure (schizophrenia), while the vertical axis is the SNP effect on outcome (employment status). The three fitted lines (colors) represent the results of MR under three methods (shown in the top panel), with the slope of each line corresponding to the estimated causal effect for each method.

Abbreviations: IVW, inverse variance weighted; MR, Mendelian randomisation; SIMEX, simulation extrapolation; SNP, single nucleotide polymorphism.


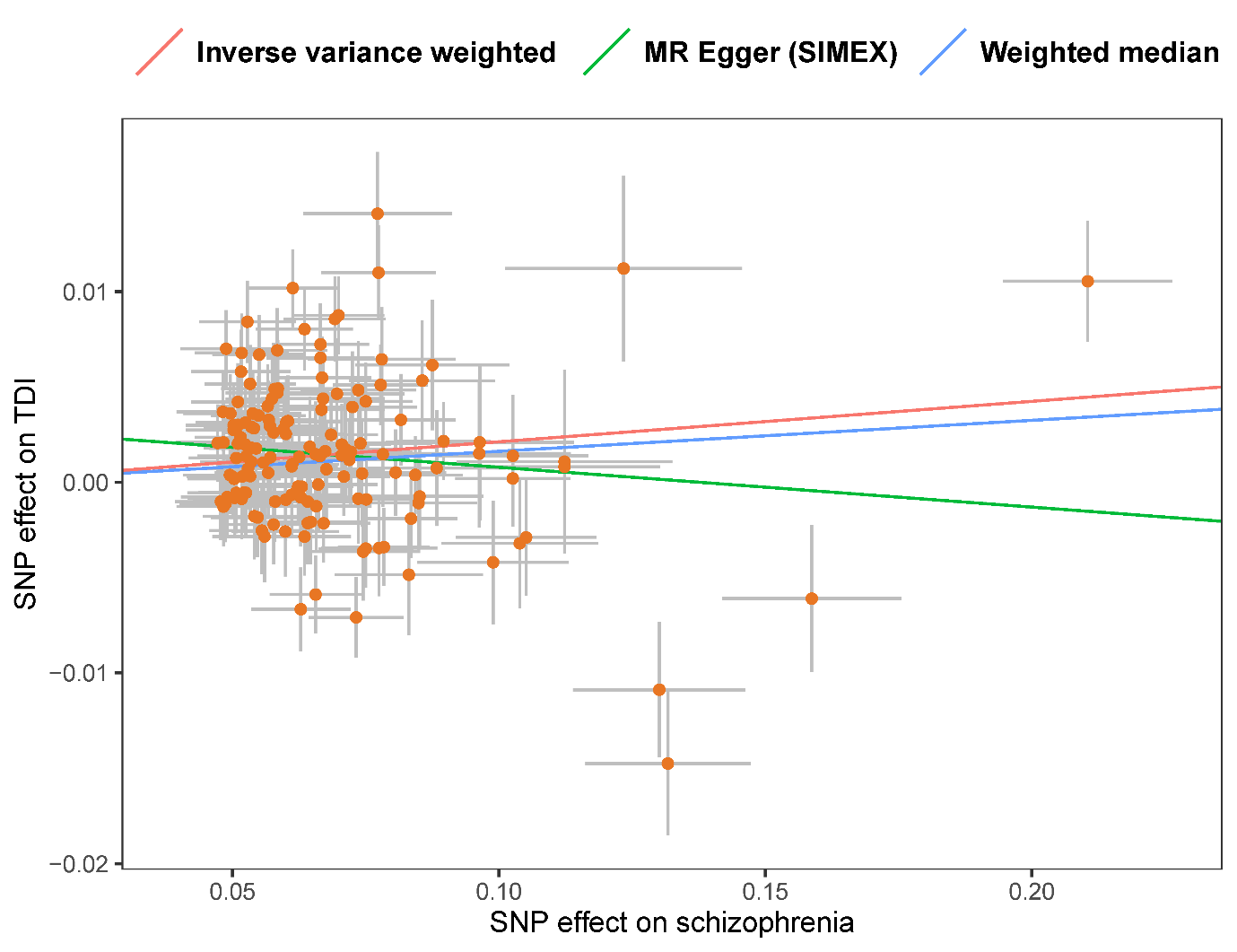


## **Fig. S**9. Scatter plot for the causal effect of schizophrenia on TDI.

In the scatter plot, each dot (orange) represents a SNP, the error bars (grey) at each dot represent the 95% confidence intervals. The horizontal axis is the SNP effect on exposure (schizophrenia), while the vertical axis is the SNP effect on outcome (TDI). The three fitted lines (colors) represent the results of MR under three methods (shown in the top panel), with the slope of each line corresponding to the estimated causal effect for each method.

Abbreviations: IVW, inverse variance weighted; MR, Mendelian randomisation; SIMEX, simulation extrapolation; SNP, single nucleotide polymorphism; TDI, Townsend deprivation index.

# References

1 Bycroft, C., Freeman, C., Petkova, D., Band, G., Elliott, L. T., Sharp, K. et al. The UK Biobank resource with deep phenotyping and genomic data. *Nature* **562**, 203-209, doi:10.1038/s41586-018-0579-z (2018).

2 O'Connell, J., Sharp, K., Shrine, N., Wain, L., Hall, I., Tobin, M. et al. Haplotype estimation for biobank-scale data sets. *Nature genetics* **48**, 817-820, doi:10.1038/ng.3583 (2016).

3 Howie, B., Marchini, J. & Stephens, M. Genotype imputation with thousands of genomes. *G3* **1**, 457-470, doi:10.1534/g3.111.001198 (2011).

4 Huang, J., Howie, B., McCarthy, S., Memari, Y., Walter, K., Min, J. L. et al. Improved imputation of low-frequency and rare variants using the UK10K haplotype reference panel. *Nature communications* **6**, 8111, doi:10.1038/ncomms9111 (2015).

5 Mitchell, R., Hemani, G., Dudding, T. & Paternoster, L. UK biobank genetic data: mrc-ieu quality control, version 1. *University of Bristol* (2017).

6 Loh, P. R., Tucker, G., Bulik-Sullivan, B. K., Vilhjalmsson, B. J., Finucane, H. K., Salem, R. M. et al. Efficient Bayesian mixed-model analysis increases association power in large cohorts. *Nature genetics* **47**, 284-290, doi:10.1038/ng.3190 (2015).

7 Loh, P. R., Kichaev, G., Gazal, S., Schoech, A. P. & Price, A. L. Mixed-model association for biobank-scale datasets. *Nature genetics* **50**, 906-908, doi:10.1038/s41588-018-0144-6 (2018).

8 Sanderson, E., Spiller, W. & Bowden, J. Testing and correcting for weak and pleiotropic instruments in two-sample multivariable Mendelian randomization. *Statistics in medicine* **40**, 5434-5452, doi:10.1002/sim.9133 (2021).

9 Burgess, S. Sample size and power calculations in Mendelian randomization with a single instrumental variable and a binary outcome. *Int J Epidemiol* **43**, 922-929, doi:10.1093/ije/dyu005 (2014).

10 Lee, J. J., Wedow, R., Okbay, A., Kong, E., Maghzian, O., Zacher, M. et al. Gene discovery and polygenic prediction from a genome-wide association study of educational attainment in 1.1 million individuals. *Nat Genet* **50**, 1112-1121, doi:10.1038/s41588-018-0147-3 (2018).

11 Hemani, G., Zheng, J., Elsworth, B., Wade, K. H., Haberland, V., Baird, D. et al. The MR-Base platform supports systematic causal inference across the human phenome. *Elife* **7**, doi:10.7554/eLife.34408 (2018).

12 Elsworth, B., Lyon, M., Alexander, T., Liu, Y., Matthews, P., Hallett, J. et al. The MRC IEU OpenGWAS data infrastructure. *bioRxiv*, 2020.2008.2010.244293, doi:10.1101/2020.08.10.244293 (2020).

13 Trubetskoy, V., Pardiñas, A. F., Qi, T., Panagiotaropoulou, G., Awasthi, S., Bigdeli, T. B. et al. Mapping genomic loci implicates genes and synaptic biology in schizophrenia. *Nature* **604**, 502-508, doi:10.1038/s41586-022-04434-5 (2022).

14 Wray, N. R., Ripke, S., Mattheisen, M., Trzaskowski, M., Byrne, E. M., Abdellaoui, A. et al. Genome-wide association analyses identify 44 risk variants and refine the genetic architecture of major depression. *Nat Genet* **50**, 668-681, doi:10.1038/s41588-018-0090-3 (2018).
